# Supplementary material for: Synthesis of metalla-dual-azulenes with fluoride ion recognition properties
Source: Nat Commun. 2023 Sep 11;14:5583. doi: 10.1038/s41467-023-41250-5 (PMC10495402; doi:10.1038/s41467-023-41250-5)
Supplement: Supplementary file 1 — Supplementary Information [file 41467_2023_41250_MOESM1_ESM.pdf]

Supplementary Information

Synthesis of Metalla-Dual-Azulenes with Fluoride Ion Recognition Properties

Hai-Cheng Liu,[1,#] Kaidong Ruan,[1,#] Kexin Ma,[1] Jiawei Fei,[1] Yu-Mei Lin,[1]* Haiping Xia[1,2]*

[1]State Key Laboratory of Physical Chemistry of Solid Surfaces, College of Chemistry and Chemical Engineering, Xiamen University, Xiamen, Fujian 361005, China

[2]Shenzhen Grubbs Institute, Department of Chemistry, Southern University of Science and Technology, Shenzhen 518055, China

[#]These authors contributed equally: H.-C. Liu, K. Ruan

*Corresponding Authors: linyum@xmu.edu.cn (Y.-M. Lin); hpxia@xmu.edu.cn (H. Xia)

Table of Contents

Supplementary methods 3

1. General information 3

2. Synthesis and characterization of complex **1** 5

3 General preparation procedure and characterization of complexes **2a-2h** 9

4. Separation and characterization of intermediate **3a** 15

5. General preparation procedure and characterization of complexes **4a-4j**, **5a**, **6a**, **6b** 17

6. Separation and characterization of complex **7a** 30

7. DFT calculation of the NICS, ACID, CDD, NRT and others. 35

8. UV/Vis absorption spectra of complexes **4a-4j** 39

9. The cyclic voltammetry (CV) experiments of complexes **2a-2c**, **2h**, **4c**, **4f**, and **6b** 39

10. Thermal stability tests 40

11. X-ray crystallographic analysis 42

12. Computational methods 68

13. NMR and HRMS Spectra 69

Supplementary references 118

Supplementary methods

1. General information

Unless stated otherwise, all syntheses and manipulations were carried out under an atmosphere of high purity nitrogen using Schlenk line techniques or a glove box. All solvents were dried over sodium/benzophenone (*n*-hexane and diethyl ether) or calcium hydride (dichloromethane) and distilled under N_2 prior to use. All other reagents were purchased from commercial suppliers (TCI, Aldrich, Alfa and J&K) and used without further purification. Column chromatography was performed using silica gel (200-300 mesh), neutral aluminum oxide (200-300 mesh) or basic aluminum oxide (200-300 mesh) in air. NMR spectra was recorded at 298 K using a Bruker Advance III 500 spectrometer (^{31}P, 202.5 MHz; ^{1}H, 500.2 MHz; ^{13}C, 125.8 MHz;). The ^{1}H and ^{13}C{^{1}H} NMR chemical shifts (δ) were measured relative to tetramethylsilane, and the ^{31}P{^{1}H} NMR chemical shifts are relative to 85% H_3PO_4. The absolute values of the coupling constants are given in hertz (Hz). Assignments of signals in ^{1}H and ^{13}C NMR spectra were done by reference to heteronuclear single quantum coherence (HSQC), heteronuclear multiple bond correlation (HMBC), and distortionless enhancement by polarization transfer (DEPT) NMR spectra. Multiplicities are abbreviated as singlet (s), doublet (d), triplet (t), multiplet (m), and broad (br). High-resolution mass spectrometry (HRMS) was conducted using an Agilent 1290-6545XT. Absorption spectra were recorded on an Agilent Cary 5000 UV-Vis spectrophotometer (**1**, **2a-2d**, **2g**, **3a**, **4a-4j**, **5a**, **6a**, **6b**, **7a**) or Shimadzu UV-2700 UV-Vis spectrophotometer (**2e**, **2f**, **2h**). Gas phasic products (H_2) were quantified by gas chromatography with a thermal conductivity detector (TCD, column: TDX-01 and 5Å molecular sieve, GC7900). Cyclic Voltammetry (CV) was performed with a CHI660E electrochemical workstation in anhydrous CH_2Cl_2 containing $^{n}Bu_4NBF_4$ (0.1 M) as supporting electrolyte. All potentials were recorded versus Ag/AgCl (saturated) as a reference electrode. The scan rate was 0.1 Vs^{-1}. Single-crystal X-ray diffraction data were collected on a Rigaku XtaLAB Synergy, Dualflex, Rigaku XtaLAB Synergy-S diffractometer coupled to a RigakuHypix detector with Cu Kα radiation (λ = 1.54184 Å) or Mo Kα radiation (λ = 0.71073 Å). The structures were realised with the SHELXT solution

program by using Olex 2 as the graphical interface. The model was refined using Least Squares minimisation with the 2018/3 version of the program SHELXL.[1] Non-H atoms were refined anisotropically unless otherwise stated. The disordered solvents were removed using the SOLVENT MASK routine of Olex 2. The nucleus-independent chemical shifts (NICS)[2-4] and anisotropy of the induced current density (ACID)[5-6] calculations were performed at the B3LYP/6-31G* level. The Condensed dual descriptor (CDD)[7] were performed at the B3LYP-D3BJ/6-311G**. The effective core potentials (ECPs) of Hay and Wadt with a double-ζ valence basis set LanL2DZ[8,9] for **2a**' was used to describe Ir and P atoms. Polarization functions were added for Ir ($\zeta(f) = 0.938$), P ($\zeta(d) = 0.340$).[10,11] All calculations were performed with the Gaussian 16 software package.[12]

2. Synthesis and characterization of complex 1

2.1 Preparation and characterization of diyne compound L

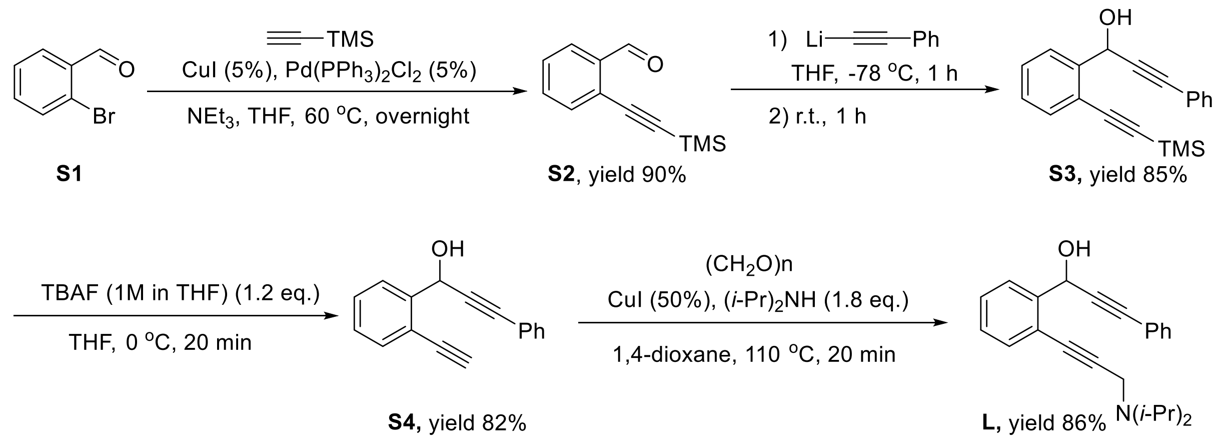

Preparation and characterization of compound S2[13]:

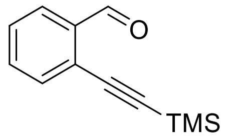

S2, yield 90%

To a 250 ml flask containing $Pd(PPh_3)_2Cl_2$ (5 mol%, 1.40 g) and copper iodide (5 mol%, 0.40 g) and 2-bromobenzaldehyde (7.41 g, 40.0 mmol) and dry THF (100.0 mL) was added (trimethylsilyl)acetylene (6.82 mL, 48.0 mmol) and Et_3N (50.0 mL), then the mixture was stirred at 60 °C overnight under nitrogen. After the reaction was complete, the mixture was filtered and the filter cake was washed by THF (2 × 20.0 mL), and the collected filtrate was concentrated. Then the residue was chromatographed by silica column with hexane/EtOAc (30:1) to give the desired product **S2** in yellow oil. Yield: 90%. ^{1}H NMR (500.0 MHz, $CDCl_3$) δ = 10.56 (s, 1H), 7.91 (d, J = 7.8 Hz, 1H), 7.56-7.54 (m, 2H), 7.43 (t, J = 7.8 Hz, 1H), 0.29 ppm (s, 9H, $C(CH_3)_3$); ^{13}C NMR (125.8 MHz, $CDCl_3$) δ = 191.8, 136.2, 133.7, 133.5, 128.8, 126.9, 126.7, 102.4, 100.1, 0.10. The spectra were consistent with those previously reported.

Preparation and characterization of compound S3[13]:

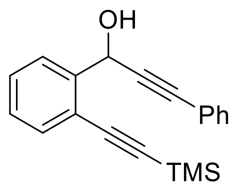

S3, yield 85%

To an oven-dried flask containing **S2** (8.07 g, 40.0 mmol) in anhydrous tetrahydrofuran (200.0 mL) was dropwise added Lithium phenylacetylide solution (48.0 mL, 1.0 M in THF, 48.0 mmol) over 40 min under N_2 at –78 °C. After the reaction mixture was stirred for an additional 20 min under –78 °C, the mixture was then warmed to rt for another 1 h. The reaction was quenched with saturated aqueous NH_4Cl (100.0 mL), the resulting mixture was extracted with Et_2O (3 ×100.0 mL), and the extract was washed with saturated brine (3 × 100.0 mL), dried over $MgSO_4$, and concentrated to dryness. The residue was chromatographed by silica column with hexane/EtOAc (30:1) to afford **S3** in yellow oil. Yield: 85%. ^{1}H NMR (500.2 MHz, $CDCl_3$): δ = 7.76 (d, J = 7.5 Hz, 1H, *C1*H), 7.55-7.49 (m, 3H, *C2/C3/C4*H), 7.42-7.29 (m, 5H, *Ph*H), 6.09 (d, J = 6.0 Hz, 1H, C8H), 3.05 (d, J = 6.0 Hz, 1H, C8OH), 0.31 (s, 9H, $C(CH_3)_3$). ^{13}C{^{1}H} NMR (125.8 MHz, $CDCl_3$): δ = 142.9, 132.8, 131.8, 129.2, 128.5, 128.3, 128.2.7, 126.7, 122.6, 121.2, 102.4, 100.7, 88.1, 86.5, 63.8, -0.10. The spectra were consistent with those previously reported.

Preparation and characterization of compound S4[13]

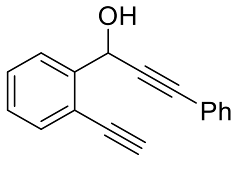

S4, yield 82%

To a solution of **S3** (6.1 g, 20.0 mmol) in tetrahydrofuran (100.0 mL), a solution of $^{n}Bu_4NF$ (1.0 M in THF, 24.0 mL, 24.0 mmol) was added at 0 °C; the mixture was stirred at the same temperature until the starting material disappeared (20 min). The mixture was then poured into a saturated aqueous solution of NH_4Cl (100.0 mL), extracted with Et_2O (3 × 100.0 mL), and washed with saturated brine (3 × 50.0 mL). The organic layer was dried over $MgSO_4$ and concentrated to dryness. The residue was chromatographed by silica column with hexane/EtOAc

(10:1) to afford **S4** in yellow oil. Yield: 82%.^{1}H NMR (500.2 MHz, $CDCl_3$): δ = 7.83 (d, *J* = 7.1 Hz, 1H), 7.58 (dd, J_1 = 7.6 Hz, J_2 = 1.1 Hz, 1H), 7.52-7.49 (m, 2H), 7.45 (t, *J* = 7.6 Hz, 1H), 7.35-7.32 (m, 4H), 6.14 (d, *J* = 4.4 Hz, 1H, CH(OH)), 3.44 (s, 1H, C≡CH), 2.90 (d, *J* = 4.4 Hz, 1H, CH(OH)); ^{13}C{^{1}H} NMR (125.8 MHz, $CDCl_3$): δ = 142.9, 132.2, 131.8, 129.5, 128.6, 128.3, 126.9, 122.5, 120.4, 88.3, 86.6, 82.9, 81.1, 63.3. The spectra were consistent with those previously reported.

Preparation and characterization of compound L:

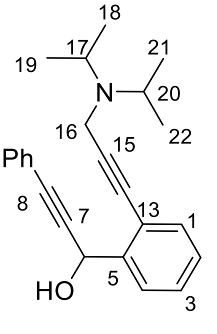

L, yield 86%

To a 100 mL flask containing **S4** (1.16 g, 5.0 mmol) and copper iodide (5 mol%, 0.05 g) and paraformaldehyde (0.75 g, 25.0 mmol) and 1,4-dioxane (30.0 mL) was added $(i\text{-Pr})_2NH$ (1.26 mL, 9.0 mmol), then the mixture was stirred at 110 °C for 20 min under nitrogen. The mixture was then poured into an ice-water mixture. extracted with Et_2O (3 × 100.0 mL) and washed with saturated brine (3 × 50.0 mL). The organic layer was dried over $MgSO_4$ and concentrated to dryness. Then the residue was chromatographed by silica column with hexane/EtOAc (2:1) to give the desired product **L** in reddish-brown solid, Yield: 86%. ^{1}H NMR (500.2 MHz, $CDCl_3$): δ = 7.79 (d, *J* = 7.5 Hz, 1H, *C1*H), 7.49-7.46 (m, 3H, *C2/C3/C4*H), 7.39-7.29 (m, 5H, *Ph*H), 6.09 (s, C6H), 3.73 (s, *C16*H), 3.32-3.27 (m, 2H, *C17/20*H), 1.18 (d, *J* = 6.5 Hz, 12H, *C18*H,*19*H,*21H,22*H); ^{13}C{^{1}H} NMR (125.8 MHz, $CDCl_3$): δ = 142.3, 132.5, 131.8, 128.5, 128.4, 128.2, 128.1, 126.7, 122.7, 121.9, 94.9, 88.6, 86.3, 81.0, 63.5 (s, C6), 48.6 (s, C16), 34.9 (s, C17/C20), 20.6 (C18, C19, C21, C22); IR (film): ν (cm^{-1}) 3061, 2970, 1653, 1598, 1489, 1466, 1444, 1387, 1332, 1175, 1137, 1036, 967, 756, 691; HRMS (ESI): (*m/z*) Calcd for $[C_{24}H_{27}NO+H^{+}]^{+}$ requires 346.2165, Found 346.2406.

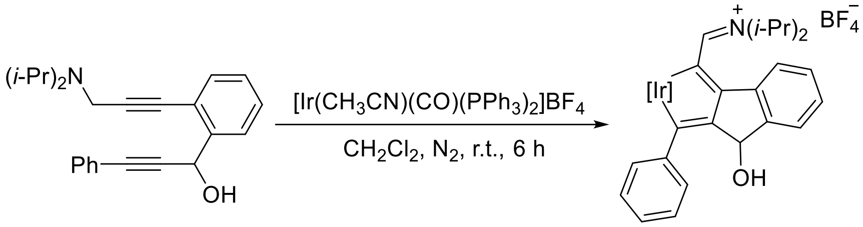

L

[Ir] = IrH(CO)(PPh$_3$)$_2$
1, yield 81%

2.2 Synthesis and characterization of complex 1

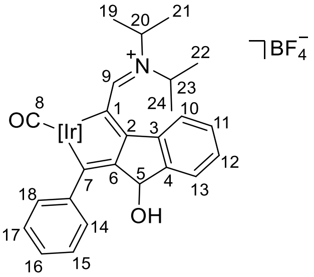

[Ir] = IrH(PPh$_3$)$_2$
1

To a 25 mL Schlenk tube containing [Ir(CH$_3$CN)(CO)(PPh$_3$)$_2$]BF$_4$ (1.11 g, 1.27 mmol) and **L** (0.39 g, 1.15 mmol) was added CH$_2$Cl$_2$ (5.0 mL), then the mixture was stirred at room temperature for 6 h under nitrogen to give a brown solution. The residue was purified by column chromatography (neutral alumina, eluent: dichloromethane/acetone = 2:1) to afford complex **1** in pink solid, yield: 81%. ^{1}H NMR (500.2 MHz, CD$_2$Cl$_2$): δ = 7.78 (s, 1H, *C9*H), 5.10 (d, J = 5.5 Hz, 1H, *C5*H), 4.79-4.75 (m, 1H, *C20*H), 3.75-3.71 (m, 1H, *C23*H), 1.54 (d, J = 5.5 Hz, 1H, *C5*OH), 1.13 (d, J = 6.5 Hz, 3H, *C19*H), 0.99 (t, J = 6.0 Hz, 6H, *C22*H, *C24*H), 0.64 (d, J = 6.5 Hz, 3H, *C21*H), -9.95 (t, J = 14.5 Hz, 1H, *Ir*H); ^{31}P{^{1}H} NMR (202.5 MHz, CD$_2$Cl$_2$): δ = -0.68 (dd, J_1 = 303.9 Hz, J_2 = 397.9 Hz, *Ir*PPh$_3$); ^{13}C{^{1}H} NMR (125.8 MHz, CD$_2$Cl$_2$, plus ^{13}C-dept 135, ^{1}H -^{13}C HSQC and ^{1}H-^{13}C HMBC): δ =182.9 (s, C7), 180.4 (s, C1), 172.3 (t, J = 8.8 Hz, C8), 171.4 (s, C9), 170.1 (s, C6), 161.8 (s, C4), 152.2 (s, C3), 148.9 (s, C14), 137.0 (s, C13), 123.6 (s, C10), 69.4 (s, C5), 53.5 (s, C20), 51.3 (s, C23), 23.7 (s, C19), 23.3 (s, C21), 20.2 (s, C22), 18.6 (s, C24); HRMS (ESI): (*m/z*) Calcd for [C$_{61}$H$_{57}$IrNO$_2$P$_2$]$^+$ requires 1090.3493, Found 1090.3516.

2.3 The possible mechanism for the formation of compound 1

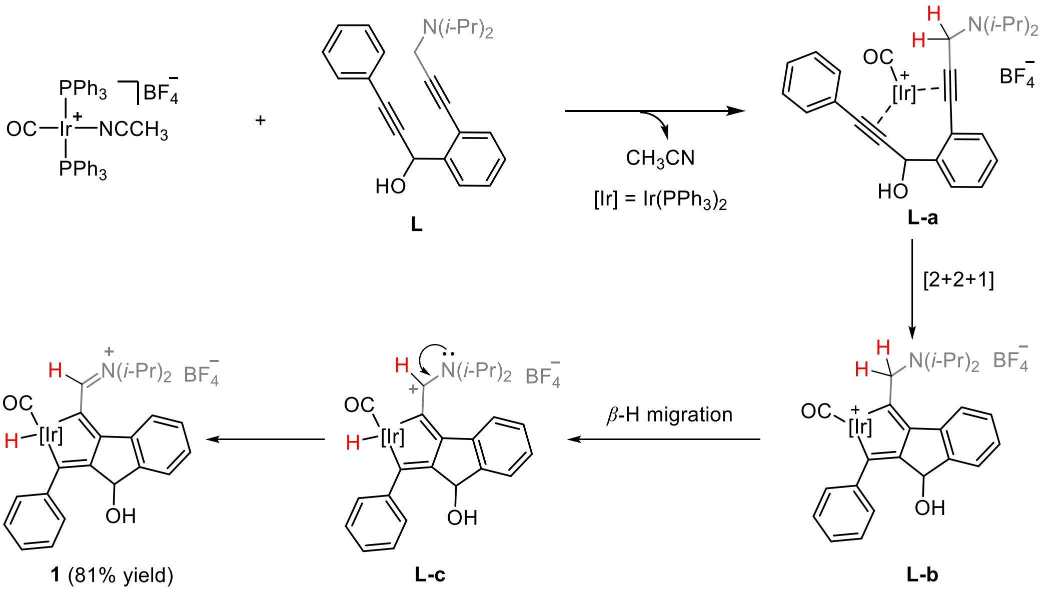

Supplementary Figure 1. The proposed mechanism for the formation of compound **1**.

The process begins with the dissociation of CH_3CN in $[Ir(CH_3CN)(CO)(PPh_3)_2]BF_4$, then coordination of diyne chain **L** to iridium center affords species **L-a**. Next, intramolecular [2+2+1] cycloaddition within **L-a** generates species **L-b**. Subsequently, *β*-H migration process occurs to form species **L-c**[14,15]and finally resulted in the formation of stable compound **1**.

3 General preparation procedure and characterization of complexes 2a-2h

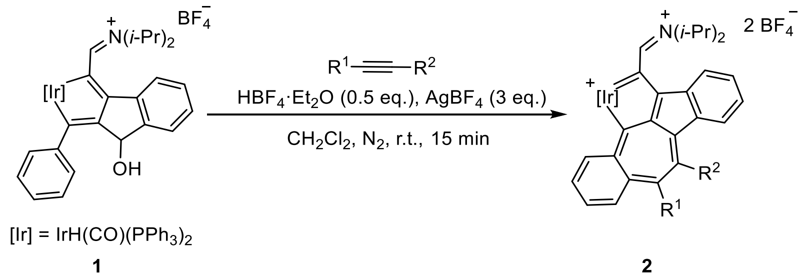

A mixture of **1** (235.4 mg, 0.20 mmol) and $AgBF_4$ (116.4 mg, 0.60 mmol) in CH_2Cl_2 (2.0 mL) was added alkyne (0.24 mmol) and $HBF_4{\cdot}Et_2O$ (0.10 mmol) was stirred at room temperature under nitrogen for 15 min to give a brown solution. Then the solution was filtered by Celite, and the filtrate was concentrated under vacuum. The resulting residue was purified by column chromatography (silica gel (200–300 mesh), eluent: dichloromethane/acetone = 2:1) to afford complexes **2**.

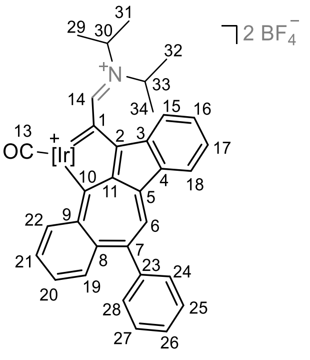

[Ir] = IrH(PPh$_3$)$_2$

2a

According to general procedure starting from **1** (235.4 mg, 0.20 mmol), phenylacetylene (25.8 μL, 0.24 mmol), the product **2a** was obtained in orange red solid (yield: 87%). ^{1}H NMR (500.2 MHz, CD_2Cl_2): δ = 8.54 (s, 1H, *C14*H), 8.30 (d, *J* = 7.5Hz, 1H, *C18*H), 8.12 (d, *J* = 8.5 Hz, 1H, *C19*H), 8.04 (d, *J* = 8.0Hz, 1H, *C22*H), 6.69 (d, *J* = 7.5 Hz, 1H, *C6*H), 3.38-3.30 (m, 1H, *C30*H), 4.33-4.23 (m, 1H, *C33*H), -11.09 (t, *J* = 12Hz, 1H, *Ir*H); ^{31}P{^{1}H} NMR (202.5 MHz, CD_2Cl_2): δ = -2.6 (s, *Ir*PPh$_3$); ^{13}C{^{1}H} NMR (125.8 MHz, CD_2Cl_2, plus ^{13}C-dept 135, ^{1}H -^{13}C HSQC and ^{1}H-^{13}C HMBC): δ = 227.3 (s, C10), 176.6 (s, C1), 175.3 (s, C2), 171.1 (t, *J* = 10.1Hz, C13), 163.9 (s, C8), 161.3 (s, C7), 152.0 (s, C5), 147.8 (s, C9), 145.5 (s, C22), 143.4 (s, C11), 136.9 (s, C19), 126.4 (s, C18), 122.6 (s, C6), 59.4 (s, C30), 54.6 (s, C33); HRMS (ESI): (*m/z*) Calcd for [$C_{69}H_{60}IrNOP_2$-H^+]$^+$ requires 1172.3702, Found 1172.3739.

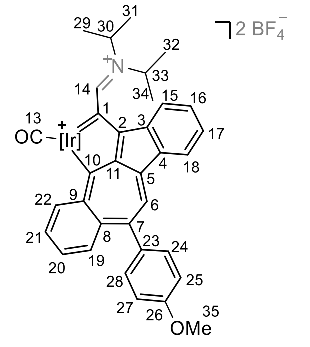

[Ir] = IrH(PPh$_3$)$_2$

2b

According to general procedure starting from **1** (235.4 mg, 0.20 mmol), 4-methoxyphenylacetylene (31.1 μL, 0.24 mmol), the product **2b** was obtained in orange red solid (yield: 90%).^{1}H NMR (500.2 MHz, CD_2Cl_2): δ = 8.53 (s, 1H, *C14*H), 8.32 (d, *J* = 8.5 Hz, 1H, *C18*H), 8.17 (s, *J* = 8.5 Hz, 1H, *C19*H), 8.00 (d, *J* = 8.0 Hz, 1H, *C22*H), 6.64 (d, *J* = 8.0 Hz, 1H,

*C6*H), 4,28-4.22 (m, 1H, *C30*H), 3.98 (s, 3H, *C35*H), 3.47-3.39 (m, 1H, *C33*H), 1.64-0.89 (m, 12H, *C29*H, *C31*H, *C32*H, *C34*H), -11.05 (t, J = 12.5 Hz, 1H, *Ir*H); $^{31}P\{^1H\}$ NMR (202.5 MHz, CD_2Cl_2): δ = -3.44 (s, *Ir*PPh_3); $^{13}C\{^1H\}$ NMR (125.8 MHz, CD_2Cl_2, plus ^{13}C-dept 135, ^1H -^{13}C HSQC and ^1H-^{13}C HMBC): δ = 223.9 (t, J = 7.4 Hz, C10), 176.5 (s, C1), 171.2 (t, J = 10.2 Hz, C13), 168.8 (s, C2), 164.8 (s, C7), 162.2 (s, C23), 161.8 (t, J = 3.3 Hz, C28), 149.7 (s, C9), 147.6 (s, C5), 144.3 (s, C22), 137.1 (s, C19), 136.9 (s, C8), 135.8 (s, C20), 134.1 (s, C21), 130.2 (s, C14), 126.3 (s, C18), 122.6 (s, C6), 115.0 (s, C27), 59.2 (s, C35), 55.8 (s, C30), 54.5 (s, C33), 23.9 (s, C29, C31), 19.2 (s, C32, C34); HRMS (ESI): (*m/z*) Calcd for $[C_{70}H_{62}IrNO_2P_2\text{-}H^+]^+$ requires 1202.3808, Found 1202.3863.

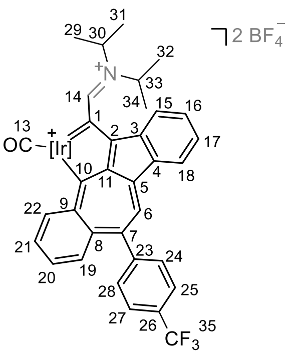

[Ir] = IrH(PPh3)2

2c

According to general procedure starting from **1** (235.4 mg, 0.20 mmol), 4'-trifluoromethylphenyl acetylene (34.2 μL, 0.24 mmol), the product **2c** was obtained in orange red solid (yield: 85%). ^1H NMR (500.2 MHz, CD_2Cl_2): δ = 8.53 (s, 1H, *C14*H), 8.35 (d, J = 7.5 Hz, 1H, *C18*H), 8.04 (d, J = 8.5 Hz, 1H, *C22*H), 6.69 (d, J = 7.5 Hz, 1H, *C6*H), 4.32-4.24 (m, 1H, *C30*H), 3.32-3.24 (m, 1H, *C33*H), 1.63-0.89 (m, 12H, *C29*H, *C31*H, *C32*H, *C34*H), -11.13 (t, J = 12 Hz, 1H, *Ir*H); $^{31}P\{^1H\}$ NMR (202.5 MHz, CD_2Cl_2): δ = -2.90 (s, *Ir*PPh_3); $^{13}C\{^1H\}$ NMR (125.8 MHz, CD_2Cl_2, plus ^{13}C-dept 135, ^1H -^{13}C HSQC and ^1H-^{13}C HMBC): δ = 229.3 (s, C10), 176.6 (s, C1), 171.0 (t, J = 10.6 Hz, C13), 169.8 (s, C11), 161.2 (s, C8), 160.9 (s, C28), 153.1 (s, C7), 149.6 (s, C9), 147.9 (s, C5), 146.8 (s, C3), 146.3 (s, C2), 144.3 (s, C22), 136.5 (s, C4), 136.2 (s, C17), 126.7 (s, C18), 126.2 (q, J = 7.3 Hz, C25), 122.5 (s, C6), 59.5 (s, C30), 54.6 (s, C33), 23.9, 19.3; HRMS (ESI): (*m/z*) Calcd for $[C_{70}H_{59}F_3IrNOP_2\text{-}H^+]^+$ requires 1240.3576, Found 1240.3651.

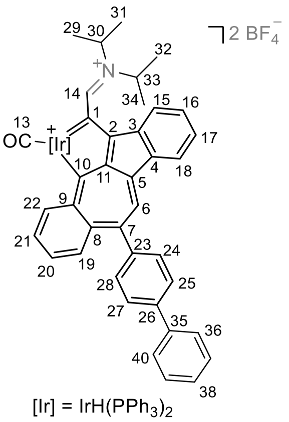

[Ir] = $IrH(PPh_3)_2$

2d

According to general procedure starting from **1** (235.4 mg, 0.20 mmol), 4-ethynylbiphenyl (43.8 mg, 0.24 mmol), the product **2d** was obtained in orange red solid (yield: 83%). ^{1}H NMR (500.2 MHz, CD_2Cl_2): δ = 8.59 (s, 1H, *C14*H), 8.33 (d, *J* = 7.5 Hz, 1H, *C18*H), 8.22 (d, *J* = 8.5 Hz, 1H, *C19*H), 8.05 (d, *J* = 8.5 Hz, 1H, *C22*H), 6.69 (d, *J* = 7.5 Hz, 1H, *C6*H), 4.31-4.24 (m, 1H, *C30*H), 3.41-3.35 (m, 1H, *C33*H), 1.32-0.90 (m, 12H, *C29*H, *C31*H, *C32*H, *C34*H), -11.07 (t, *J* = 12.0 Hz, 1H, *Ir*H); $^{31}P\{^{1}H\}$ NMR (202.5 MHz, CD_2Cl_2): δ = -2.98 (s, *Ir*PPh_3); $^{13}C\{^{1}H\}$ NMR (125.8 MHz, CD_2Cl_2, plus ^{13}C-dept 135, ^{1}H -^{13}C HSQC and ^{1}H-^{13}C HMBC): δ = 237.9 (s, C14), 176.5 (s, C22), 171.1 (s, C13), 169.3 (s, C1), 163.8 (s, C9), 161.4 (s, C8), 149.7 (s, C7), 147.7 (s, C5), 145.3 (s, C22), 144.3 (s, C21), 136.9 (s, C19), 136.7 (s, C2), 126.4 (s, C18), 122.6 (s, C6), 59.4 (s, C30), 54.6 (s, C33); HRMS (ESI): *(m/z)* Calcd for $[C_{75}H_{64}IrNOP_2\text{-}H^+]^+$ requires 1248.4016, Found 1248.4079.

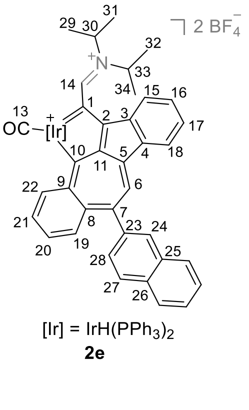

[Ir] = $IrH(PPh_3)_2$

2e

According to general procedure starting from **1** (235.4 mg, 0.20 mmol), 2-ethynyl-naphthalene (36.5 mg, 0.24 mmol), product **2e** was obtained in orange red solid (yield: 86%). ^{1}H NMR (500.2 MHz, CD_2Cl_2): δ = 8.73 (s, 1H, *C14*H), 8.39 (d, *J* = 8.0Hz, 1H, *C22*H), 6.74 (d, *J* = 7.5 Hz, 1H,

*C6*H), 4.36-4.29 (m, 1H, *C30*H), 3.46-3.41 (m, 1H, *C33*H), 1.37 (d, *J* = 6.5 Hz, 6H, C29H, C31H), 0.96 (d, *J* = 7.0 Hz, 6H, C32H, C34H), -11.01 (t, *J* = 12Hz, 1H, *Ir*H); $^{31}P\{^1H\}$ NMR (202.5 MHz, CD_2Cl_2): δ = -2.5 (s, *Ir*PPh_3); $^{13}C\{^1H\}$ NMR (125.8 MHz, CD_2Cl_2, plus ^{13}C-dept 135, ^1H -^{13}C HSQC and ^1H-^{13}C HMBC): δ = 226.9 (s, C10), 176.6 (s, C1), 175.2 (s, C2), 171.1 (t, *J* = 10.1Hz, C13), 164.1 (s, C8), 161.5 (s, C7), 149.8 (s, C5), 147.7 (s, C9), 145.3 (s, C22), 144.4 (s, C11), 137.1 (s, C19), 126.5 (s, C18), 122.6 (s, C6), 59.3 (s, C30), 54.6 (s, C33), 23.9 (s, C29, C31), 19.3 (s, C32, C34); HRMS (ESI): (*m/z*) Calcd for $[C_{73}H_{62}IrNOP_2]^{2+}$, requires 611.6966, Found 611.7025.

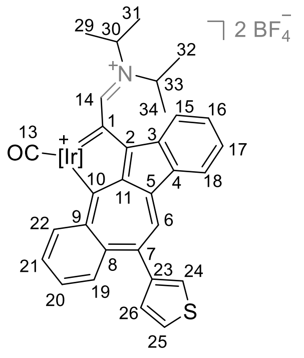

[Ir] = $IrH(PPh_3)_2$

2f

According to general procedure starting from **1** (235.4 mg, 0.20 mmol), 3-ethynylthiophene (23.6 *μ*L, 0.24 mmol), product **2f** was obtained in orange red solid (yield: 68%). ^1H NMR (500.2 MHz, CD_2Cl_2): δ = 8.72 (s, 1H, *C14*H), 8.44 (d, *J* = 8.5Hz, 1H, *C22*H), 8.32 (d, *J* = 8.5 Hz, 1H, *C19*H), 6.68 (d, *J* = 5.5 Hz, 1H, *C6*H), 4.33-4.28 (m, 1H, *C30*H), 3.39-3.34 (m, 1H, *C33*H), 1.34 (d, *J* = 7.0 Hz, 6H, C29H, C31H), 0.96 (d, *J* = 6.5 Hz, 6H, C32H, C34H), -11.03 (t, *J* = 12Hz, 1H, *Ir*H); $^{31}P\{^1H\}$ NMR (202.5 MHz, CD_2Cl_2): δ = -2.63 (s, *Ir*PPh_3); $^{13}C\{^1H\}$ NMR (125.8 MHz, CD_2Cl_2, plus ^{13}C-dept 135, ^1H -^{13}C HSQC and ^1H-^{13}C HMBC): δ = 225.1 (s, C10), 176.6 (s, C1), 171.2 (t, *J* = 10.1Hz, C13), 169.0 (s, C2), 161.4(s, C8), 158.8 (s, C7), 151.1 (s, C5), 149.4 (s, C9), 147.9 (s, C22), 144.8 (s, C11), 136.4 (s, C19), 126.6 (s, C18), 122.4 (s, C6), 59.3 (s, C30), 54.5 (s, C33), 23.9 (s, C29, C31), 19.2 (s, C32, C34); HRMS (ESI): (*m/z*) Calcd for $[C_{67}H_{58}IrNOSP_2]^{2+}$, requires 589.6668, Found 589.6724.

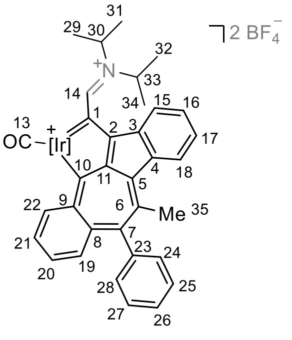

[Ir] = IrH(PPh3)2

2g

According to general procedure starting from **1** (235.4 mg, 0.20 mmol), 1-phenyl-1-propyne (29.7 *μ*L, 0.24 mmol), product **2g** was obtained in orange red solid (yield: 82%). ^{1}H NMR (500.2 MHz, CD_2Cl_2): δ = 8.19 (d, *J* = 8.0 Hz, 1H, *C18*H), 7.97 (d, *J* = 8.5 Hz, 1H, *C22*H), 7.83 (s, 1H, *C14*H), 6.55 (d, *J* = 7.5 Hz, 1H, *C19*H), 4.31-4.27 (m, 1H, *C30*H), 3.98-3.95 (m, 1H, *C33*H), 2.66 (s, 3H, *C35*H), 1.35 (d, *J* = 6.5 Hz, 6H, *C29/31*H), 0.92 (d, *J* = 6.5 Hz, 6H, *C32/34*H), -10.84 (t, *J* = 12.5 Hz, 1H, *Ir*H); ^{31}P{^{1}H} NMR (202.5 MHz, CD_2Cl_2): δ = -0.94 (s, *Ir*PPh_3); ^{13}C{^{1}H} NMR (125.8 MHz, CD_2Cl_2, plus ^{13}C-dept 135, ^{1}H -^{13}C HSQC and ^{1}H-^{13}C HMBC): δ = 222.6 (s, C10), 176.1 (s, C14), 170.8 (t, *J* = 9.9 Hz, C13), 170.5 (s, C1), 161.8 (s, C7), 153.8 (s, C5), 149.6 (s, C2), 145.3 (s, C9), 143.3 (s, C22), 142.2 (s, C11), 137.9 (s, C3), 136.1 (s, C18), 122.9 (s, C19), 59.0 (s, C30), 54.8 (s, C33), 26.9 (s, C35), 23.8 (s, C29/31), 18.8 (s, C32/34); HRMS (ESI): (*m/z*) Calcd for $[C_{70}H_{62}IrNOP_2\text{-}H]^+$ requires 1186.3859, Found 1186.2895.

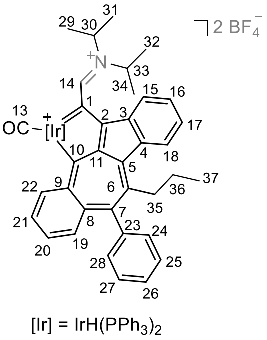

[Ir] = IrH(PPh3)2

2h

According to general procedure starting from **1** (235.4 mg, 0.20 mmol), 1-phenyl-1-pentyne (37.6 *μ*L, 0.24 mmol), product **2h** was obtained in orange red solid (yield: 84%). ^{1}H NMR (500.2 MHz, CD_2Cl_2): δ = 8.42 (d, *J* = 8.5 Hz, 1H, *C18*H), 7.85 (d, *J* = 8.5 Hz, 1H, *C22*H), 7.73 (s, 1H, *C14*H),

6.47 (d, *J* = 8.0 Hz, 1H, *C19*H), 4.29-4.24 (m, 1H, *C30*H), 3.25 (t, *J* = 8.0 Hz, 2H, *C35*H), 3.21-3.16 (m, 1H, *C33*H), 1.53-1.47 (m, 2H, *C36*H), 1.33 (d, *J* = 6.5 Hz, 6H, *C29/31*H), 0.88 (d, *J* = 6.5 Hz, 6H, *C32/34*H), 0.76 (t, *J* = 7.5 Hz, 3H, *C37*H), -10.99 (t, *J* = 12.5 Hz, 1H, *Ir*H); ^{31}P{^{1}H} NMR (202.5 MHz, CD_2Cl_2): δ = -1.88 (s, *Ir*PPh_3); ^{13}C{^{1}H} NMR (125.8 MHz, CD_2Cl_2, plus ^{13}C-dept 135, ^{1}H -^{13}C HSQC and ^{1}H-^{13}C HMBC): δ = 222.9 (s, C10), 176.6 (s, C14), 171.1 (t, *J* = 10.1 Hz, C13), 170.2 (s, C1), 161.2 (s, C7), 159.6 (s, C5), 150.9 (s, C2), 147.8 (s, C9), 145.1 (s, C22), 142.5 (s, C11), 137.9 (s, C3), 127.7 (s, C18), 122.5 (s, C19), 59.4 (s, C30), 54.6 (s, C33), 38.1 (s, C35), 24.6 (s, C36), 23.9 (s, C29/31), 19.2 (s, C32/34), 14.0 (s, C37); HRMS (ESI): (*m/z*) Calcd for $[C_{72}H_{66}IrNOP_2]^{2+}$ requires 607.7122, Found 607.7172.

4. Separation and characterization of intermediate 3a

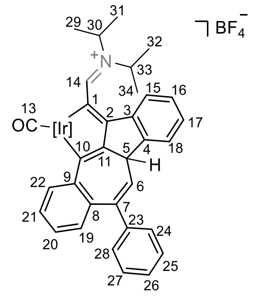

[Ir] = $IrH(PPh_3)_2$

3a

A mixture of **1** (235.4 mg, 0.20 mmol) and phenylacetylene (25.8 *μ*L, 0.24 mmol) in CH_2Cl_2 (2.0 mL) was added $HBF_4{\cdot}Et_2O$ (0.60 mmol) and then stirred at room temperature under nitrogen for 15 min to give a brown solution. Then the solution was concentrated under vacuum. The resulting residue was purified by column chromatography (alumina (200-300 mesh), eluent: dichloromethane/acetone = 2:1) to afford complex **3a** in pink solid. Yield: 93%. ^{1}H NMR (500.2 MHz, CD_2Cl_2): δ = 7.99 (s, 1H, C14*H*), 5.27-5.21 (m, 1H, C30*H*), 5.03 (d, *J* = 4.5 Hz, 1H, C6*H*), 3.87-3.81 (m, 1H, C33*H*), 3.28 (d, *J* = 4.5 Hz, 1H, C5*H*), 1.43 (d, *J* = 7.0 Hz, 3H, C29*H*), 1.18 (d, *J* = 6.0 Hz, 3H, C32*H*), 0.83 (d, *J* = 7.0 Hz, 3H, C31*H*), 0.64 (d, *J* = 6.0 Hz, 3H, C34*H*), -10.00 (t, *J* = 13.5 Hz, 1H, Ir*H*); ^{31}P{^{1}H} NMR (202.5 MHz, CD_2Cl_2): δ = 3.63 (dd, J_1 = 318.9 Hz, J_2 = 384.3 Hz, Ir*PPh_3*); ^{13}C{^{1}H} NMR (125.8 MHz, CD_2Cl_2, plus ^{13}C-dept 135, ^{1}H -^{13}C HSQC and ^{1}H-^{13}C HMBC): δ = 186.8 (s, C14), 175.6 (s, C1), 173.4 (t, *J*(PC) = 8.8 Hz, C13),

170.4 (s, C2), 167.7 (s, C3), 153.9 (s, C4), 148.7 (s, C8), 143.7 (s, C9), 138.2 (s, C7), 136.6 (s, C6), 122.4 (s, C10), 53.1 (s, C33), 51.3 (s, C30), 43.7 (s, C5); HRMS (ESI): (*m/z*) Calcd for $[C_{69}H_{61}IrNOP_2]^+$ requires 1174.3858, Found 1174.3900.

Observation of silver mirror in the vessel after reaction.

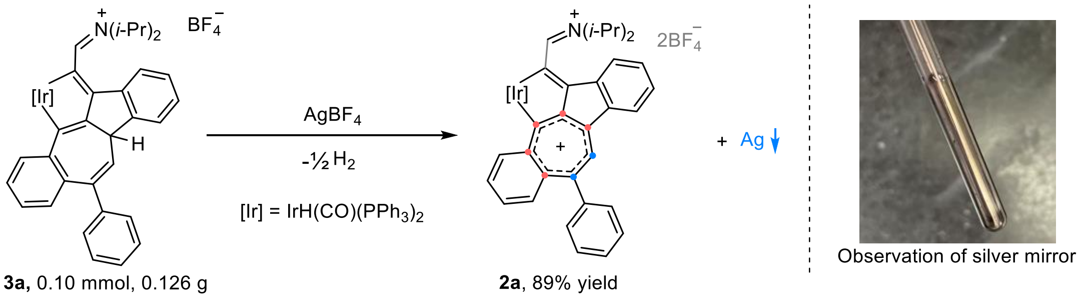

Supplementary Figure 2. The reaction of isolated **3a** with $AgBF_4$ and the silver mirror phenomenon.

Treatment of the isolated **3a** with $AgBF_4$ in CH_2Cl_2 led to the corresponding **2a**. Silver mirror can be observed in the vessel after the reaction, suggesting the conversation of **3a** to **2a** underwent oxidative dehydrogenation enabled by $AgBF_4$.

The effect of temperature on the reaction.

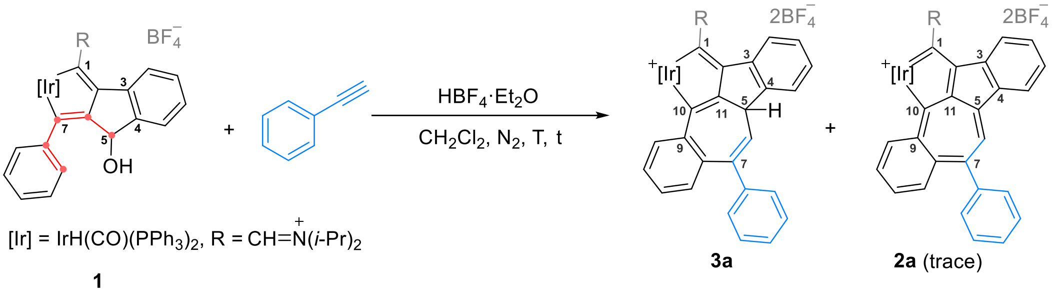

Supplementary Figure 3. The reaction of **1** with phenylacetylene in the absence of $AgBF_4$.

As shown in Supplementary Figure 3 and 4, control experiments involving the reaction of compound **1** reacts with phenylacetylene in the presence of $HBF_4{\cdot}Et_2O$, heated to 80 °C for 24 h, even to 7 days, remain led to mainly product **3a**, like the results obtained at room temperature. We consider the competitive process from species **Int3** to **3a** through **TS3** exhibiting an extremely low energy barrier (0.1 kcal mol^{-1}), consequently, this process is predominantly governed by kinetic factors rather than thermodynamic considerations.

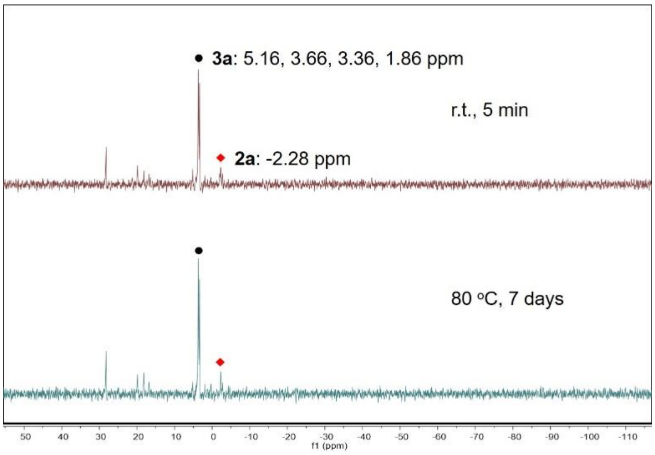

Supplementary Figure 4. The *in situ* $^{31}P\{^1H\}$ NMR in the reaction of **1** (23.5 mg, 0.02 mmol) with phenylacetylene (2.6 μL, 1.2 eq.) in the presence of $HBF_4 \cdot Et_2O$ (3.0 eq.).

5. General preparation procedure and characterization of complexes 4a-4j, 5a, 6a, 6b

5.1 Preparation and characterization of complexes 4a-4j.

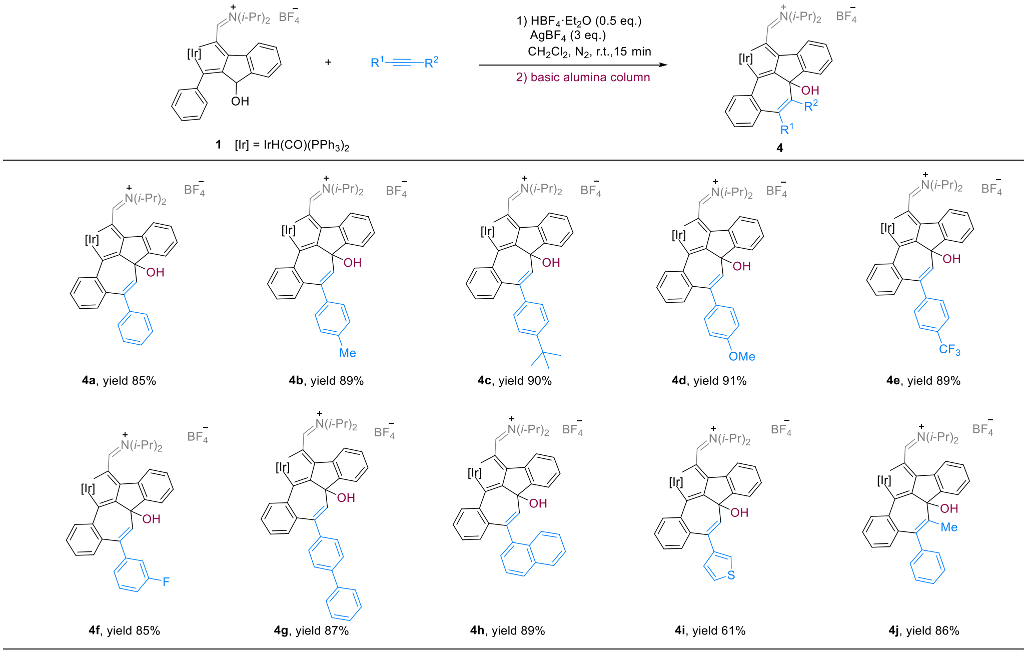

Supplementary Figure 5 Formation of complexes **4a-4j** by reactions of complex **1** with alkynes.

A mixture of **1** (235.4 mg, 0.20 mmol) and $AgBF_4$ (116.4 mg, 0.60 mmol) in CH_2Cl_2 (2.0 mL) was added alkyne (0.24 mmol) and $HBF_4 \cdot Et_2O$ (0.10 mmol) was stirred at room temperature under nitrogen for 15 min to give a brown solution. Then the solution was filtered by Celite, and

the filtrate was concentrated under vacuum. The resulting residue was purified by column chromatography (basic alumina (200–300 mesh), eluent: dichloromethane/acetone = 2:1) to afford complexes **4**.

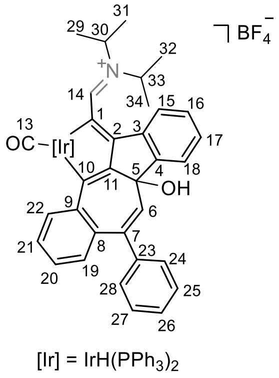

4a

According to general procedure starting from **1** (235.4 mg, 0.20 mmol), phenylacetylene (25.8 μL, 0.24 mmol), the product **4a** was obtained in orange red solid (yield: 85%). ^{1}H NMR (500.2 MHz, CD_2Cl_2): δ = 7.72 (s, 1H, *C14*H), 5.66 (s, 1H, *C6*H), 5.27-5.22 (m, 1H, *C30*H), 3.88-3.83 (m, 1H, *C33*H), 2.33 (br s, 1H, *C5*OH), 1.29 (d, J = 6.5 Hz, 3H, *C29*H), 1.02 (d, J = 6.5 Hz, 3H, *C31*H), 0.94 (d, J = 7.0 Hz, 3H, *C32*H), 0.71 (d, J = 7.0 Hz, 3H, *C34*H), -10.34 (t, J = 14.5 Hz, 1H, *Ir*H); ^{31}P{^{1}H} NMR (202.5 MHz, CD_2Cl_2): δ = -0.53 (dd, J_1 = 305.6 Hz, J_2 = 1009.3 Hz, *Ir*PPh_3); ^{13}C{^{1}H} NMR (125.8 MHz, CD_2Cl_2, plus ^{13}C-dept 135, ^{1}H -^{13}C HSQC and ^{1}H-^{13}C HMBC): δ = 210.8 (s, C10), 184.7 (s, C1), 173.1 (t, J = 9.8 Hz, C13), 169.9 (s, C14), 167.5 (s, C11), 155.5 (s, C4), 146.7 (s, C9), 143.8 (s, C8), 141.9 (s, C19), 135.0 (s, C6), 134.9 (s, C7), 123.9 (s, C15), 72.6 (s, C5), 52.9 (s, C30), 51.7 (s, C33), 24.0 (s, C29), 23.1 (s, C31), 20.2 (s, C32), 18.9 (s, C34); HRMS (ESI): (*m/z*) Calcd for $[C_{69}H_{61}IrNO_2P_2]^+$ requires 1190.3808, Found 1190.3767.

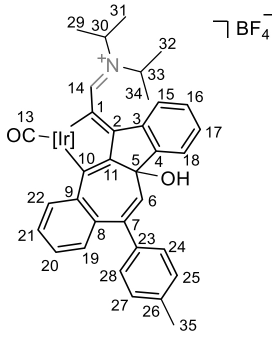

[Ir] = $IrH(PPh_3)_2$

4b

According to general procedure starting from **1** (235.4 mg, 0.20 mmol), 4-ethynyltoluene (29.9 μL, 0.24 mmol), the product **4b** was obtained in orange red solid (yield: 89%). ^{1}H NMR (500.2 MHz, CD_2Cl_2): δ = 7.72 (s, 1H, *C14*H), 6.67 (d, *J* = 7.5 Hz, 1H, *C15*H), 5.63 (s, 1H, *C6*H), 5.28-5.23 (m, 1H, *C30*H), 3.83-3.78 (m, 1H, *C33*H), 2.31 (s, 3H, *C35*H), 2.07 (s, 1H, *C5*OH), 1.29 (d, *J* = 7.0 Hz, 3H, *C29*H), 1.01 (d, *J* = 6.0 Hz, 3H, *C31*H), 0.95 (d, *J* = 7.0 Hz, 3H, *C32*H), 0.70 (d, *J* = 6.0 Hz, 3H, *C34*H), -10.34 (t, *J* = 13.5 Hz, 1H, *Ir*H); ^{31}P{^{1}H} NMR (202.5 MHz, CD_2Cl_2): δ = -0.59 (dd, J_1 = 302.9 Hz, J_2 = 1047.7 Hz, *Ir*PPh_3); ^{13}C{^{1}H} NMR (125.8 MHz, CD_2Cl_2, plus ^{13}C-dept 135, ^{1}H -^{13}C HSQC and ^{1}H-^{13}C HMBC): δ = 184.7 (s, C1), 173.1 (t, *J* = 9.4 Hz, C13), 169.9 (s, C14), 167.6 (s, C11), 155.5 (s, C4), 146.7 (s, C9), 141.9 (s, C8), 140.8 (s, C19), 137.3 (s, C25), 137.2 (s, C26), 134.9 (s, C6), 123.9 (s, C15), 72.7 (s, C5), 52.9 (s, C30), 51.7 (s, C33), 23.9 (s, C29), 23.0 (s, C31), 20.7 (s, C32), 20.2 (s, C35), 18.9 (s, C34); HRMS (ESI): (*m/z*) Calcd for $[C_{70}H_{63}IrNO_2P_2]^+$ requires 1202.3808, Found 1202.3834.

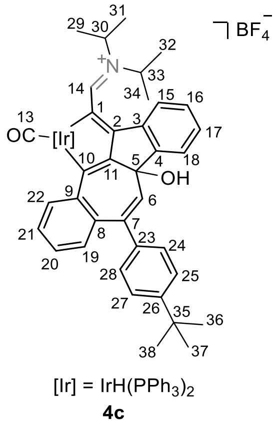

[Ir] = $IrH(PPh_3)_2$

4c

According to general procedure starting from **1** (235.4 mg, 0.20 mmol), 4-(tert-butyl)phenylacetylene (41.7 μL, 0.24 mmol), the product **4c** was obtained in orange red solid

(yield: 90%). ^{1}H NMR (500.2 MHz, CD_2Cl_2): δ = 6.68 (d, *J* = 7.5 Hz, 1H, *C15*H), 5.63 (s, 1H, *C6*H), 5.25-5.19 (m, 1H, *C30*H), 3.84-3.77 (m, 1H, *C33*H), 2.07 (s, 1H, *C5*OH), 1.30-1.27 (m, 12H, *C29*H and *C36-38*H), 1.01 (d, *J* = 6.0 Hz, 3H, *C31*H), 0.95 (d, *J* = 6.5 Hz, 3H, *C32*H), 0.70 (d, *J* = 6.5 Hz, 3H, *C34*H), -10.33 (t, *J* = 15.0 Hz, 1H, *Ir*H); $^{31}P\{^{1}H\}$ NMR (202.5 MHz, CD_2Cl_2): δ = -0.91 (dd, J_1 = 301.7 Hz, J_2 = 893.0 Hz, *Ir*PPh_3); $^{13}C\{^{1}H\}$ NMR (125.8 MHz, CD_2Cl_2, plus ^{13}C-dept 135, ^{1}H -^{13}C HSQC and ^{1}H-^{13}C HMBC): δ = 184.5 (s, C1), 172.9 (t, *J* = 10.1 Hz, C13), 170.1 (s, C14), 167.5 (s, C11), 155.5 (s, C4), 150.7 (s, C37), 146.7 (s, C9), 141.8 (s, C8), 140.2 (s, C19), 134.9 (s, C6), 123.9 (s, C15), 72.7 (s, C5), 51.7 (s, C30), 34.4 (s, C35), 31.0 (s, C36-38), 23.9 (s, C29), 22.9 (s, C31), 20.2 (s, C33), 18.9 (s, C34); HRMS (ESI): (*m/z*) Calcd for $[C_{73}H_{69}IrNO_2P_2]^+$ requires 1244.4278, Found 1244.4301.

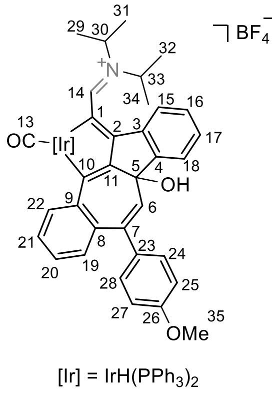

4d

According to general procedure starting from **1** (235.4 mg, 0.20 mmol), 4-methoxyphenylacetylene (31.1 *μ*L, 0.24 mmol), product **4d** was obtained in orange red solid (yield: 91%). ^{1}H NMR (500.2 MHz, CD_2Cl_2): δ = 5.59 (s, 1H, *C6*H), 5.29-5.22 (m, 1H, *C30*H), 3.84-3.73 (m, 4H, *C33*H, *C35*H), 3.77 (s, 3H, *C35*H), 2.06 (br s, 1H, *C5*OH), 1.29 (d, *J* = 6.5 Hz, 3H, *C29*H), 1.01 (d, *J* = 6.0 Hz, 3H, *C31*H), 0.95 (d, *J* = 6.5 Hz, 3H, *C32*H), 0.71 (d, *J* = 6.0 Hz, 3H, *C34*H), -10.33 (t, *J* = 13.0 Hz, 1H, *Ir*H); $^{31}P\{^{1}H\}$ NMR (202.5 MHz, CD_2Cl_2): δ = -0.71 (dd, J_1 = 302.7 Hz, J_2 = 1005.4 Hz, *Ir*PPh_3); $^{13}C\{^{1}H\}$ NMR (125.8 MHz, CD_2Cl_2, plus ^{13}C-dept 135, ^{1}H -^{13}C HSQC and ^{1}H-^{13}C HMBC): δ = 184.2 (s, C1), 173.1 (t, *J* = 8.8 Hz, C13), 169.9 (s, C14), 167.7 (s, C10), 159.2 (s, C26), 155.5 (s, C4), 146.7 (s, C9), 141.5 (s, C19), 135.1 (s, C6), 125.9 (s, C18), 123.9 (s, C15), 113.3 (s, C27), 72.7 (s, C5), 55.2 (s, C35), 52.9 (s, C30), 51.7 (s, C33), 23.9 (s, C29), 23.0 (s, C31), 20.2 (s, C32), 18.9 (s, C34); HRMS (ESI): (*m/z*) Calcd for

$[C_{70}H_{63}IrNO_3P_2]^+$ requires 1220.3913, Found 1220.3927.

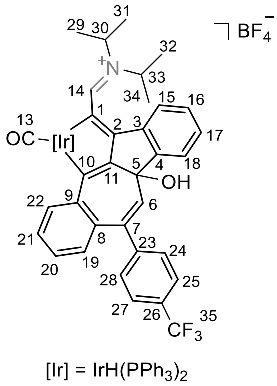

[Ir] = $IrH(PPh_3)_2$
4e

According to general procedure starting from **1** (235.4 mg, 0.20 mmol), 4'-trifluoromethylphenyl acetylene (34.2 μL, 0.24 mmol), product **4e** was obtained in orange red solid (yield: 89%). ^{1}H NMR (500.2 MHz, CD_2Cl_2): δ = 5.75 (s, 1H, *C6*H), 5.22-5.17 (m, 1H, *C30*H), 3.84-3.79 (m, 1H, *C33*H), 2.12 (br s, 1H, *C5*OH), 1.30-1.28 (m, 3H, *C29*H), 1.00-0.98 (m, 3H, *C31*H), 0.96-0.94 (m, 3H, *C32*H), 0.75-0.73 (m, 3H, *C34*H), -10.35 (t, J = 13.5 Hz, 1H, *Ir*H); $^{31}P\{^1H\}$ NMR (202.5 MHz, CD_2Cl_2): δ = -1.14 (dd, J_1 = 302.7 Hz, J_2 = 903.6 Hz, *Ir*PPh_3); $^{13}C\{^1H\}$ NMR (125.8 MHz, CD_2Cl_2, plus ^{13}C-dept 135, ^{1}H -^{13}C HSQC and ^{1}H-^{13}C HMBC): δ = 183.7 (s, C1), 172.9 (t, J = 8.8 Hz, C13), 170.3 (s, C14), 167.2 (s, C11), 155.0 (s, C4), 147.4 (s, C8), 146.7 (s, C19), 136.0 (s, C6), 124.9 (q, J = 7.5 Hz, C26), 123.9 (s, C15), 72.6 (s, C5), 53.1 (s, C30), 51.8 (s, C33), 23.9 (s, C29), 23.0 (s, C31), 20.1 (s, C32), 18.9 (s, C34); HRMS (ESI): (*m/z*) Calcd for $[C_{70}H_{60}F_3IrNO_2P_2]^+$ requires 1256.3525, Found 1256.3545.

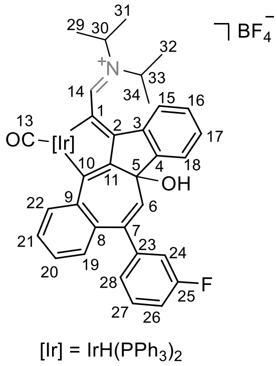

[Ir] = $IrH(PPh_3)_2$
4f

According to general procedure starting from **1** (235.4 mg, 0.20 mmol), 3-fluorophenylacetylene (27.2 μL, 0.24 mmol), product **4f** was obtained in orange red solid (yield: 85%). ^{1}H NMR (500.2

MHz, CD_2Cl_2): δ = 6.64 (d, *J* = 10.5 Hz, 1H, *C26*H), 5.75 (s, 1H, *C6*H), 5.27-5.22 (m, 1H, *C29*H), 3.89-3.83 (m, 1H, *C33*H), 2.13 (br s, 1H, *C5*OH), 1.33 (d, *J* = 7.0 Hz, 3H, *C29*H), 1.04 (d, *J* = 6.5 Hz, 3H, *C31*H), 0.98 (d, *J* = 6.5 Hz, 3H, *C32*H), 0.77 (d, *J* = 7.0 Hz, 3H, *C22*H), -10.30 (t, *J* = 13.0 Hz, 1H, *Ir*H); $^{31}P\{^1H\}$ NMR (202.5 MHz, CD_2Cl_2): δ = -1.14 (dd, J_1 = 301.5 Hz, J_2 = 931.5 Hz, *Ir*PPh3); $^{13}C\{^1H\}$ NMR (125.8 MHz, CD_2Cl_2, plus ^{13}C-dept 135, ^{1}H -^{13}C HSQC and ^{1}H-^{13}C HMBC): δ = 183.8 (s, C1), 172.9 (t, *J* = 8.8 Hz, C13), 170.2 (s, C14), 167.3 (s, C11), 162.45 (d, *J* = 245.3 Hz, C25), 155.1 (s, C4), 146.6 (s, C8), 135.5 (s, C6), 123.9 (s, C15), 115.9 (d, *J* = 22.6 Hz, C26), 114.1 (s, *J* = 20.1 Hz, C27), 72.5 (s, C5), 53.1 (s, C30), 51.8 (s, C33), 23.9 (s, C29), 23.0 (s, C31), 20.1 (s, C32), 18.9 (s, C34); HRMS (ESI): (*m/z*) Calcd for $[C_{69}H_{60}FIrNO_2P_2]^+$ requires 1208.3713, Found 1208.3723.

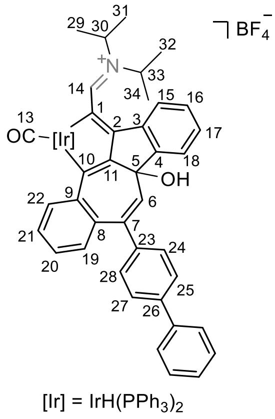

4g

According to general procedure starting from **1** (235.4 mg, 0.20 mmol), 4-ethynylbiphenyl (43.8 mg, 0.24 mmol), product **4g** was obtained in orange red solid (yield: 87%). ^{1}H NMR (500.2 MHz, CD_2Cl_2): δ = 6.69 (d, *J* = 7.5 Hz, 1H, *C15*H), 5.72 (s, 1H, *C6*H), 5.27-5.20 (m, 1H, *C30*H), 3.85-3.79 (m, 1H, *C33*H), 2.12 (br s, 1H, *C5*OH), 1.3 (d, *J* = 7.0 Hz, 3H, *C29*H), 1.01 (d, *J* = 6.5 Hz, 3H, *C31*H), 0.95 (d, *J* = 6.5 Hz, 3H, *C32*H), 0.71 (d, *J* = 7.0 Hz, 3H, *C34*H), -10.32 (t, *J* = 14.0 Hz, 1H, *Ir*H); $^{31}P\{^1H\}$ NMR (202.5 MHz, CD_2Cl_2): δ = -1.39 (dd, J_1 = 302.5 Hz, J_2 = 975.4 Hz, *Ir*PPh3); $^{13}C\{^1H\}$ NMR (125.8 MHz, CD_2Cl_2, plus ^{13}C-dept 135, ^{1}H -^{13}C HSQC and ^{1}H-^{13}C HMBC): δ = 184.4 (s, C1), 173.0 (t, *J* = 10.1 Hz, C13), 170.1 (s, C14), 167.5 (s, C11), 155.4 (s, C4), 146.7 (s, C9), 142.7 (s, C8), 141.2 (s, C19), 134.8 (s, C6), 123.9 (s, C15), 72.7 (s, C5), 51.7 (s, C30), 23.9 (s, C29), 23.0 (s, C31), 20.2 (s, C32), 18.9 (s, C34); HRMS (ESI): (*m/z*) Calcd for

$[C_{75}H_{65}IrNO_2P_2]^+$ requires 1266.4122, Found 1266.4104.

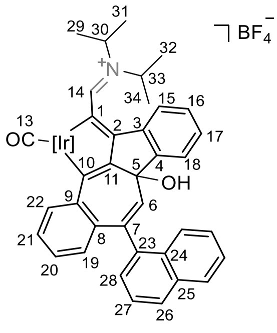

[Ir] = IrH(PPh3)2

4h

According to general procedure starting from **1** (235.4 mg, 0.20 mmol), 1-ethynylnaphthalene (36.5 mg, 0.24 mmol), product **4h** was obtained in orange red solid (yield: 89%). ^{1}H NMR (500.2 MHz, CD_2Cl_2): δ = 5.78 (s, 1H, *C6*H), 5.28-5.21 (m, 1H, *C30*H), 3.87-3.81 (m, 1H, *C33*H), 2.33 (br s, 1H, *C5*OH), 1.31 (d, *J* = 6.5 Hz, 3H, *C29*H), 1.02 (d, *J* = 6.5 Hz, 3H, *C31*H), 0.94 (d, *J* = 6.5 Hz, 3H, *C32*H), 0.70 (d, *J* = 6.5 Hz, 3H, *C34*H), -10.33 (t, *J* = 13.0 Hz, 1H, *Ir*H); ^{31}P{^{1}H} NMR (202.5 MHz, CD_2Cl_2): δ = -0.67 (dd, J_1 = 302.8 Hz, J_2 = 1165.4 Hz, *Ir*PPh$_3$); ^{13}C{^{1}H} NMR (125.8 MHz, CD_2Cl_2, plus ^{13}C-dept 135, ^{1}H -^{13}C HSQC and ^{1}H-^{13}C HMBC): δ = 184.7 (s, C1), 173.1 (t, *J* = 7.5 Hz, C13), 170.1 (s, C14), 167.5 (s, C11), 155.5 (s, C4), 146.8 (s, C9), 141.9 (s, C19), 141.1 (s, C23), 135.7 (s, C6), 134.9 (s, C8), 123.9 (s, C15), 72.7 (s, C5), 52.9 (s, C30), 51.7 (s, C33), 24.1 (s, C29), 23.1 (s, C31), 20.3 (s, C32), 18.9 (s, C34); HRMS (ESI): (*m/z*) Calcd for $[C_{73}H_{63}IrNO_2P_2]^+$ requires 1240.3965, Found 1240.3651.

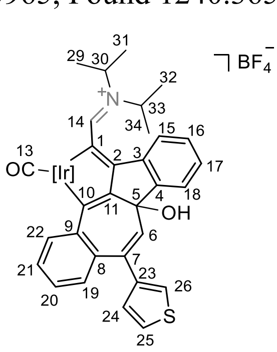

[Ir] = IrH(PPh3)2

4i

According to general procedure starting from **1** (235.4 mg, 0.20 mmol), 3-ethynylthiophene (23.6 μL, 0.24 mmol), product **4i** was obtained in orange red solid (yield: 61%). ^{1}H NMR (500.2 MHz, CD_2Cl_2): δ = 5.65 (s, 1H, *C6*H), 5.23-5.16 (m, 1H, *C30*H), 3.85-3.79 (m, 1H, *C33*H), 2.04 (br s,

1H, *C5*OH), 1.02 (d, *J* = 6.5 Hz, 3H, *C29*H), 0.95 (d, *J* = 6.5 Hz, 3H, *C31*H), 0.89 (d, *J* = 7.0 Hz, 3H, *C32*H), 0.69 (d, *J* = 7.0 Hz, 3H, *C34*H), -10.32 (t, *J* = 14.0 Hz, 1H, *Ir*H); $^{31}P\{^{1}H\}$ NMR (202.5 MHz, CD_2Cl_2): δ = -1.12 (dd, J_1 = 302.9 Hz, J_2 = 714.2 Hz, *Ir*PPh_3); $^{13}C\{^{1}H\}$ NMR (125.8 MHz, CD_2Cl_2, plus ^{13}C-dept 135, ^{1}H -^{13}C HSQC and ^{1}H-^{13}C HMBC): δ = 184.2 (s, C1), 172.9 (t, *J* = 8.8 Hz, C13), 170.4 (s, C14), 167.5 (s, C11), 155.3 (s, C4), 146.5 (s, C9), 144.8 (s, C19), 134.6 (s, C6), 123.0 (s, C15), 72.8 (s, C5), 53.1 (s, C30), 51.7 (s, C33), 23.9 (s, C29), 22.9 (s, C31), 20.3 (s, C32), 18.7 (s, C34); HRMS (ESI): (*m/z*) Calcd for $[C_{67}H_{59}IrNO_2P_2S]^+$ requires 1196.3370, Found 1196.3382.

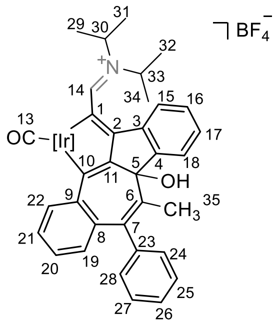

[Ir] = IrH(PPh_3)$_2$

4j

According to general procedure starting from **1** (235.4 mg, 0.20 mmol), 1-phenyl-1-propyne (29.7 *μ*L, 0.24 mmol), product **4j** was obtained in orange red solid (yield: 86%). ^{1}H NMR (500.2 MHz, CD_2Cl_2): δ = 7.79 (d, *J* = 7.5 Hz, 1H, *C18*H), 7.69 (s, 1H, *C14*H), 6.66 (d, *J* = 7.5 Hz, 1H, *C15*H), 5.22-5.17 (m, 1H, *C30*H), 3.91-3.85 (m, 1H, *C33*H), 2.76 (s, 1H, *C5*OH), 1.24 (d, *J* = 6.5 Hz, 3H, *C29*H), 1.20 (s, 3H, *C35*H), 1.00 (d, *J* = 6.5 Hz, 3H, *C31*H), 0.89 (d, *J* = 6.0 Hz, 3H, *C32*H), 0.86 (d, *J* = 6.0 Hz, 3H, *C34*H), -10.16 (t, *J* = 14.5 Hz, 1H, *Ir*H); $^{31}P\{^{1}H\}$ NMR (202.5 MHz, CD_2Cl_2): δ = -2.91 (dd, J_1 = 51.0 Hz, J_2 = 63.9 Hz, *Ir*PPh_3); $^{13}C\{^{1}H\}$ NMR (125.8 MHz, CD_2Cl_2, plus ^{13}C-dept 135, ^{1}H -^{13}C HSQC and ^{1}H-^{13}C HMBC): δ = 182.9 (s, C1), 172.9 (t, *J* = 8.8 Hz, C13), 170.1 (s, C14), 166.5 (s, C11), 154.2 (s, C4), 145.4 (s, C8), 144.3 (s, C19), 139.9 (s, C3), 137.8 (s, C7), 136.7 (s, C6), 123.9 (s, C15), 76.3 (s, C5), 51.5 (s, C33), 23.7 (s, C29), 23.5 (s, C31), 22.9 (s, C35), 19.8 (s, C32), 19.6 (s, C34); HRMS (ESI): (*m/z*) Calcd for $[C_{70}H_{63}IrNO_2P_2]^+$ requires 1204.3964, Found 1204.3978.

5.2 The ^{18}O labeling experiment

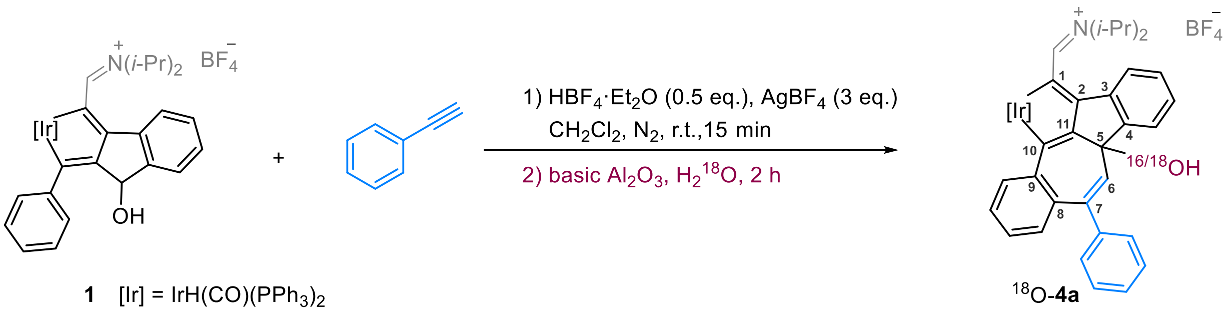

Supplementary Figure 6. Direct synthesis of -OH substituted products **4a** by reactions of **1** with phenylacetylene.

Reaction of **1** with phenylacetylene was carried in the presence of $H_2{}^{18}O$ (Supplementary Figure 6). The ESI-MS spectrum of the resulting product showed a mixture of molecular ions at m/z = 1190.3748 for ^{16}O-**4a** (calculated value $[C_{69}H_{61}IrNO_2P_2]^+$ at m/z = 1190.3808) and 1192.3816 for ^{18}O-**4a** (calculated value $[C_{69}H_{61}IrNO^{18}OP_2]^+$ at m/z = 1192.3849) (Supplementary Figure 7). The result shows that the hydroxyl group (-^{18}OH) attached at C5 of compound **4** derived from H_2O in the system other than the original hydroxyl group of compound **1**.

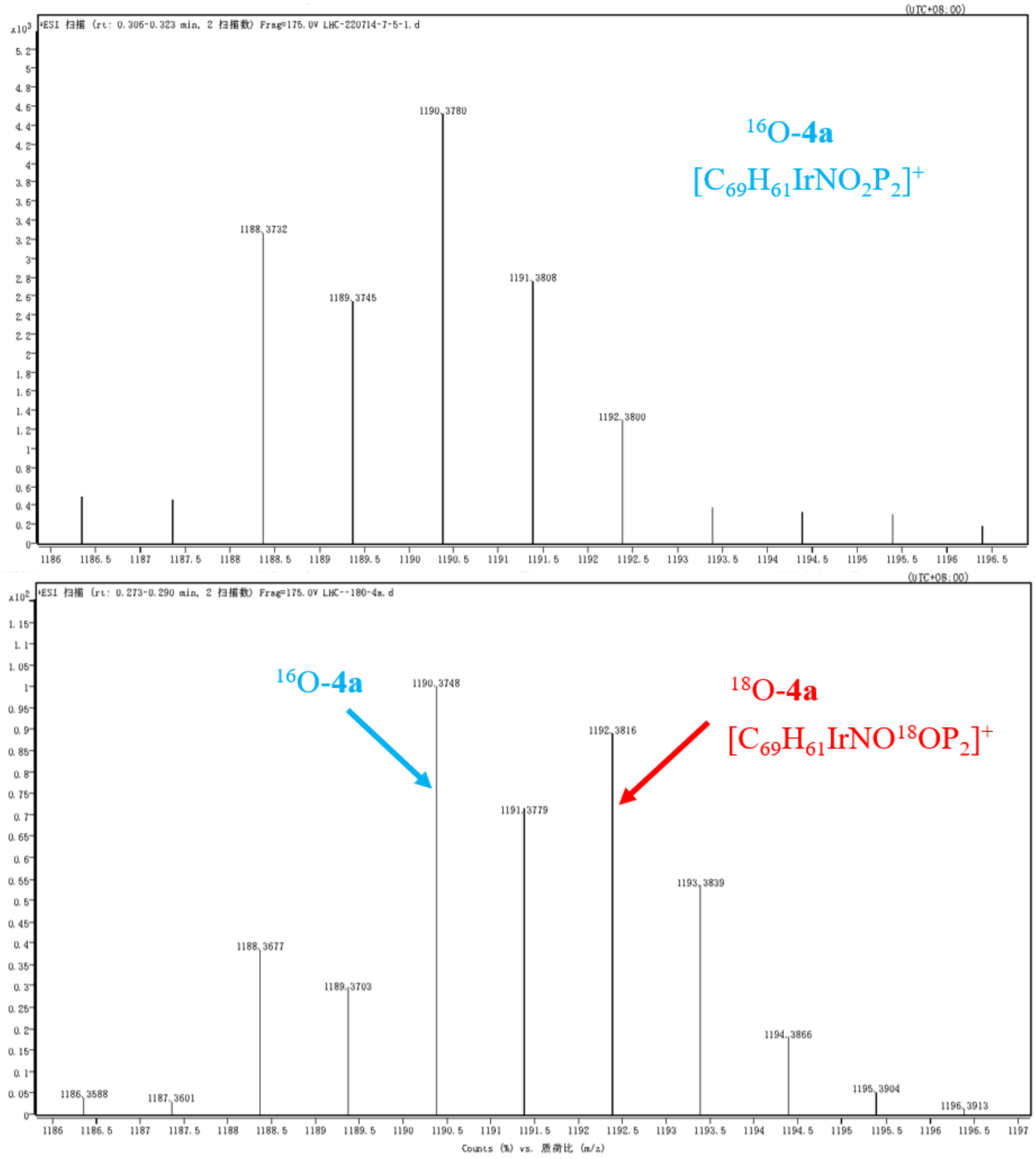

Supplementary Figure 7. Positive-ion ESI-MS spectrum for complex [^{18}O-**4a**]$^+$ measured in methanol.

5.3 The conversion between 2a and 4a.

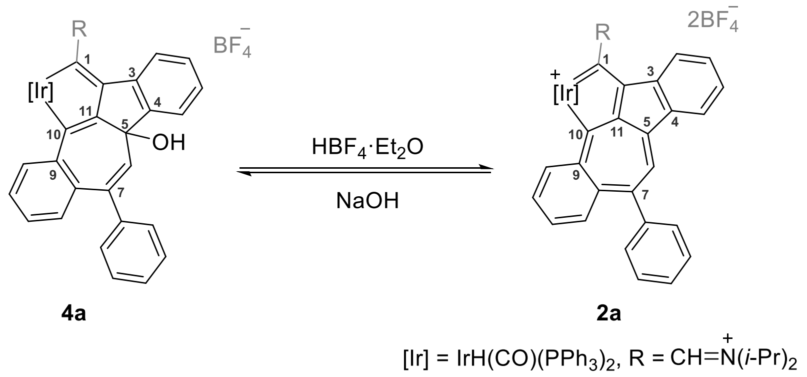

Supplementary Figure 8. The reversible conversion between compounds **2a** and **4a** by controlling the acid and base**.**

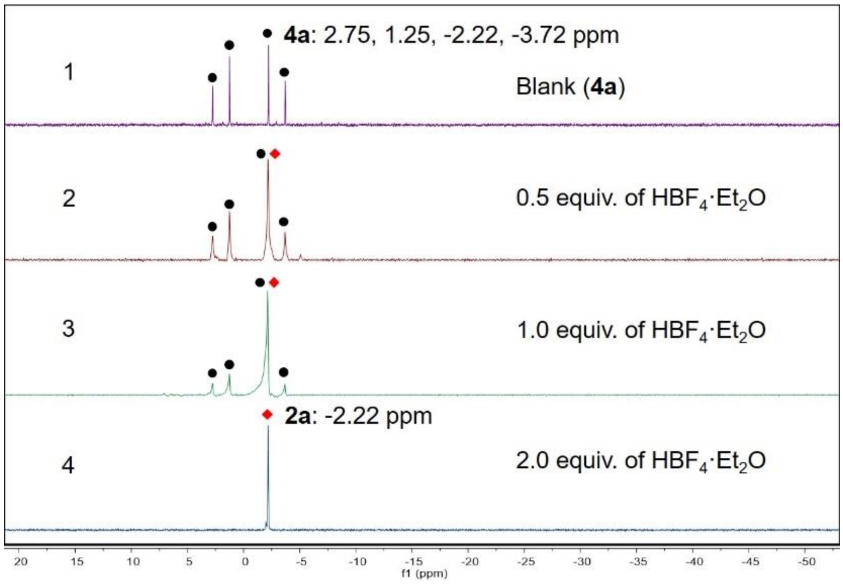

Supplementary Figure 9. The *in situ* $^{31}P\{^1H\}$ NMR spectra of addition varied amounts of $HBF_4 \cdot Et_2O$ (from 0.5 equiv. to 2.0 equiv.) in the CH_2Cl_2 solution of compound **4a**.

To explore the reversibility of this reaction, we performed the reaction of **4a** with acid. The *in situ* $^{31}P\{^1H\}$ NMR spectra revealed that by increasing the amount of $HBF_4 \cdot Et_2O$ (from 0.5 equiv. to 2.0 equiv.) in the CH_2Cl_2 solution of compound **4a**, the quantity of **4a** gradually decreased while that of **2a** increased. When the acid was added to 2.0 equiv., a complete transformation of **4a** into **2a** was observed. The result indicates that the reversible conversion between compounds **2a** and **4a** can be achieved by regulating the acidity and basicity of the system.

5.4 Preparation and characterization of complex 5a

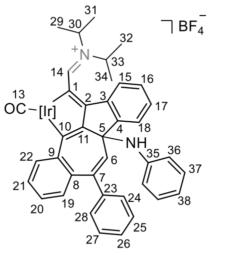

[Ir] = $IrH(PPh_3)_2$

5a

A mixture of **2a** (269.4 mg, 0.20 mmol) and aniline (21.9 μL, 0.24 mmol) in CH_2Cl_2 (2.0 mL) was stirred at room temperature under nitrogen for 6 h to give a pink solution. Then the solution was filtered by Celite, and the filtrate was concentrated under vacuum. The resulting residue was purified by reverse precipitation was performed with hexane to afford complex **5a** in pink solid. Yield: 95%. ^{1}H NMR (500.2 MHz, CD_2Cl_2): δ = 8.08 (s, 1H, *C14*H), 6.10 (d, *J* = 8.0 Hz, 2H, *C36*H), 5.53 (s, 1H, C6H), 5.42-5.37 (m, 1H, *C30*H), 3.69-3.65 (m, 1H, *C33*H), 3.52 (s, *C5N*H), 1.28 (d, *J* = 6.5 Hz, 3H, *C29*H), 0.77 (d, *J* = 7.0 Hz, 3H, *C31*H), 0.69 (d, *J* = 7.0 Hz, 3H, *C32*H), 0.61 (d, *J* = 6.5 Hz, 3H, *C34*H), -10.47 (dd, J_1 = 12.5 Hz, J_2 = 18.0 Hz, 1H, *Ir*H); ^{31}P{^{1}H} NMR (202.5 MHz, CD_2Cl_2): δ = -3.87 (dd, J_1 = 304.8 Hz, J_2 = 1732.6 Hz, *Ir*PPh_3); ^{13}C{^{1}H} NMR (125.8 MHz, CD_2Cl_2, plus ^{13}C-dept 135, ^{1}H -^{13}C HSQC and ^{1}H-^{13}C HMBC): δ = 185.6 (s, C1), 175.2 (t, *J* = 7.0 Hz, C2), 173.1 (t, *J* = 10.1 Hz, C13), 169.1 (s, C14), 166.4 (s, C11), 157.6 (s, C4), 147.9 (s, C9), 146.6 (s, C8), 143.7 (s, C19), 140.6 (s, C3), 136.3 (s, C6), 118.7 (s, C35), 116.5 (s, C36), 62.1 (s, C5), 52.5 (s, C30), 51.9 (s, C33), 23.9 (s, C29), 23.8 (s, C31), 20.4 (s, C32), 19.3 (s, C34); HRMS (ESI): (*m/z*) Calcd for $[C_{75}H_{66}IrN_2OP_2]^+$ requires 1265.4281, Found 1265.4333.

5.5 Preparation and characterization of 6a

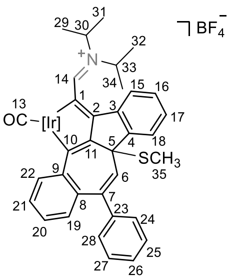

[Ir] = $IrH(PPh_3)_2$

6a

A mixture of **2a** (269.4 mg, 0.20 mmol) and sodium thiomethoxide (16.8 mg, 0.24 mmol) in CH_2Cl_2 (2.0 mL) was stirred at room temperature under nitrogen for 2 h to give a pink solution. Then the solution was filtered by Celite, and the filtrate was concentrated under vacuum. The resulting residue was purified by column chromatography (alumina (200–300 mesh), eluent: dichloromethane/acetone = 2:1) to afford complex **6a** in orange red solid. Yield: 90%. ^{1}H NMR (500.2 MHz, CD_2Cl_2): 5.56 (s, 1H, *C6*H), 5.37-5.36 (m, 1H, *C30*H), 3.78-3.73 (m, 1H, *C33*H), 2.06 (s, 3H, *C35*H), 1.31 (d, J = 6.5 Hz, 3H, *C29*H), 0.98 (d, J = 6.5 Hz, 3H, *C31*H), 0.82 (d, J = 6.5 Hz, 3H, *C32*H), 0.67 (d, J = 6.5 Hz, 3H, *C34*H), -10.49 (dd, J_1 = 12.0 Hz, J_2 = 17.0 Hz, 1H, *Ir*H); ^{31}P{^{1}H} NMR (202.5 MHz, CD_2Cl_2): δ = -2.30 (dd, J_1 = 301.5 Hz, J_2 = 2009.6 Hz, *Ir*PPh_3); ^{13}C{^{1}H} NMR (125.8 MHz, CD_2Cl_2, plus ^{13}C-dept 135, ^{1}H -^{13}C HSQC and ^{1}H-^{13}C HMBC): δ = 211.2 (s, C10), 185.6 (s, C1), 177.5 (s, C2), 173.2 (t, J = 10.1 Hz, C13), 169.6 (s, C14), 165.4 (s, C11), 158.5 (s, C4), 148.4 (s, C9), 144.5 (s, C8), 143.3 (s, C19), 136.7 (s, C6), 136.4 (s, C7), 69.7 (s, C5), 53.3 (s, C30), 52.9 (s, C35), 51.9 (s, C33), 24.3 (s, C29), 23.4 (s, C31), 20.2 (s, C32), 19.7 (s, C34); HRMS (ESI): (*m/z*) Calcd for $[C_{70}H_{63}IrNOP_2S]^+$ requires 1220.3734, Found 1220.4097.

5.6 Preparation and characterization of complex 6b

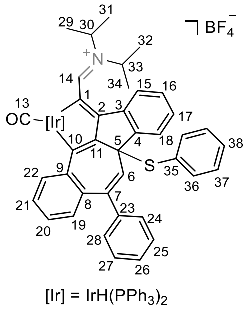

6b

A mixture of **2a** (269.4 mg, 0.20 mmol) and sodium thiophenoxide (31.7 mg, 0.24 mmol) in CH_2Cl_2 (2.0 mL) was stirred at room temperature under nitrogen for 2 h to give a pink solution. Then the solution was filtered by Celite, and the filtrate was concentrated under vacuum. The resulting residue was purified by column chromatography (alumina (200–300 mesh), eluent: dichloromethane/acetone = 2:1) to afford complex **6b** in orange red solid. Yield: 81%. ^{1}H NMR (500.2 MHz, $CDCl_3$): δ = 7.64 (s, 1H, *C14*H), 6.34 (d, J = 8.0 Hz, 1H, *C18*H), 5.20-5.15 (m, 1H,

*C30*H), 5.17 (s, 1H, C6H), 3.99-3.95 (m, 1H, *C33*H), 1.32 (d, J = 6.5 Hz, 3H, *C29*H), 1.03 (d, J = 6.5 Hz, 3H, *C31*H), 0.81 (d, J = 6.5 Hz, 3H, *C32*H), 0.69 (d, J = 6.5 Hz, 3H, *C34*H), -10.51 (dd, J_1 = 12.0 Hz, J_2 = 17.0 Hz, 1H, *Ir*H); $^{31}P\{^1H\}$ NMR (202.5 MHz, $CDCl_3$): δ = -2.07 (dd, J_1 = 301.5 Hz, J_2 = 2003.5 Hz, *Ir*PPh_3); $^{13}C\{^1H\}$ NMR (125.8 MHz, $CDCl_3$, plus ^{13}C-dept 135, ^1H -^{13}C HSQC and ^1H-^{13}C HMBC): δ = 183.2 (s, C1), 175.4 (t, J = 14.2 Hz, C2), 172.7 (t, J = 9.6 Hz, C13), 170.0 (s, C14), 164.7 (s, C11), 156.9 (s, C4), 148.3 (s, C9), 144.3 (s, C8), 142.3 (s, C3), 137.8 (s, C19), 134.2 (s, C6), 127.2 (s, C16), 125.6 (s, C18), 124.8 (s, C15), 57.1 (s, C5), 53.3 (s, C30), 51.9 (s, C33), 24.3 (s, C29), 22.9 (s, C31), 20.2 (s, C32), 18.9 (s, C34); HRMS (ESI): (*m/z*) Calcd for $[C_{75}H_{65}IrNOP_2S]^+$ requires 1282.3892, Found 1282.4068.

6. Separation and characterization of complex 7a

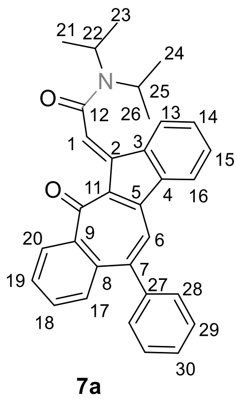

7a

A mixture of **2a** (269.4 mg, 0.20 mmol) and ^{n}Bu$_4$NF (1.0 mmol, 1M in THF) in CH_2Cl_2 (2.0 mL) was stirred at room temperature under air for 3 h to give a flavescens solution. The resulting residue was purified by column chromatography (alumina (200-300 mesh), eluent: dichloromethane/acetone = 10:1) to afford complex **7a** in pale yellow solid. Yield: 62%. ^1H NMR (500.2 MHz, $CDCl_3$): δ = 8.33 (dd, J_1 = 8.0 Hz, J_2 = 1.5 Hz, 1H, *C13*H), 8.05 (s, 1H, *C1*H), 7.94 (d, J = 7.5Hz, 1H, *C20*H), 7.72 (d, J = 7.5 Hz, 1H, C6H), 7.64 (t, J = 7.0 Hz, 1H, C19H), 4.34-4.29 (m, 1H, *C22*H), 3.68-3.63 (m, 1H, C*25*H), 1.63 (d, J = 7.0 Hz, 6H, *C21*H, C*23*H), 1.21 (d, J = 7.0 Hz, 6H, *C24*H, C*26*H); $^{13}C\{^1H\}$ NMR (125.8 MHz, $CDCl_3$, plus ^{13}C-dept 135, ^1H -^{13}C HSQC and ^1H-^{13}C HMBC): δ =188.4 (s, C10), 167.1 (s, C12), 149.7 (s, C4), 143.9 (s, C8), 140.5 (s, C9), 131.8, 130.7, 130.3, 130.2, 129.5, 129.0, 128.7, 128.6, 128.2, 124.3, 122.4, 120.9, 45.9

(s, C22, C25), 21.2 (C21, C23), 20.5 (C24, C26); HRMS (ESI): (*m/z*) Calcd for $[C_{32}H_{29}NO_2+Na^+]^+$ requires 482.2091, Found 482.2081.

6.1 The ^{18}O labeling experiment

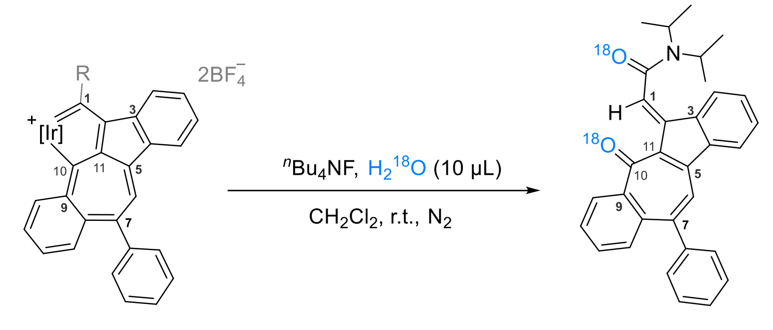

2a **^{18}O-7a**

[Ir] = IrH(CO)(PPh$_3$)$_2$, R = CH=N$^+$(*i*-Pr)$_2$

Supplementary Figure 10. The reaction of compound **2a** with ^{n}Bu$_4$NF in the presence of $H_2{}^{18}O$.

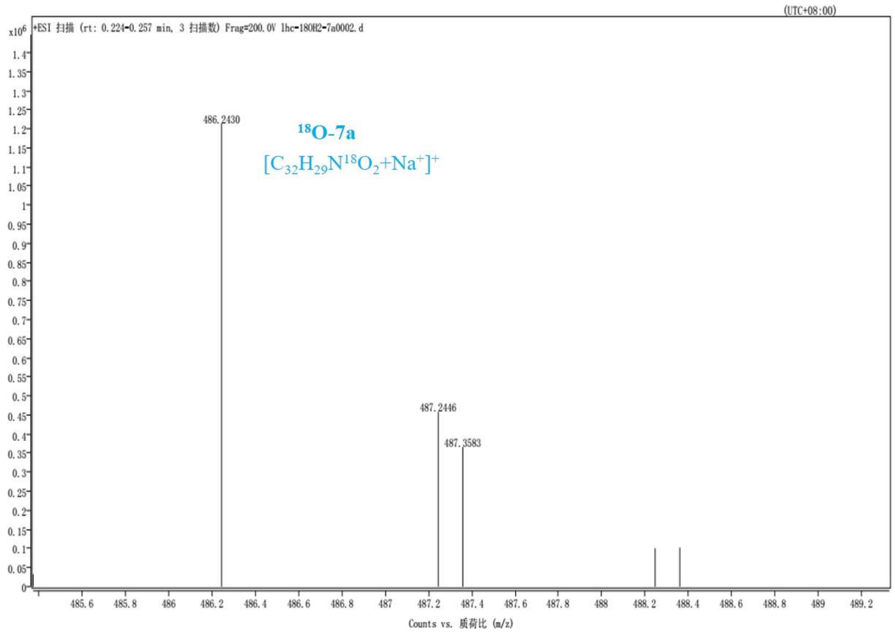

Supplementary Figure 11. Positive-ion ESI-MS spectrum for complex [^{18}O-**7a**]$^+$ measured in methanol.

To investigate the source of the carbonyl oxygen in tropone within compound **7a**, a control isotope label experiment was performed. The reaction involved treating compound **2a** with anhydrous tetrahydrofuran solution of ^{n}Bu$_4$NF in the presence of $H_2{}^{18}O$ (Supplementary Figure 10). The ESI-MS spectrum of the resulting product showed the molecular ions at m/z = 486.2430 for **^{18}O-7a** (calculated value $[C_{32}H_{29}N^{18}O_2+Na^+]^+$ at m/z = 486.2404) (Supplementary Figure 11).

The result confirms that the oxygen atom of carbonyl group (C=O) attached to C10 in compound **7a** originates from the H_2O present in the system.

It is noted that fluoride ion is considered less nucleophilic in comparison to -O, -N and -S nucleophilic reagents. Furthermore, the introduction of metal in compound **2a** results in a more dispersed positive charge distribution on the tropylium unit in resonance structure **2a'** compared with conventional organic tropylium ions, thereby reducing its binding ability with nucleophilic reagents. Based on these observations, it is hypothesized that fluoride ion does not proceed through direct nucleophilic addition in this case. Instead, the natural resonance theory analysis suggests that resonance structure **2a** (Supplementary Figure 16), which exhibits significant positive charge distribution on the metal center, may facilitate interaction with fluoride ion at the metal center, subsequently inducing the demetalation process. Notably, transition-metal fluorides are known to be intermediates with high reactivity[16-19]. Therefore, the hypothesis proposed is that fluoride ions preferentially interact with the metal center, leading to demetalation, rather than undergoing direct nucleophilic reaction.

6.2 Absorbance titration for detection limitation.

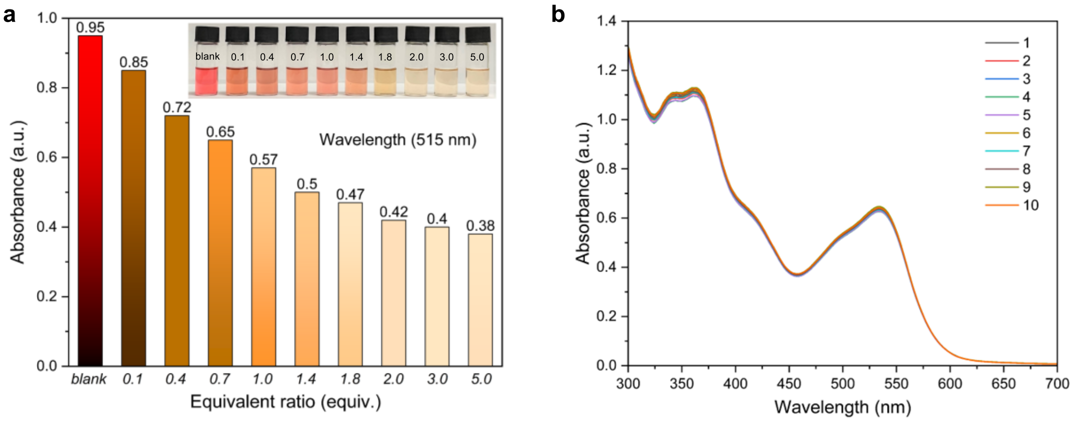

Supplementary Figure 12. a UV/Vis absorption spectra absorbance at the characteristic absorption peak (515 nm) of **2a** (0.07 mM) with varied equivalents of $^{n}Bu_4NF$ (0.1-5.0 equiv.) in CH_2Cl_2 and the corresponding photographs of solutions (insert). b Blank measurements: UV-Vis absorption spectra of the **2a** (0.07 mM) for 10 times.

The recognition property **2a** toward the F^- was further investigated by adding varied equivalents of F^- from 0.1 to 5.0 into **2a**. The color of the mixture changed from orange to pink, light yellow,

and almost colorless, depending on the amount of F^- (Supplementary Figure 12a). The limit of detection (LOD) was calculated according to LOD = $3\sigma/k$, where σ is the standard deviation of the blank measurements and k is the slope between the absorbance against the guest concentration[20]. The standard deviation (σ) of blank measurements was calculated according to measuring the absorbance of **2a** for 10 times. The collected date: 0.641, 0.639. 0.638, 0.639, 0.64, 0.641, 0.639, 0.640, 0.638, 0.641. Calculated σ: 0.00111.

6.3 The effect of water on recognizing fluoride ions

We conducted further investigations to evaluate the effect of water on the recognition of fluoride ions. Supplementary Figure 13 illustrates the results obtained by adding different amounts of water (0.01- 2.0 mL) into the reaction systems containing **2a** and $^{n}Bu_4NF$. The color of the solution changed rapidly from red to light yellow, which was like the observation in the blank reaction where no water was added. The UV-Vis absorption spectra of the resulting solutions also exhibit no significant changes compared to the blank sample. Based on these findings, it can be concluded that the fluoride ion recognition capacity of compound **2a** is unaffected by the presence of water.

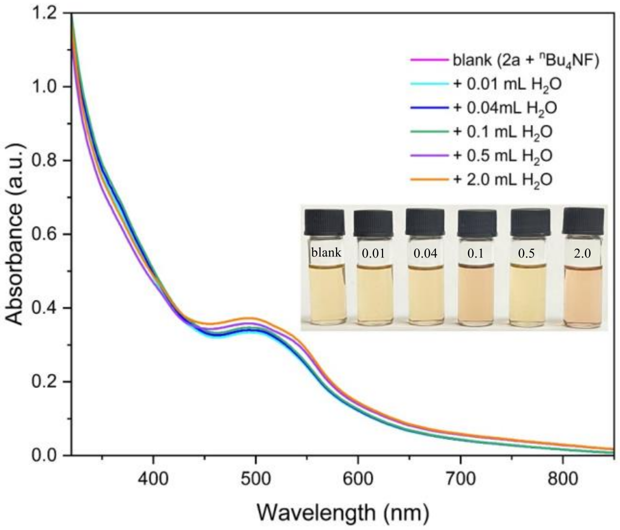

Supplementary Figure 13 UV-Vis absorption spectra of **2a** (0.07 mM) in the presence of different volumes of water after the addition of 5.0 equiv. $^{n}Bu_4NF$ in CH_3CN and color changes of solution (insert).

6.4 The fluoride recognition capability of compounds 2b-2h bearing different substituents.

We investigated the interaction between compounds **2** bearing different substituents and fluoride ions. As shown in the Supplementary Figure 14, the color of the solutions (**2a**-**2h**) rapidly changed from red to pale yellow after adding $^{n}Bu_4NF$ for 5 minutes. Despite the variations in the substituents, all the compounds demonstrated good recognition capability towards fluoride anions. The UV-Vis absorption spectra analysis revealed that the absorption around 515 nm of **2g** (R^1 = Ph, R^2 = Me) and **2h** (R^1 = Ph, R^2 = *n*-propyl) displayed a bit more decay compared with that of compound **2a** (R^1 = Ph, R^2 = H) when interacting with the fluoride ion. The studies suggest different substituents on the phenyl group had no significant effects on the sensing activity of compounds **2**.

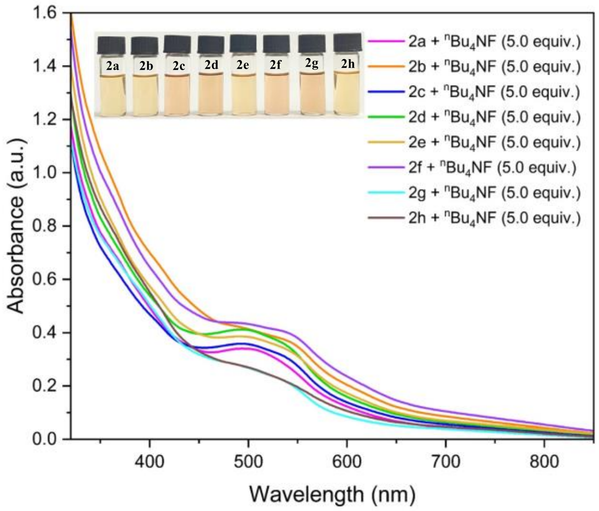

Supplementary Figure 14 UV-Vis absorption spectra of **2a-2h** (0.07 mM) after the addition of 5.0 equiv. $^{n}Bu_4NF$ in CH_2Cl_2 and the color changes of solution (insert).

7. DFT calculation of the NICS, ACID, CDD, NRT and others.

NICS(1)$_{zz}$ values

ring A = 9.4

ring B = 5.1

ring C = -10.1

ring D = -26.7

ring E = -20.4

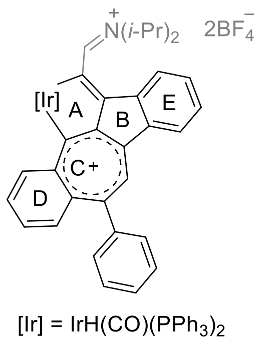

2a

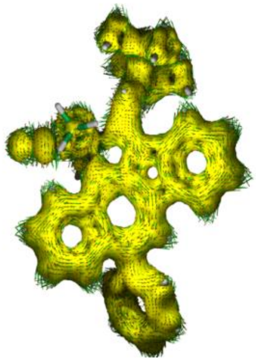

NICS(1)$_{zz}$ values for compound **2a**

ACID analysis of **2a**

Supplementary Figure 15 left. Aromaticity evaluation. NICS(1)$_{zz}$ values and NICS(1)$_{zz}$ grids for model compounds **2a** (calculated at 1.0 Å above the ring centers), the average value was used when the environments above and below the ring centers were not equivalent.; right. The ACID plot of model complex **2a** with an isosurface value of 0.025 a.u.

Calculated results of the nucleus-independent chemical shift (NICS) values based on the model compounds **2a** showed the value for C ring is -10.1 ppm (Supplementary Figure 15 left), suggesting the aromatic character of C ring. Whereas the value for A ring is 9.4 ppm, indicating A ring has certain antiaromatic character. The aromatic property of the model complex **2a** is further investigated by the anisotropy of the current-induced density (ACID) calculations, which was simplified by replacing the PPh_3 groups with PH_3 groups (Supplementary Figure 15 right). The obvious diatropic ring currents (clockwise vectors) of 7MR C suggests the aromaticity, whereas paratropic ring currents of iridacyclopentadiene A exhibits the character of antiaromatic character.

Condensed Dual Descriptor(CDD)

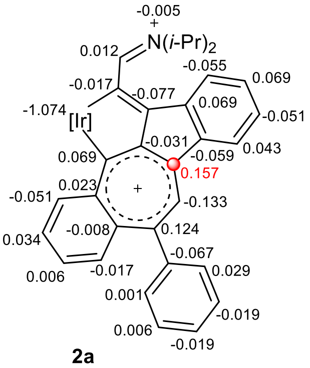

2a

C5 = 0.157

C6 = -0.133

C7 = 0.124

C8 = -0.008

C9 = 0.023

C10 = 0.069

C11 = -0.031

Reaction site

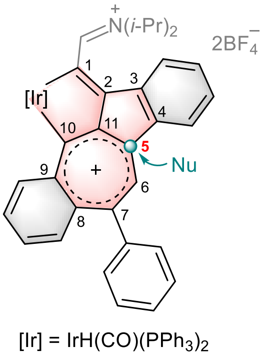

Supplementary Figure 16 Condensed Dual Descriptor (CDD) study and reaction site of **2a**.

Condensed Dual Descriptor (CDD)[7] is shown in Supplementary Figure 16. The calculated CDD values for complex **2a** showed the electron charges of C5 and C7 sites are significantly more positive than the other carbon atoms, which are supposed to be easily attack by nucleophiles.

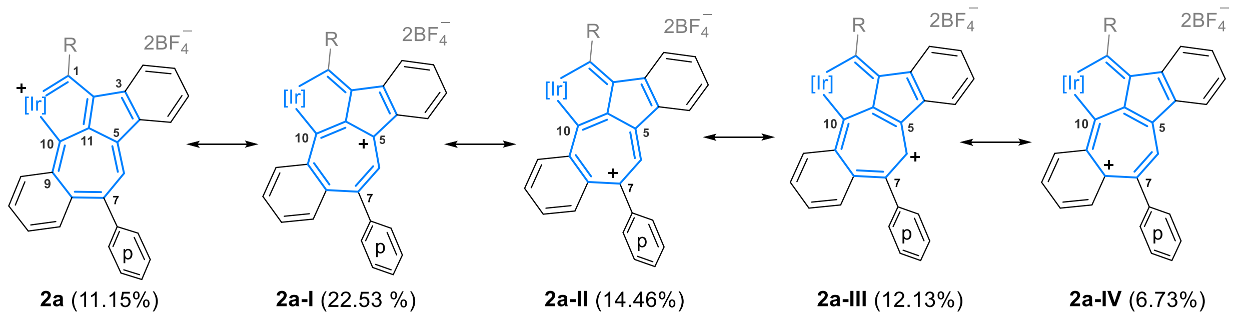

Supplementary Figure 17. The main contributions of resonance structures (**2a-I** ~ **2a-IV**) with positive charge distribution on the seven-membered ring (7MR) evaluated by natural resonance theory analysis method.

We performed further calculations to evaluate the main contributions of resonance structures (**2a-I** ~ **2a-IV**) with positive charge distribution on the seven-membered ring (7MR) using natural resonance theory analysis method (NRT) [21] (Supplementary Figure 17). The results revealed that resonance structures **2a-I** (with a positive charge at C5) and **2a-II** (with a positive charge at C7) made more significant contributions compared to other resonance structures. This is due to it could keep aromatic nature of the attached phenyl group in these two forms. The observations are consistent with the findings of the CDD studies. In addition, the resonance structure **2a-I** had the highest contribution, accounting for a maximum proportion of 22.53%. This is because the positive charge on C5 is stabilized through p-π conjugation with one benzene ring and two vinyl groups. Similarly, in resonance structure **2a-II**, the positive charge on C7 can be stabilized through p-π conjugation with a benzene ring and a vinyl group. However, it should be noted that the single crystal structure of **2a** demonstrated the presence of a large torsion angle between the planes of the benzene ring (p) and the 7MR, which hinders effective p-π conjugation. As a result, the contribution of resonance structure **2a-II** is 14.46%. Based on these analyses, we conclude that the C5 position of compound **2a** exhibits greatest electron deficiency.

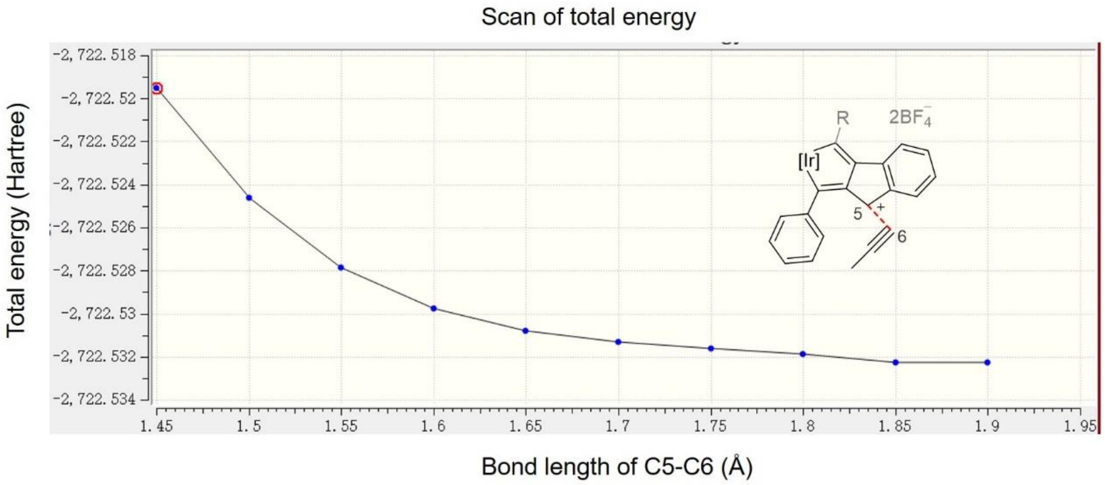

Supplementary Figure 18 Energy diagram of the interaction between propyne and **Int1**.

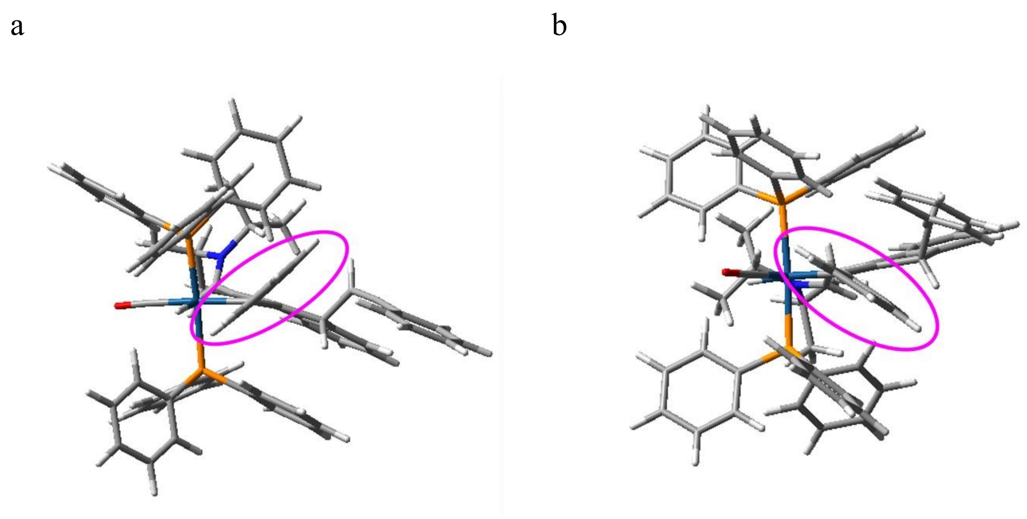

Supplementary Figure 19 Illustration of conformational changes. a, b: two states with different conformations of the phenyl group during the process from **Int2** to **Int3**.

It is reasonable to propose that the reaction proceeds electrophilic addition via **Int2** based on the several considerations and have ruled out directly calculating the [5+2] cyclization pathway. Firstly, the presence of a phenyl group attached to **Int2** is essential for the stabilization of the vinyl cation. Our calculation showed that propyne with a methyl group was used to interact with **Int 1**, the energy of the system gradually increased as the distance between the C5 and C6 atoms shortened, indicating that the formation of chemical bond between these atoms is not possible. (Supplementary Figure 18). This align well with experimental observations where trace products were obtained when alkynes were substituted with alkyl groups (e.g., 1-heptyne, ethoxyethyne

and 3-butyn-2-one). The stronger stabilizing effect of the phenyl group on the vinyl cation (**Int2**), attributed to π-π conjugation, compared to an alkyl group accounts for this discrepancy. Secondly, our calculations indicate that **Int2** is thermodynamically unstable, which can generate **Int3** with no barrier after only passing the simple conformational change as shown in Supplementary Figure 19 (a → b). Consequently, **Int2** cannot be experimentally captured. Thirdly, previous literatures (e.g. references 43-45) have shown that vinyl cations are commonly observed intermediates in carbocation-alkyne reactions (Supplementary Figure 19).

We also attempted to calculate the mechanism using a direct [5+2] cyclization method from **Int1** to **Int3**. However, no matter the initial guess for the transition state or if the virtual frequency of the vibration resembled the desired [5+2] cyclization, we consistently obtained **TS2**. This finding supports the idea that **Int2**, despite being energetically higher in energy than **Int1**, which can generate **Int3** with no barrier after only passing the simple conformational change as shown in Supplementary Figure 19 (a → b). This conformational change prevents the re-decomposition of **Int2** back into the raw materials (**Int1**) and makes it challenging to capture **Int2** experimentally. Thus, we have ruled out directly calculating the [5+2] cyclization pathway and further support the proposed reaction pathway involving the formation of **Int2** followed by the simple conformational change to yield **Int3**.

The proposed mechanism involving electrophilic addition through **Int2**, supported by calculations, experimental observations and literature references, provides a reasonable explanation for the reaction pathway.

8. UV/Vis absorption spectra of complexes 4a-4j

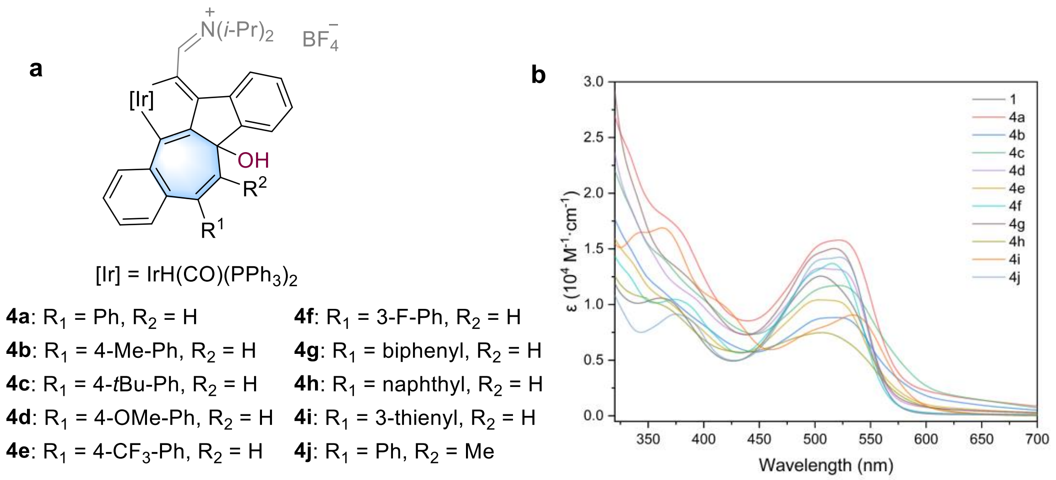

Supplementary Figure 20 a The structure of complex **4**. b UV-Vis absorption spectra of complexes **4a-4j** were measured in CH_2Cl_2 at r.t. (1.0×10^{-5} M).

The UV/Vis absorption spectra of complexes **4** exhibits broad and strong absorption, as shown in Supplementary Figure 20b. The characteristic energy absorption band is not affected by substituents, the molar absorption coefficient value (ε) of characteristic energy absorption varied from 0.8 to 1.6×10^4 M^{-1} cm^{-1}.

9. The cyclic voltammetry (CV) experiments of complexes 2a-2c, 2h, 4c, 4f, and 6b

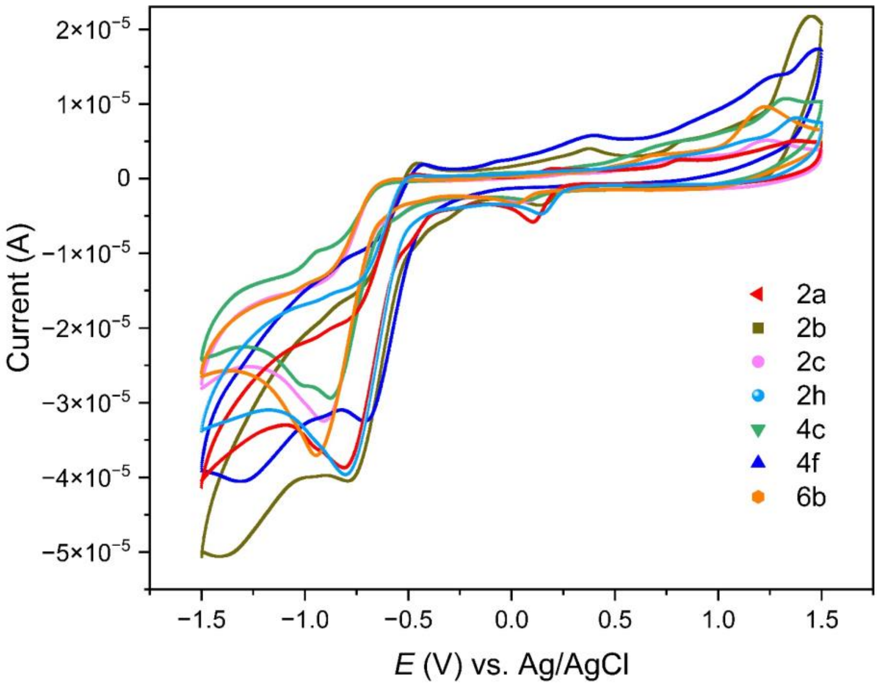

Supplementary Figure 21 Cyclic voltammograms of **2a-2c**, **2h**, **4c**, **4f**, and **6b** in CH_2Cl_2 obtained by the cyclic voltammetry (CV) with a glassy carbon as the working electrode, a platinum rod as the auxiliary electrode, Ag/AgCl as the reference electrode, [^{n}Bu_4N]BF_4 as the supporting electrolyte and a ferrocene/ferrocenium couple as the external standard.

We investigated the electrochemical properties of the pentacyclic compounds. The obtained results demonstrate the presence of redox processes in all complexes (Supplementary Figure 21), however, these processes are found to be irreversible. The influence of phenyl substituents on the reduction potentials is evident. In the case of compound **2b**, where electron-donating -OCH_3 group was introduced, an anodic shift in the reduction potential was observed compared to **2a**. Conversely, the incorporation of an electron-withdrawing -CF_3 group in compound **2c** led to a cathodic shift in reduction potential. Other derivatives such as **4c**, **4f**, and **6b** also exhibit slightly shifts in their reduction potentials compared to that of **2a**.

10. Thermal stability tests

Supplementary Table 1. Thermal stability tests for complexes **2a-2h**, **3a, 4a**, **5a**, **6a** and **6b** in the solid state under air condition.[a]

Temperature (°C) / Complexes No.	100 (4 h)	110 (4 h)	120 (4 h)	130 (4 h)	140 (4 h)	160 (4 h)
**2a**	●	●	●	●	▲	■
**2b**	●	●	●	●	▲	■
**2c**	●	●	●	●	▲	■
**2d**	●	●	●	●	▲	■
**2e**	●	●	●	●	▲	■
**2f**	●	●	●	●	▲	■
**2g**	●	●	●	●	▲	■
**2h**	●	●	●	●	▲	■
**3a**	●	▲	■	-	-	-
**4a**	●	▲	▲	■	-	-
**5a**	●	▲	▲	■	-	-
**6a**	●	▲	▲	▲	■	-
**6b**	●	▲	▲	▲	■	-

[a] ● = stable; ▲ = Partly decomposed; ■ = Completely decomposed.

11. X-ray crystallographic analysis

Single-crystal X-ray diffraction data were collected on a Rigaku XtaLAB Synergy, Dualflex, Rigaku XtaLAB Synergy-S diffractometer coupled to a RigakuHypix detector with Cu Kα radiation (λ = 1.54184 Å) or Mo Kα radiation (λ = 0.71073 Å). Single crystals suitable for X-ray diffraction were obtained by recrystallization from a solution of CH_2Cl_2 layered with *n*-hexane. The crystal was kept at a steady $T = 100$ K during the data collection. The structures were solved with the SHELXT solution program by using **Olex 2** (Dolomanov *et al.*, 2009) as the graphical interface. The model was refined using Least Squares minimisation with the 2018/3 version of the program SHELXL[1]. Non-H atoms were refined anisotropically unless otherwise stated. The hydrogen atoms were introduced at their geometric positions and refined as riding atoms unless otherwise stated. The disordered solvents were removed from the dataset using the Solvent Mask routine of Olex 2 which was reported in the CIF. CCDC 2254984 (**L**), 2254983 (**1**), 2254985 (**2a**), 2254986 (**2b**), 2254987 (**2c**), 2254988 (**2d**), 2254991 (**3a**), 2254992 (**3x**), 2254993 (**4a**), 2254994 (**4d**), 2254995 (**4f**), 2262455 (**4j**), 2254996 (**6a**), 2254997 (**6b**), 2254998 (**7a**) contain the supplementary crystallographic data for this paper. Further details on the crystal data, data collection, and refinements are provided in Supplementary Supplementary Table 2-23. These data can be obtained free of charge from the Cambridge Crystallographic Data Centre via https://www.ccdc.cam.ac.uk/structures/?access=referee. Among, the checkcif of **4a**, **4d** and **6a** emerged B-level alerts with large residual density, which is probably due to the heavy atom Ir01.

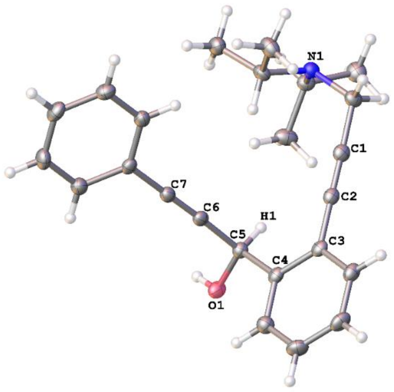

CCDC: 2254984

Supplementary Figure 22. X-ray molecular structure of **L**. (ellipsoids set at 50% probability). The phenyl groups in the PPh_3 moieties are omitted for clarity.

Supplementary Table 2. Selected bond distances and angles for complex **L**.

Bond Distances(Å)			
C1–C2	1.2007(19)	C2-C3	1.4428(18)
C3-C4	1.4059(3)	C4-C5	1.5233(17)
C5-C6	1.4786(17)	C6-C7	1.2001(18)
**Bond Angles(°)**			
C1-C2-C3	178.59(14)	C4-C5-C6	109.88(10)
C2-C3-C4	121.33(11)	C5-C6-C7	177.38(14)
C3-C4-C5	120.31(11)	C4-C5-O1	108.65(10)

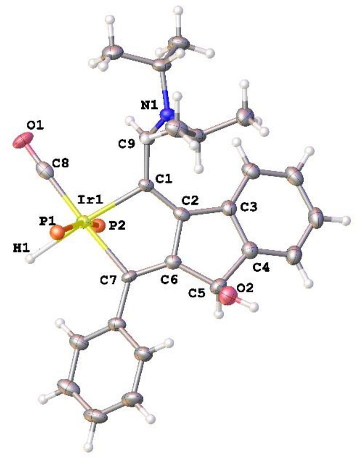

CCDC: 2254983

Supplementary Figure 23. X-ray molecular structure of **1**. (ellipsoids set at 50% probability). The phenyl groups in the PPh_3 moieties are omitted for clarity.

Supplementary Table 3. Selected bond distances and angles for complex **1**.

Bond Distances(Å)			
Ir1-C1	2.183(4)	C4-C5	1.519(6)
Ir1-C7	2.139(4)	C5-C6	1.530(6)
Ir1-C8	1.932(5)	C6-C7	1.362(6)
C8-O1	1.125(6)	C2-C6	1.447(5)
C1-C2	1.362(6)	C5-O2	1.436(5)
C2-C3	1.468(6)	C1-C9	1.418(5)
C3-C4	1.398(6)	C9-N1	1.311(5)
Bond Angles(°)			
C7-Ir-1C1	79.37(15)	C6-C7-Ir1	111.3(3)
C8-Ir1-C1	97.99(16)	O1-C8-Ir1	178.1(4)
C8-Ir1-C7	176.67(15)	O2-C5-C6	108.2(3)
C9-C1-Ir1	121.9(3)	O2-C5-C4	110.5(3)
C2-C1-Ir1	109.1(3)	C4-C5-C6	101.1(3)
C2-C1-C9	128.8(4)	C1-C2-C6	118.4(4)
N1-C9-C1	129.2(4)	C1-C2-C3	133.3(4)
C7-C6-C5	133.3(4)	C6-C2-C3	107.8(3)
C7-C6-C2	118.7(4)	C3-C4-C5	111.3(4)
C2-C6-C5	108.1(3)	C4-C3-C2	108.1(4)

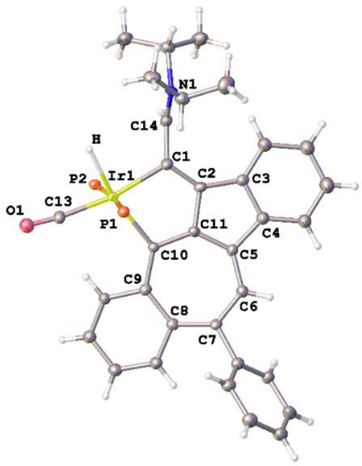

CCDC: 2254985

Supplementary Figure 24. X-ray molecular structure of **2a**. (ellipsoids set at 50% probability). The phenyl groups in the PPh_3 moieties are omitted for clarity.

Supplementary Table 4. Selected bond distances and angles for complex **2a**.

Bond Distances(Å)					
Ir1-C1	2.115(11)	C10-C11	1.438(14)	C7-C6	1.392(14)
Ir1-C10	2.182(9)	C11-C5	1.400(14)	C8-C9	1.453(14)
Ir1-C13	1.908(11)	C10-C9	1.434(14)	C5-C6	1.394(15)
C2-C1	1.351(14)	C4-C3	1.416(15)	O1-C13	1.120(13)
C3-C2	1.440(15)	C4-C5	1.463(15)	C1-C14	1.435(15)
C11-C2	1.478(13)	C7-C8	1.442(14)	N1-C14	1.303(13)

Bond Angles(°)			
C13-Ir1-C10	109.6(4)	C3-C4-C5	108.5(9)
C1-Ir1-C10	80.2(4)	C6-C7-C8	128.2(10)
C11-C10-Ir1	108.9(7)	C4-C3-C2	108.1(9)
C11-C10-C9	121.1(9)	C7-C8-C9	128.2(10)
C10-C11-C2	118.6(9)	C11-C5-C4	108.2(9)
C5-C11-C10	133.3(10)	C11-C5-C6	128.8(10)
C5-C11-C2	108.1(9)	C6-C5-C4	122.7(9)
C3-C2-C11	106.8(9)	C7-C6-C5	127.1(10)
C1-C2-C11	117.0(10)	C2-C1-Ir1	114.8(8)
C10-C9-C8	129.5(10)	C14-C1-Ir1	120.2(7)

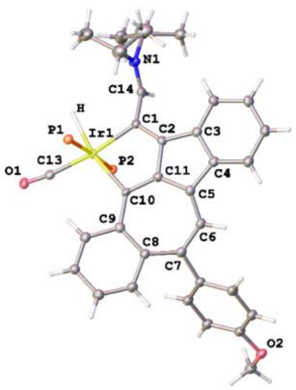

CCDC: 2254986

Supplementary Figure 25. X-ray molecular structure of **2b**. (ellipsoids set at 50% probability). The phenyl groups in the PPh_3 moieties are omitted for clarity.

Supplementary Table 5. Selected bond distances and angles for complex **2b**.

Bond Distances(Å)					
Ir1-C1	2.103(6)	C10-C11	1.410(9)	C7-C6	1.382(10)
Ir1-C10	2.197(6)	C11-C5	1.425(10)	C8-C9	1.443(10)
Ir1-C13	1.914(7)	C10-C9	1.441(9)	C5-C6	1.378(10)
C2-C1	1.355(9)	C4-C3	1.400(10)	O1-C13	1.115(8)
C3-C2	1.453(9)	C4-C5	1.474(10)	C1-C14	1.453(9)
C11-C2	1.467(9)	C7-C8	1.450(10)		

Bond Angles(°)			
C1-Ir1-C10	80.3(3)	C3-C4-C5	109.5(6)
C13-Ir1-C1	171.1(3)	N1-C14-C1	132.6(7)
C14-C1-Ir1	122.8(5)	C11-C5-C4	107.1(6)
C2-C1-Ir1	114.7(5)	C6-C5-C11	128.4(6)
C2-C1-C14	119.9(6)	C6-C5-C4	122.8(6)
C11-C10-Ir1	109.6(4)	C1-C2-C3	134.8(6)
C11-C10-C9	120.9(6)	C1-C2-C11	116.9(6)
C9-C10-Ir1	129.5(5)	C3-C2-C11	107.7(6)
C4-C3-C2	107.8(6)	C8-C9-C10	128.0(6)
C10-C11-C5	133.7(6)	C9-C8-C7	128.7(6)
C10-C11-C2	118.2(6)	C5-C6-C7	127.3(6)
C5-C11-C2	107.7(6)	C6-C7-C8	125.5(6)

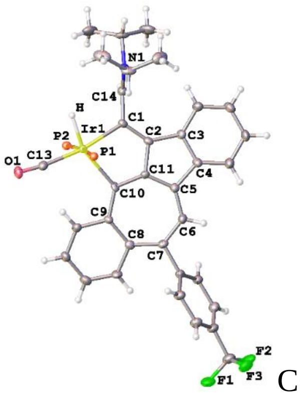

CCDC: 2254987

Supplementary Figure 26. X-ray molecular structure of **2c**. (ellipsoids set at 50% probability). The phenyl groups in the PPh_3 moieties are omitted for clarity.

Supplementary Table 6. Selected bond distances and angles for complex **2c**.

Bond Distances(Å)					
Ir1-C1	2.096(3)	C1-C13	1.442(4)	C8-C7	1.442(5)
Ir1-C10	2.198(3)	C3-C4	1.408(5)	C5-C4	1.472(4)
Ir1-C13	1.909(3)	C3-C2	1.460(4)	C5-C6	1.390(4)
C9-C10	1.455(4)	C10-C11	1.407(4)	C7-C6	1.380(4)
C9-C8	1.454(4)	C11-C5	1.413(4)	N1-C14	1.286(4)
C1-C2	1.355(5)	C11-C2	1.465(4)	O1-C13	1.134(4)

Bond Angles(°)			
C1-Ir1-C10	80.38(12)	C11-C5-C4	107.7(3)
C13-Ir1-C1	170.32(13)	C6-C5-C11	128.3(3)
C8-C9-C10	128.8(3)	C6-C5-C4	123.9(3)
C2-C1-Ir1	113.8(2)	C3-C4-C5	108.8(3)
C2-C1-C14	122.2(3)	C1-C2-C3	134.2(3)
C4-C3-C2	107.7(3)	C1-C2-C11	118.3(3)
C9-C10-Ir1	128.4(2)	C3-C2-C11	107.5(3)
C11-C10-Ir1	108.7(2)	C6-C7-C8	129.4(3)
C11-C10-C9	122.9(3)	C7-C6-C5	128.0(3)
C10-C11-C5	133.8(3)	N1-C14-C1	129.4(3)
C10-C11-C2	118.1(3)	C12-Ir1-C10	109.30(12)
C5-C11-C2	108.1(3)	C7-C8-C9	127.8(3)

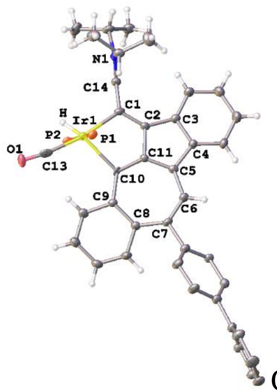

CCDC: 2254988

Supplementary Figure 27. X-ray molecular structure of **2d**. (ellipsoids set at 50% probability). The phenyl groups in the PPh_3 moieties are omitted for clarity.

Supplementary Table 7. Selected bond distances and angles for complex **2d**.

Bond Distances(Å)					
Ir1-C10	2.214(3)	C4-C5	1.468(5)	C6-C5	1.396(5)
Ir1-C1	2.075(4)	C1-C14	1.470(5)	C6-C7	1.365(5)
C3-C4	1.412(5)	C1-C2	1.341(6)	C5-C11	1.419(5)
C3-C2	1.461(5)	C8-C9	1.452(5)	N1-C14	1.278(5)
C10-C9	1.445(5)	C8-C7	1.463(6)	O1-C13	1.157(5)
C10-C11	1.391(5)	C2-C11	1.469(5)		

Bond Angles(°)			
C1-Ir1-C10	79.44(19)	C10-C9-C8	127.5(5)
C12-Ir1-C10	110.1(2)	C3-C2-C11	107.6(4)
C4-C3-C2	107.6(4)	C1-C2-C3	135.4(5)
C9-C10-Ir1	127.9(4)	C1-C2-C11	117.0(4)
C11-C10-Ir1	108.6(3)	C7-C6-C5	127.4(5)
C11-C10-C9	123.5(4)	C6-C5-C4	123.6(5)
C3-C4-C5	109.2(4)	C6-C5-C11	128.6(5)
C14-C1-Ir1	120.6(4)	C11-C5-C4	107.5(4)
C2-C1-Ir1	115.7(3)	C10-C11-C2	118.6(4)
C2-C1-C14	122.0(5)	C10-C11-C5	133.5(4)
N1-C14-C1	130.1(5)	C5-C11-C2	107.9(4)
C7-C8-C9	128.6(4)	C6-C7-C8	127.7(4)

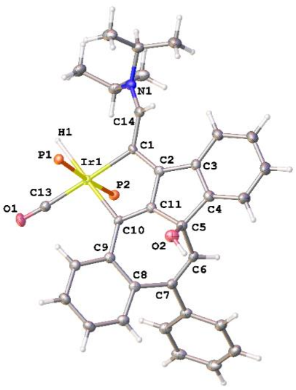

CCDC: 2254993

Supplementary Figure 28 X-ray molecular structure of **4a**. (ellipsoids set at 50% probability). The phenyl groups in the PPh_3 moieties are omitted for clarity.

Supplementary Table 8. Selected bond distances and angles for complex **4a**.

Bond Distances(Å)					
Ir1-C10	2.185(4)	C5-C4	1.515(7)	C2-C3	1.453(7)
Ir1-C1	2.140(4)	C1-C2	1.403(6)	C8-C9	1.430(6)
C10-C11	1.347(6)	C1-C14	1.432(6)	C4-C3	1.394(7)
C10-C9	1.479(6)	C7-C6	1.340(7)	O2-C5	1.441(5)
C5-C6	1.494(6)	C7-C8	1.479(6)	N1-C14	1.308(6)
C5-C11	1.513(6)	C11-C2	1.436(6)	O1-C13	1.123(6)

Bond Angles(°)			
C1-Ir1-C10	80.32(18)	C7-C6-C5	123.8(5)
C13-Ir1-C10	101.3(2)	C10-C11-C5	129.7(4)
C11-C10-Ir1	110.1(3)	C10-C11-C2	120.4(4)
C11-C10-C9	119.6(4)	C2-C11-C5	109.8(4)
C9-C10-Ir1	130.3(3)	C1-C2-C11	118.3(4)
O2-C5-C6	111.8(4)	C1-C2-C3	133.4(5)
C6-C5-C11	108.8(4)	C11-C2-C3	108.2(4)
C6-C5-C4	112.9(4)	C9-C8-C7	124.8(4)
C11-C5-C4	101.6(4)	C3-C4-C5	111.8(4)
C2-C1-Ir1	110.5(3)	C4-C3-C2	108.3(5)
C2-C1-C13	114.5(4)	C8-C9-C10	124.4(4)
C6-C7-C8	123.3(5)	O2-C5-C4	111.2(4)

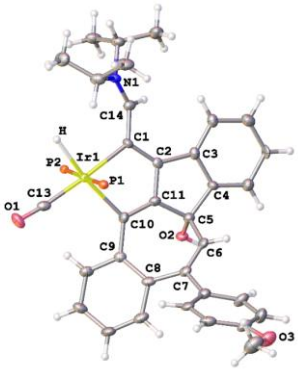

CCDC: 2254994

Supplementary Figure 29. X-ray molecular structure of **4d**. (ellipsoids set at 50% probability). The phenyl groups in the PPh_3 moieties are omitted for clarity.

Supplementary Table 9. Selected bond distances and angles for complex **4d**.

Bond Distances(Å)					
Ir1-C10	2.181(8)	C2-C3	1.484(11)	C8-C9	1.447(12)
Ir1-C13	1.883(9)	C2-C11	1.431(11)	C6-C7	1.333(12)
Ir1-C1	2.141(8)	C2-C1	1.385(12)	C6-C5	1.509(12)
O2-C5	1.444(10)	C10-C11	1.361(12)	C4-C3	1.416(12)
C1-C14	1.449(11)	C10-C9	1.478(11)	C4-C5	1.519(11)
C8-C7	1.480(12)	C11-C5	1.512(11)		

Bond Angles(°)			
C13-Ir1-C10	102.2(3)	C6-C7-C8	124.3(8)
C1-Ir1-C10	80.4(3)	C2-C11-C5	110.9(7)
C11-C2-C3	107.9(7)	C10-C11-C2	120.9(7)
C1-C2-C3	133.6(8)	C10-C11-C5	128.1(7)
C1-C2-C11	118.5(7)	C2-C1-Ir1	110.9(5)
C11-C10-Ir1	109.3(6)	C8-C9-C10	123.7(7)
C11-C10-C9	121.4(7)	O2-C5-C6	111.3(7)
C9-C10-Ir1	129.3(6)	O2-C5-C4	112.5(7)
C9-C8-C7	125.8(7)	C6-C5-C4	112.4(7)
C7-C6-C5	124.6(8)	C6-C5-C11	108.7(7)
C3-C4-C5	111.5(7)	C11-C5-C4	101.9(7)
C4-C3-C2	107.7(7)	N1-C14-C1	132.1(8)

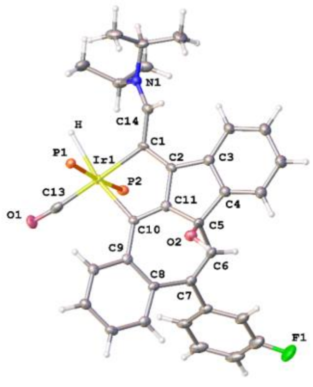

CCDC: 2254995

Supplementary Figure 30. X-ray molecular structure of **4f**. (ellipsoids set at 50% probability). The phenyl groups in the PPh_3 moieties are omitted for clarity.

Supplementary Table 10. Selected bond distances and angles for complex **4f**.

Bond Distances(Å)					
Ir1-C1	2.163(6)	C2-C11	1.453(8)	C9-C10	1.490(8)
Ir1-C10	2.190(5)	C7-C8	1.486(8)	C9-C8	1.430(8)
C6-C7	1.334(9)	C1-C14	1.413(8)	C10-C11	1.340(8)
C6-C5	1.502(8)	C3-C4	1.392(9)	Ir1-C13	1.909(7)
C2-C1	1.366(8)	C5-C4	1.526(8)	O1-C13	1.116(8)
C2-C3	1.474(8)	C5-C11	1.496(8)	N1-C14	1.317(8)

Bond Angles(°)			
C1-Ir1-C10	79.7(2)	C11-C5-C4	102.2(5)
C13-Ir1-C1	178.5(2)	C8-C9-C10	123.8(5)
C7-C6-C5	123.4(5)	C9-C10-Ir1	129.8(4)
C1-C2-C3	133.4(5)	C11-C10-Ir1	110.6(4)
C1-C2-C11	120.1(5)	C11-C10-C9	119.5(5)
C11-C2-C3	106.5(5)	C9-C8-C7	125.1(5)
C6-C7-C8	123.5(5)	N1-C14-C1	133.8(6)
C2-C1-Ir1	110.0(4)	C3-C4-C5	110.8(5)
C2-C1-C14	116.5(5)	C2-C11-C5	110.7(5)
C4-C3-C2	109.5(5)	C10-C11-C2	119.1(5)
C6-C5-C4	112.5(5)	C10-C11-C5	130.0(5)
C11-C5-C6	109.2(5)	C13-Ir1-C10	101.8(2)

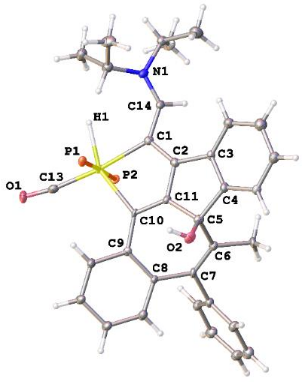

CCDC: 2262455

Supplementary Figure 31. X-ray molecular structure of **4j**. (ellipsoids set at 50% probability). The phenyl groups in the PPh_3 moieties are omitted for clarity.

Supplementary Table 10. Selected bond distances and angles for complex **4j**.

Bond Distances(Å)					
Ir1-C1	2.158(7)	C11-C2	1.442(9)	C7-C6	1.346(9)
Ir1-C10	2.186(6)	C11-C10	1.349(8)	C7-C8	1.488(9)
O2-C5	1.431(7)	C14-C1	1.405(9)	C8-C9	1.438(9)
C4-C5	1.536(9)	C1-C2	1.396(9)	Ir1-C13	1.891(8)
C4-C3	1.408(9)	C5-C6	1.523(9)	O1-C13	1.139(9)
N1-C14	1.309(8)	C2-C3	1.465(9)	Ir1-P1	2.3481(17)
C11-C5	1.517(8)	C10-C9	1.448(9)		

Bond Angles(°)			
C1-Ir1-C10	81.0(2)	C4-C5-C6	115.3(5)
C8-C9-C10	123.4(6)	C11-C5-C6	106.8(5)
C9-C8-C7	125.0(6)	C11-C2-C3	108.3(5)
C3-C4-C5	111.4(5)	C1-C2-C11	118.9(6)
C2-C11-C5	110.5(5)	C1-C2-C3	132.6(6)
C10-C11-C5	127.8(6)	C11-C10-Ir1	109.1(4)
C10-C11-C2	121.4(5)	C11-C10-C9	120.7(5)
C2-C1-Ir1	109.6(5)	C9-C10-Ir1	130.2(4)
O2-C5-C4	110.3(5)	C6-C7-C8	124.7(6)
C7-C6-C5	118.8(6)	C4-C3-C2	108.3(6)
C4-C5-C11	101.4(5)		

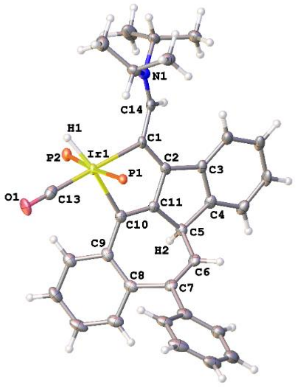

CCDC: 2254991

Supplementary Figure 32. X-ray molecular structure of **3a**. (ellipsoids set at 50% probability). The phenyl groups in the PPh_3 moieties are omitted for clarity.

Supplementary Table 11. Selected bond distances and angles for complex **3a**.

Bond Distances(Å)					
Ir1-C1	2.170(4)	C2-C11	1.442(6)	C6-C5	1.501(7)
Ir1-C10	2.160(5)	C2-C1	1.386(6)	C4-C5	1.511(6)
Ir1-C13	1.890(5)	C3-C4	1.406(7)	C1-C14	1.423(7)
C8-C7	1.487(7)	C7-C6	1.343(7)	C10-C9	1.481(6)
C8-C9	1.424(7)	C11-C5	1.509(6)	N1-C14	1.305(6)
C2-C3	1.473(6)	C11-C10	1.342(7)		

Bond Angles(°)			
C10-Ir1-C1	80.30(17)	C7-C6-C5	121.7(5)
C13-Ir1-C1	178.63(19)	C3-C4-C5	111.4(4)
C13-Ir1-C10	99.41(19)	C2-C1-Ir1	109.5(3)
C9-C8-C7	124.9(4)	C2-C1-C14	115.6(4)
C11-C2-C3	107.5(4)	C14-C1-Ir1	133.1(3)
C1-C2-C3	133.5(4)	C11-C5-C4	102.2(4)
C1-C2-C11	119.0(4)	C6-C5-C11	108.3(4)
C4-C3-C2	108.3(4)	C6-C5-C4	115.5(4)
C6-C7-C8	123.1(5)	C11-C10-Ir1	110.8(3)
C2-C11-C5	110.4(4)	C11-C10-C9	119.1(4)
C10-C11-C2	120.3(4)	C9-C10-Ir1	129.9(3)
C10-C11-C5	129.1(4)	C8-C9-C10	123.7(4)

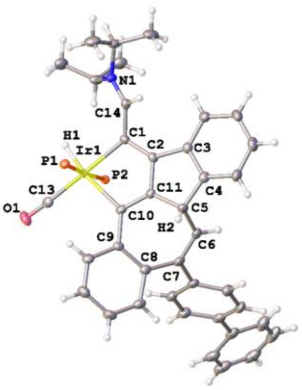

CCDC: 2254992

Supplementary Figure 33. X-ray molecular structure of **3x**. (ellipsoids set at 50% probability). The phenyl groups in the PPh_3 moieties are omitted for clarity.

Supplementary Table 12. Selected bond distances and angles for complex **3x**.

Bond Distances(Å)					
Ir1-C1	2.148(3)	C2-C3	1.472(4)	C6-C7	1.346(4)
Ir1-C13	1.895(3)	C5-C11	1.511(4)	C7-C8	1.483(4)
Ir1-C10	2.165(3)	C5-C4	1.510(4)	C8-C9	1.431(4)
C1-C2	1.389(4)	C5-C6	1.506(4)	C9-C10	1.478(4)
C1-C14	1.429(4)	C11-C10	1.358(4)	N1-C14	1.306(4)
C2-C11	1.435(4)	C4-C3	1.409(4)	O1-C13	1.137(4)

Bond Angles(°)			
C1-Ir1-C10	80.25(11)	C10-C11-C2	120.3(3)
C13-Ir1-C1	178.53(12)	C10-C11-C5	128.5(3)
C13-Ir1-C10	101.09(12)	C3-C4-C5	111.5(3)
C2-C1-Ir1	110.7(2)	C7-C6-C5	121.8(3)
C2-C1-C14	115.1(3)	C4-C3-C2	108.3(3)
C1-C2-C11	118.3(3)	C6-C7-C8	122.9(3)
C1-C2-C3	134.4(3)	C9-C8-C7	125.0(3)
C11-C2-C3	107.3(2)	C8-C9-C10	124.2(3)
C4-C5-C11	101.8(2)	C11-C10-Ir1	110.2(2)
C6-C5-C11	108.4(2)	C11-C10-C9	118.3(3)
C6-C5-C4	114.7(3)	C9-C10-Ir1	131.4(2)
C2-C11-C5	110.9(2)	N1-C14-C1	132.9(3)

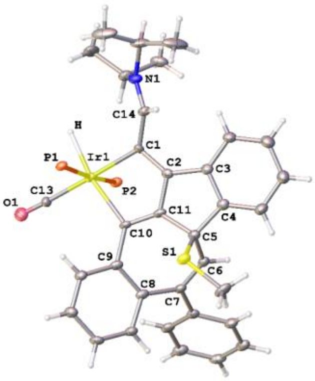

CCDC: 2254996

Supplementary Figure 34. X-ray molecular structure of **6a**. (ellipsoids set at 50% probability). The phenyl groups in the PPh_3 moieties are omitted for clarity.

Supplementary Table 13. Selected bond distances and angles for complex **6a**.

Bond Distances(Å)					
Ir1-C1	2.137(5)	C7-C6	1.343(9)	C8-C9	1.429(8)
Ir1-C10	2.199(5)	C7-C8	1.494(8)	C5-C4	1.502(8)
C1-C14	1.445(8)	C6-C5	1.497(8)	C5-C11	1.523(9)
C1-C2	1.386(9)	C2-C3	1.468(9)	Ir1-C13	1.913(6)
C10-C9	1.478(7)	C2-C11	1.441(8)	S1-C5	1.850(6)
C10-C11	1.330(9)	C3-C4	1.409(9)		

Bond Angles(°)			
C13-Ir1-C1	178.6(2)	C1-C2-C11	117.7(4)
C1-Ir1-C10	79.46(19)	C11-C2-C3	108.3(5)
C2-C1-Ir1	111.4(3)	C4-C3-C2	108.9(4)
C10-C11-C2	120.7(5)	C9-C8-C7	124.3(5)
C2-C11-C5	108.8(4)	C6-C5-S1	112.4(4)
C10-C11-C5	130.3(5)	C6-C5-C4	114.4(5)
C9-C10-Ir1	130.2(4)	C6-C5-C11	108.4(5)
C11-C10-Ir1	109.9(3)	C4-C5-S1	110.5(4)
C11-C10-C9	119.9(5)	C4-C5-C11	102.6(4)
C6-C7-C8	121.9(5)	C8-C9-C10	124.7(5)
C7-C6-C5	124.7(5)	C3-C4-C5	111.1(5)
C1-C2-C3	133.8(5)		

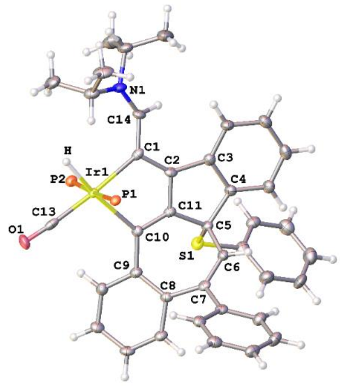

CCDC: 2254997

Supplementary Figure 35. X-ray molecular structure of **6b**. (ellipsoids set at 50% probability). The phenyl groups in the PPh_3 moieties are omitted for clarity.

Supplementary Table 14. Selected bond distances and angles for complex **6b**.

Bond Distances(Å)					
Ir1-C1	2.117(4)	C5-C6	1.499(5)	C7-C6	1.345(5)
Ir1-C10	2.178(4)	C5-C4	1.515(5)	C7-C8	1.482(5)
S1-C5	1.874(4)	C2-C1	1.390(5)	C8-C9	1.425(5)
C11-C5	1.515(5)	C2-C3	1.474(5)	C1-C13	1.436(5)
C11-C2	1.441(5)	C10-C9	1.481(5)	N1-C14	1.315(5)
C11-C10	1.361(5)	C3-C4	1.408(5)	Ir1-C13	1.881(5)

Bond Angles(°)			
C1-Ir1-C10	80.34(14)	C1-C2-C3	134.7(4)
C13-Ir1-C10	103.62(15)	C11-C10-Ir1	109.9(3)
C14-C1-Ir1	132.9(3)	C11-C10-C9	120.7(3)
C2-C11-C5	110.2(3)	C9-C10-Ir1	129.4(3)
C10-C11-C5	129.6(3)	C2-C1-Ir1	111.9(3)
C10-C11-C2	120.1(3)	C4-C3-C2	108.5(3)
C11-C5-S1	104.9(2)	C6-C7-C8	125.2(4)
C6-C5-C11	110.9(3)	C7-C6-C5	124.3(4)
C6-C5-C4	112.3(3)	C3-C4-C5	111.3(3)
C4-C5-C11	102.2(3)	C9-C8-C7	125.3(3)
C11-C2-C3	107.6(3)	C8-C9-C10	124.9(3)
C1-C2-C11	117.7(3)	C4-C5-S1	112.3(2)

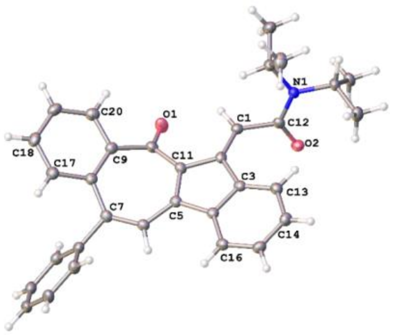

CCDC: 2254998

Supplementary Figure 36. X-ray molecular structure of **7a**. (ellipsoids set at 50% probability). The phenyl groups in the PPh_3 moieties are omitted for clarity.

Supplementary Table 15. Selected bond distances and angles for complex **7a**.

Bond Distances(Å)					
O2-C12	1.2353(17)	C2-C11	1.4812(19)	C8-C9	1.419(2)
O1-C10	1.2395(18)	C2-C1	1.348(2)	C11-C10	1.457(2)
N1-C12	1.3472(18)	C2-C3	1.475(2)	C12-C1	1.4998(19)
C5-C4	1.4756(19)	C4-C16	1.394(2)	C7-C6	1.363(2)
C5-C11	1.380(2)	C4-C3	1.4027(19)	C9-C10	1.497(2)
C5-C6	1.4311(19)	C8-C7	1.470(2)	C3-C13	1.390(2)

Bond Angles(°)			
C11-C5-C4	107.96(12)	O2-C12-C1	118.66(12)
C11-C5-C6	129.89(13)	N1-C12-C1	117.44(12)
C6-C5-C4	121.89(12)	C6-C7-C8	126.77(13)
C1-C2-C11	126.31(13)	C8-C9-C10	127.79(13)
C1-C2-C3	127.75(13)	O1-C10-C11	120.40(13)
C3-C2-C11	105.86(12)	O1-C10-C9	117.97(13)
C3-C4-C5	109.05(12)	C11-C10-C9	121.36(12)
C9-C8-C7	126.53(13)	C2-C1-C12	123.97(13)
C5-C11-C2	109.22(12)	C4-C3-C2	107.66(12)
C5-C11-C10	127.13(13)	C7-C6-C5	128.69(13)
C10-C11-C2	123.43(12)		
O2-C12-N1	123.86(13)		

Supplementary Table 16. Crystal data and structure refinement for **L** and **1**.

**Compound**	**L**	**1**·3CHCl$_3$
Empirical formula	$C_{24}H_{27}NO$	$C_{61}H_{57}Cl_1IrNO_2P_2\cdot 3CHCl_3$
Formula weight	345.46	1125.75
Temperature/K	100.0(2)	99.9(8)
Crystal system	monoclinic	triclinic
Space group	$P2_1/n$	P-1
a/Å	7.9322(2)	13.8574(3)
b/Å	13.4649(3)	16.2636(4)
c/Å	18.6639(4)	17.5276(4)
α/°	90	70.728(2)
β/°	96.452(2)	89.697(2)
γ/°	90	66.272(2)
Volume/Å3	1980.80(8)	3376.27(15)
Z	4	2
$D_{calc.}$/ g cm^{-3}	1.158	1.460
μ/mm^{-1}	0.536	8.242
F(000)	744.0	1488.0
Crystal size/mm^3	0.1 × 0.1 × 0.05	0.1 ×0.05 ×0.05
Radiation	Cu Kα (λ = 1.54184)	Cu Kα (λ = 1.54184)
2Θ range for data collection/°	8.114 to 134.794	5.4 to 150.698
Index ranges	$-9 \le h \le 9, -15 \le k \le 16, -22 \le l \le 9$	$-13 \le h \le 17, -19 \le k \le 20, -20 \le l \le 21$
Reflections collected	12234	44308
Independent reflections	3472 [R_{int} = 0.0432, R_{sigma} =0.0335]	13323 [R_{int} = 0.0424, R_{sigma} = 0.0359]
Data/restraints/parameters	3472/0/240	13323/1/730
Goodness-of-fit on F^2	1.089	1.031
Final R indexes [I>=2σ (I)]	R_1 = 0.0368, wR_2 = 0.0922	R_1 = 0.0444, wR_2 = 0.1157

Final R indexes [all data]	R_1 = 0.0532, wR_2 = 0.1065	R_1 = 0.0457, wR_2 = 0.1168
Largest diff. peak/hole / e Å^{-3}	0.28/-0.31	2.19/-1.62

Supplementary Table 17. Crystal data and structure refinement for **2a** and **2b**.

**Compound**	**2a**·$3CH_2Cl_2$	**2b**·$0.6CHCl_3$
Empirical formula	$C_{69}H_{60}B_2F_8IrNOP_2 \cdot 3CH_2Cl_2$	$C_{70}H_{62}B_2F_8IrNO_2P_2 \cdot 0.6CHCl_3$
Formula weight	1347.02	1377.04
Temperature/K	100.0(2)	100.00(10)
Crystal system	monoclinic	triclinic
Space group	$P2_1/c$	P-1
a/Å	21.7286(3)	11.9391(3)
b/Å	13.0307(2)	16.3055(4)
c/Å	24.3115(3)	17.1269(4)
α/°	90	102.697(2)
β/°	90.4460(10)	97.042(2)
γ/°	90	94.762(2)
Volume/Å3	6883.32(17)	3207.19(14)
Z	4	2
$D_{calc.}$/ g cm^{-3}	1.546	1.505
μ/mm^{-1}	6.896	5.779
F(000)	3216.0	1463.0
Crystal size/mm^3	0.05 × 0.05 × 0.02	0.1 × 0.05 × 0.05
Radiation	Cu Kα (λ = 1.54184)	Cu Kα (λ = 1.54184)
2Θ range for data collection/°	7.272 to 139.992	5.346 to 150.562
Index ranges	-26 ≤ h ≤ 26, -15 ≤ k ≤ 15, -21 ≤ l ≤ 29	-14 ≤ h ≤ 14, -19 ≤ k ≤ 20, -20 ≤ l ≤ 21

Reflections collected	46223	39007
Independent reflections	12882 [R_{int} = 0.0422, R_{sigma} = 0.0331]	12721 [R_{int} = 0.0650, R_{sigma} = 0.0601]
Data/restraints/parameters	12882/72/830	12721/37/824
Goodness-of-fit on F^2	1.210	1.033
Final R indexes [I>=2σ (I)]	R_1 = 0.0832, wR_2 = 0.2106	R_1 = 0.0762, wR_2 = 0.2031
Final R indexes [all data]	R_1 = 0.0858, wR_2 = 0.2114	R_1 = 0.0845, wR_2 = 0.2148
Largest diff. peak/hole / e Å^{-3}	3.47/-4.18	4.04/-2.84

Supplementary Table 18. Crystal data and structure refinement for **2c** and **2d**.

**Compound**	**2c**·$3CH_2Cl_2$	**2d**·$4CH_2Cl_2$
Empirical formula	$C_{70}H_{59}B_2F_{11}IrNOP_2 \cdot 3CH_2Cl_2$	$C_{75}H_{64}B_2F_8IrNOP_2 \cdot 4CH_2Cl_2$
Formula weight	1415.02	1423.11
Temperature/K	100.0(2)	99.9(4)
Crystal system	monoclinic	triclinic
Space group	$P2_1/c$	P-1
a/Å	24.9909(2)	14.7559(3)
b/Å	17.25980(10)	15.8245(3)
c/Å	17.2581(2)	16.4731(3)
α/°	90	79.663(2)
β/°	109.6040(10)	82.4500(10)
γ/°	90	87.316(2)
Volume/Å3	7012.57(11)	3750.24(13)
Z	4	2
$D_{calc.}$/ g cm^{-3}	1.582	1.560
μ/mm^{-1}	6.859	7.027
F(000)	3344.0	1770.0

Crystal size/mm^3	0.1 × 0.1 × 0.05	0.1 × 0.05 × 0.05
Radiation	Cu Kα (λ = 1.54184)	Cu Kα (λ = 1.54184)
2Θ range for data collection/°	3.754 to 148.728	5.496 to 150.244
Index ranges	-31 ≤ h ≤ 31, -21 ≤ k ≤ 17, -17 ≤ l ≤ 21	-17 ≤ h ≤ 18, -19 ≤ k ≤ 19, -19 ≤ l ≤ 20
Reflections collected	49418	43974
Independent reflections	13840 [R_{int} = 0.0609, R_{sigma} = 0.0410]	14709 [R_{int} = 0.0825, R_{sigma} = 0.0757]
Data/restraints/parameters	13840/0/918	14709/0/954
Goodness-of-fit on F^2	1.083	1.039
Final R indexes [I>=2σ (I)]	R_1 = 0.0356, wR_2 = 0.0855	R_1 = 0.0603, wR_2 = 0.1564
Final R indexes [all data]	R_1 = 0.0402, wR_2 = 0.0913	R_1 = 0.0669, wR_2 = 0.1631
Largest diff. peak/hole / e Å^{-3}	0.60/-1.32	1.81/-2.38

Supplementary Table 19. Crystal data and structure refinement for **4a** and **4d**.

**Compound**	**4a**·3.5CH_2Cl_2	**4d**·2CH_2Cl_2
Empirical formula	$C_{69}H_{61}$ $BF_4IrNO_2P_2$·3.5CH_2Cl_2	$C_{70}H_{63}BF_4IrNO_3P_2$·2$CH_2Cl_2$
Formula weight	1225.87	1307.25
Temperature/K	100.2(8)	100(1)
Crystal system	triclinic	monoclinic
Space group	P-1	P21/c
a/Å	11.6825(2)	13.1208(2)
b/Å	13.3102(2)	23.3380(3)
c/Å	23.6427(2)	22.6098(3)
α/°	95.7460(10)	90
β/°	95.6890(10)	91.8690(10)
γ/°	103.1830(10)	90

Volume/Å3	3533.15(9)	6919.74(17)
Z	2	4
$D_{calc.}$/ g cm^{-3}	1.435	1.418
μ/mm^{-1}	7.248	6.055
F(000)	1541.0	2984.0
Crystal size/mm^3	0.01 × 0.01 × 0.01	0.1 × 0.05 × 0.05
Radiation	Cu Kα (λ = 1.54184)	Cu Kα (λ = 1.54184)
2Θ range for data collection/°	6.876 to 150.804	5.444 to 129.98
Index ranges	-14 ≤ h ≤ 14, -16 ≤ k ≤ 16, -29 ≤ l ≤ 22	-15 ≤ h ≤ 15, -27 ≤ k ≤ 27, -26 ≤ l ≤ 26
Reflections collected	38405	109045
Independent reflections	13867 [R_{int} = 0.0639, R_{sigma} = 0.0613]	11695 [R_{int} = 0.0547, R_{sigma} = 0.0270]
Data/restraints/parameters	13867/6/847	11695/1/803
Goodness-of-fit on F^2	1.071	1.072
Final R indexes [I>=2σ (I)]	R_1 = 0.0536, wR_2 = 0.1468	R_1 = 0.0670, wR_2 = 0.2003
Final R indexes [all data]	R_1 = 0.0582, wR_2 = 0.1510	R_1 = 0.0704, wR_2 = 0.2017
Largest diff. peak/hole / e Å^{-3}	1.87/-1.35	6.38/-2.56

Supplementary Table 20. Crystal data and structure refinement for **4f**.

**Compound**	**4f**·$1CH_2Cl_2$	**4j**·$2CH_2Cl_2$
Empirical formula	$C_{69}H_{60}BF_4IrNO_2P_2\cdot 1CH_2Cl_2$	$C_{72}H_{66}ClIrNO_2P_2\cdot 2CH_2Cl_2$
Formula weight	1243.86	1408.14
Temperature/K	100.0(3)	100.00(10)
Crystal system	triclinic	monoclinic
Space group	P-1	P21/c
a/Å	11.6599(2)	13.8045(2)

b/Å	13.4212(2)	24.1998(4)
c/Å	23.6466(3)	40.6310(7)
$\alpha/^\circ$	95.8400(10)	90
$\beta/^\circ$	96.1210(10)	97.708(2)
$\gamma/^\circ$	102.3130(10)	90
Volume/Å3	3565.08(9)	13450.8(4)
Z	2	8
$D_{calc.}$/ g cm^{-3}	1.238	1.391
μ/mm^{-1}	5.413	6.462
F(000)	1344.0	5700.0
Crystal size/mm^3	0.1 × 0.05 × 0.05	0.2 × 0.15 × 0.13
Radiation	Cu Kα (λ = 1.54184)	Cu Kα (λ = 1.54184)
2Θ range for data collection/°	6.798 to 129.978	4.39 to 134.99
Index ranges	$-13 \le h \le 13$, $-15 \le k \le 13$, $-27 \le l \le 27$	$-16 \le h \le 16$, $-29 \le k \le 28$, $-48 \le l \le 38$
Reflections collected	39946	88741
Independent reflections	12102 [R_{int} = 0.0881, R_{sigma} = 0.0687]	24116 [R_{int} = 0.0559, R_{sigma} = 0.0543]
Data/restraints/parameters	12102/73/716	24116/878/1516
Goodness-of-fit on F^2	1.048	1.025
Final R indexes [I>=2σ (I)]	R_1 = 0.0567, wR_2 = 0.1415	R_1 = 0.0642, wR_2 = 0.1463
Final R indexes [all data]	R_1 = 0.0670, wR_2 = 0.1452	R_1 = 0.0772, wR_2 = 0.1521
Largest diff. peak/hole / e Å^{-3}	3.24/-1.41	2.22/-1.92

Supplementary Table 21. Crystal data and structure refinement for **3a** and **3x**.

**Compound**	**3a**·1CH_2Cl_2	**3x**·3CH_2Cl_2

Empirical formula	$C_{69}H_{61}F_6IrNOP_3\cdot 2CH_2Cl_2$	$C_{75}H_{63}BF_4IrNOP_2\cdot 3CH_2Cl_2$
Formula weight	1319.38	1335.30
Temperature/K	99.9(6)	100.0(9)
Crystal system	monoclinic	monoclinic
Space group	$P2_1/n$	$P2_1/c$
a/Å	21.6406(4)	13.4692(2)
b/Å	21.9709(4)	22.8852(3)
c/Å	28.1813(5)	23.1796(3)
α/°	90	90
β/°	101.917(2)	93.1140(10)
γ/°	90	90
Volume/Å3	13110.4(4)	7134.45(17)
Z	8	4
$D_{calc.}$/ g cm^{-3}	1.509	1.513
μ/mm^{-1}	6.645	2.235
F(000)	6000.0	3276.0
Crystal size/mm^3	0.5 × 0.2 × 0.1	0.1 × 0.1 × 0.05
Radiation	Cu Kα (λ = 1.54184)	Mo Kα (λ = 0.71073)
2Θ range for data collection/°	4.708 to 135	3.944 to 62.296
Index ranges	-25 ≤ h ≤ 23, -25 ≤ k ≤ 26, -33 ≤ l ≤ 33	-18 ≤ h ≤ 18, -32 ≤ k ≤ 31, -32 ≤ l ≤ 32
Reflections collected	90955	122149
Independent reflections	23578 [R_{int} = 0.0467, R_{sigma} = 0.0410]	20427 [R_{int} = 0.0470, R_{sigma} = 0.0358]
Data/restraints/parameters	23578/1/1583	20427/0/873
Goodness-of-fit on F^2	1.049	1.039
Final R indexes [I>=2σ (I)]	R_1 = 0.0466, wR_2 = 0.1116	R_1 = 0.0372, wR_2 = 0.0955
Final R indexes [all data]	R_1 = 0.0544, wR_2 = 0.1143	R_1 = 0.0505, wR_2 = 0.1012

Largest diff. peak/hole / e Å^{-3}	2.50/-1.62	2.24/-1.67

Supplementary Table 22. Crystal data and structure refinement for **6a** and **6b**.

**Compound**	**6a**	**6b**·CH_2Cl_2
Empirical formula	$C_{70}H_{63}BF_4IrNOP_2S$	$C_{75}H_{67}BF_4IrNOP_2S{\cdot}CH_2Cl_2$
Formula weight	1307.22	1447.12
Temperature/K	100.00(10)	100.00(10)
Crystal system	triclinic	monoclinic
Space group	P-1	$P2_1/c$
a/Å	11.7766(3)	12.9378(2)
b/Å	13.7614(4)	21.8657(3)
c/Å	21.3934(5)	23.0720(3)
α/°	99.879(2)	90
β/°	96.979(2)	95.6070(10)
γ/°	105.888(2)	90
Volume/Å3	3232.83(15)	6495.70(16)
Z	2	4
$D_{calc.}$/ g cm^{-3}	1.343	1.480
μ/mm^{-1}	5.195	5.896
F(000)	1324.0	2930.0
Crystal size/mm^3	0.155 × 0.126 × 0.08	0.1 × 0.05 × 0.03
Radiation	Cu Kα (λ = 1.54184)	Cu Kα (λ = 1.54184)
2Θ range for data collection/°	6.838 to 129.992	5.582 to 134.994
Index ranges	-10 ≤ h ≤ 13, -16 ≤ k ≤ 16, -25 ≤ l ≤ 25	-15 ≤ h ≤ 15, -26 ≤ k ≤ 26, -27 ≤ l ≤ 25
Reflections collected	37098	72397

Independent reflections	10941 [R_{int} = 0.0870, R_{sigma} = 0.0798]	11642 [R_{int} = 0.0855, R_{sigma} = 0.0458]
Data/restraints/parameters	10941/1/739	11642/0/810 1.039
Goodness-of-fit on F^2	1.060	1.065
Final R indexes [I>=2σ (I)]	R_1 = 0.0613, wR_2 = 0.1550	R_1 = 0.0384, wR_2 = 0.1021
Final R indexes [all data]	R_1 = 0.0664, wR_2 = 0.1600	R_1 = 0.0446, wR_2 = 0.1054
Largest diff. peak/hole / e $Å^{-3}$	5.23/-1.69	1.45/-1.78

Supplementary Table 23. Crystal data and structure refinement for **7a.**

**Compound**	**7a**
Empirical formula	$C_{32}H_{29}NO_2$
Formula weight	468.42
Temperature/K	100.00(1)
Crystal system	monoclinic
Space group	$P2_1/c$
a/Å	11.1157(5)
b/Å	22.2168(8)
c/Å	10.7360(5)
α/°	90
β/°	114.892(5)
γ/°	90
Volume/Å3	2405.0(2)
Z	4
$D_{calc.}$/ g cm^{-3}	1.294
μ/mm^{-1}	0.871
F(000)	993.0
Crystal size/mm^3	0.5 × 0.2 × 0.1

Radiation	Cu Kα (λ = 1.54184)
2Θ range for data collection/°	7.96 to 134.966
Index ranges	-9 ≤ h ≤ 13, -26 ≤ k ≤ 26, -12 ≤ l ≤ 12
Reflections collected	14455
Independent reflections	4309 [R_{int} = 0.0417, R_{sigma} = 0.0413]
Data/restraints/parameters	4309/0/320
Goodness-of-fit on F^2	1.059
Final R indexes [I>=2σ (I)]	R_1 = 0.0394, wR_2 = 0.1000
Final R indexes [all data]	R_1 = 0.0483, wR_2 = 0.1053
Largest diff. peak/hole / e $Å^{-3}$	0.22/-0.20

12. Computational methods

Computational methods: All structures were optimized at the B3LYP level of density functional theory.[22-24] Frequency calculations were performed to confirm the characteristics of all the calculated structures as minima. Compounds **2a** is simplified through the way that the PH_3 groups were used to replace the PPh_3 ligands, which are named simplified model compounds **2a**'. All these structures evaluated were optimized by B3LYP-D3BJ/6–31G*;[25-29] The nucleus-independent chemical shifts (NICS)[2-4] and anisotropy of the induced current density (ACID)[5,6]calculations were performed at the B3LYP/6-31G* level. The Condensed dual descriptor (CDD)[7] were performed at the B3LYP-D3BJ/6-311G**. The effective core potentials (ECPs) of Hay and Wadt with a double-ζ valence basis set LanL2DZ[8,9] for **2a**' was used to describe Ir and P atoms. Polarization functions were added for Ir (ζ(f) = 0.938), P (ζ(d) = 0.340).[10,11] The single-point energy calculations were performed on the mechanism using the B3LYP-D3BJ/Def2-TZVP method with the SMD solvation method in DCM.[30,31] Whereas the UV-Vis-NIR spectrum was used TD-DFT calculations[32] at the B3LYP-D3BJ/Def2-TZVP level of DFT with an SMD solvation model in DCM. All calculations were performed with the Gaussian 16 software package.[12]

13. NMR and HRMS Spectra

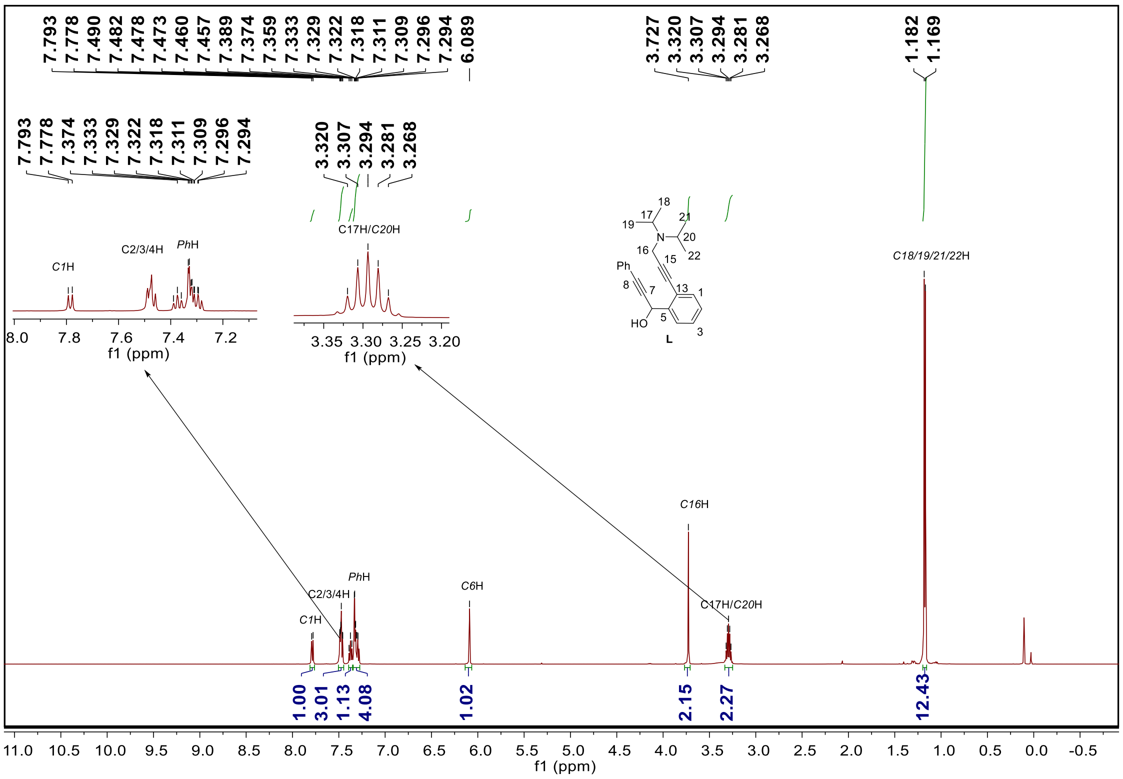

Supplementary Figure 37. The ^{1}H NMR (500.2 MHz, $CDCl_3$) spectrum for complex **L**.

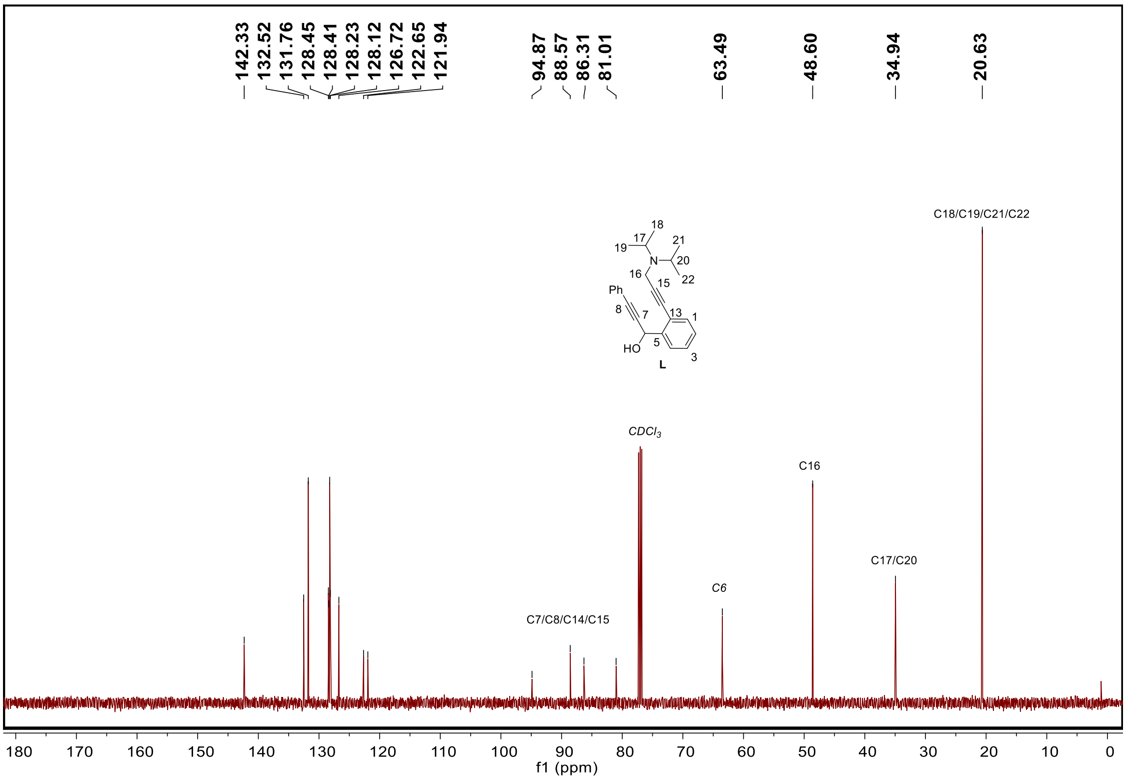

Supplementary Figure 38. The $^{13}C\{^{1}H\}$ NMR (125.8 MHz, $CDCl_3$) spectrum for complex **L**

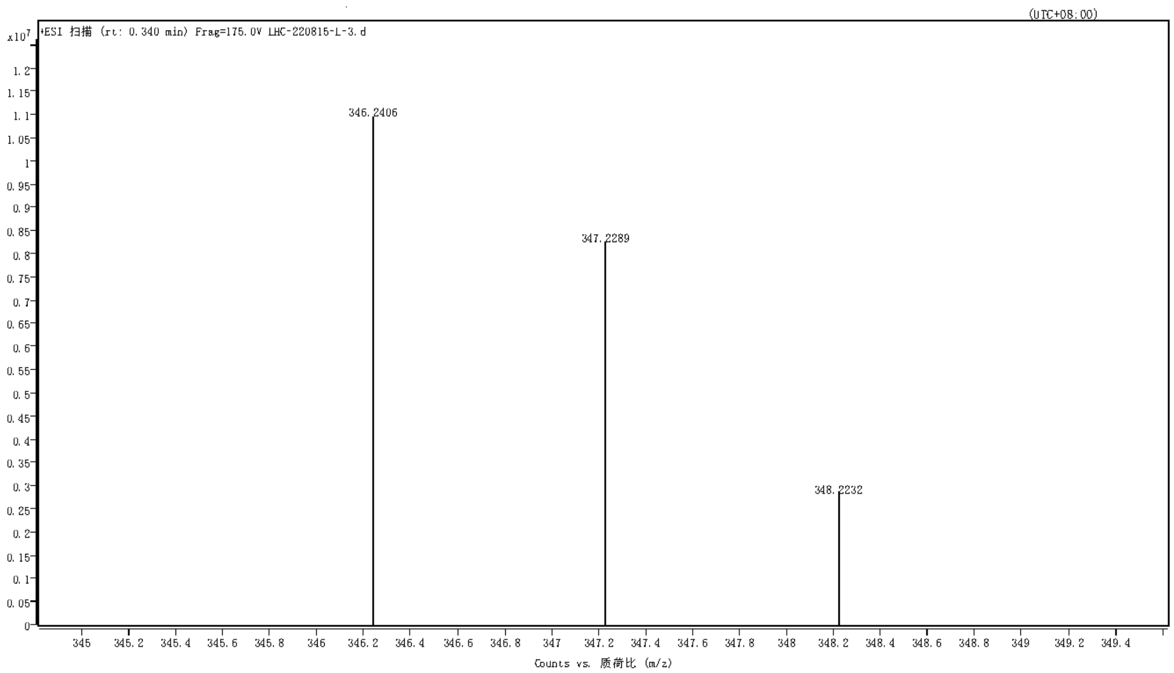

Supplementary Figure 39. Positive-ion ESI-MS spectrum for complex [**L**+H^+]$^+$ measured in methanol.

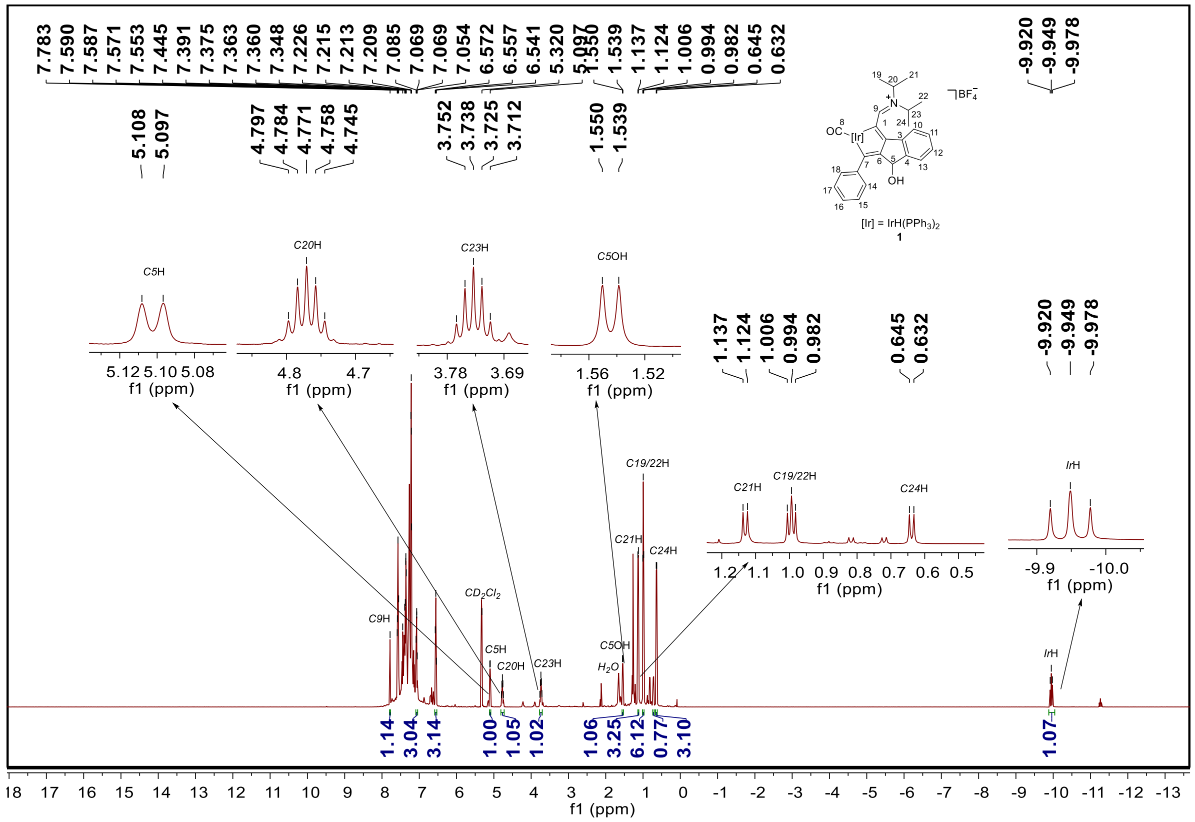

Supplementary Figure 40. The ^{1}H NMR (500.2 MHz, CD_2Cl_2) spectrum for complex **1**.

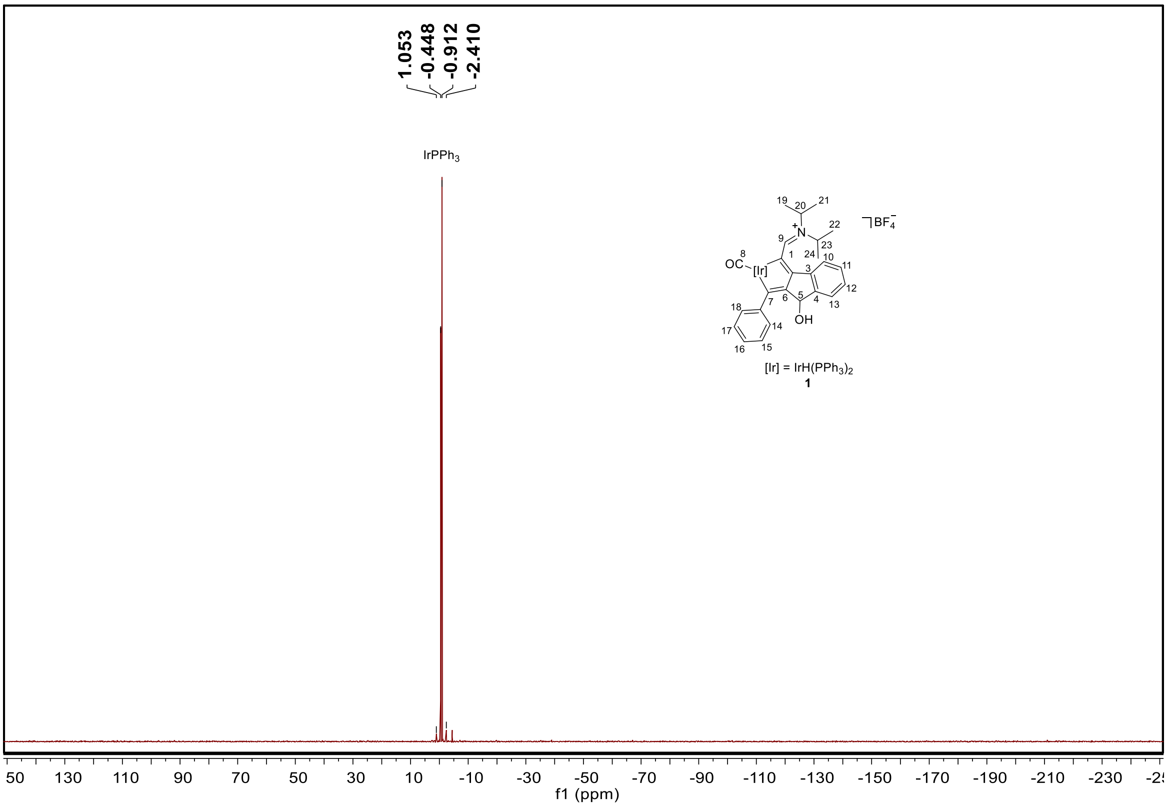

Supplementary Figure 41. The $^{31}P\{^{1}H\}$ NMR spectrum (202.5 MHz, CD_2Cl_2) for complex **1**.

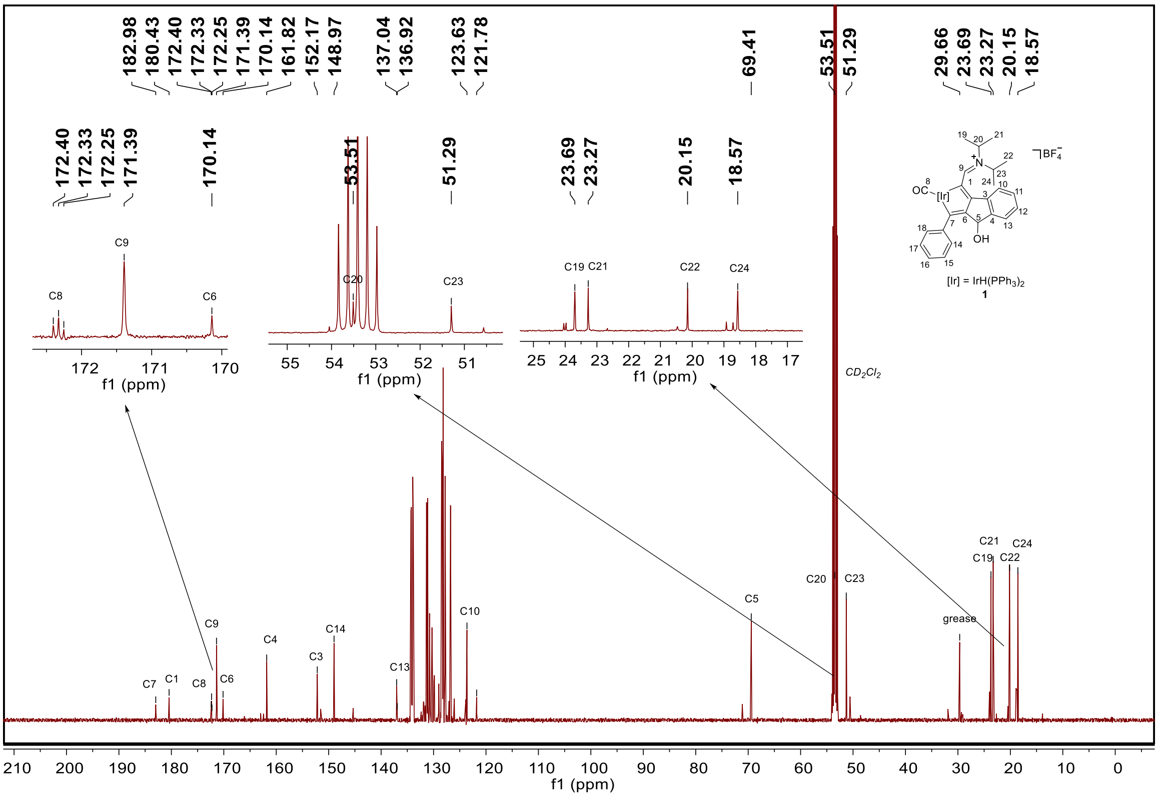

Supplementary Figure 42. The $^{13}C\{^{1}H\}$ NMR (125.8 MHz, CD_2Cl_2) spectrum for complex **1**

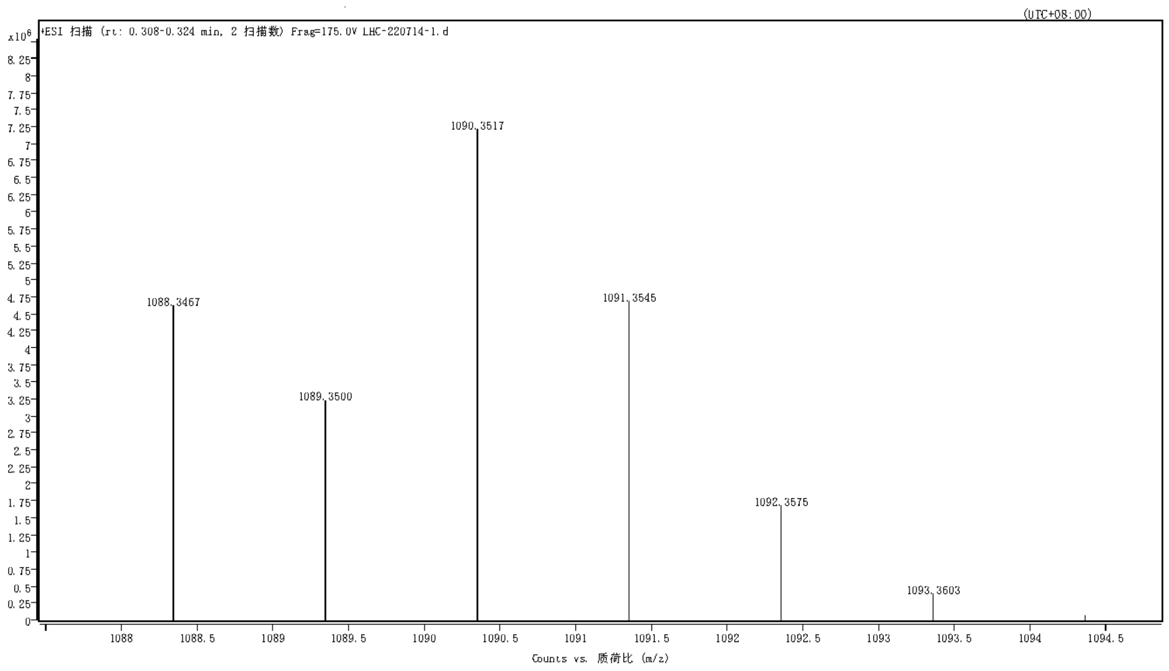

Supplementary Figure 43. Positive-ion ESI-MS spectrum for complex [**1**]$^+$ measured in methanol.

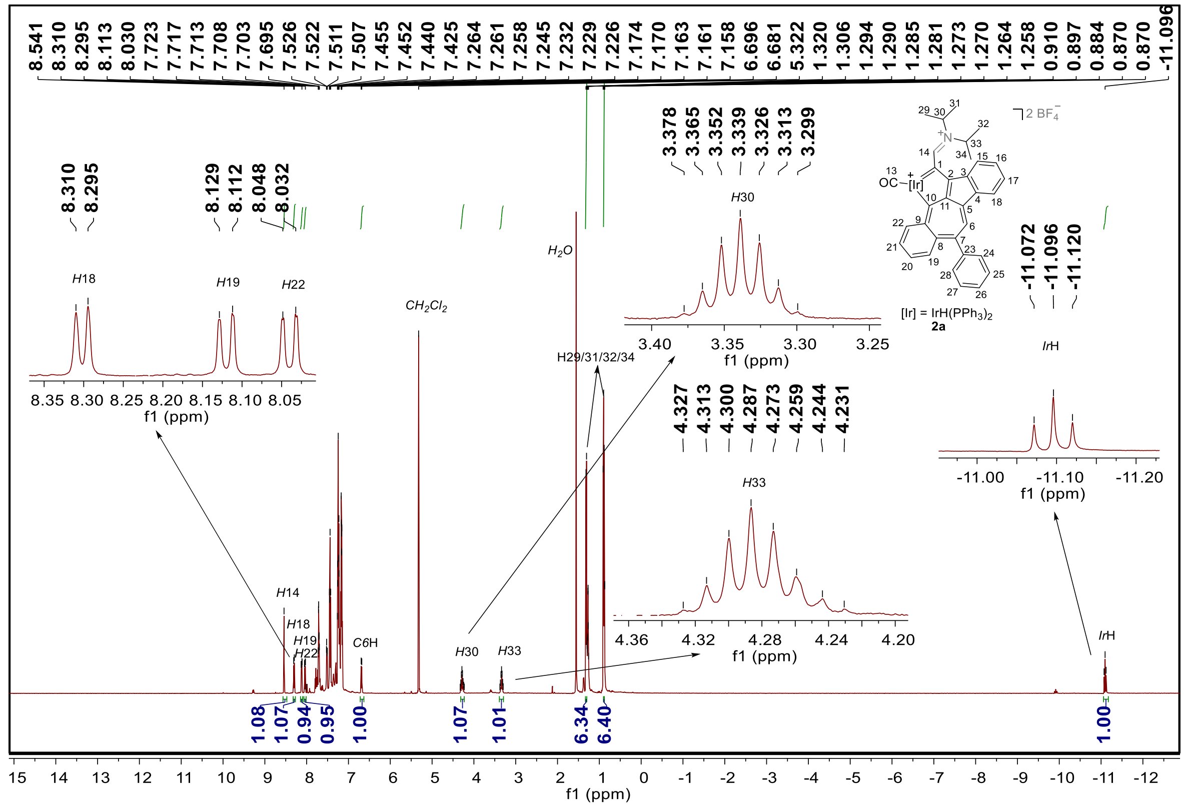

Supplementary Figure 44. The ^{1}H NMR (500.2 MHz, CD_2Cl_2) spectrum for complex **2a**.

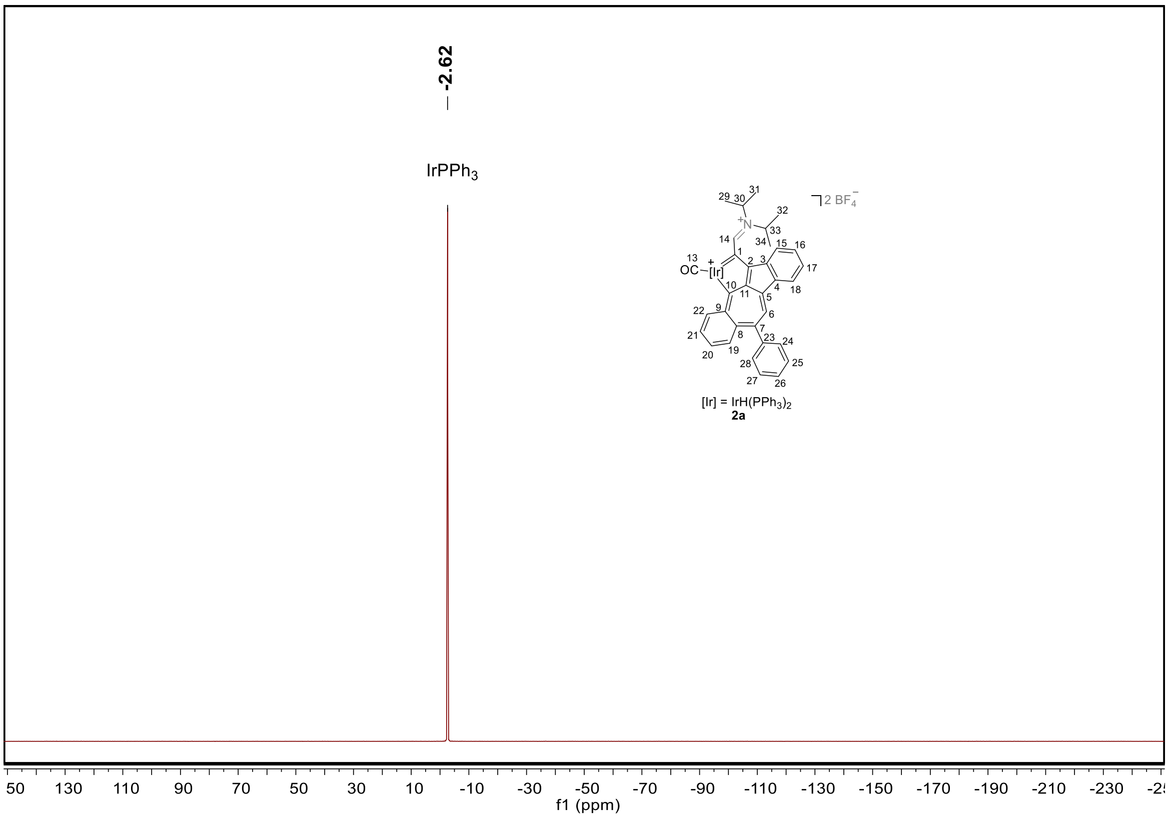

Supplementary Figure 45. The $^{31}P\{^{1}H\}$ NMR spectrum (202.5 MHz, CD_2Cl_2) for complex **2a**.

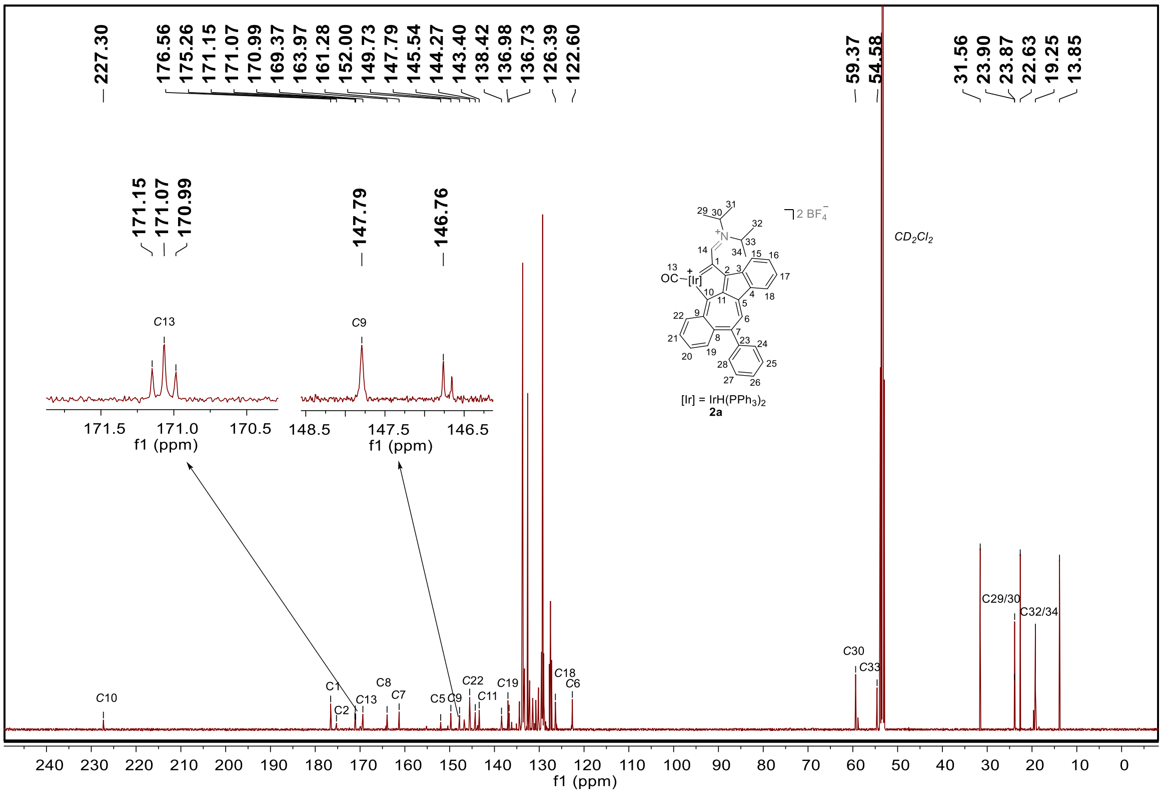

Supplementary Figure 46. The $^{13}C\{^{1}H\}$ NMR (125.8 MHz, CD_2Cl_2) spectrum for complex **2a.**

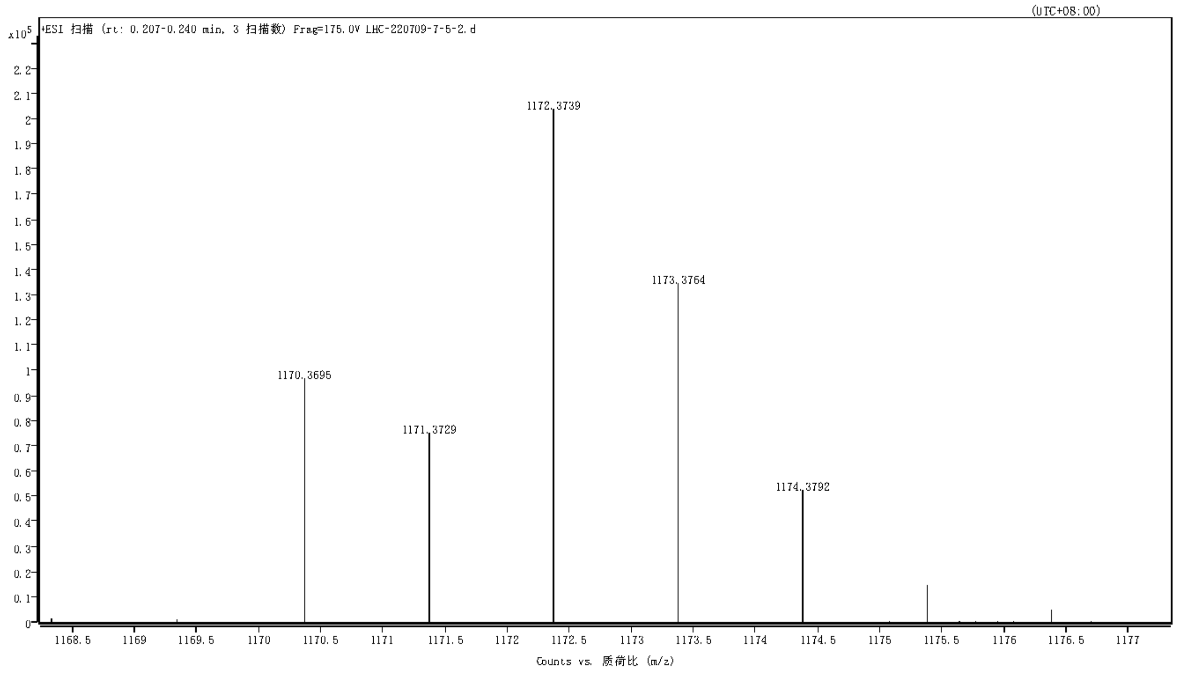

Supplementary Figure 47. Positive-ion ESI-MS spectrum for complex [**2a**-H$^+$]$^+$ measured in methanol.

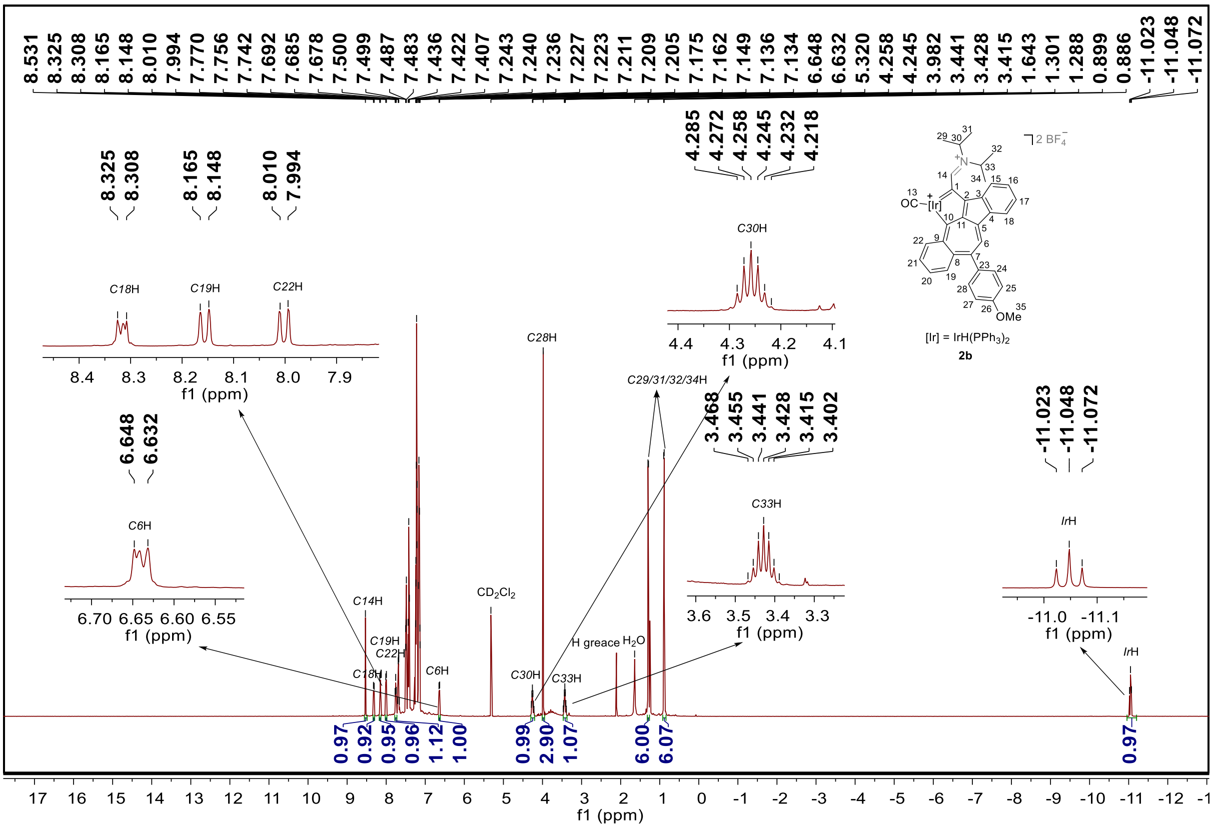

Supplementary Figure 48. The ^{1}H NMR (500.2 MHz, CD_2Cl_2) spectrum for complex **2b**.

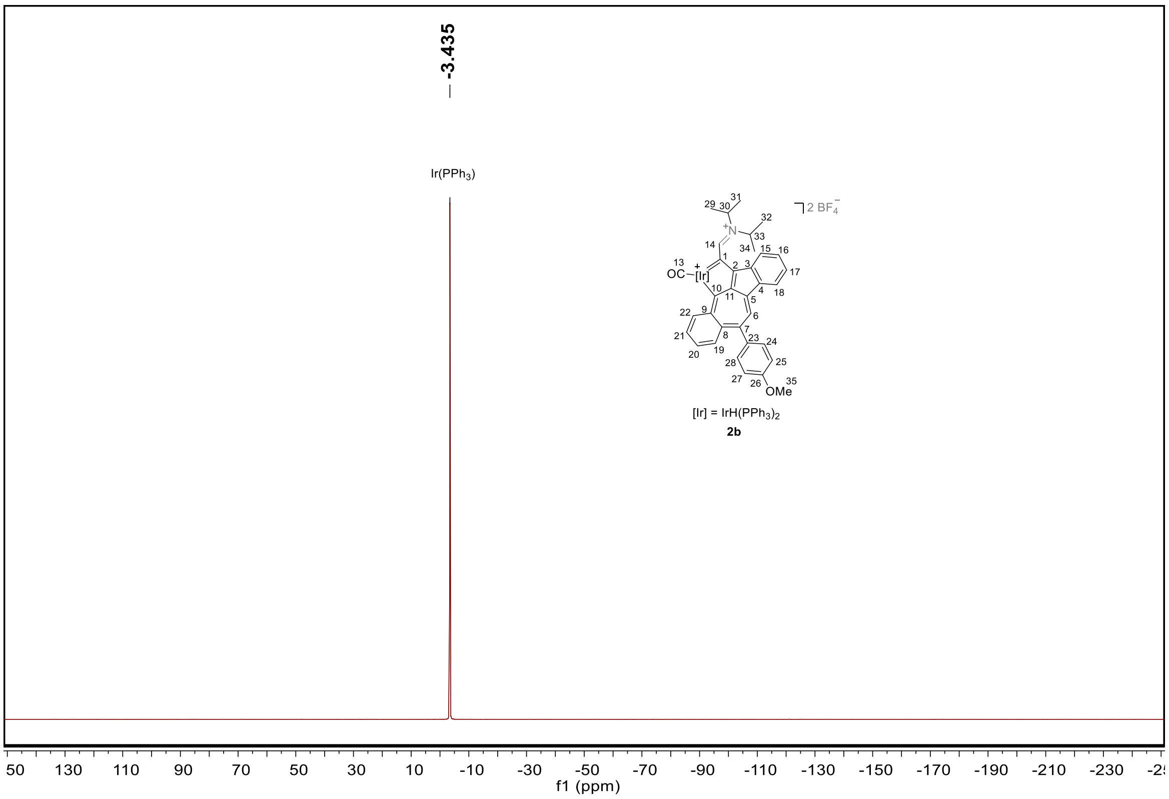

Supplementary Figure 49. The $^{31}P\{^{1}H\}$ NMR spectrum (202.5 MHz, CD_2Cl_2) for complex **2b**.

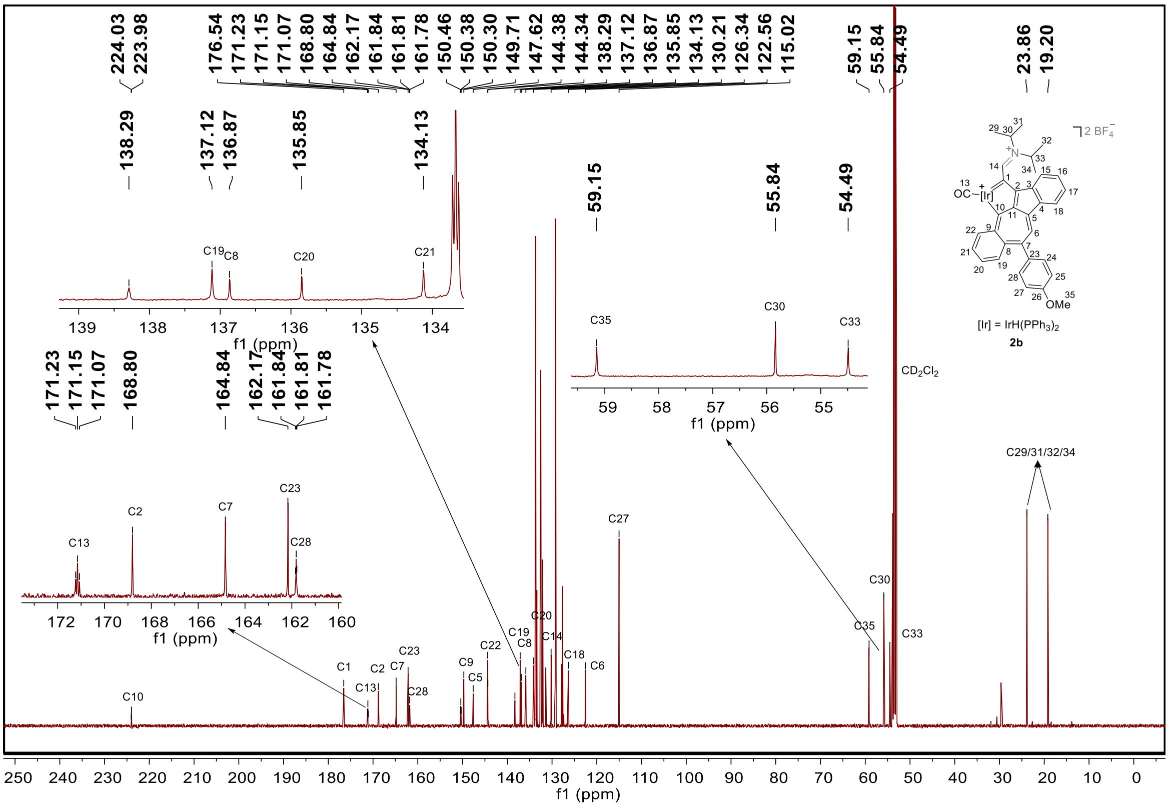

Supplementary Figure 50. The $^{13}C\{^{1}H\}$ NMR (125.8 MHz, CD_2Cl_2) spectrum for complex **2b.**

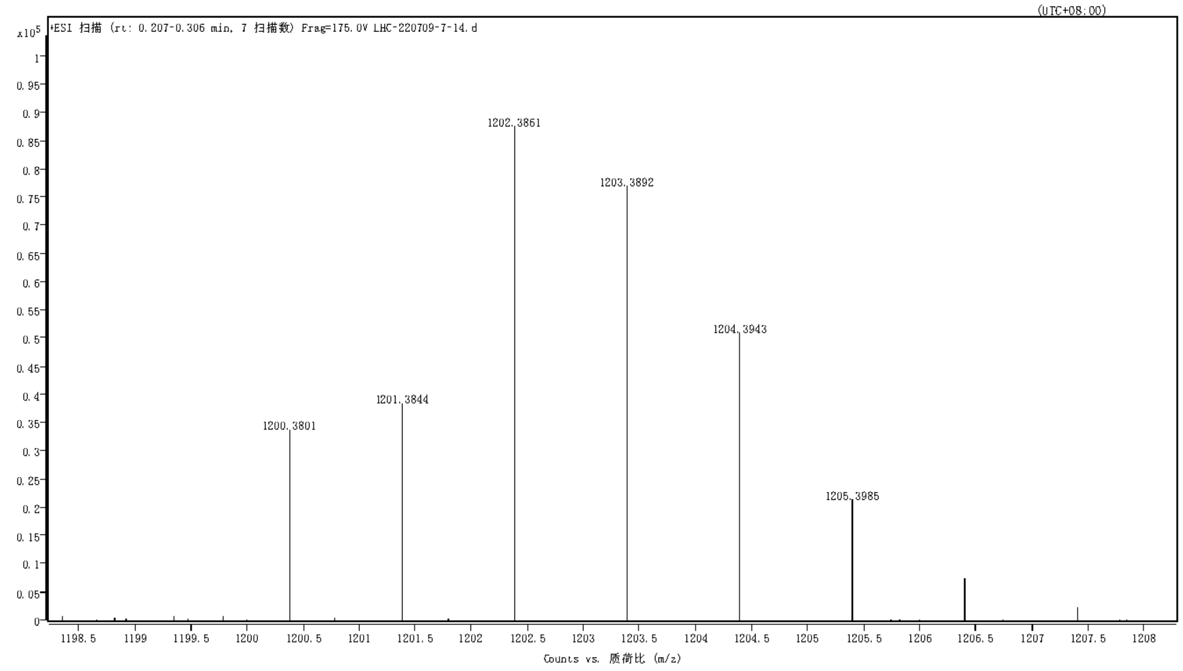

Supplementary Figure 51. Positive-ion ESI-MS spectrum for complex [**2b**-H$^+$]$^+$ measured in methanol.

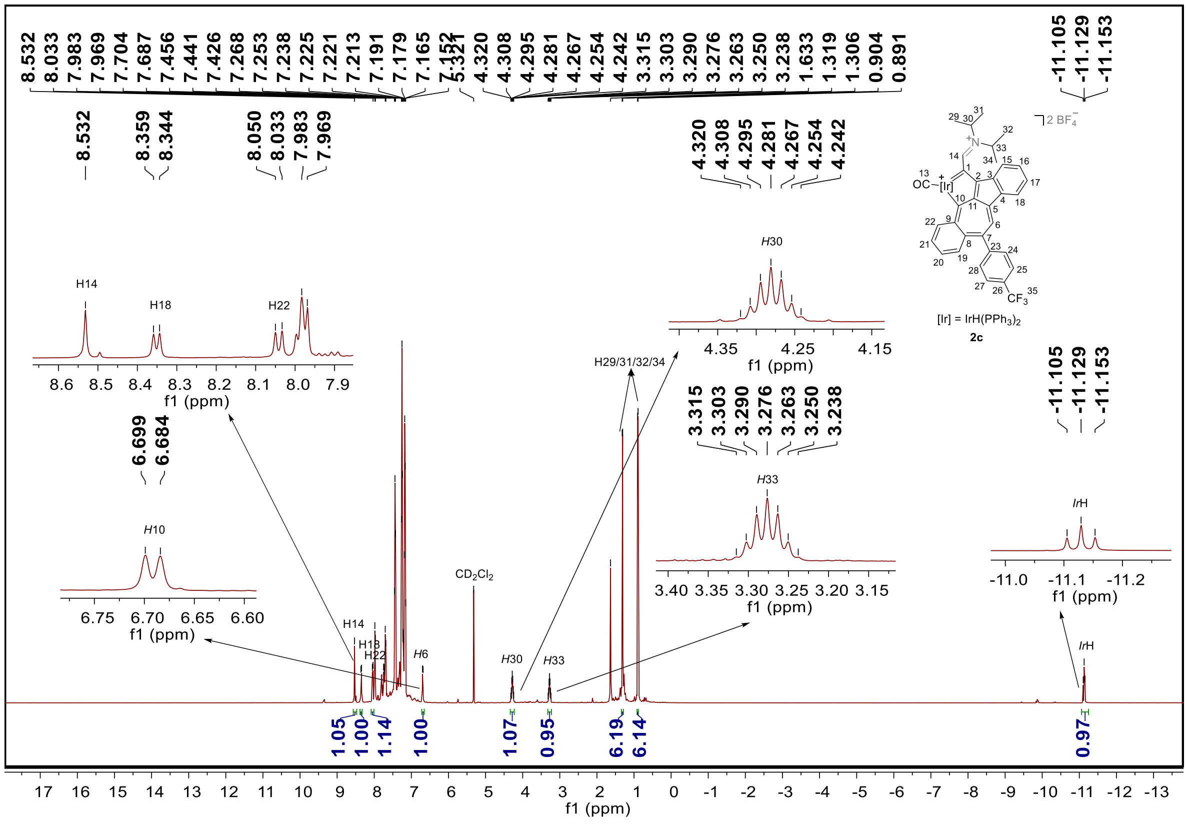

Supplementary Figure 52. The ^{1}H NMR (500.2 MHz, CD_2Cl_2) spectrum for complex **2c**.

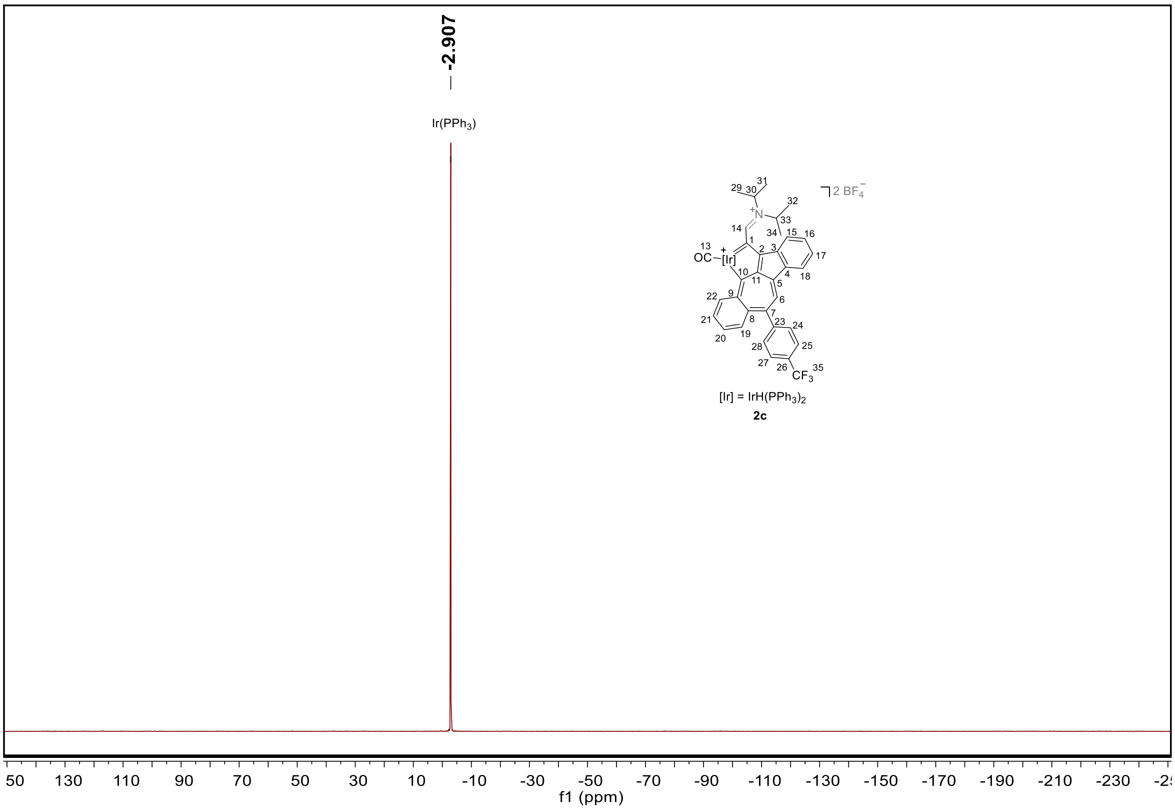

Supplementary Figure 53. The $^{31}P\{^{1}H\}$ NMR spectrum (202.5 MHz, CD_2Cl_2) for complex **2c**.

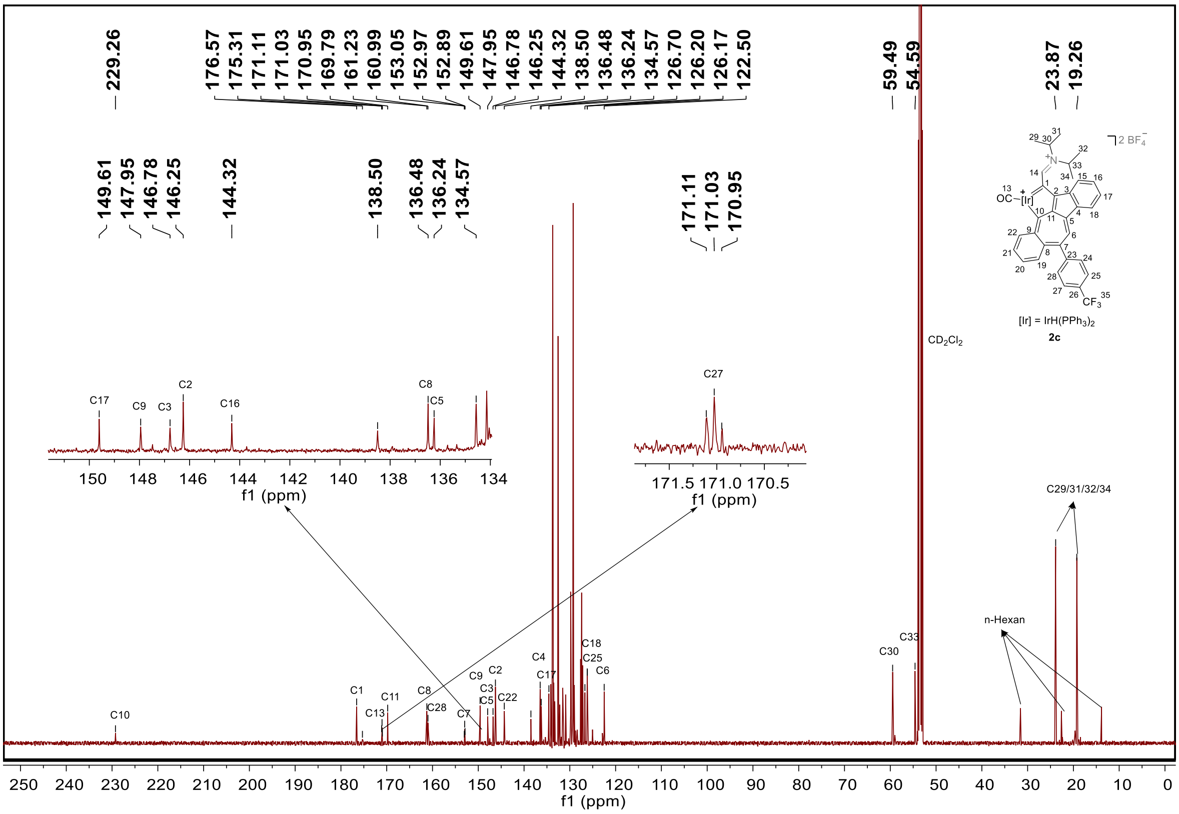

Supplementary Figure 54. The $^{13}C\{^{1}H\}$ NMR (125.8 MHz, CD_2Cl_2) spectrum for complex **2c.**

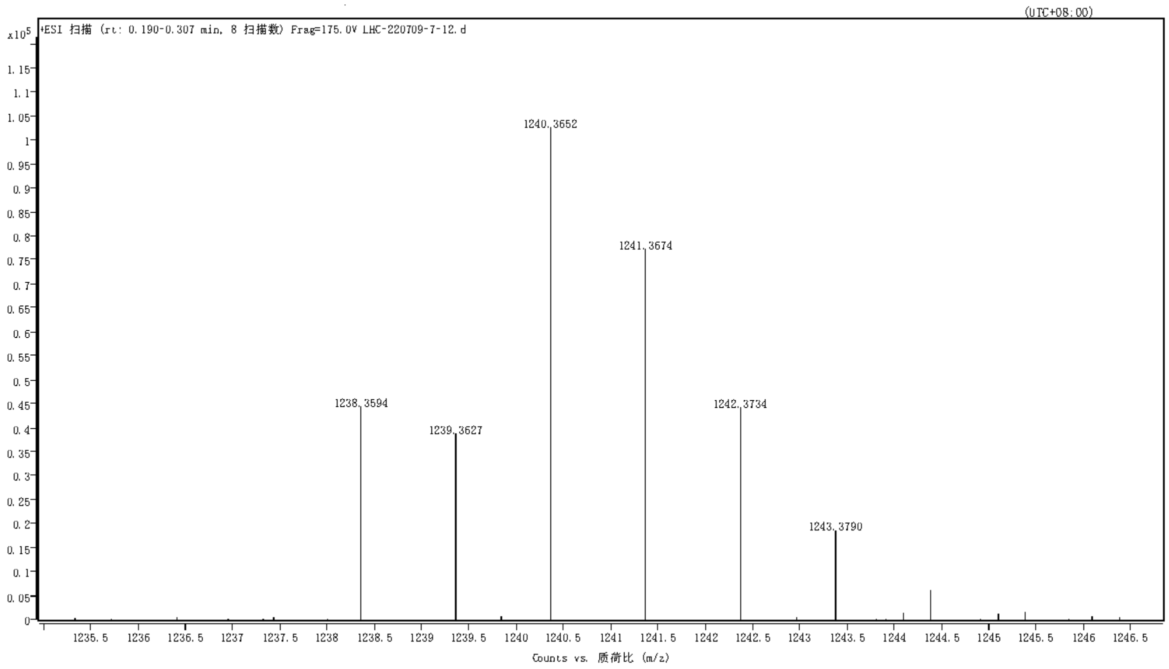

Supplementary Figure 55. Positive-ion ESI-MS spectrum for complex [**2c**-H$^+$]$^+$ measured in methanol.

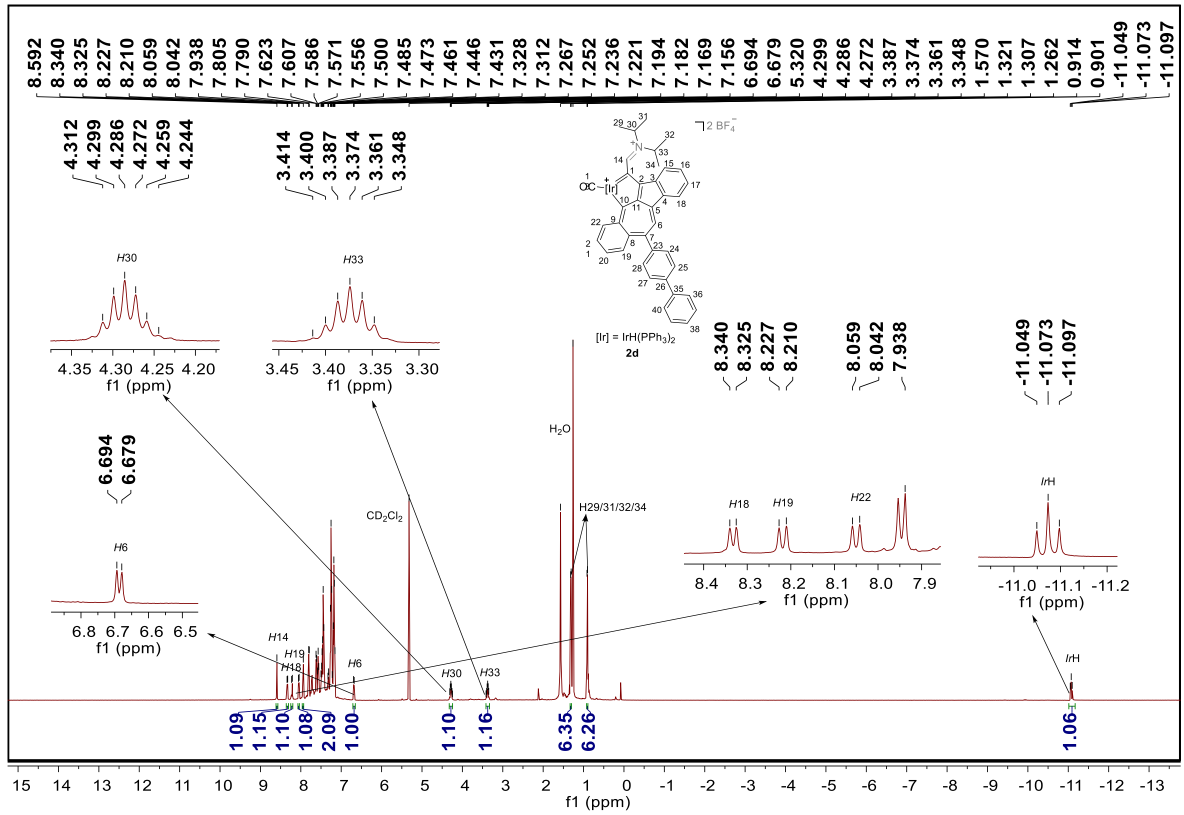

Supplementary Figure 56. The ^{1}H NMR (500.2 MHz, CD_2Cl_2) spectrum for complex **2d**.

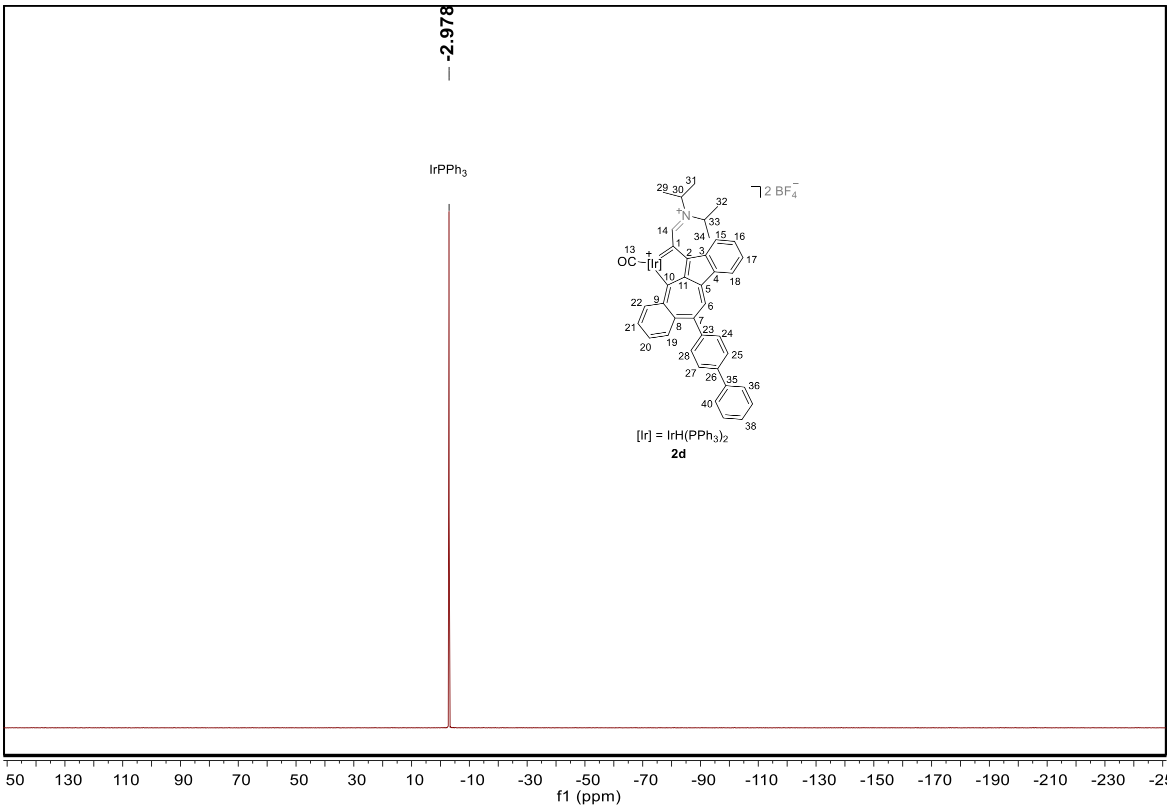

Supplementary Figure 57. The $^{31}P\{^1H\}$ NMR spectrum (202.5 MHz, CD_2Cl_2) for complex **2d**.

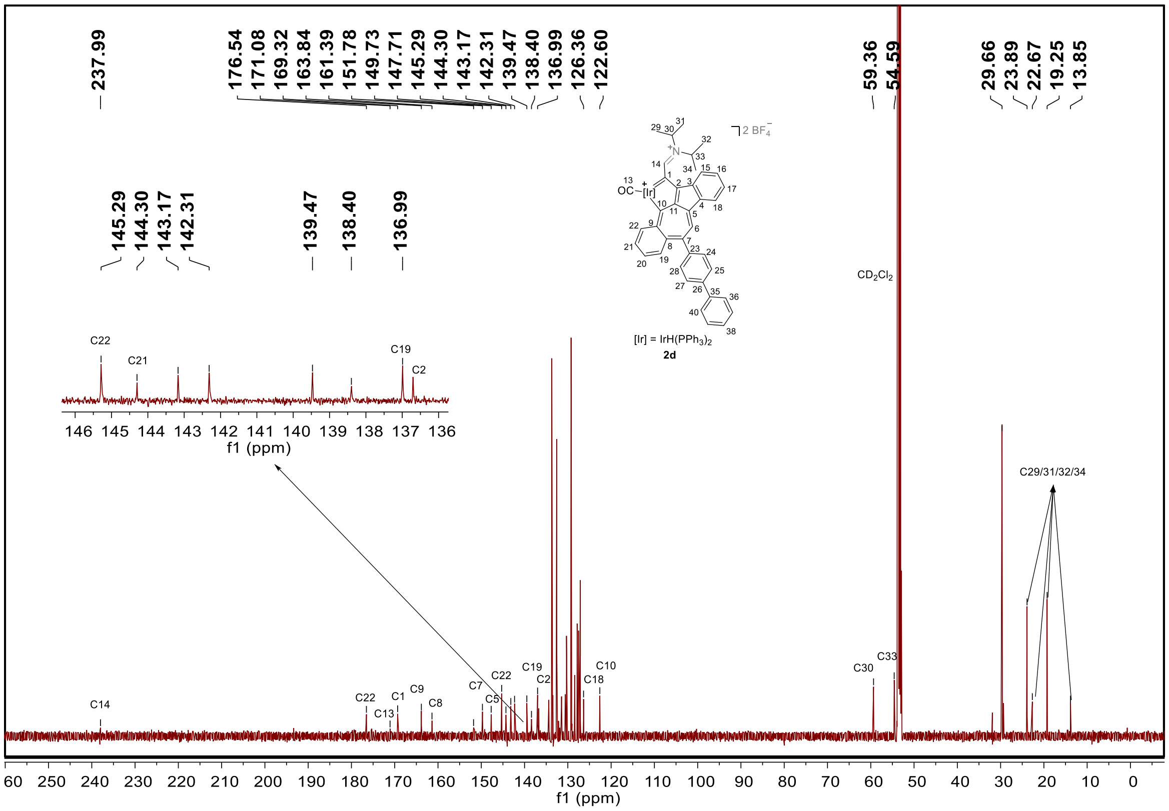

Supplementary Figure 58. The $^{13}C\{^1H\}$ NMR (125.8 MHz, CD_2Cl_2) spectrum for complex **2d.**

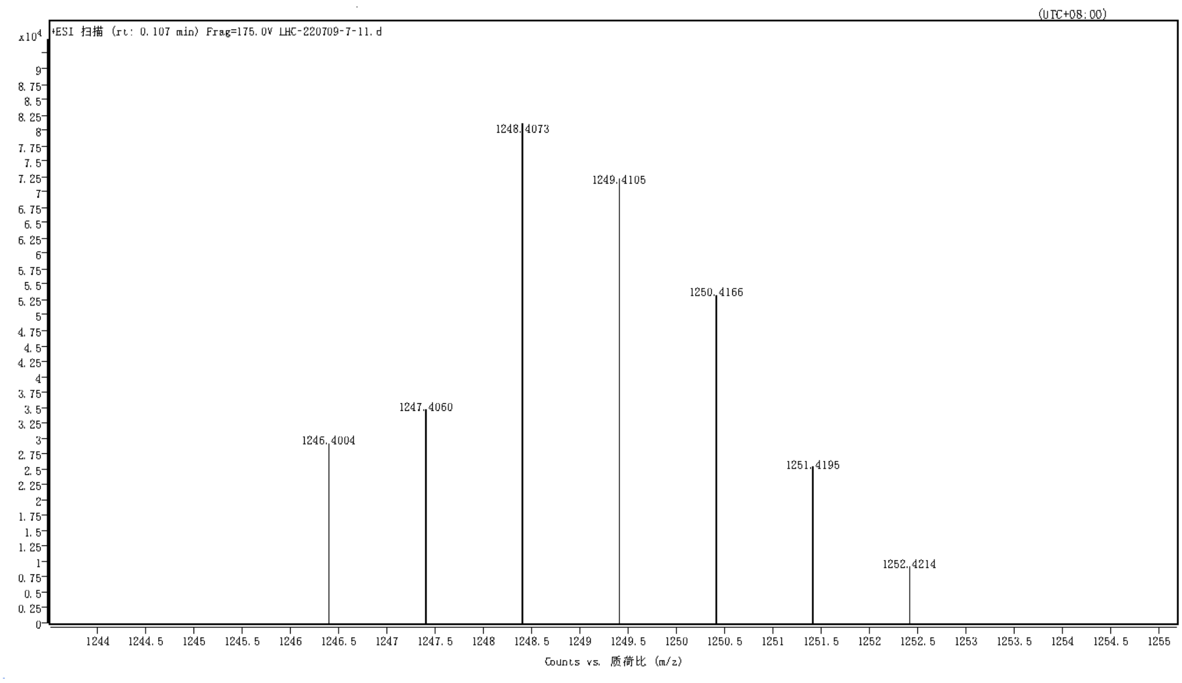

Supplementary Figure 59. Positive-ion ESI-MS spectrum for complex [**2d**-H^+]$^+$ measured in methanol

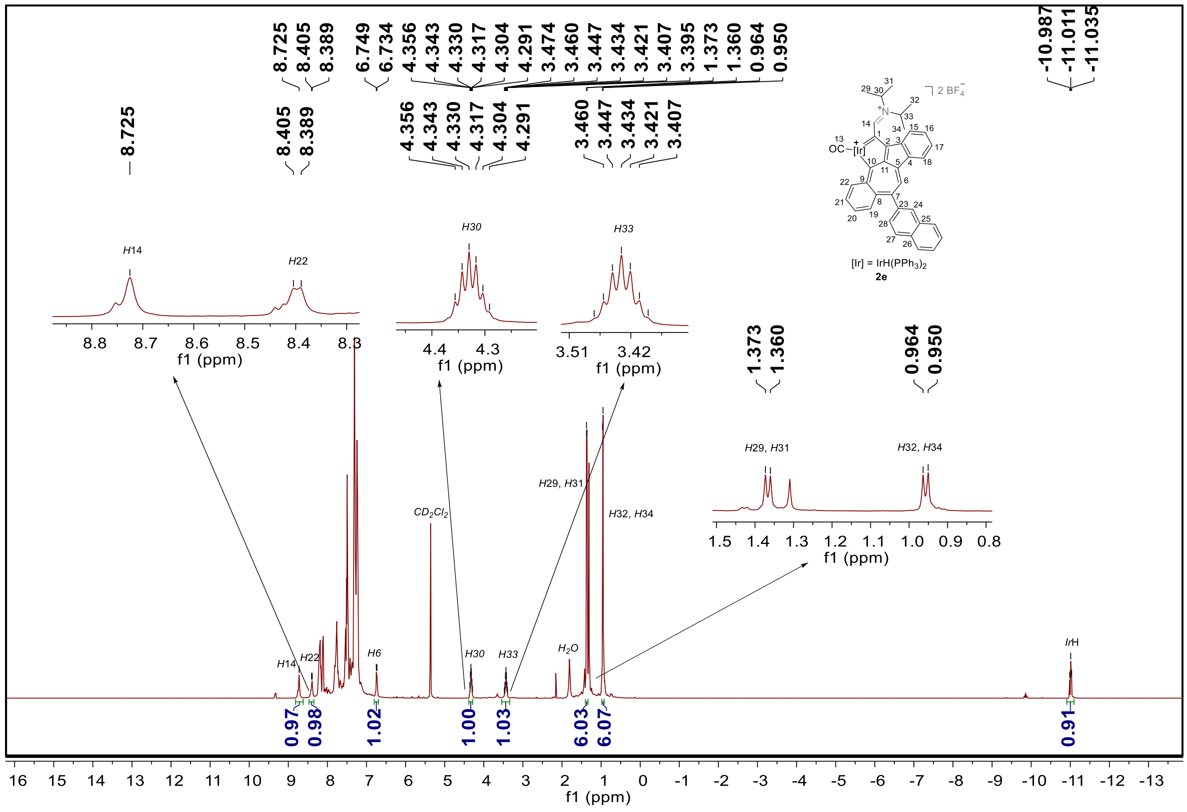

Supplementary Figure 60. The ^{1}H NMR (500.2 MHz, CD_2Cl_2) spectrum for complex **2e**.

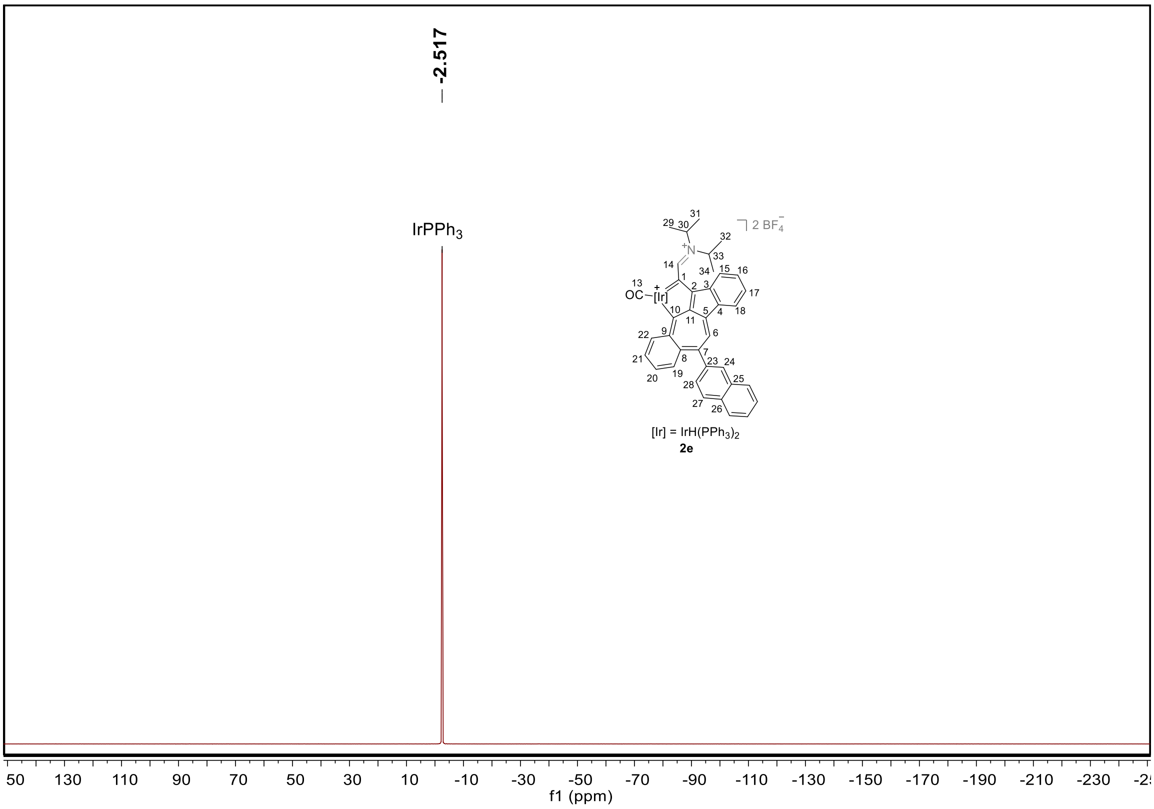

Supplementary Figure 61. The $^{31}P\{^{1}H\}$ NMR spectrum (202.5 MHz, CD_2Cl_2) for complex **2e**.

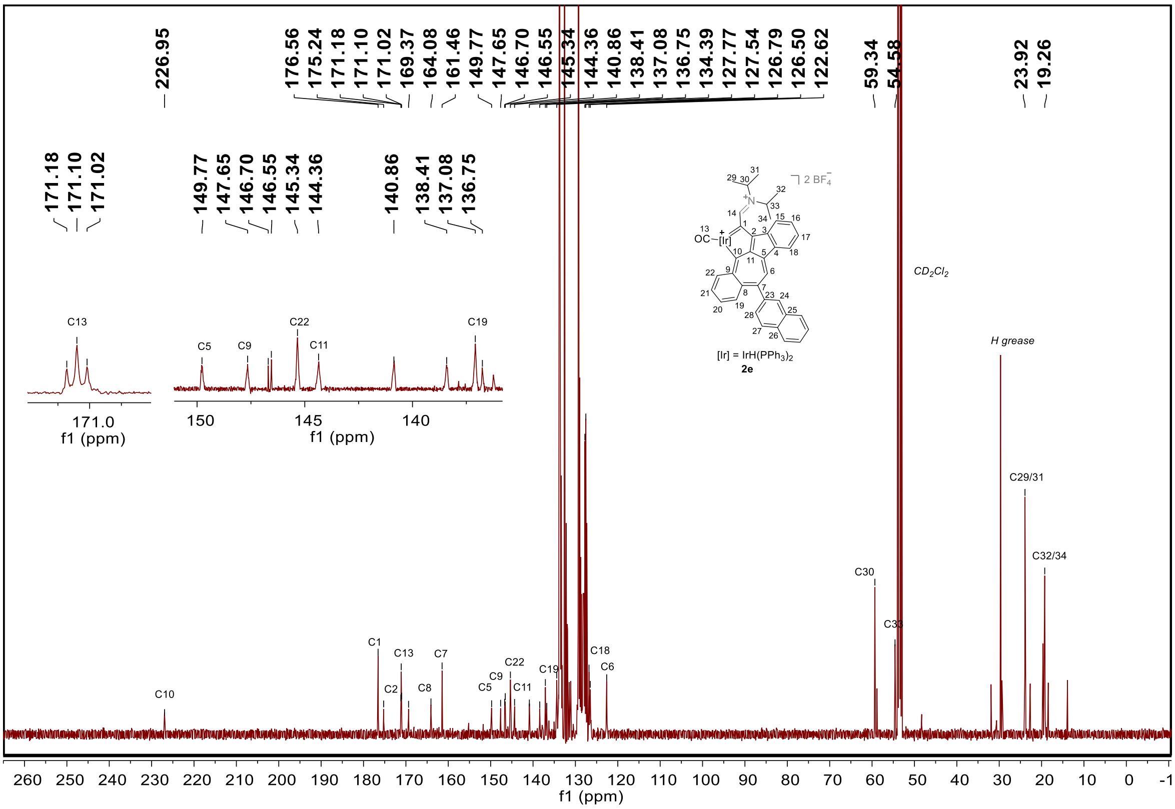

Supplementary Figure 62. The $^{13}C\{^{1}H\}$ NMR (125.8 MHz, CD_2Cl_2) spectrum for complex **2e.**

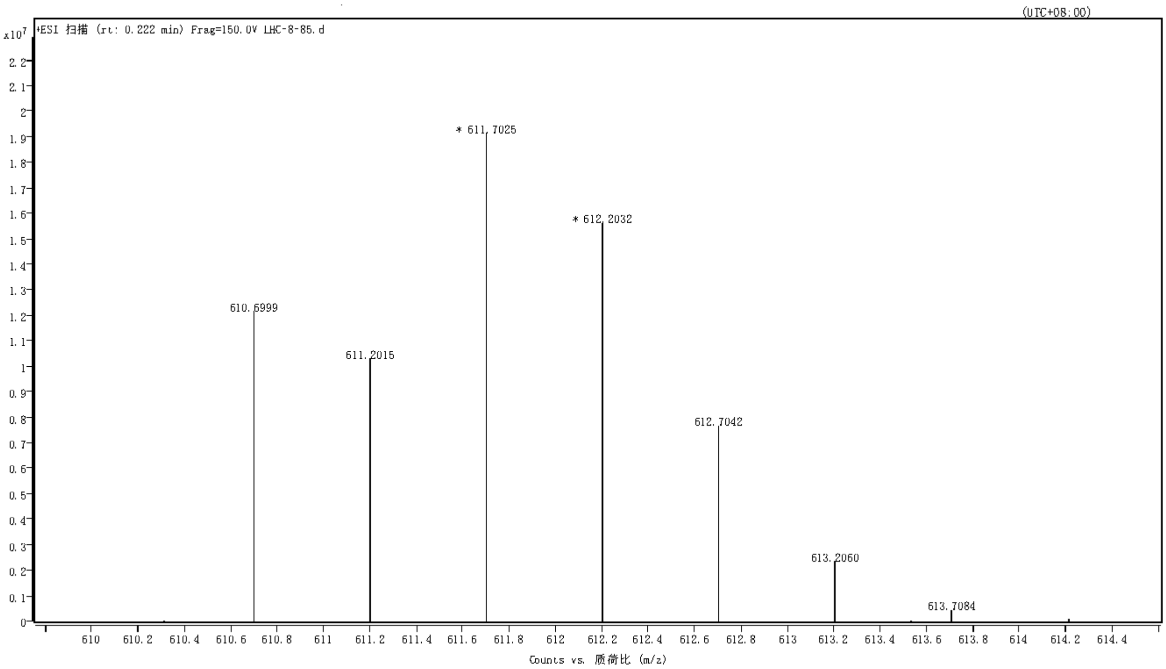

Supplementary Figure 63. Positive-ion ESI-MS spectrum for complex [**2e**]$^{2+}$ measured in methanol.

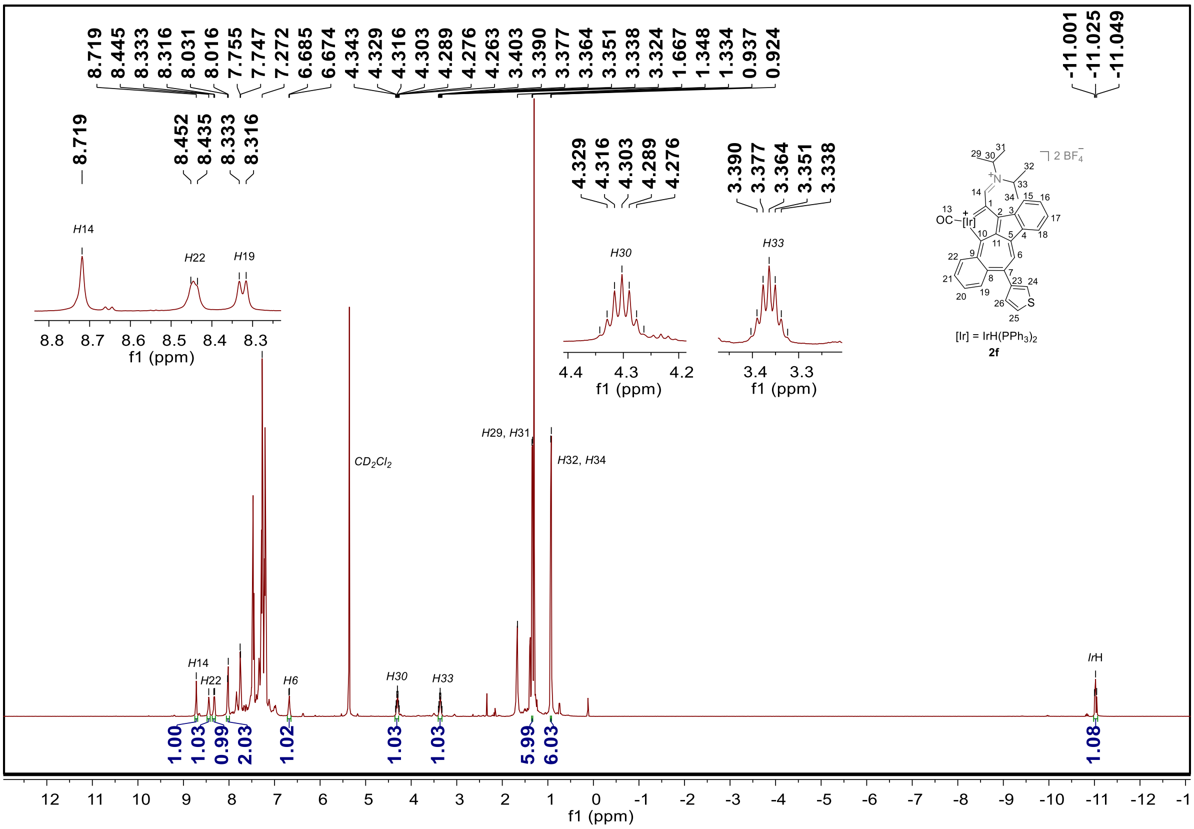

Supplementary Figure 64. The ^{1}H NMR (500.2 MHz, CD_2Cl_2) spectrum for complex **2f.**

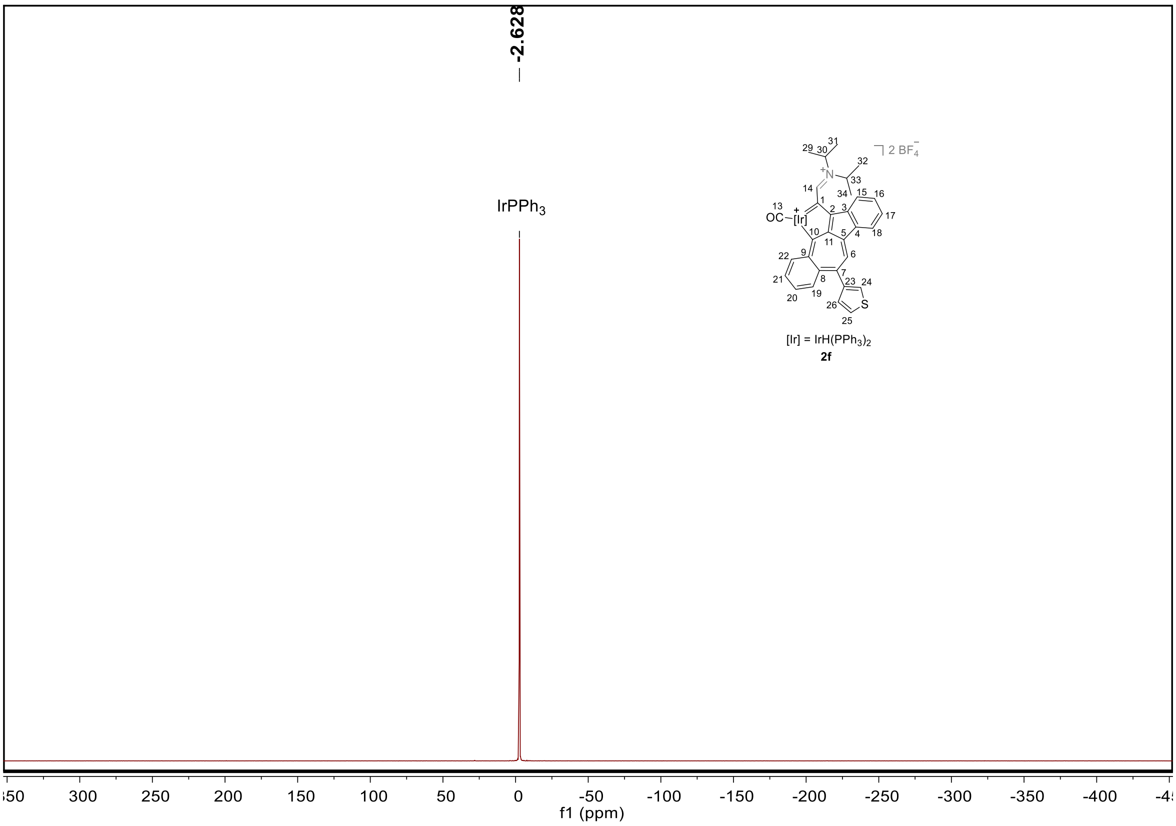

Supplementary Figure 65. The $^{31}P\{^{1}H\}$ NMR spectrum (202.5 MHz, CD_2Cl_2) for complex **2f.**

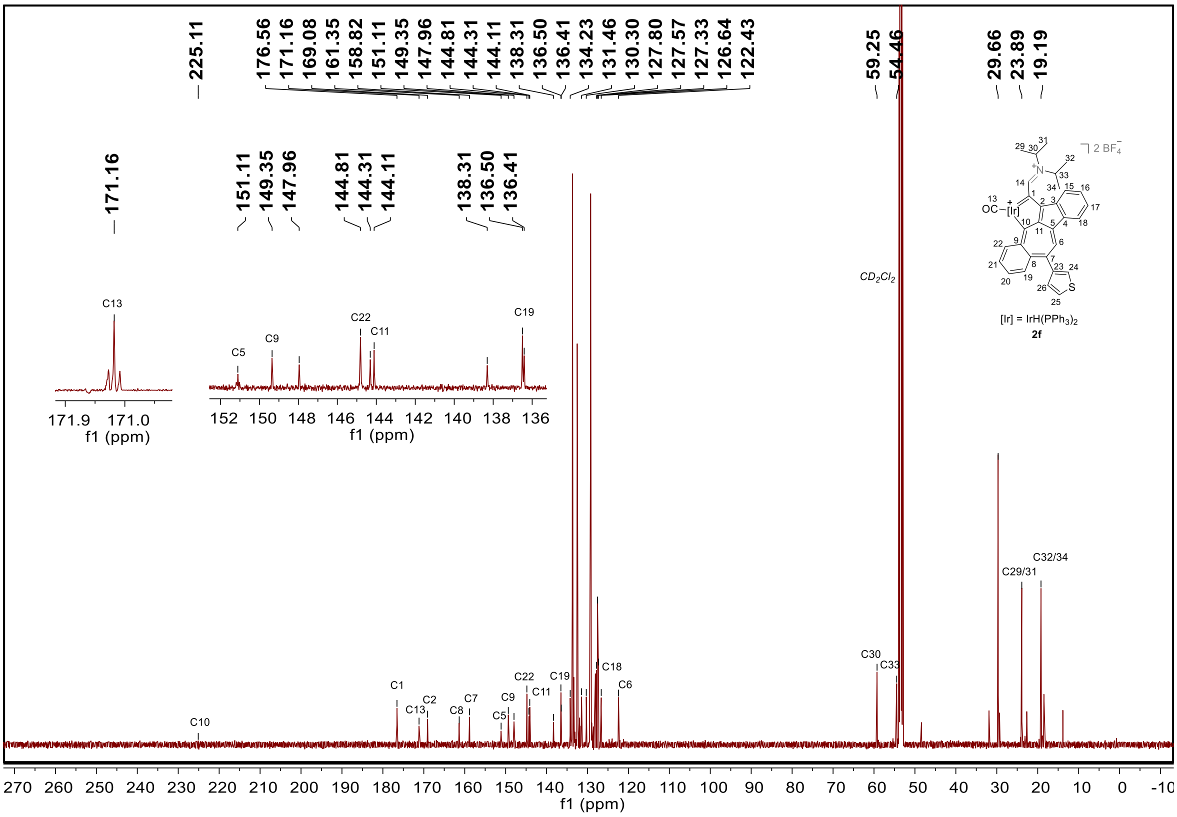

Supplementary Figure 66. The $^{13}C\{^{1}H\}$ NMR (125.8 MHz, CD_2Cl_2) spectrum for complex **2f.**

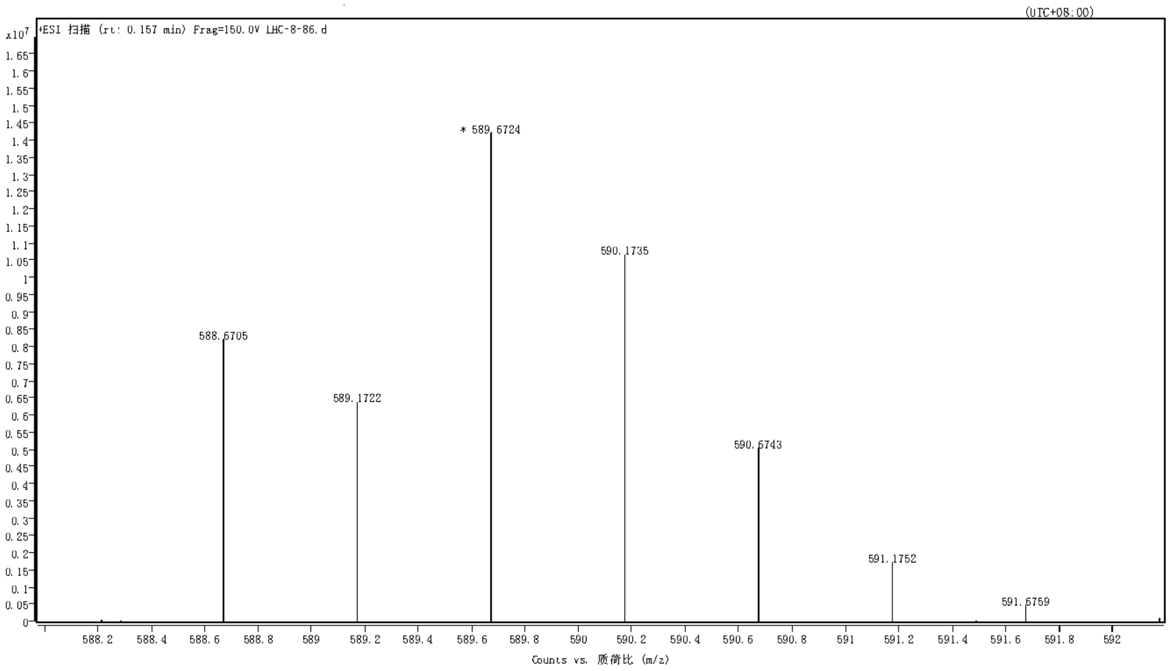

Supplementary Figure 67. Positive-ion ESI-MS spectrum for complex [**2f**]$^{2+}$ measured in methanol.

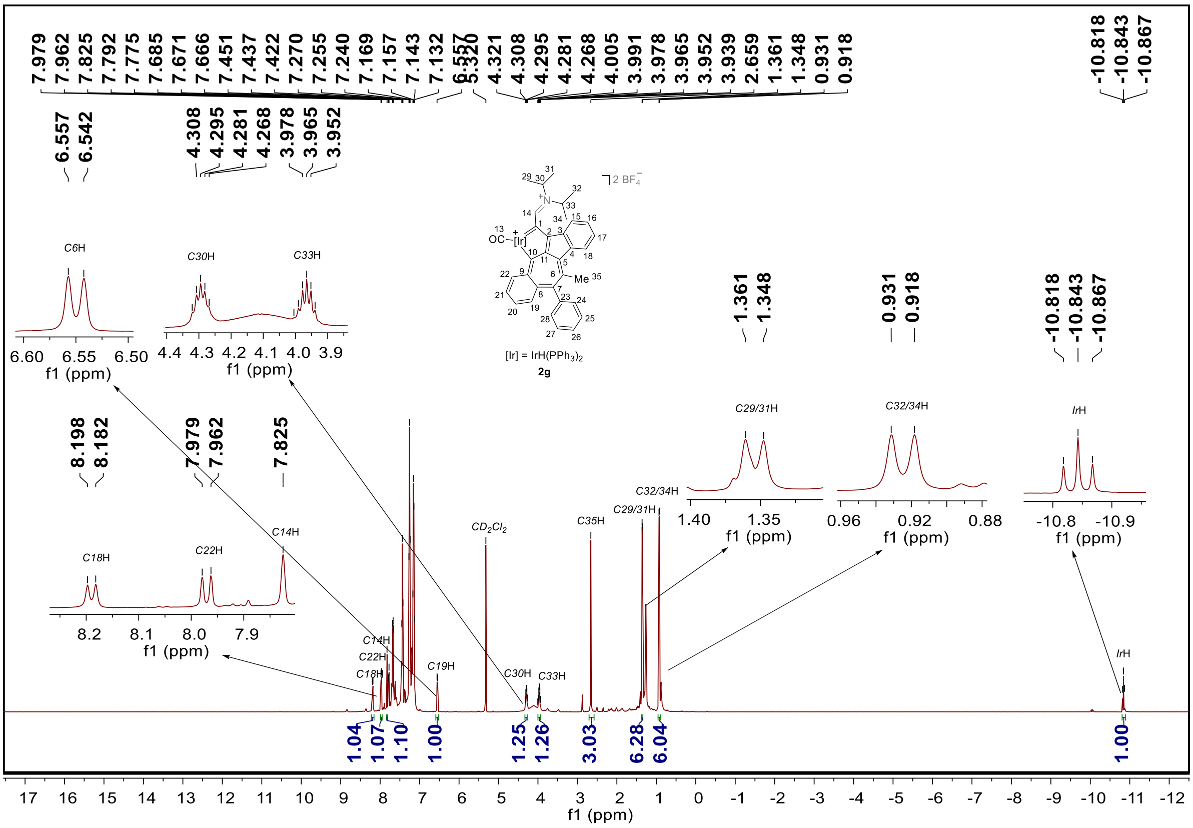

Supplementary Figure 68. The ^{1}H NMR (500.2 MHz, CD_2Cl_2) spectrum for complex **2g.**

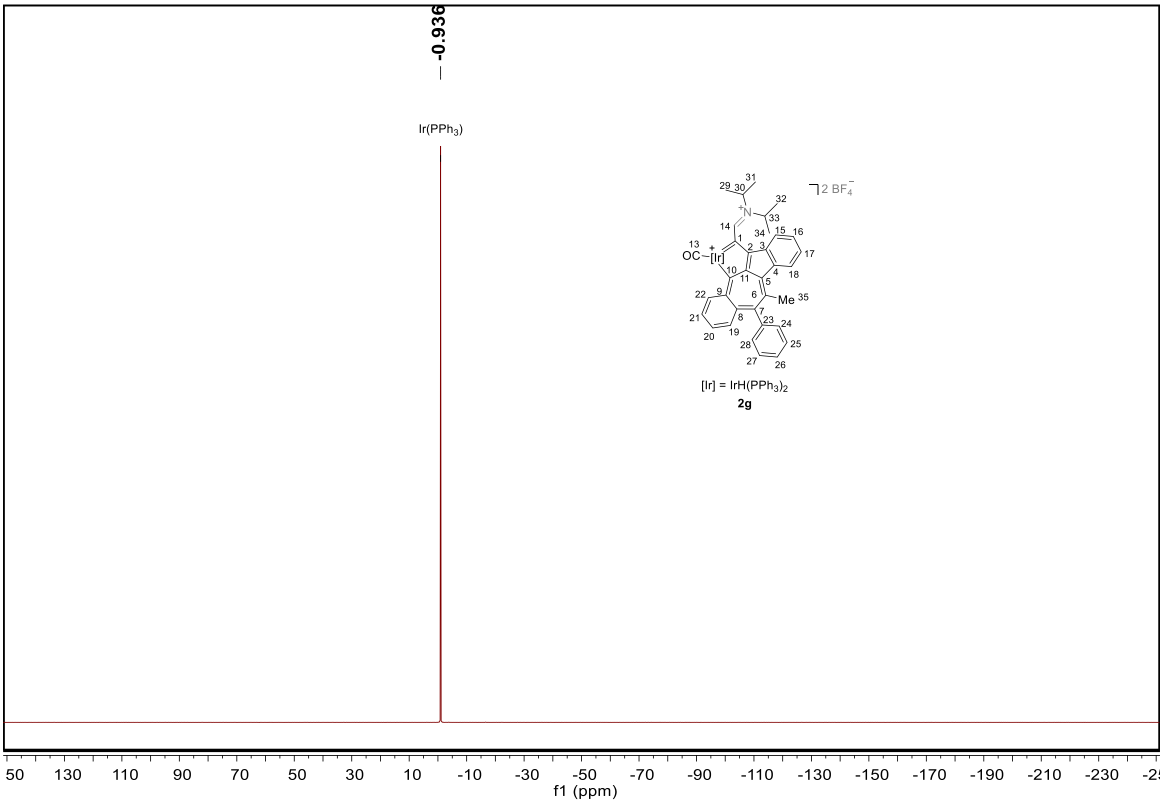

Supplementary Figure 69. The $^{31}P\{^1H\}$ NMR spectrum (202.5 MHz, CD_2Cl_2) for complex **2g.**

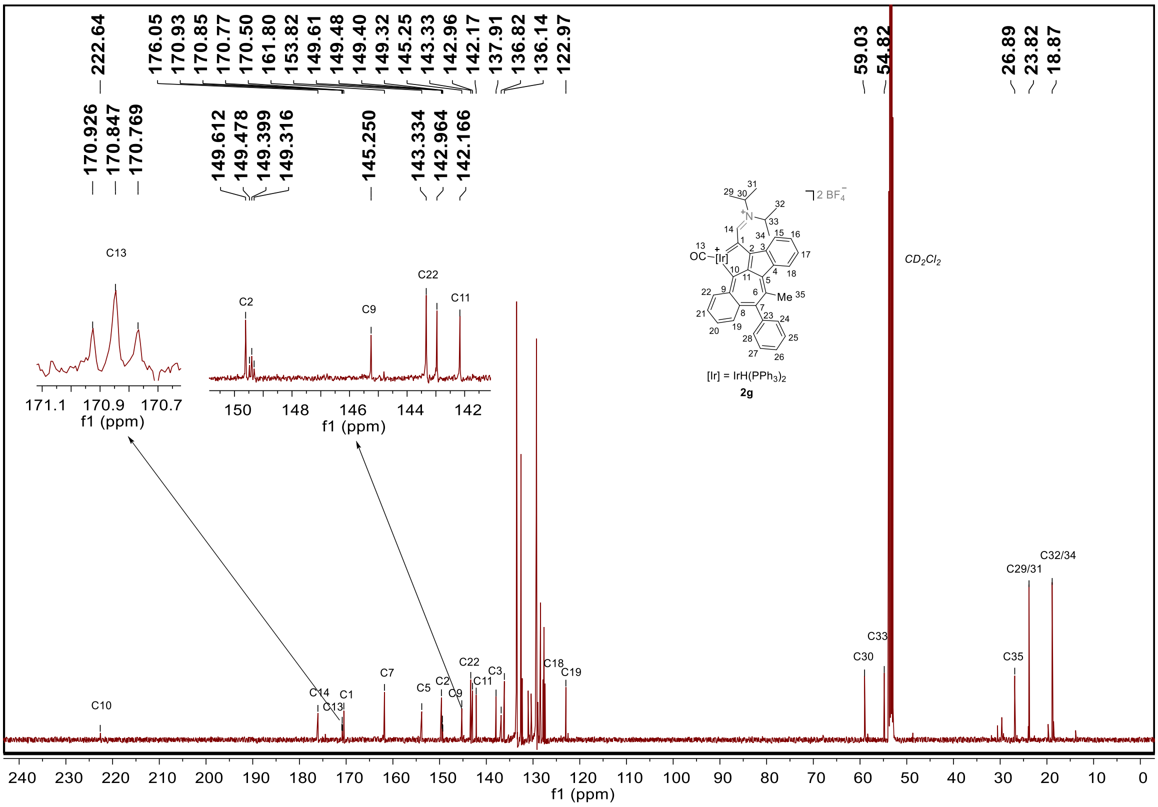

Supplementary Figure 70. The $^{13}C\{^1H\}$ NMR (125.8 MHz, CD_2Cl_2) spectrum for complex **2g.**

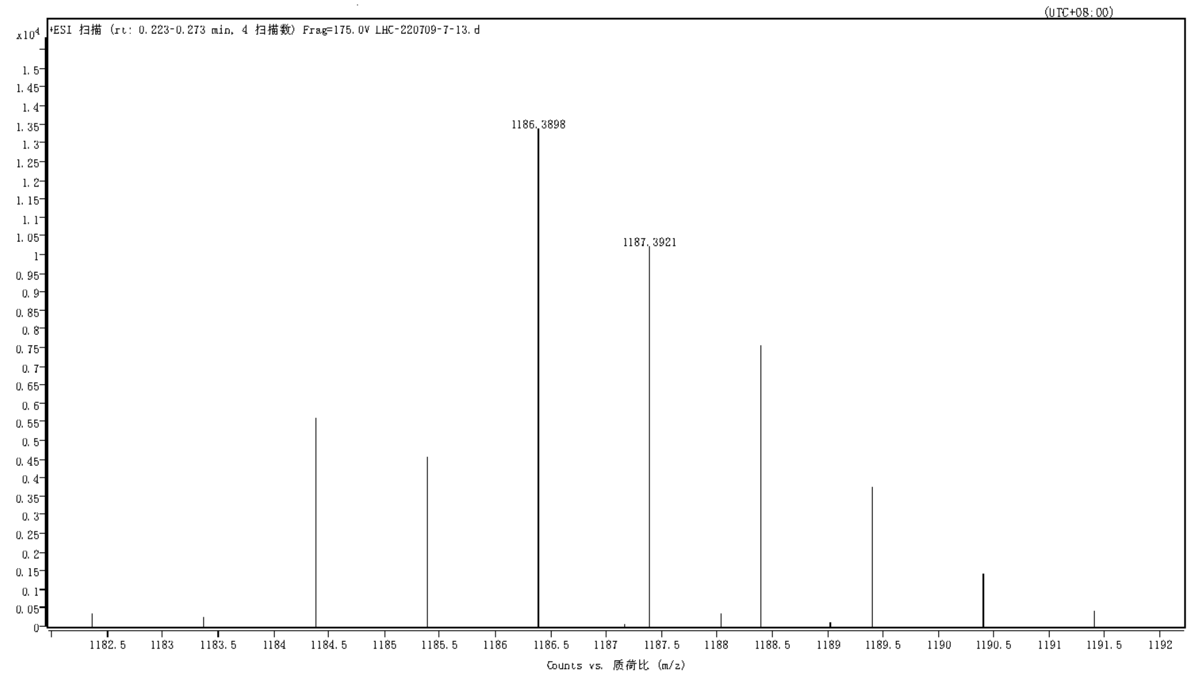

Supplementary Figure 71. Positive-ion ESI-MS spectrum for complex [**2g**-H^+]$^+$ measured in methanol.

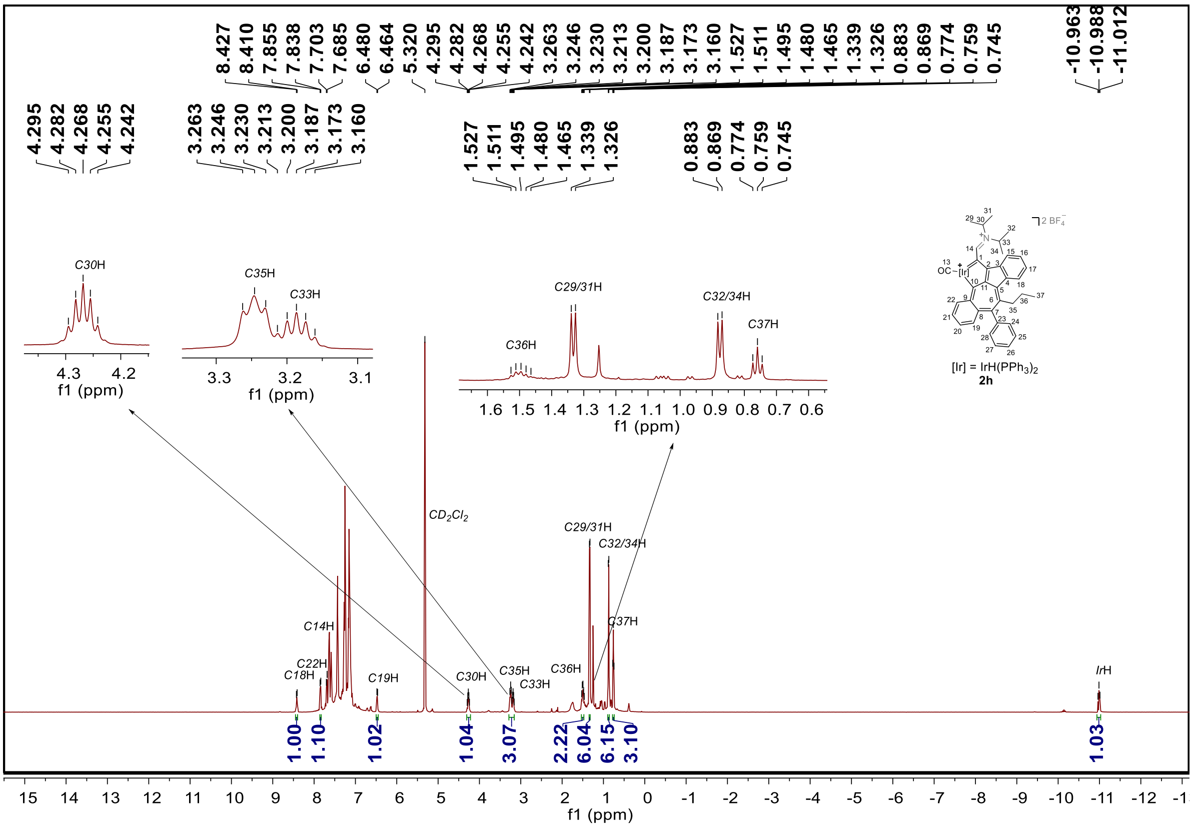

Supplementary Figure 72. The ^{1}H NMR (500.2 MHz, CD_2Cl_2) spectrum for complex **2h.**

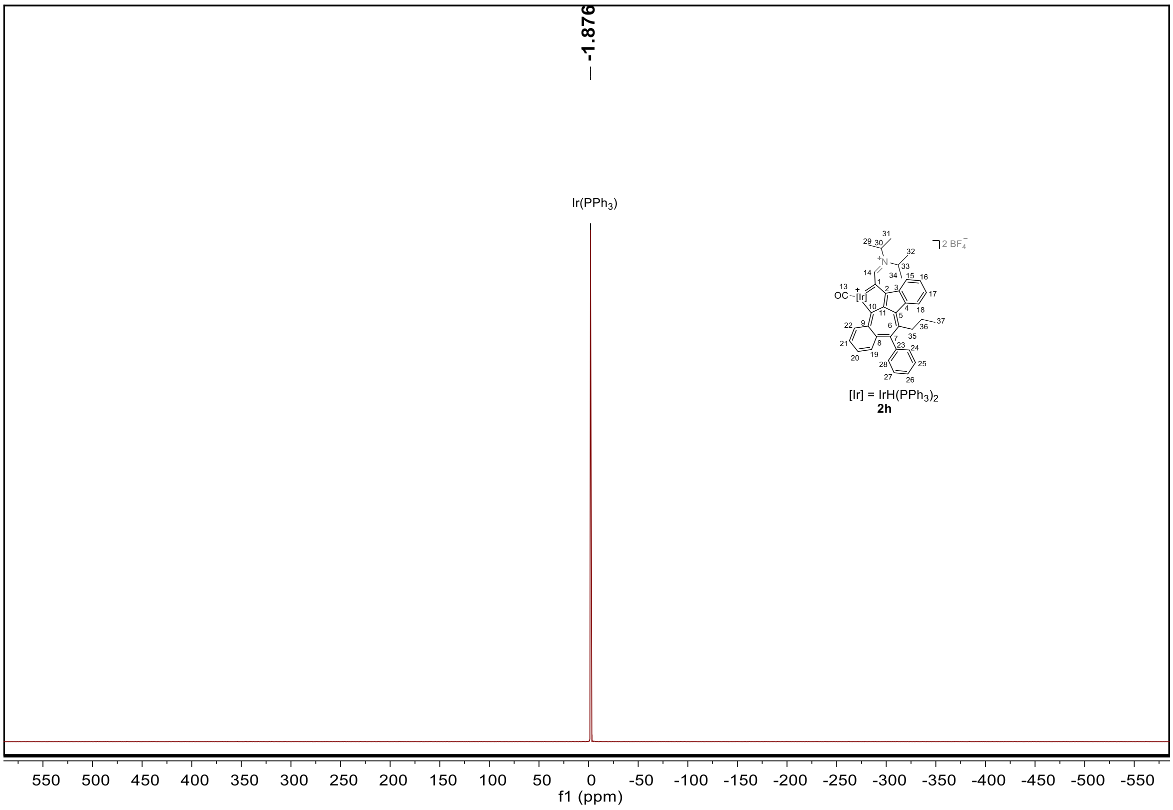

Supplementary Figure 73. The $^{31}P\{^{1}H\}$ NMR spectrum (202.5 MHz, CD_2Cl_2) for complex **2h.**

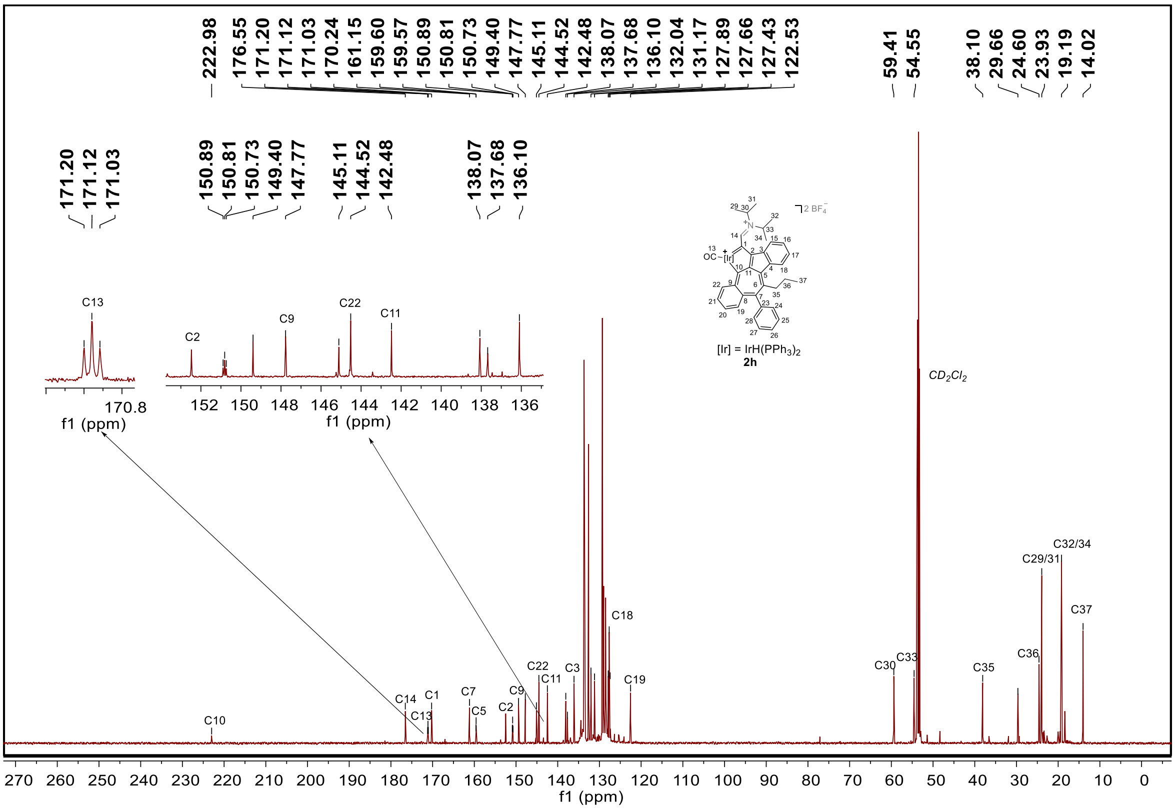

Supplementary Figure 74. The $^{13}C\{^{1}H\}$ NMR (125.8 MHz, CD_2Cl_2) spectrum for complex **2h.**

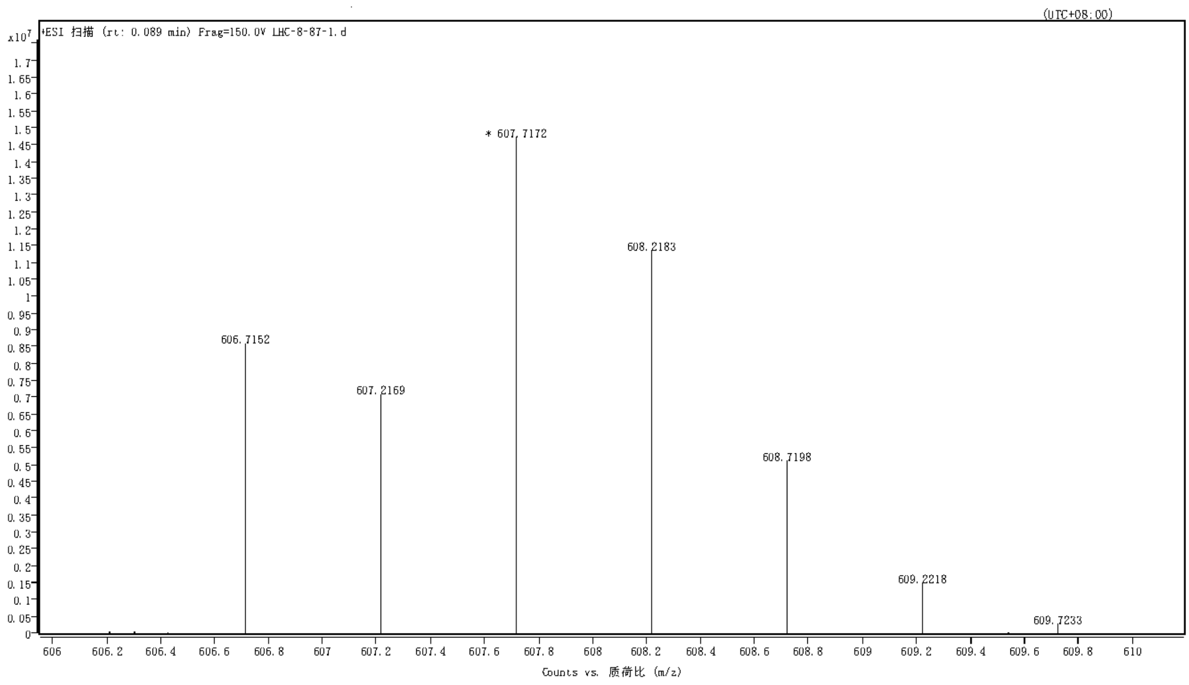

Supplementary Figure 75. Positive-ion ESI-MS spectrum for complex [**2h**]$^{2+}$ measured in methanol.

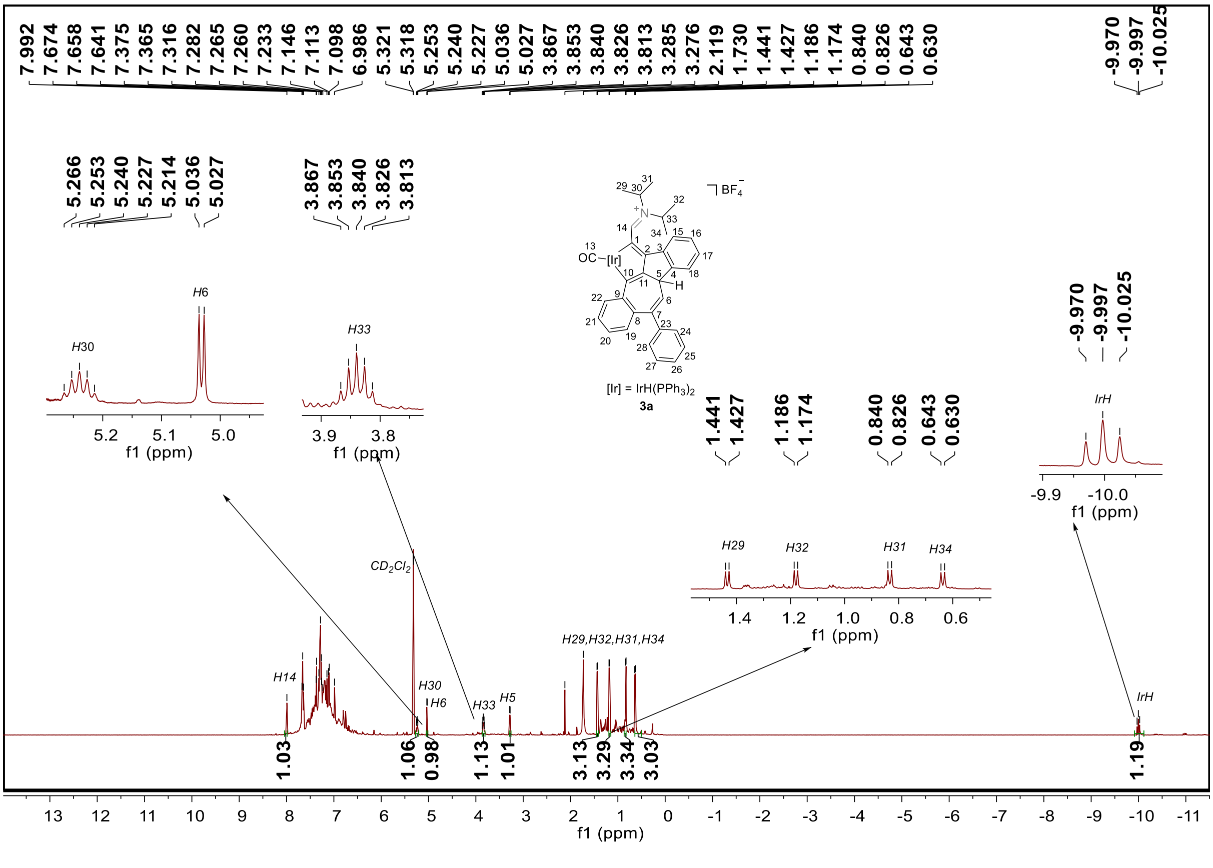

Supplementary Figure 76. The ^{1}H NMR (500.2 MHz, CD_2Cl_2) spectrum for complex **3a.**

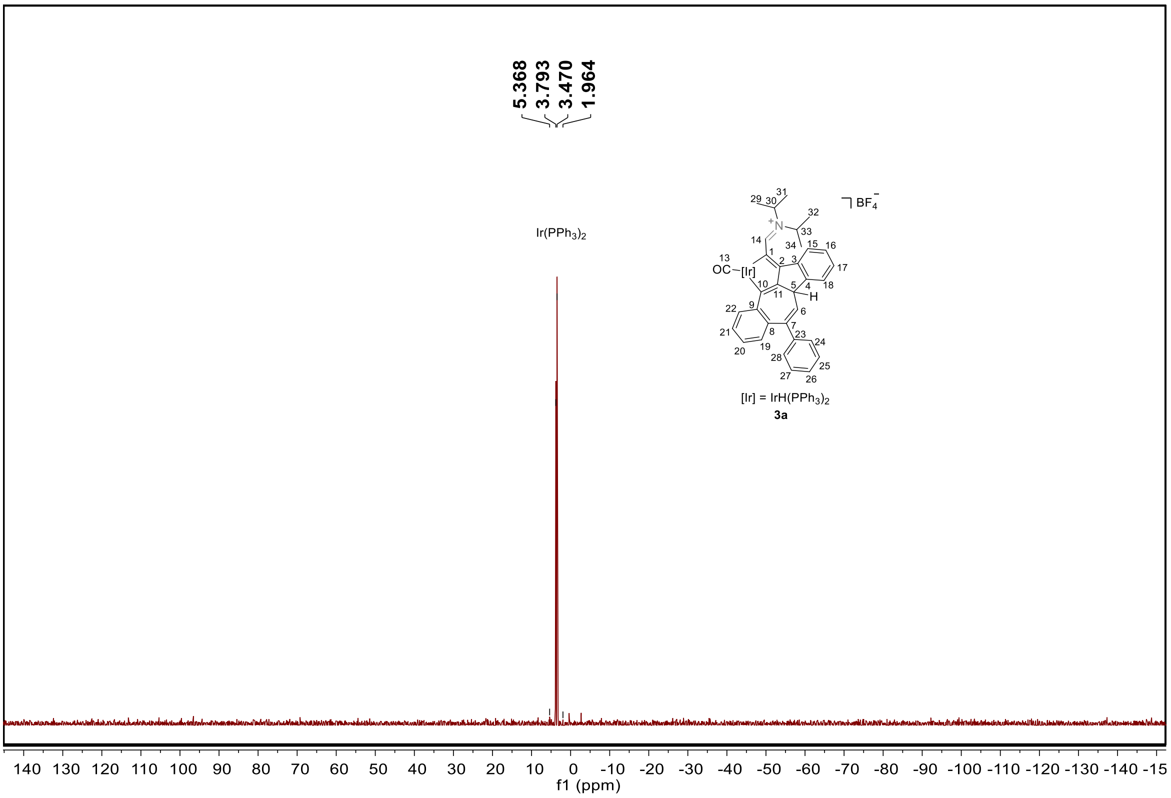

Supplementary Figure 77. The $^{31}P\{^{1}H\}$ NMR spectrum (202.5 MHz, CD_2Cl_2) for complex **3a.**

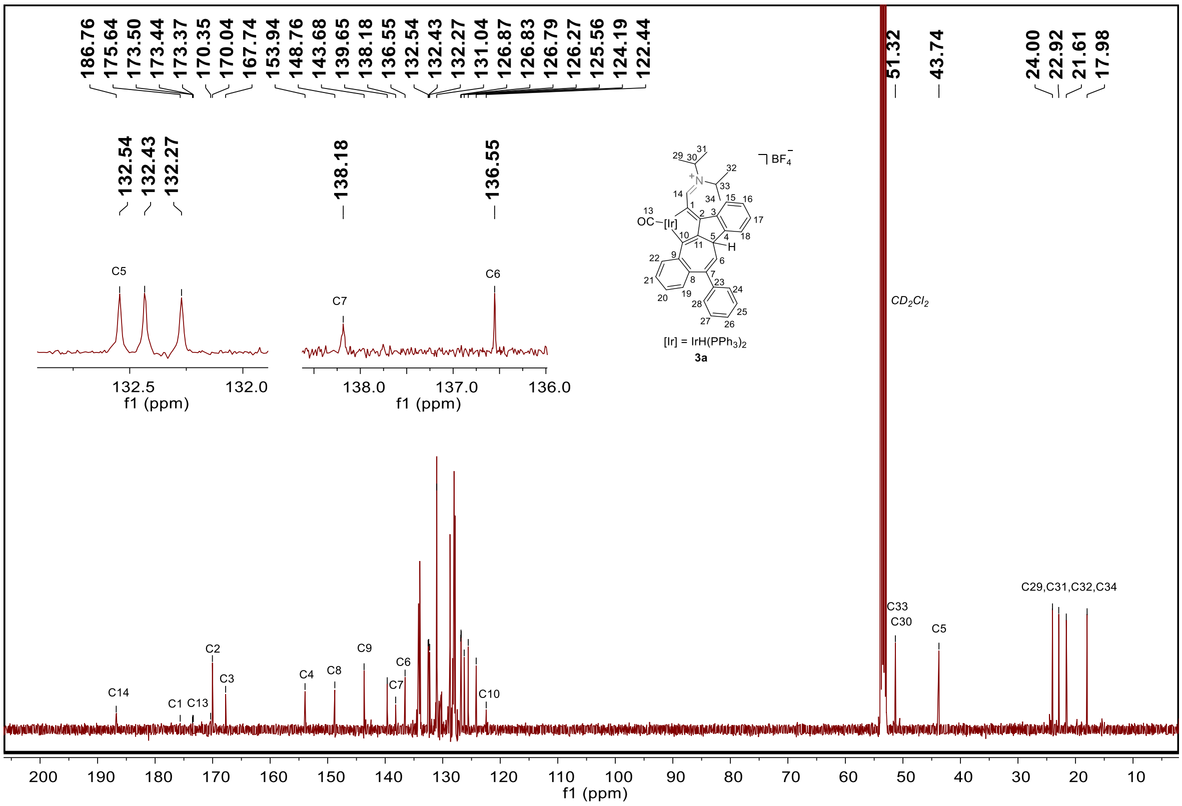

Supplementary Figure 78. The $^{13}C\{^{1}H\}$ NMR (125.8 MHz, CD_2Cl_2) spectrum for complex **3a.**

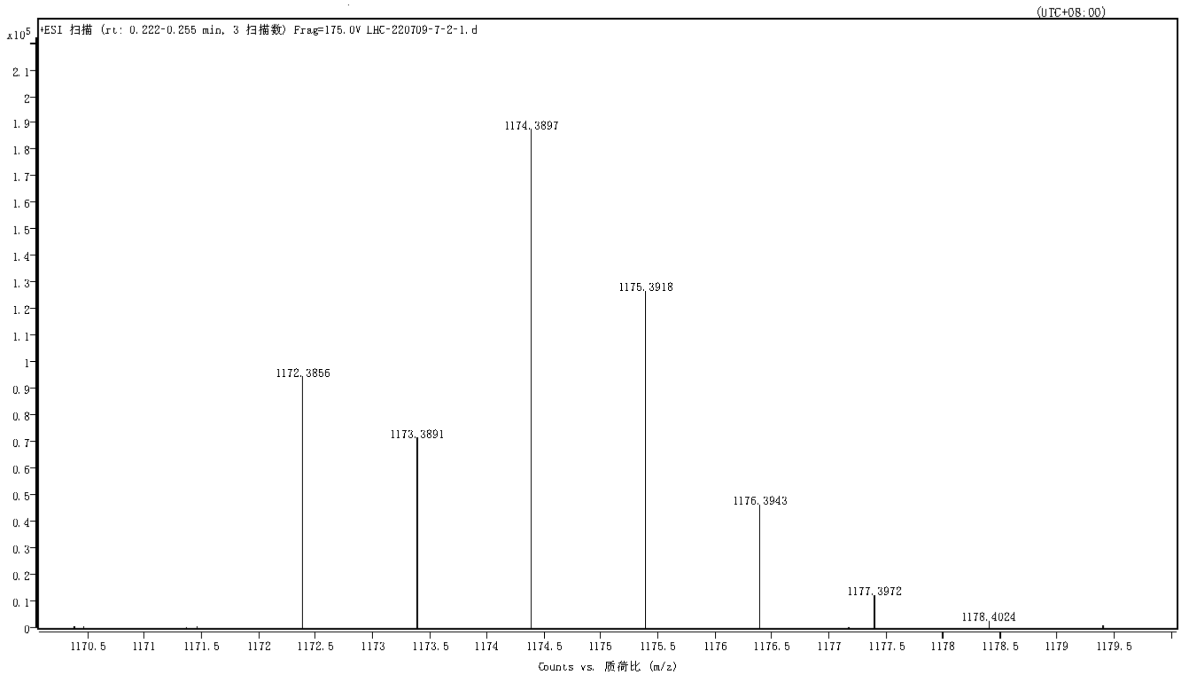

Supplementary Figure 79. Positive-ion ESI-MS spectrum for complex [**3a**]$^+$ measured in methanol.

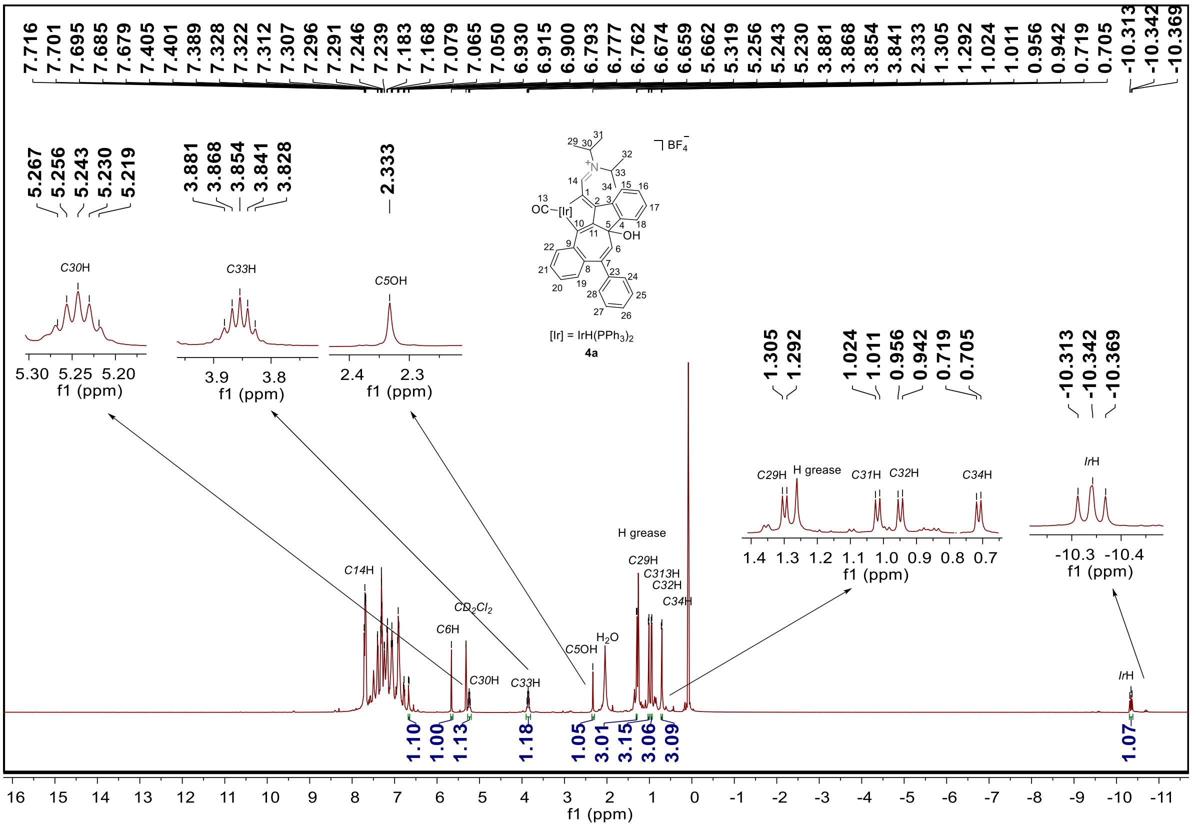

Supplementary Figure 80. The ^1H NMR (500.2 MHz, CD_2Cl_2) spectrum for complex **4a**.

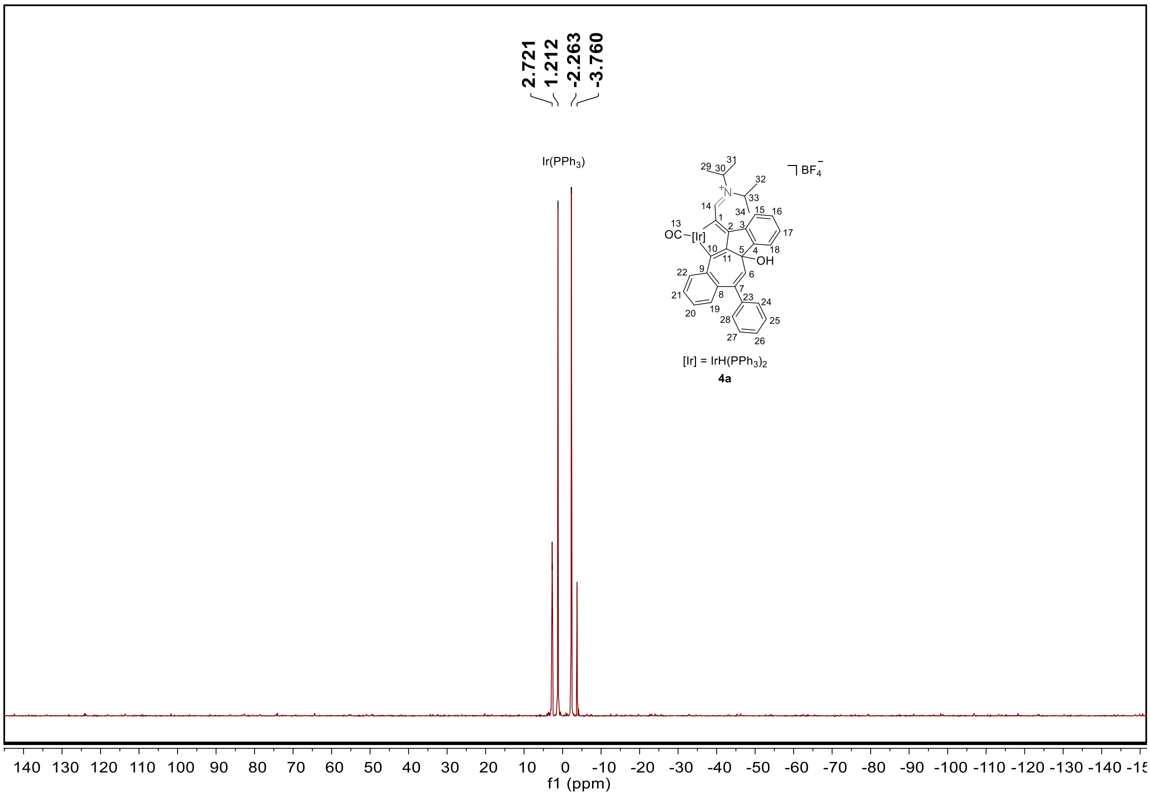

Supplementary Figure 81. The $^{31}P\{^{1}H\}$ NMR spectrum (202.5 MHz, CD_2Cl_2) for complex **4a.**

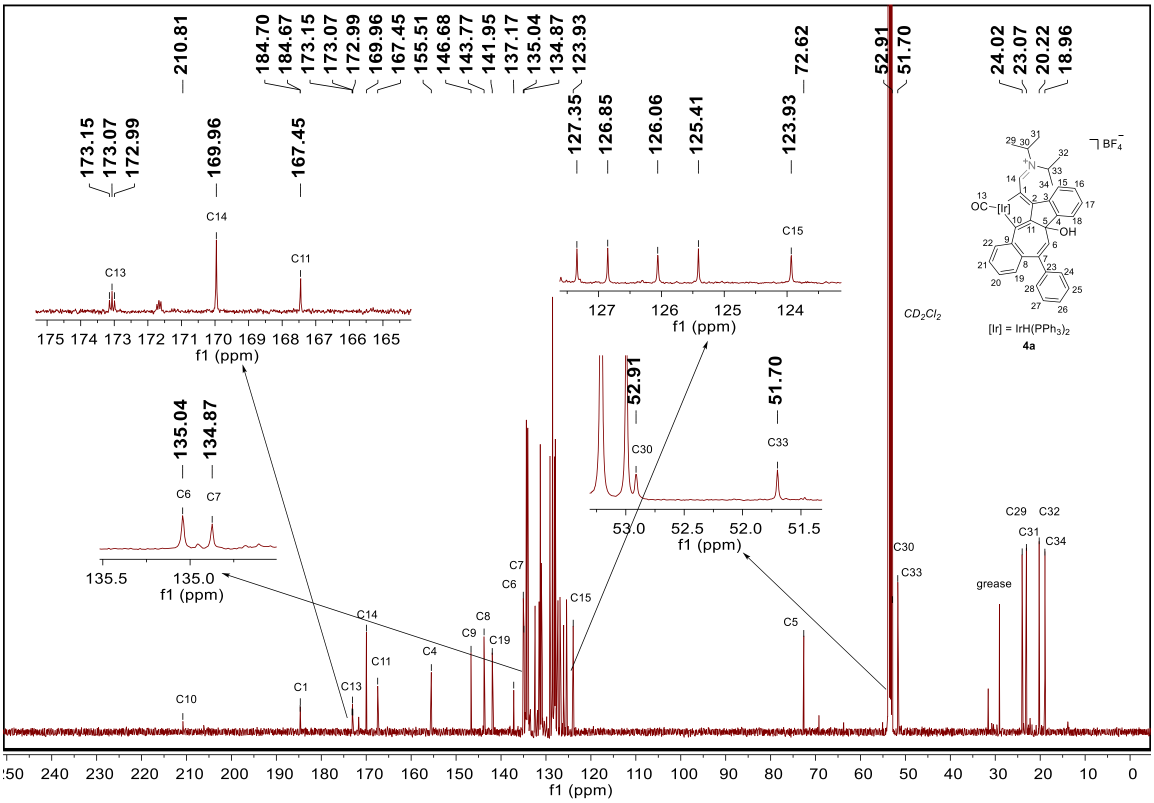

Supplementary Figure 82. The $^{13}C\{^{1}H\}$ NMR (125.8 MHz, CD_2Cl_2) spectrum for complex **4a.**

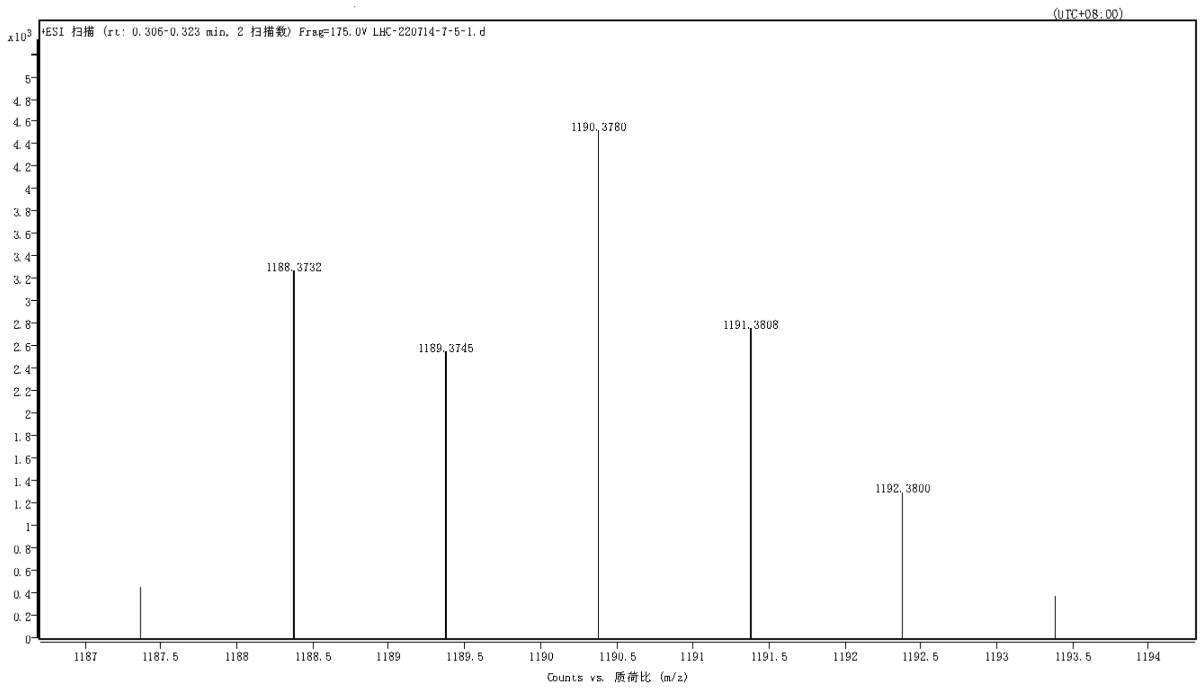

Supplementary Figure 83. Positive-ion ESI-MS spectrum for complex [**4a**]$^+$ measured in methanol

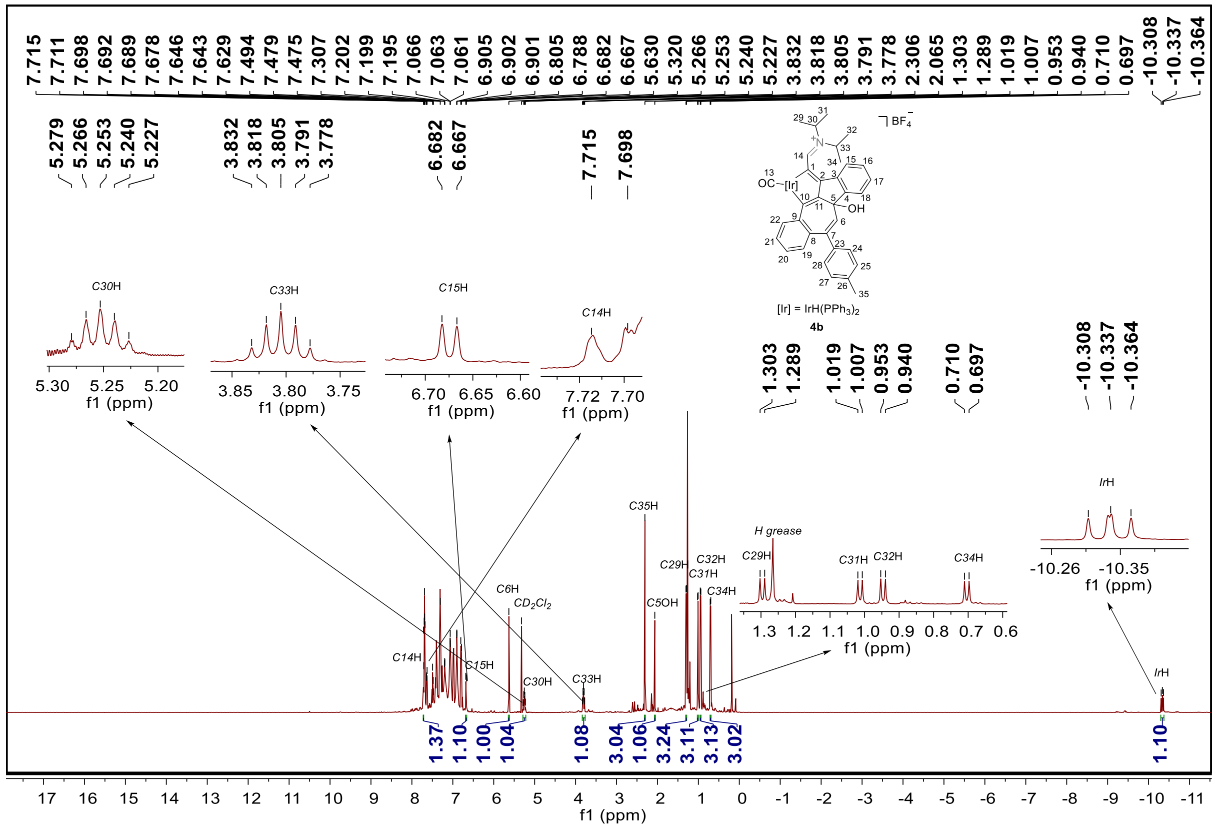

Supplementary Figure 84. The ^{1}H NMR (500.2 MHz, CD_2Cl_2) spectrum for complex **4b.**

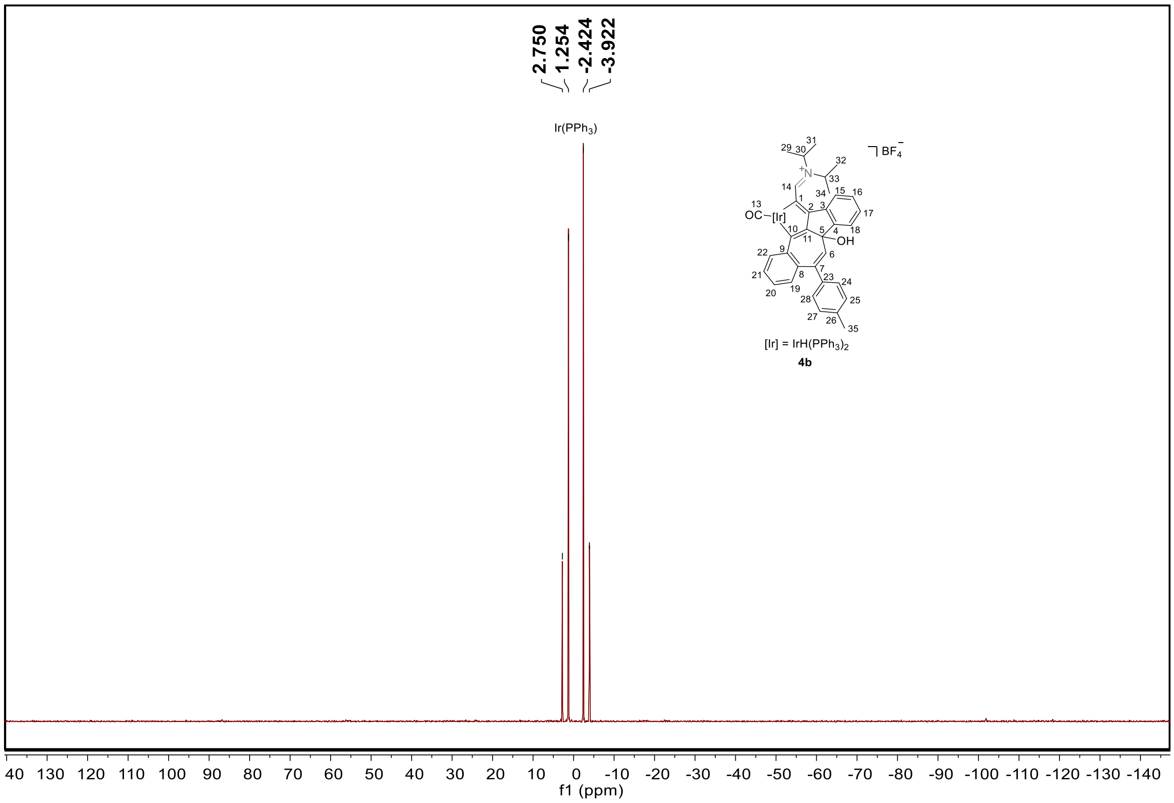

Supplementary Figure 85. The $^{31}P\{^1H\}$ NMR spectrum (202.5 MHz, CD_2Cl_2) for complex **4b.**

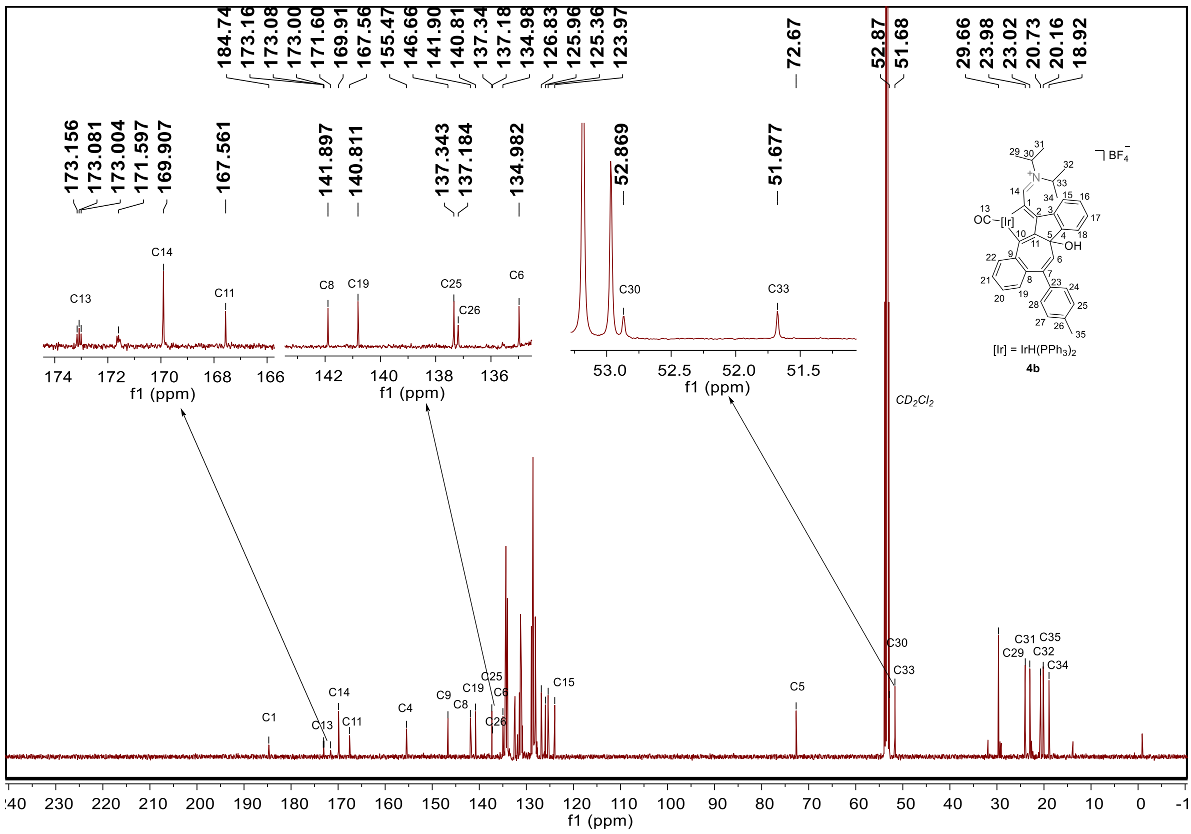

Supplementary Figure 86. The $^{13}C\{^1H\}$ NMR (125.8 MHz, CD_2Cl_2) spectrum for complex **4b.**

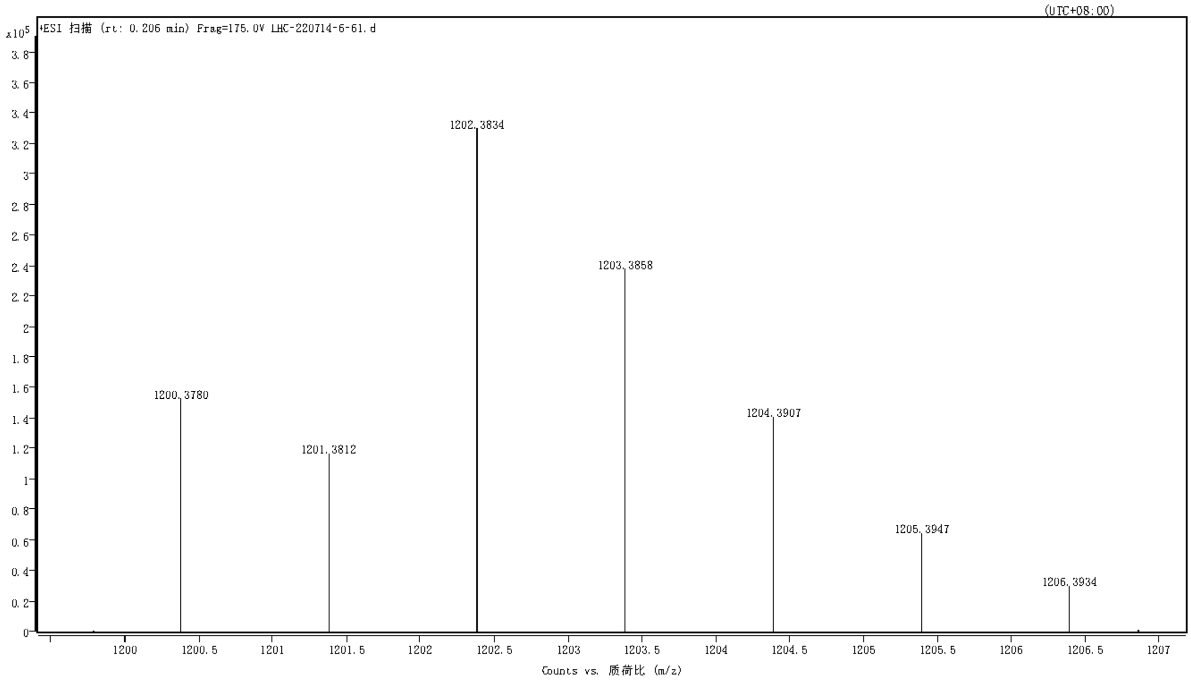

Supplementary Figure 87. Positive-ion ESI-MS spectrum for complex $[\mathbf{4b}]^+$ measured in methanol.

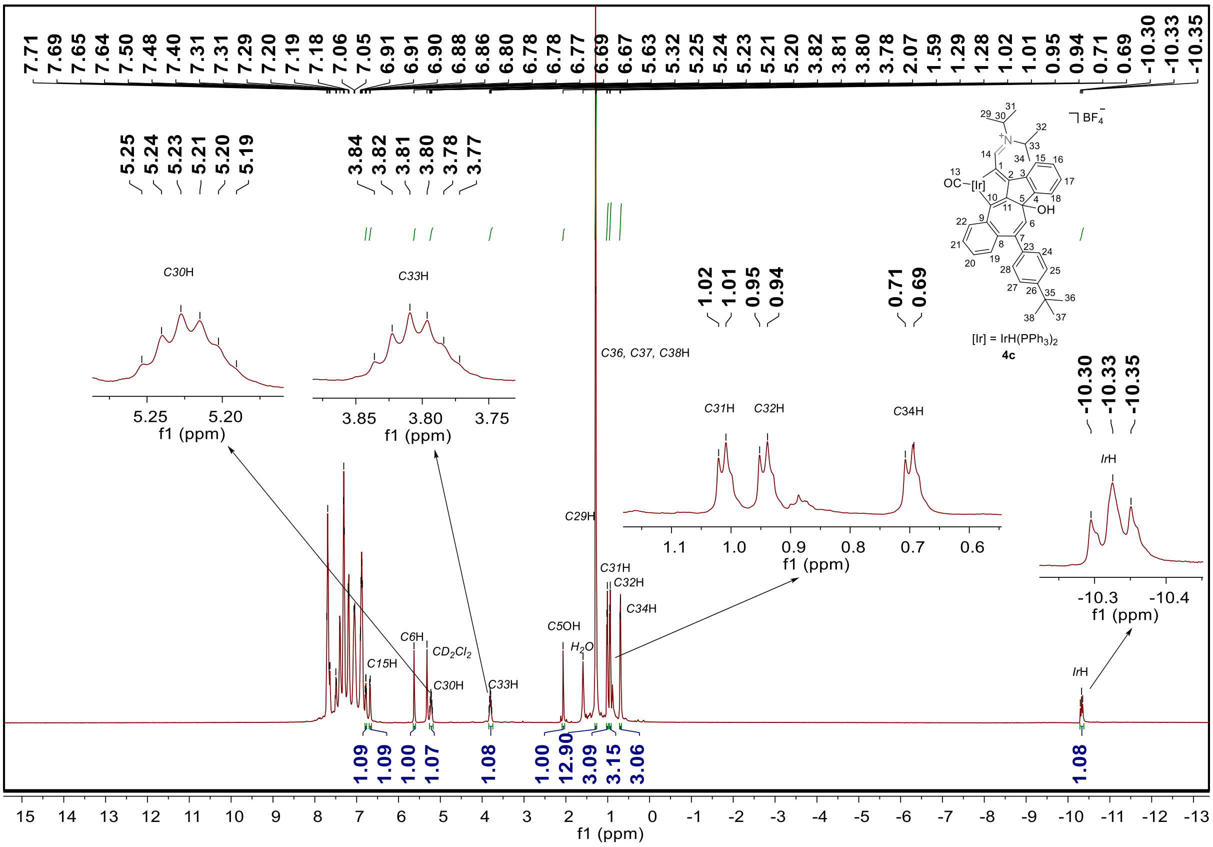

Supplementary Figure 88. The ^{1}H NMR (500.2 MHz, CD_2Cl_2) spectrum for complex **4c.**

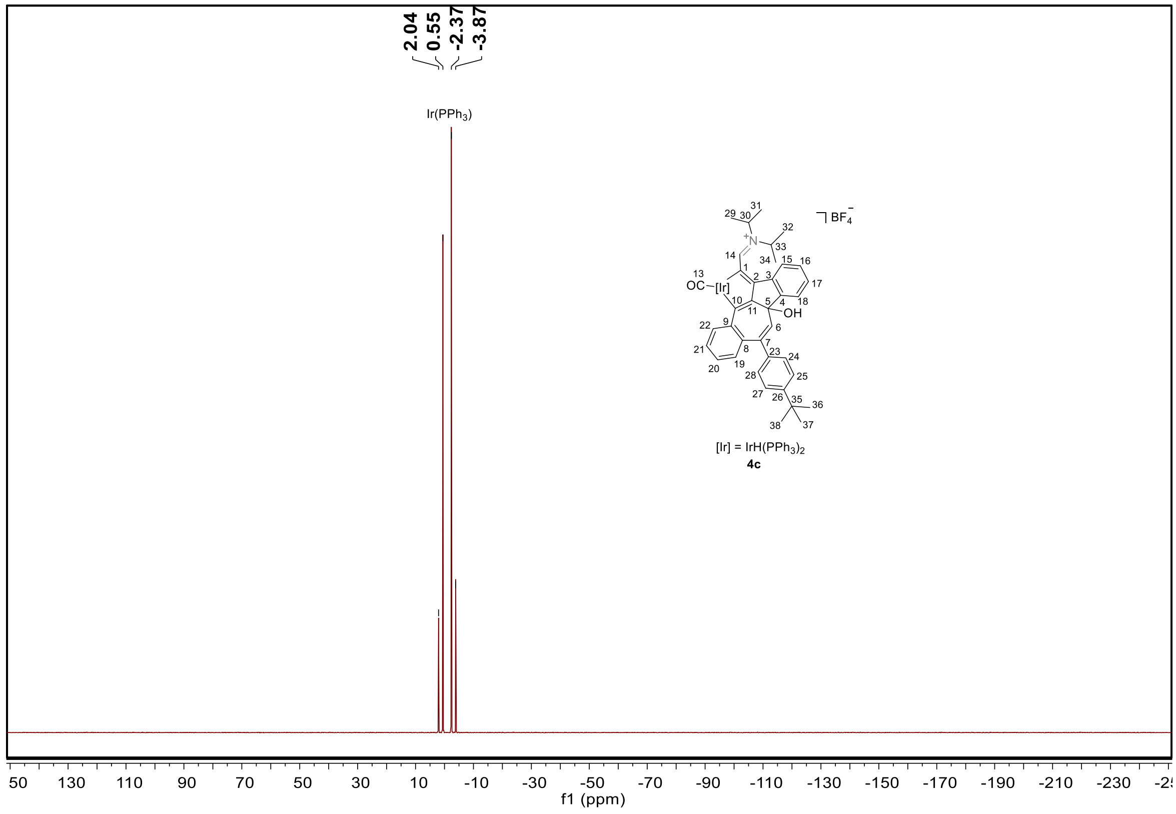

Supplementary Figure 89. The $^{31}P\{^{1}H\}$ NMR spectrum (202.5 MHz, CD_2Cl_2) for complex **4c.**

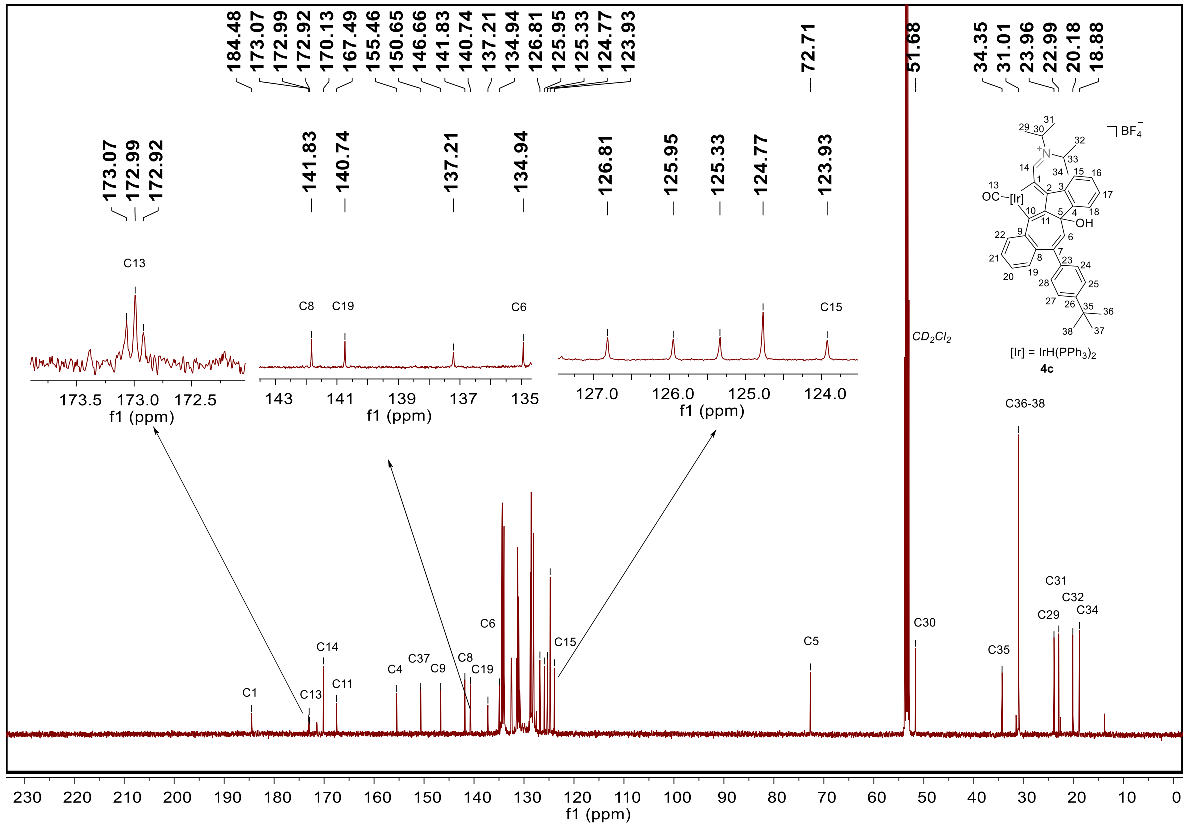

Supplementary Figure 90. The $^{13}C\{^{1}H\}$ NMR (125.8 MHz, CD_2Cl_2) spectrum for complex **4c.**

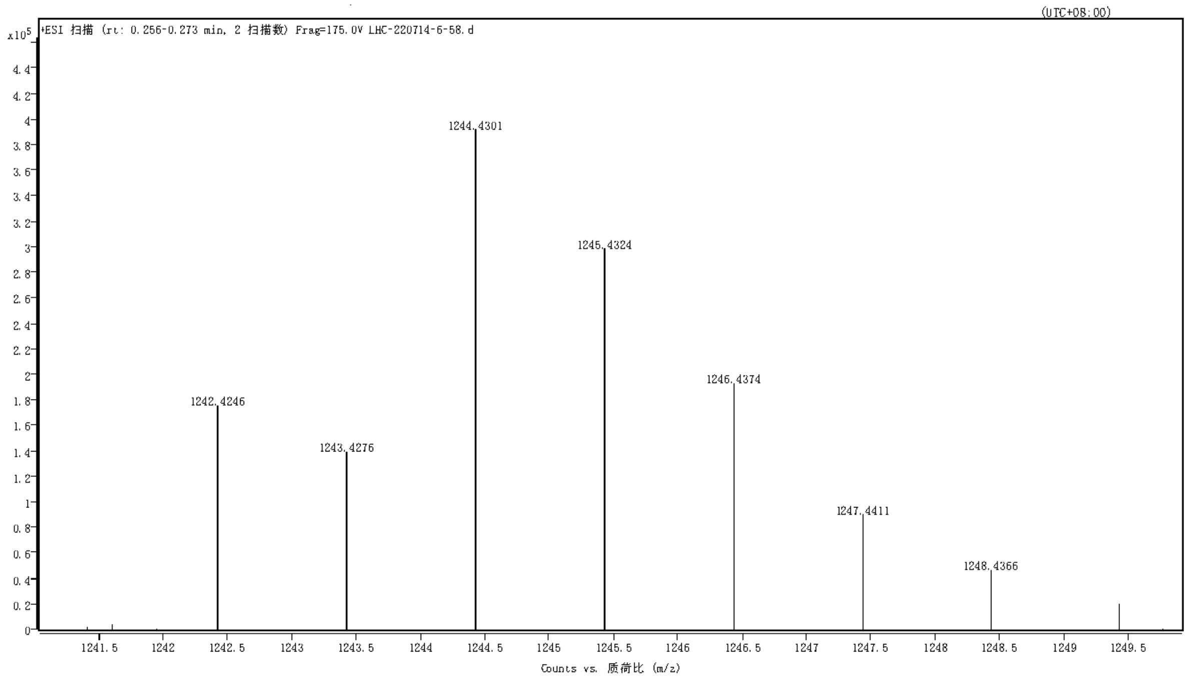

Supplementary Figure 91. Positive-ion ESI-MS spectrum for complex [**4c**]$^+$ measured in methanol

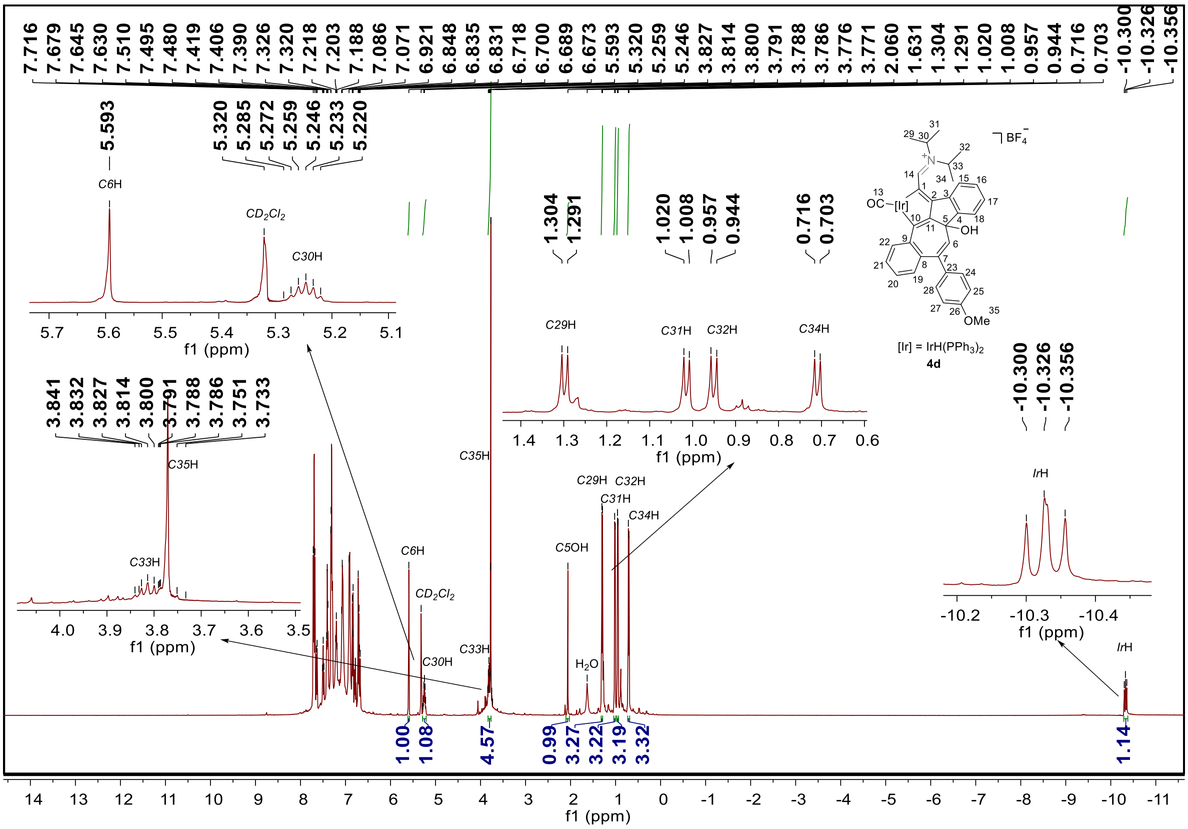

Supplementary Figure 92. The ^{1}H NMR (500.2 MHz, CD_2Cl_2) spectrum for complex **4d.**

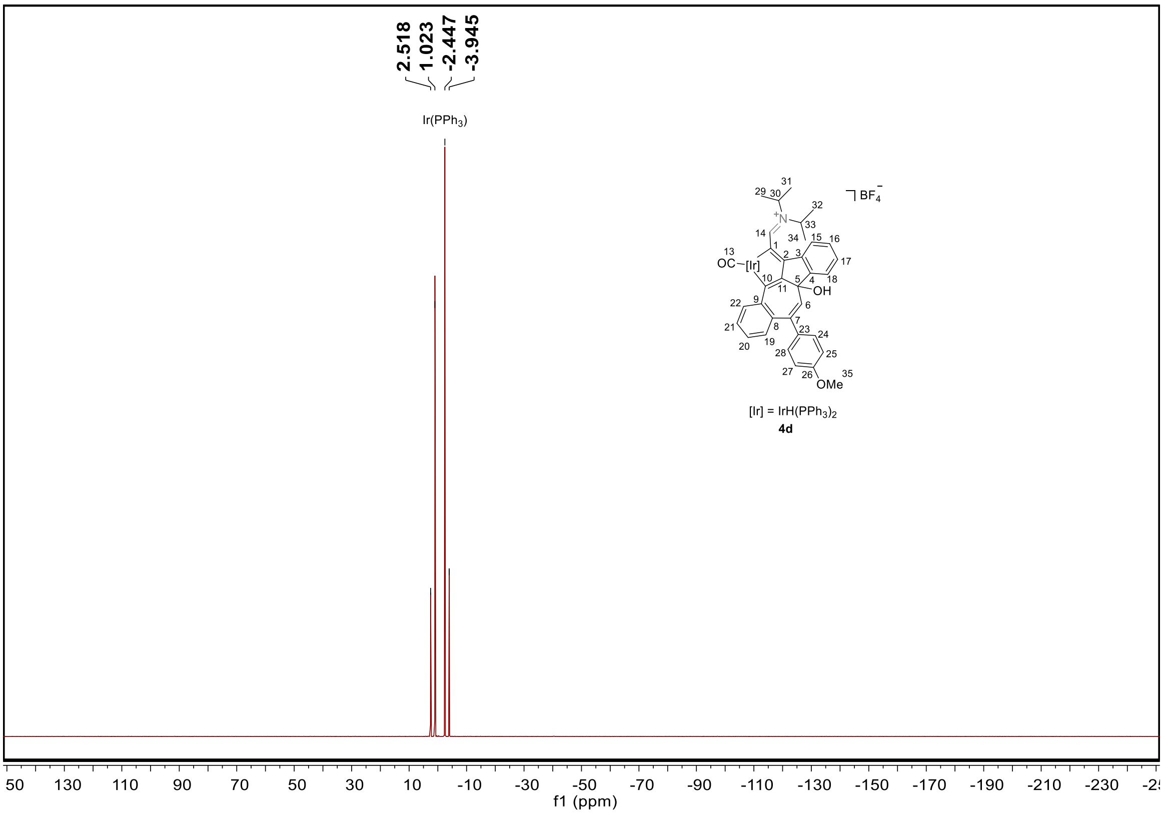

Supplementary Figure 93. The $^{31}P\{^{1}H\}$ NMR spectrum (202.5 MHz, CD_2Cl_2) for complex **4d.**

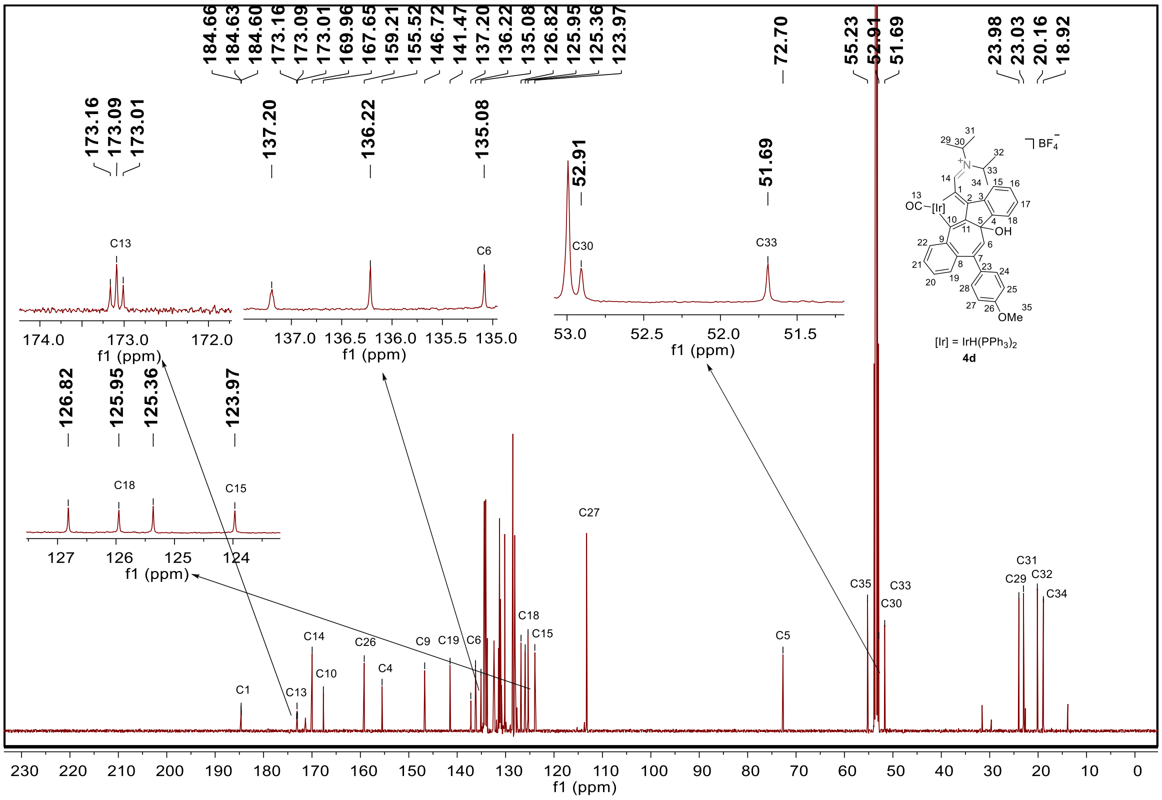

Supplementary Figure 94. The $^{13}C\{^{1}H\}$ NMR (125.8 MHz, CD_2Cl_2) spectrum for complex **4d.**

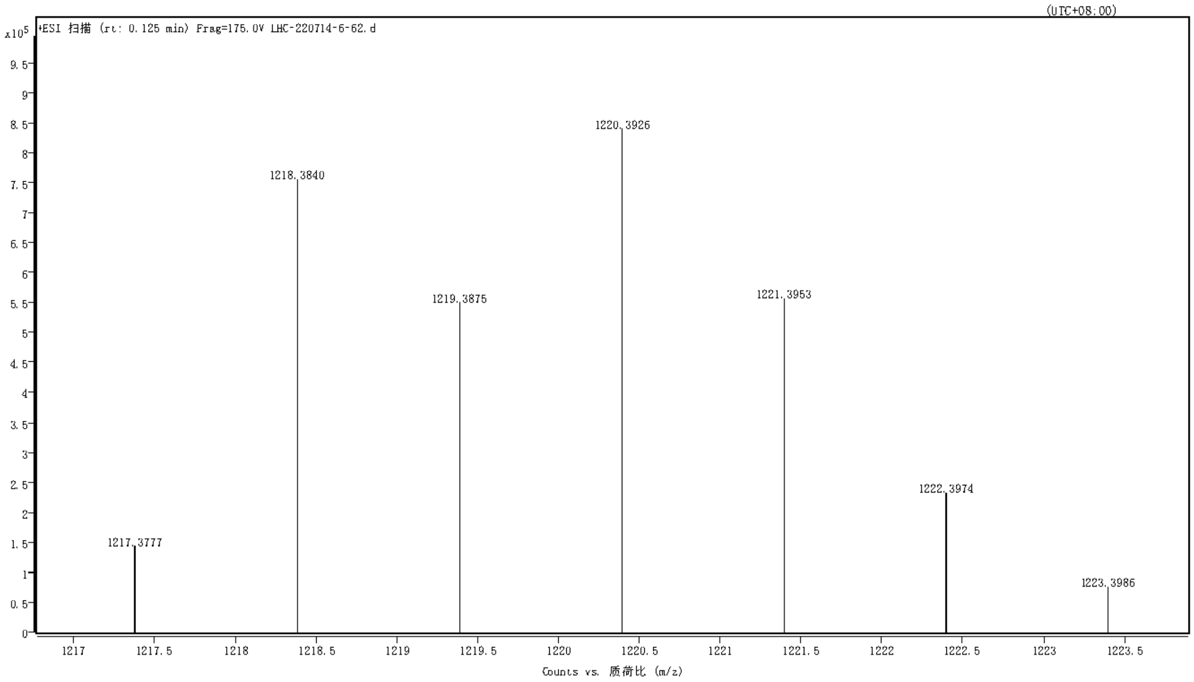

Supplementary Figure 95. Positive-ion ESI-MS spectrum for complex [**4d**]$^+$ measured in methanol

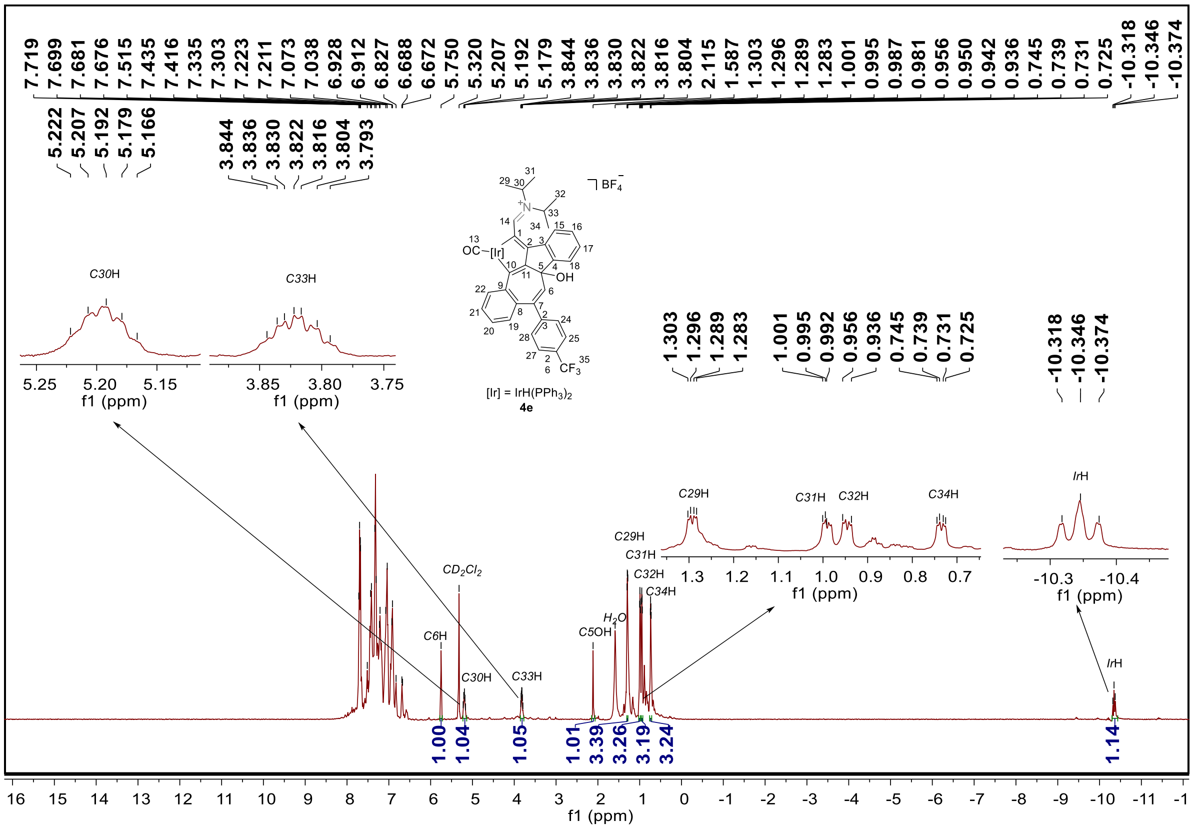

Supplementary Figure 96. The ^{1}H NMR (500.2 MHz, CD_2Cl_2) spectrum for complex **4e.**

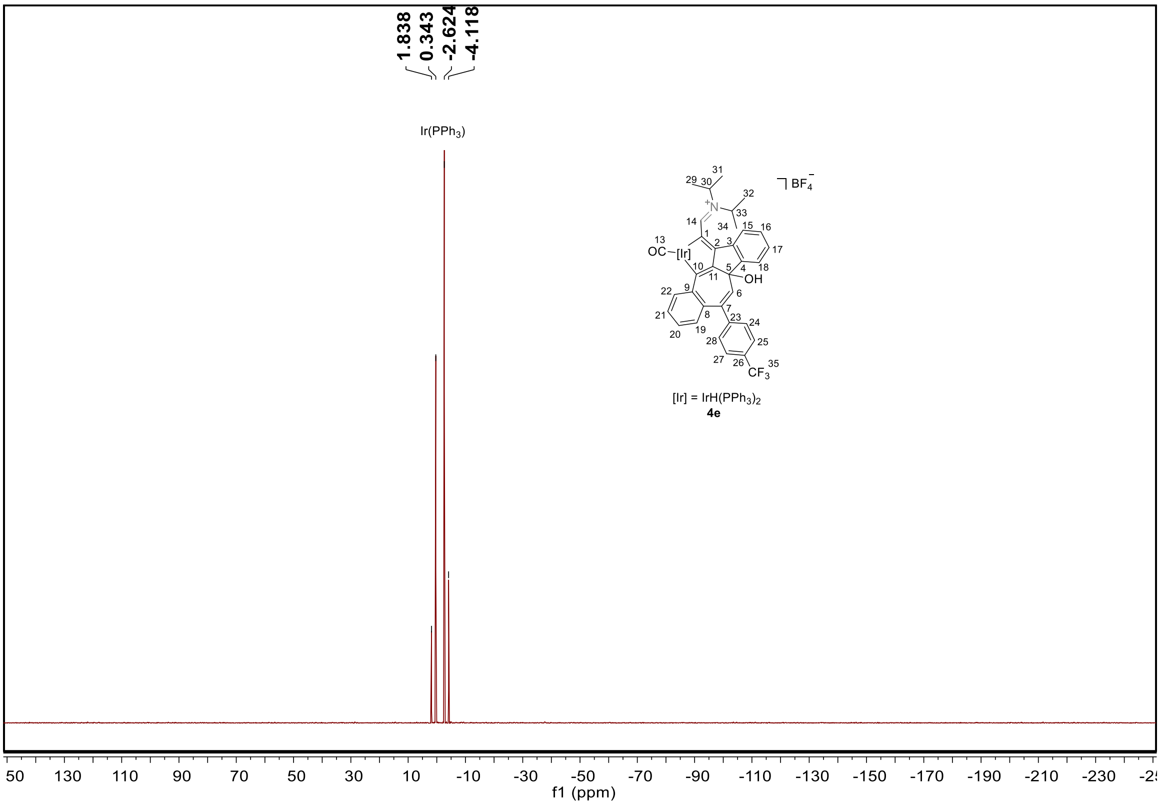

Supplementary Figure 97. The $^{31}P\{^1H\}$ NMR spectrum (202.5 MHz, CD_2Cl_2) for complex **4e.**

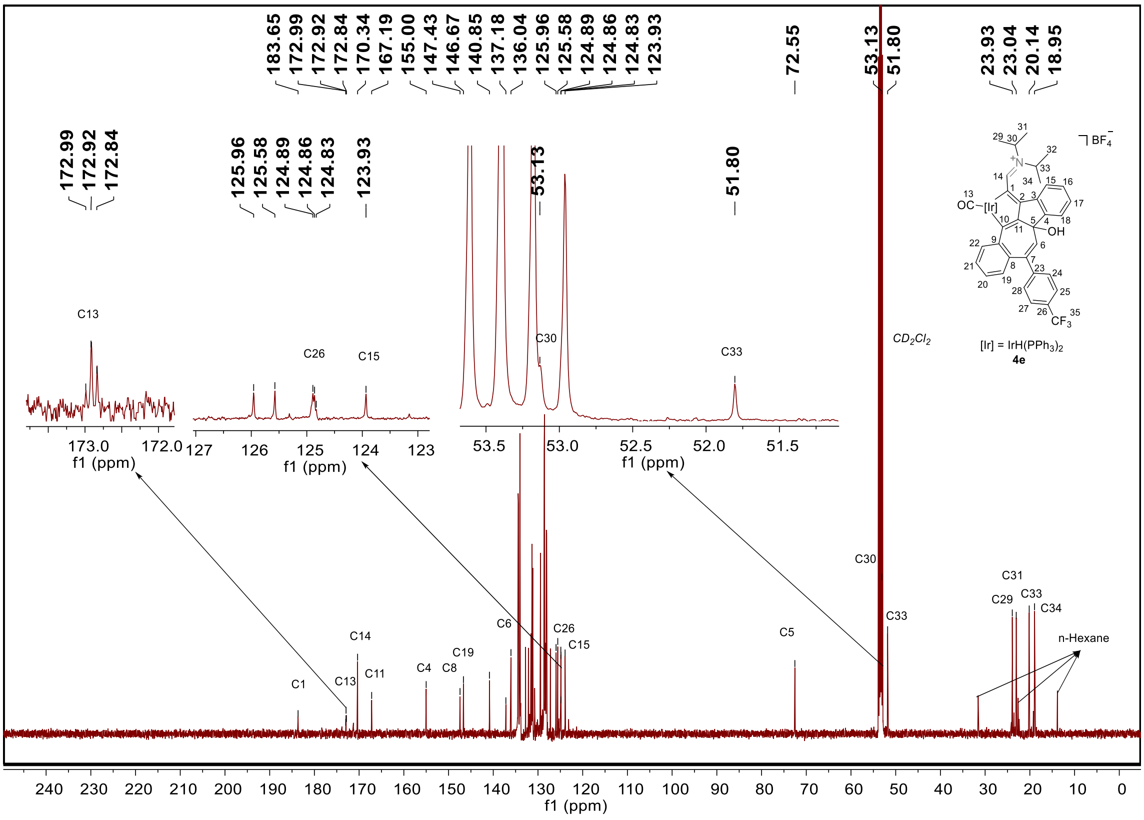

Supplementary Figure 98. The $^{13}C\{^1H\}$ NMR (125.8 MHz, CD_2Cl_2) spectrum for complex **4e.**

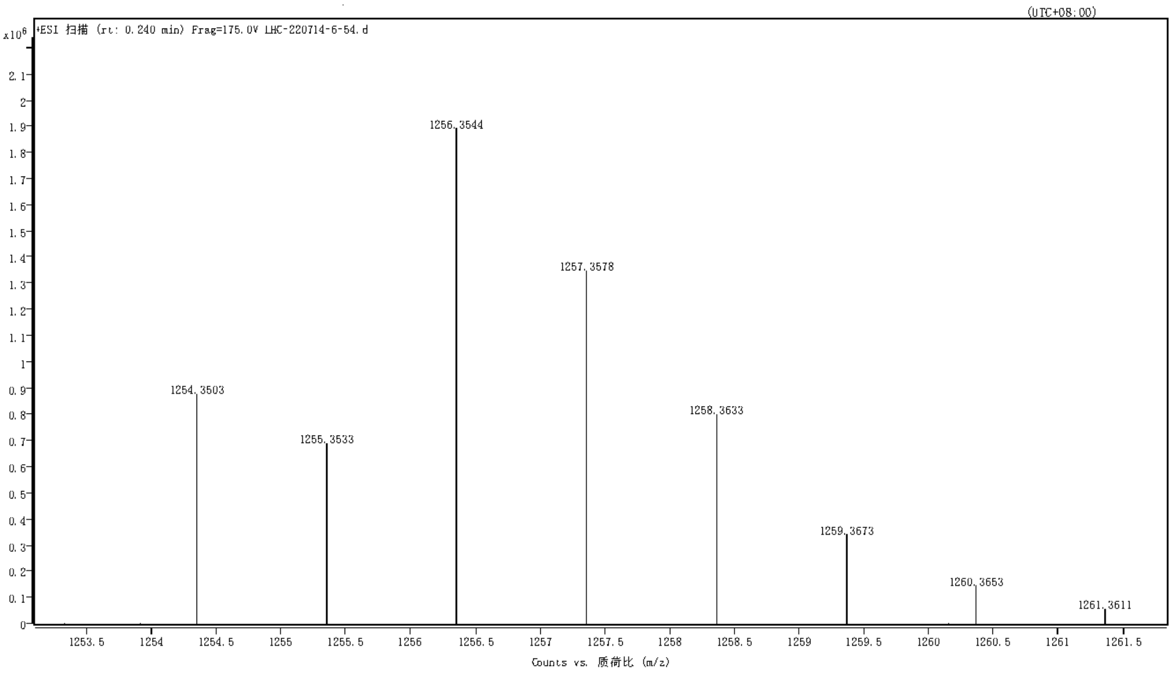

Supplementary Figure 99. Positive-ion ESI-MS spectrum for complex [**4e**]$^+$ measured in methanol

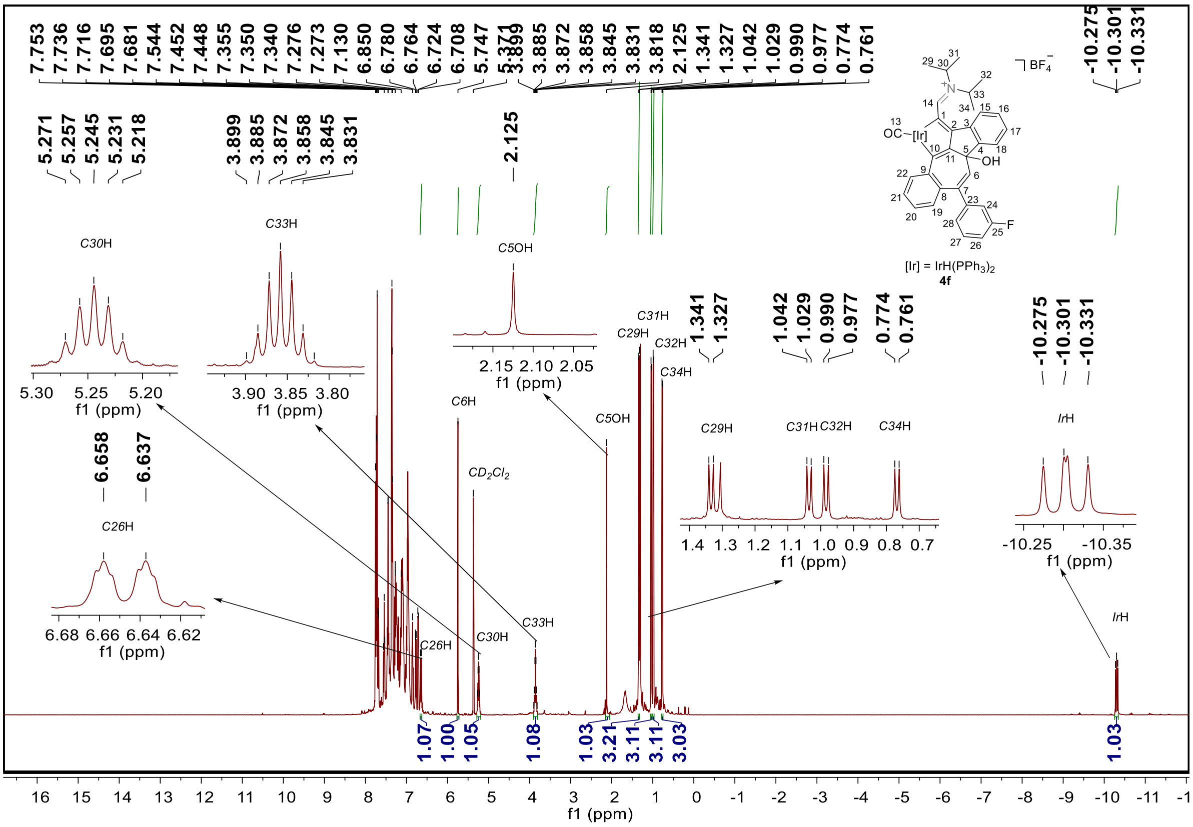

Supplementary Figure 100. The ^1H NMR (500.2 MHz, CD_2Cl_2) spectrum for complex **4f.**

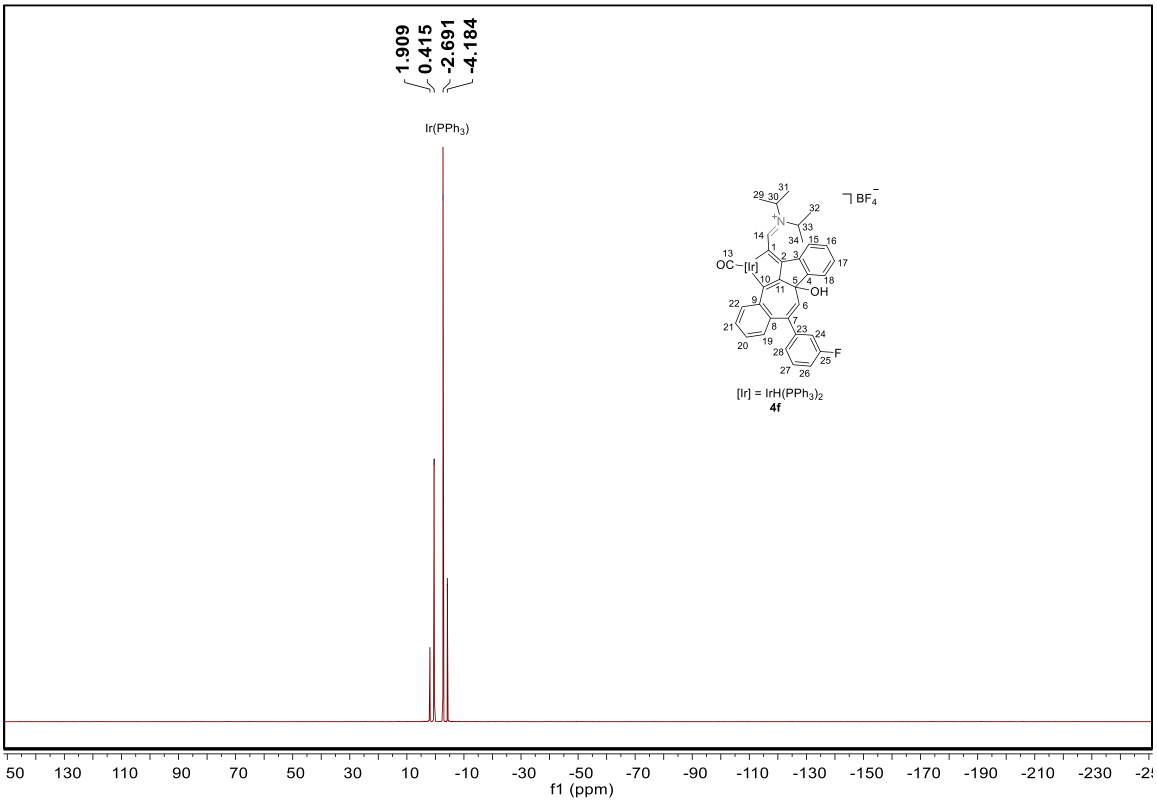

Supplementary Figure 101. The $^{31}P\{^1H\}$ NMR spectrum (202.5 MHz, CD_2Cl_2) for complex **4f.**

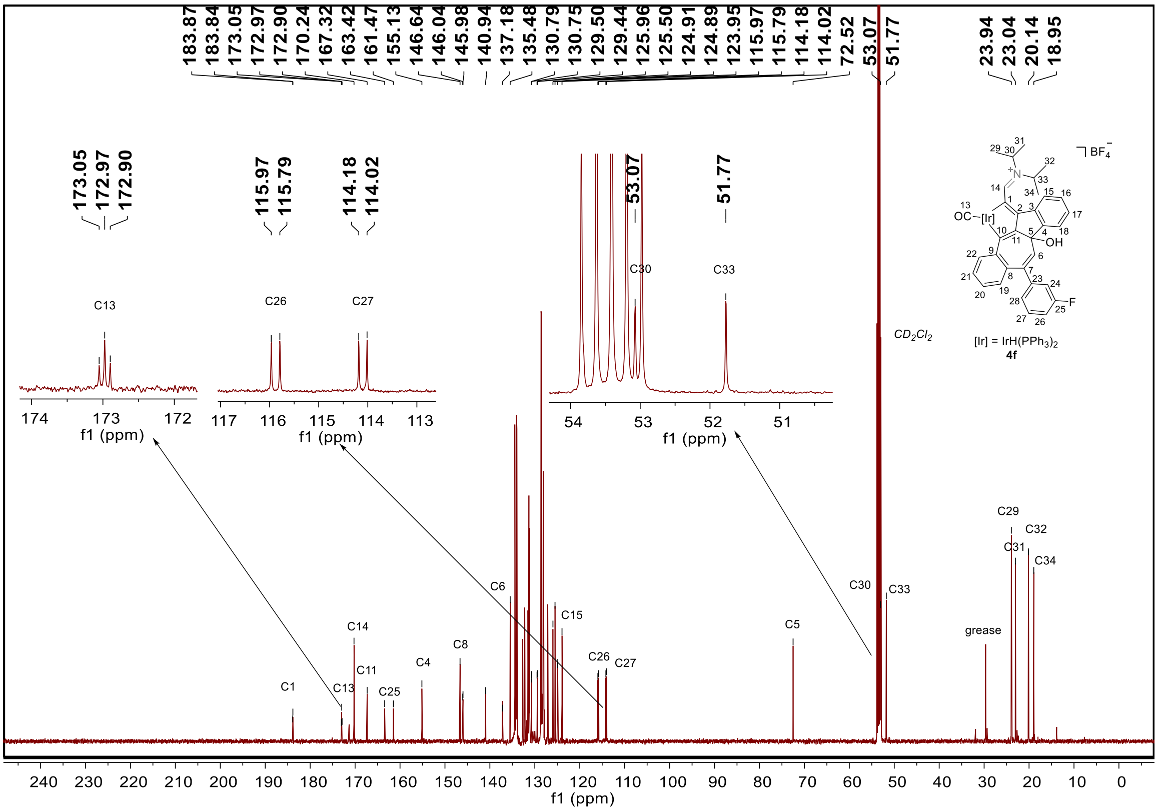

Supplementary Figure 102. The $^{13}C\{^1H\}$ NMR (125.8 MHz, CD_2Cl_2) spectrum for complex **4f**

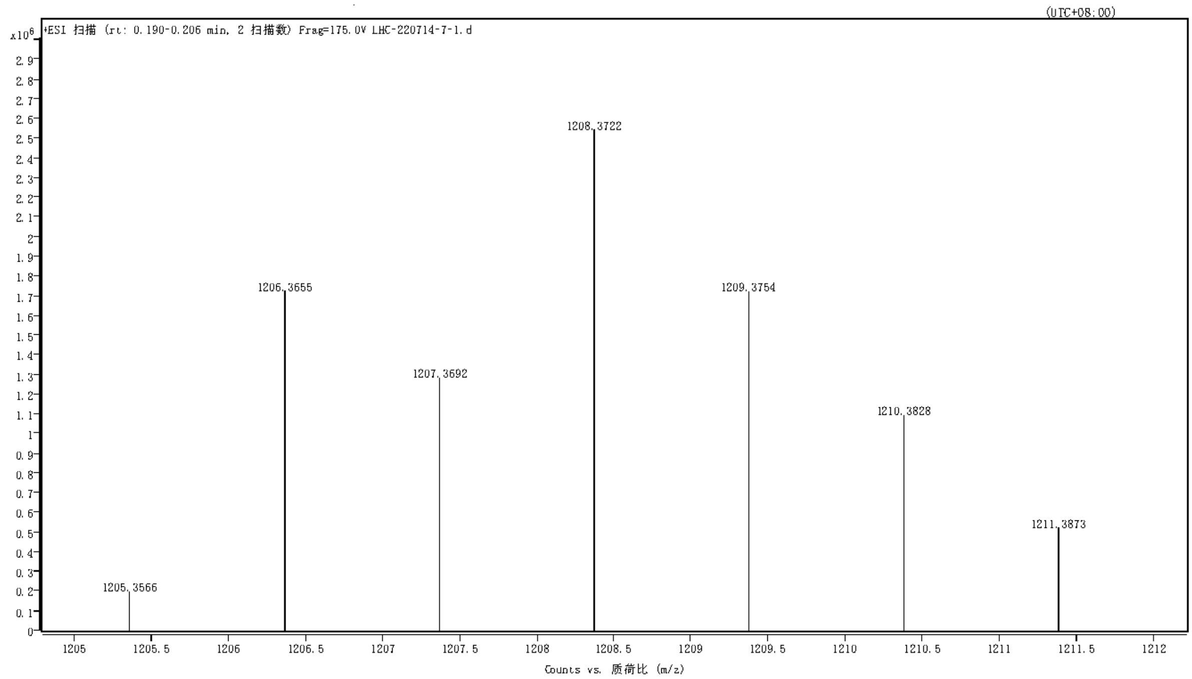

Supplementary Figure 103. Positive-ion ESI-MS spectrum for complex [**4f**]$^+$ measured in methanol

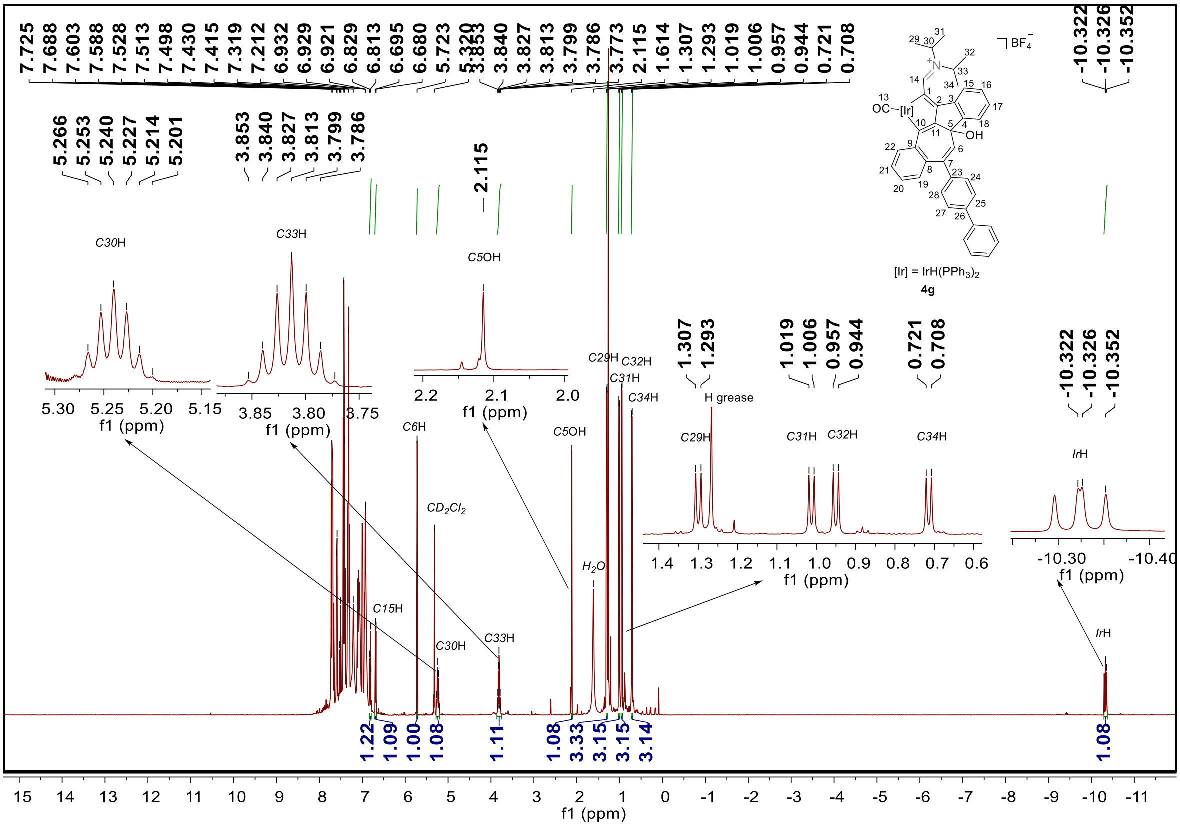

Supplementary Figure 104. The ^{1}H NMR (500.2 MHz, CD_2Cl_2) spectrum for complex **4g.**

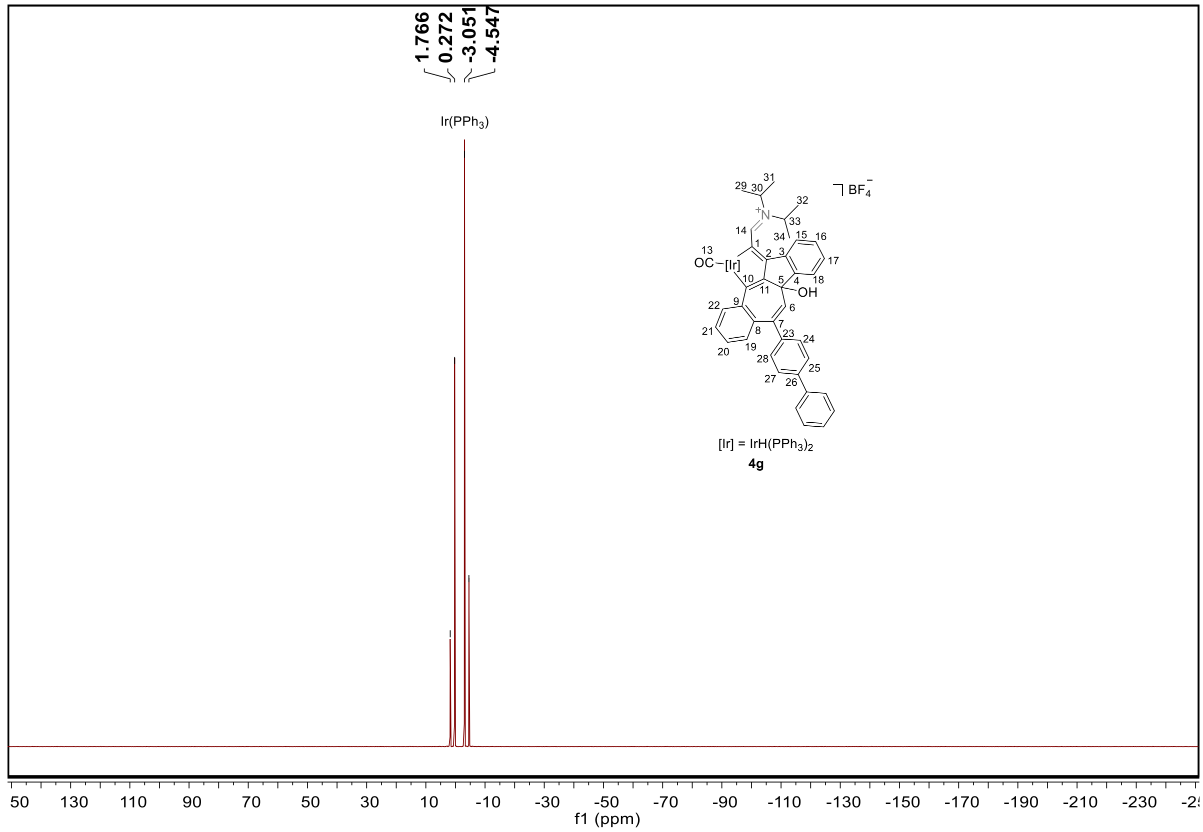

Supplementary Figure 105. The $^{31}P\{^{1}H\}$ NMR spectrum (202.5 MHz, CD_2Cl_2) for complex **4g.**

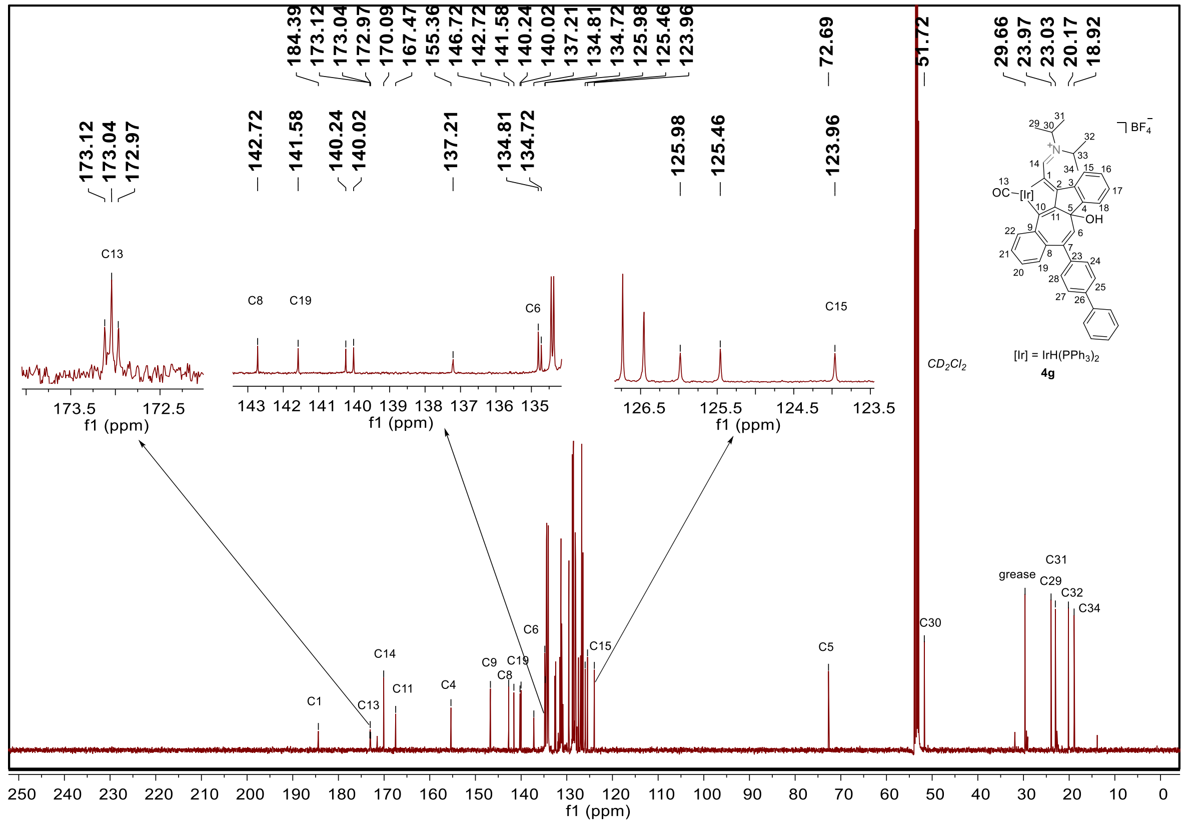

Supplementary Figure 106. The $^{13}C\{^{1}H\}$ NMR (125.8 MHz, CD_2Cl_2) spectrum for complex **4g.**

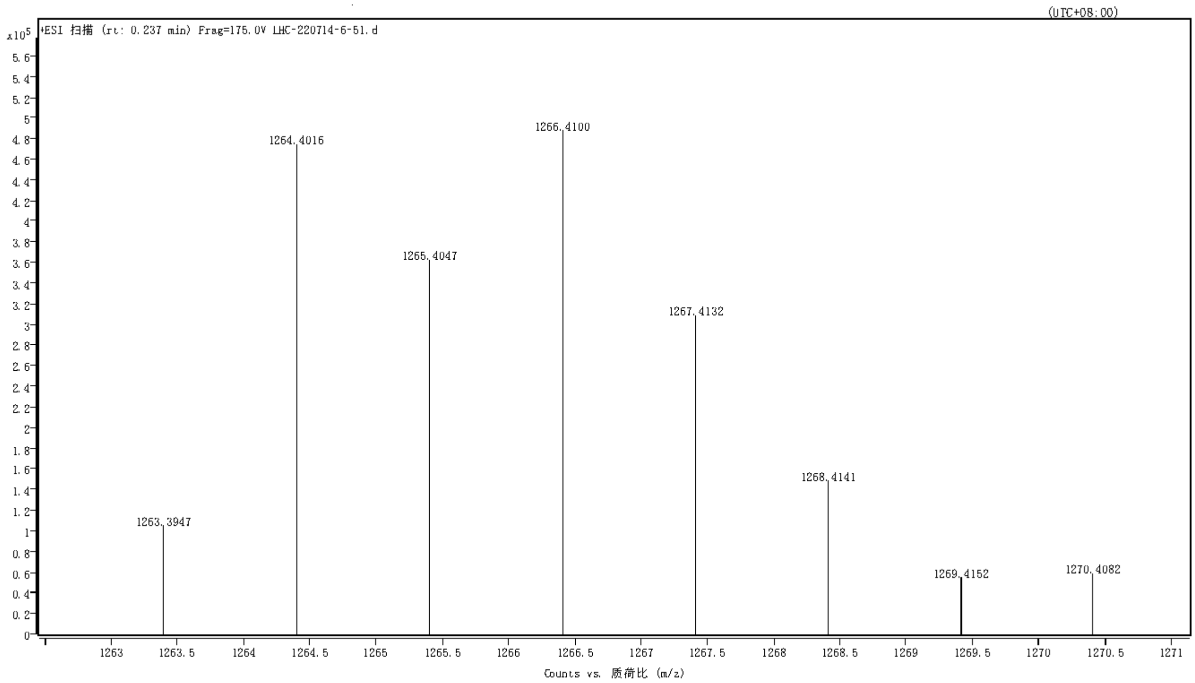

Supplementary Figure 107. Positive-ion ESI-MS spectrum for complex [**4g**]$^+$ measured in methanol.

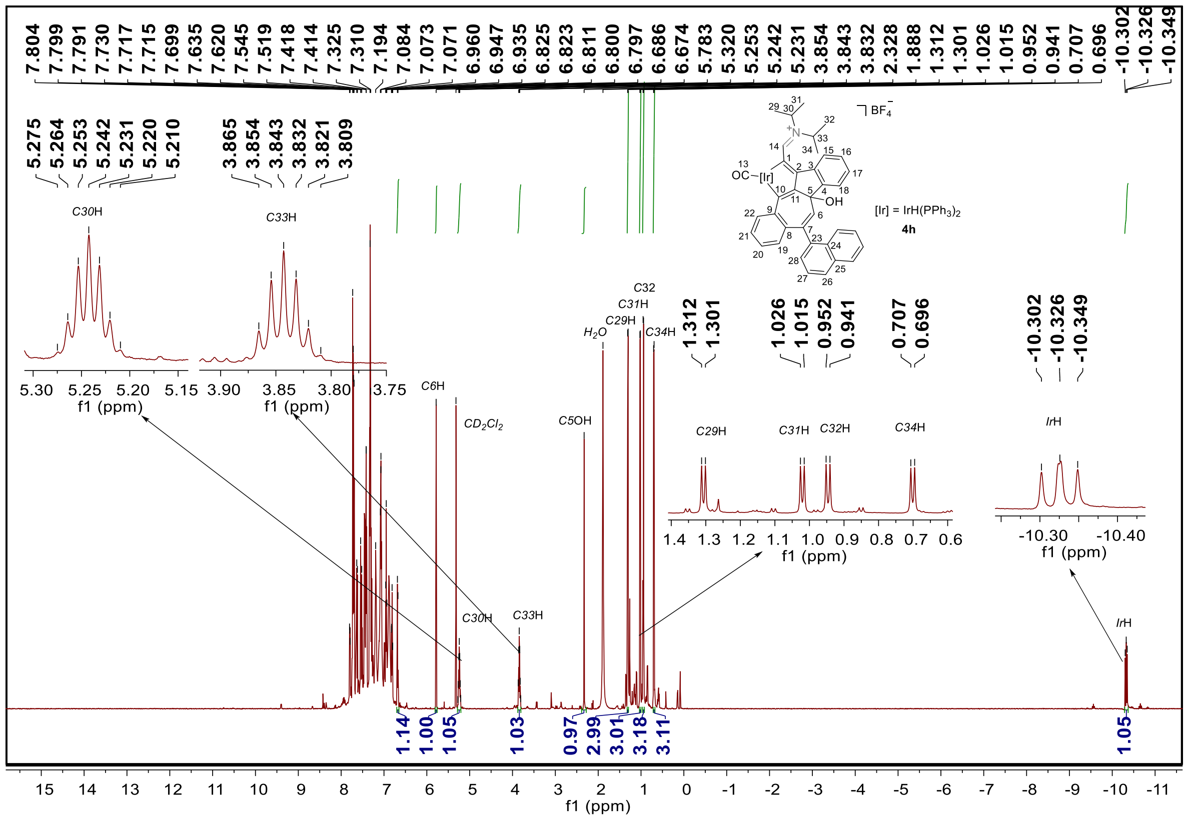

Supplementary Figure 108. The ^{1}H NMR (500.2 MHz, CD_2Cl_2) spectrum for complex **4h.**

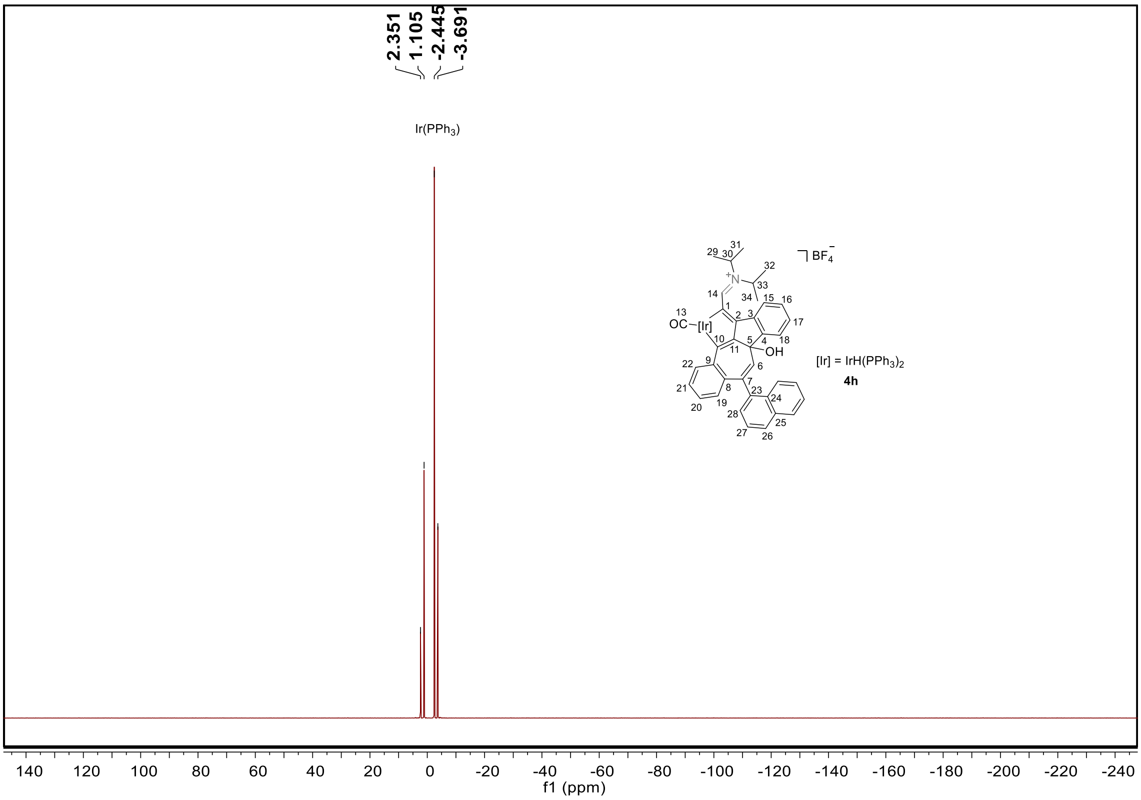

Supplementary Figure 109. The $^{31}P\{^{1}H\}$ NMR spectrum (202.5 MHz, CD_2Cl_2) for complex **4h.**

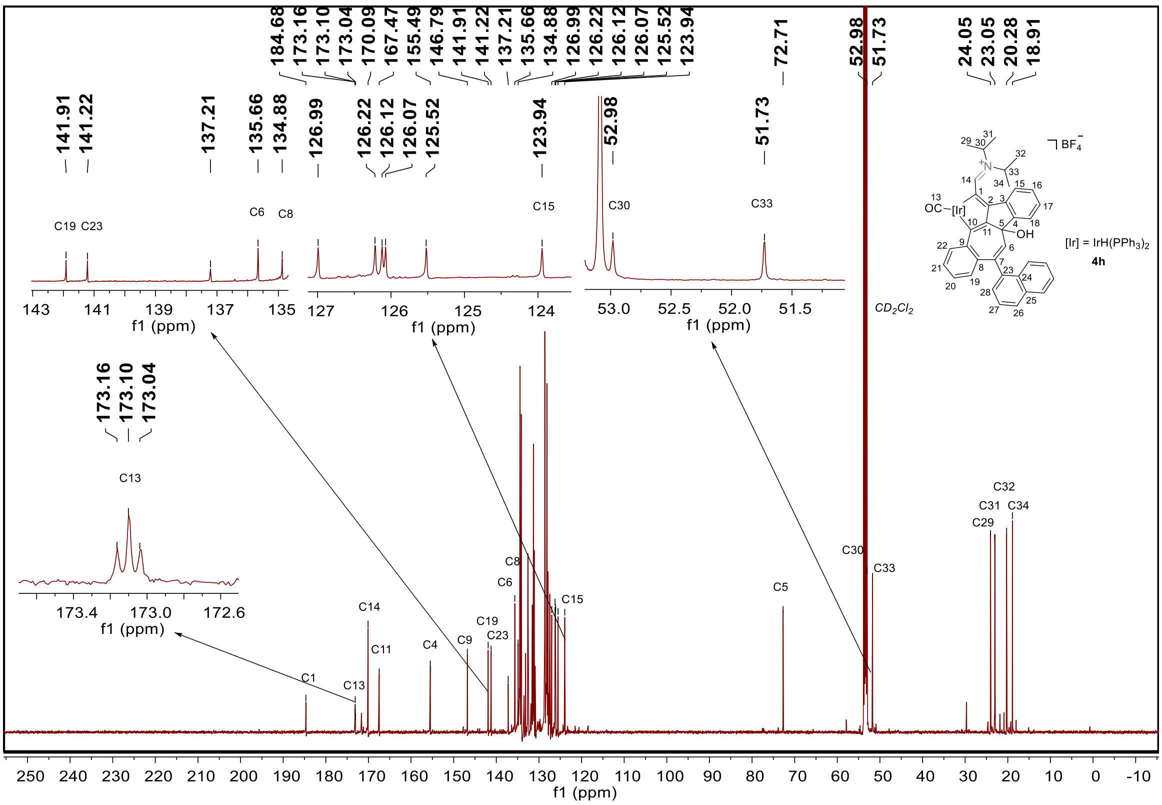

Supplementary Figure 110. The $^{13}C\{^{1}H\}$ NMR (125.8 MHz, CD_2Cl_2) spectrum for complex **4h.**

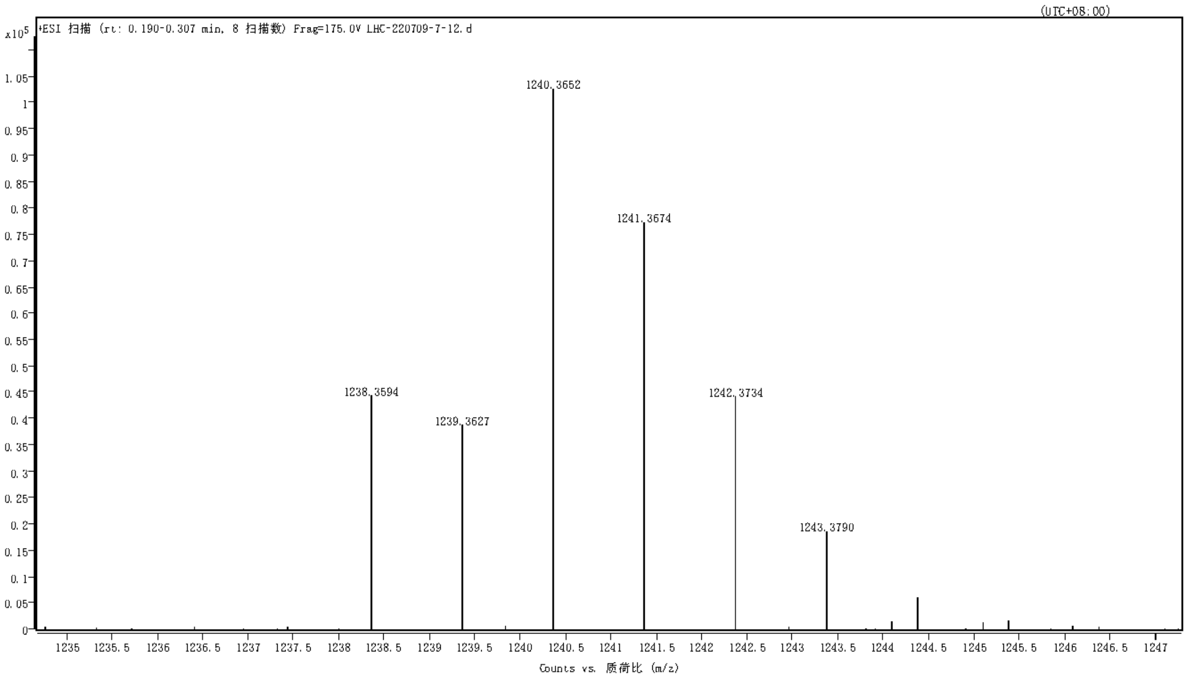

Supplementary Figure 111. Positive-ion ESI-MS spectrum for complex [**4h**]$^+$ measured in methanol

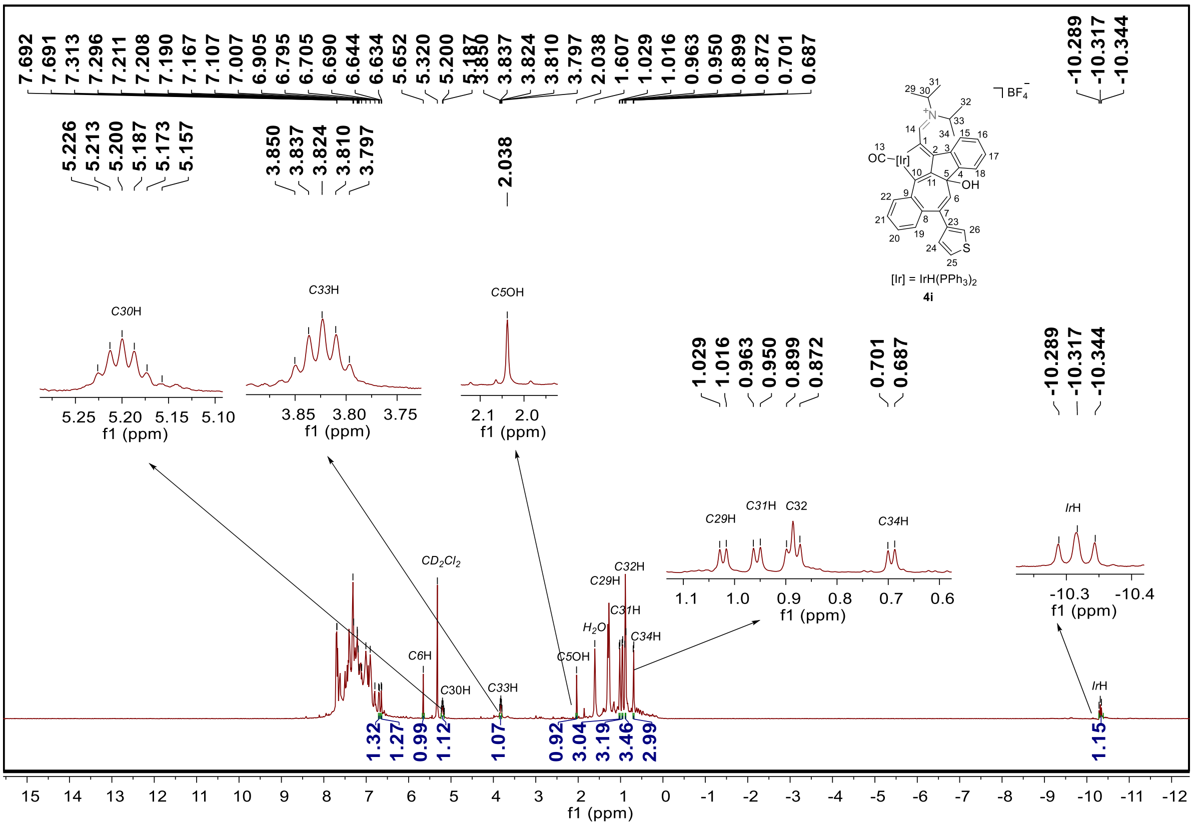

Supplementary Figure 112. The ^{1}H NMR (500.2 MHz, CD_2Cl_2) spectrum for complex **4i.**

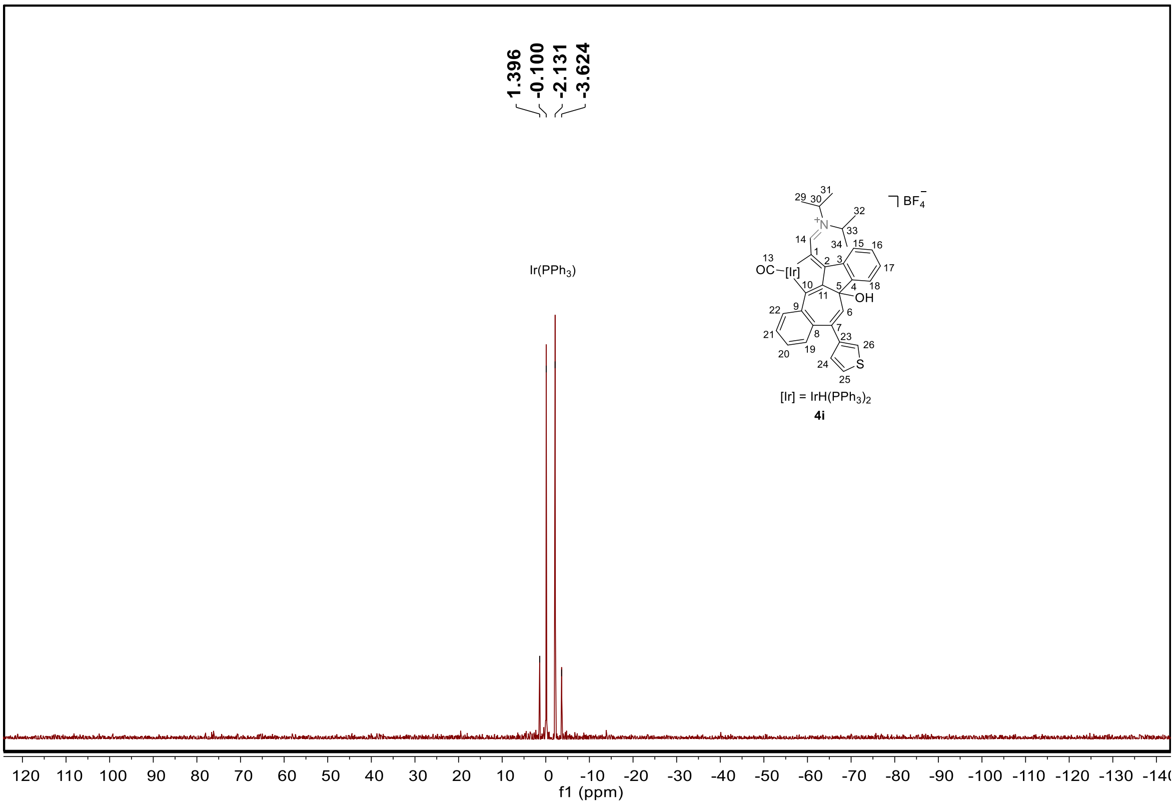

Supplementary Figure 113. The $^{31}P\{^1H\}$ NMR spectrum (202.5 MHz, CD_2Cl_2) for complex **4i.**

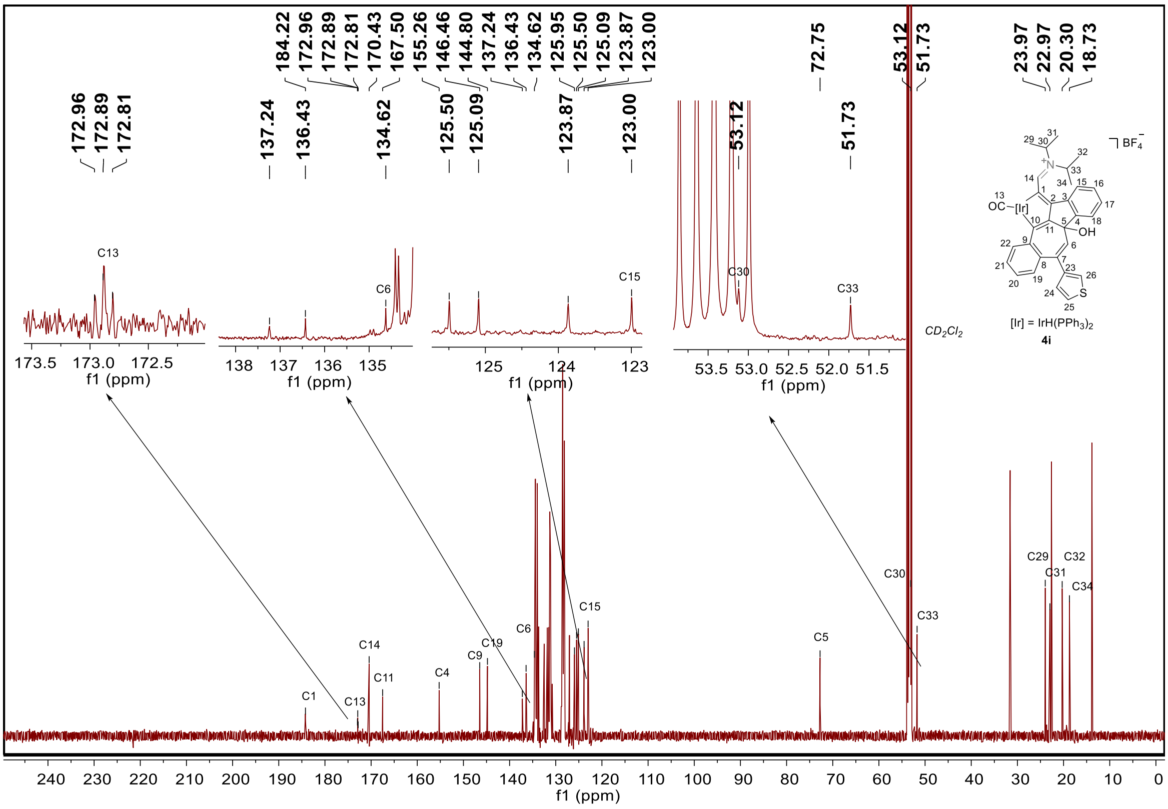

Supplementary Figure 114. The $^{13}C\{^1H\}$ NMR (125.8 MHz, CD_2Cl_2) spectrum for complex **4i.**

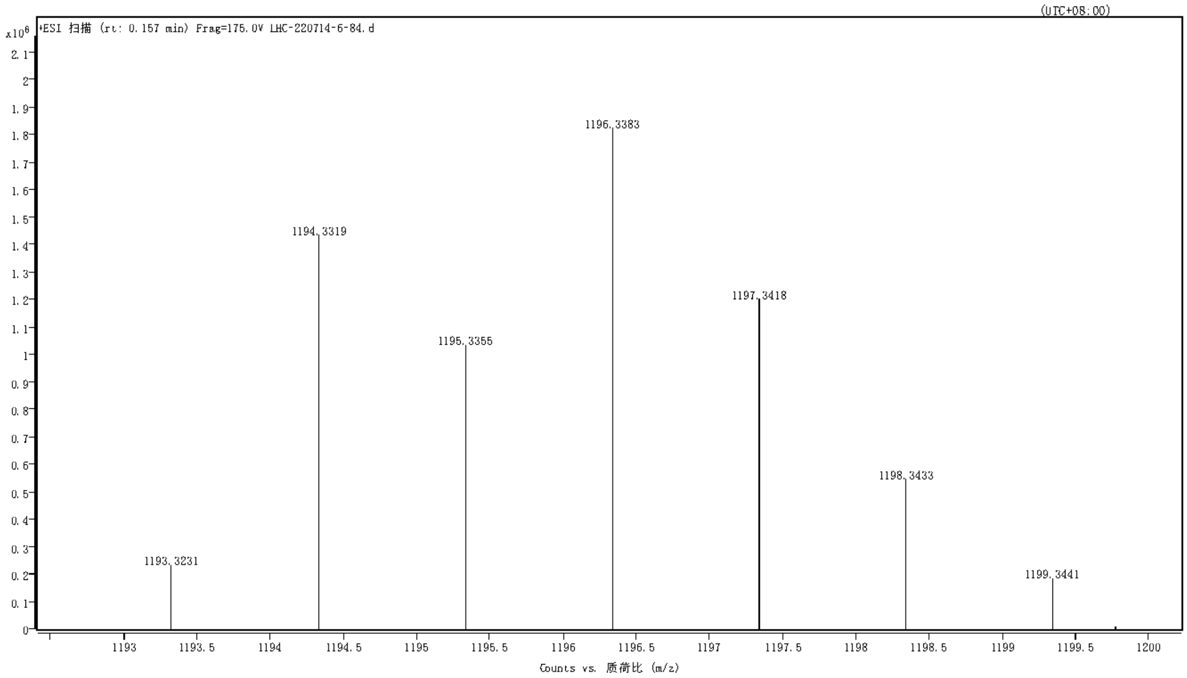

Supplementary Figure 115. Positive-ion ESI-MS spectrum for complex [**4i**]$^+$ measured in methanol

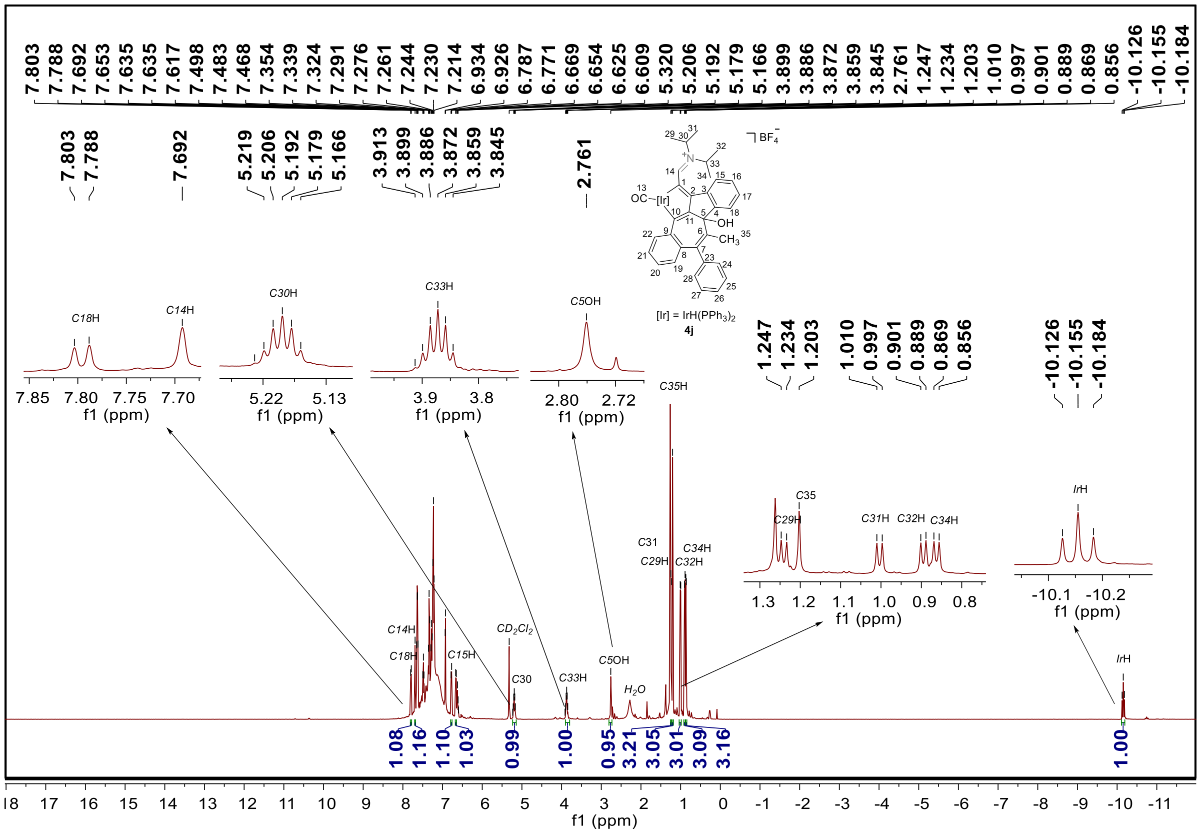

Supplementary Figure 116. The ^{1}H NMR (500.2 MHz, CD_2Cl_2) spectrum for complex **4j.**

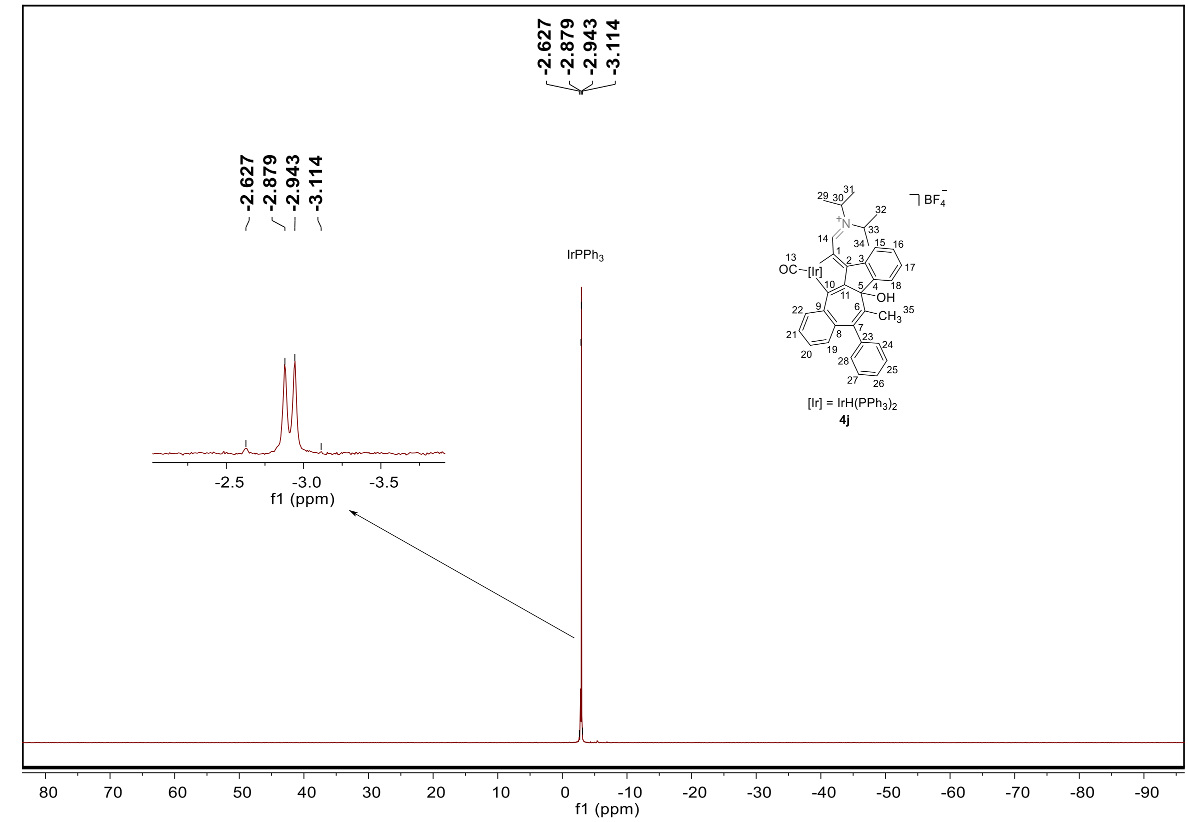

Supplementary Figure 117. The $^{31}P\{^{1}H\}$ NMR spectrum (202.5 MHz, CD_2Cl_2) for complex **4j.**

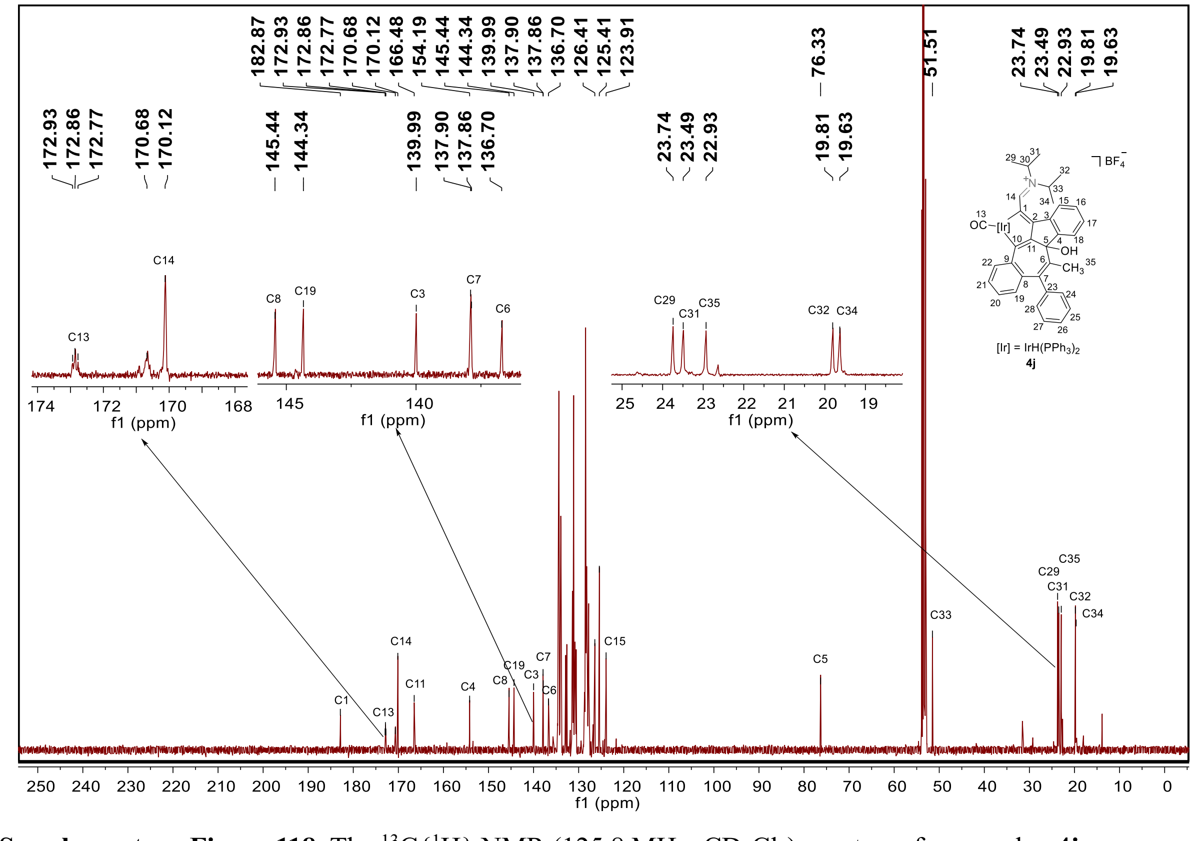

Supplementary Figure 118. The $^{13}C\{^{1}H\}$ NMR (125.8 MHz, CD_2Cl_2) spectrum for complex **4j.**

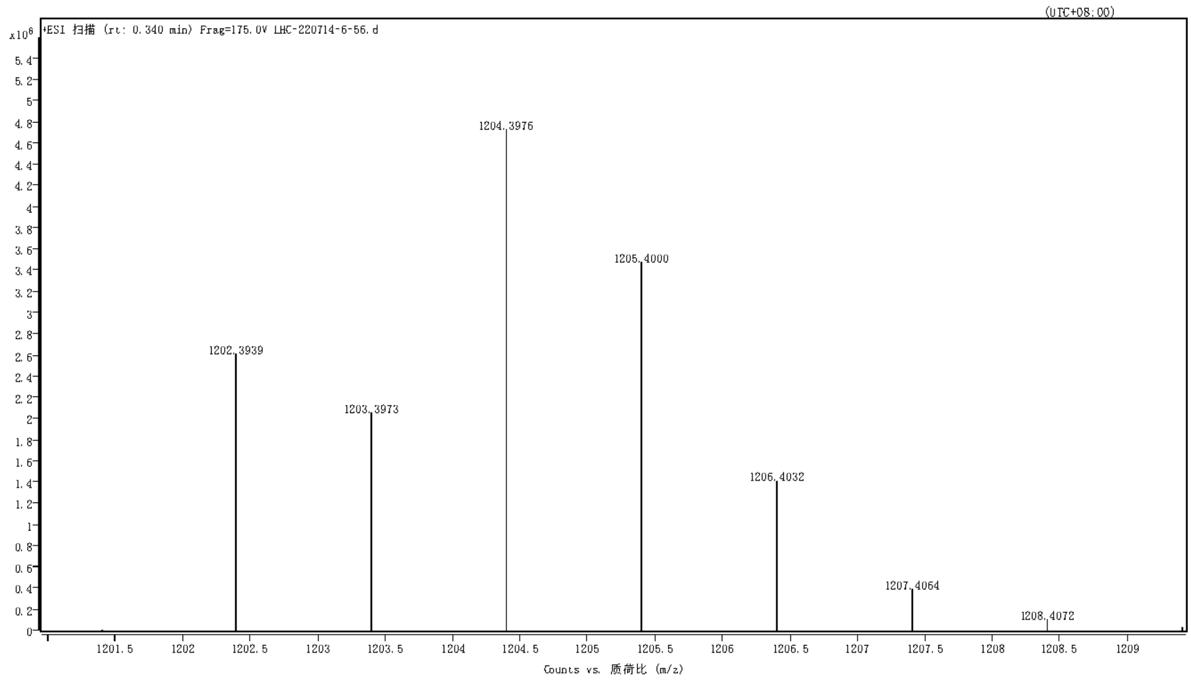

Supplementary Figure 119. Positive-ion ESI-MS spectrum for complex [**4j**]$^{+}$ measured in methanol.

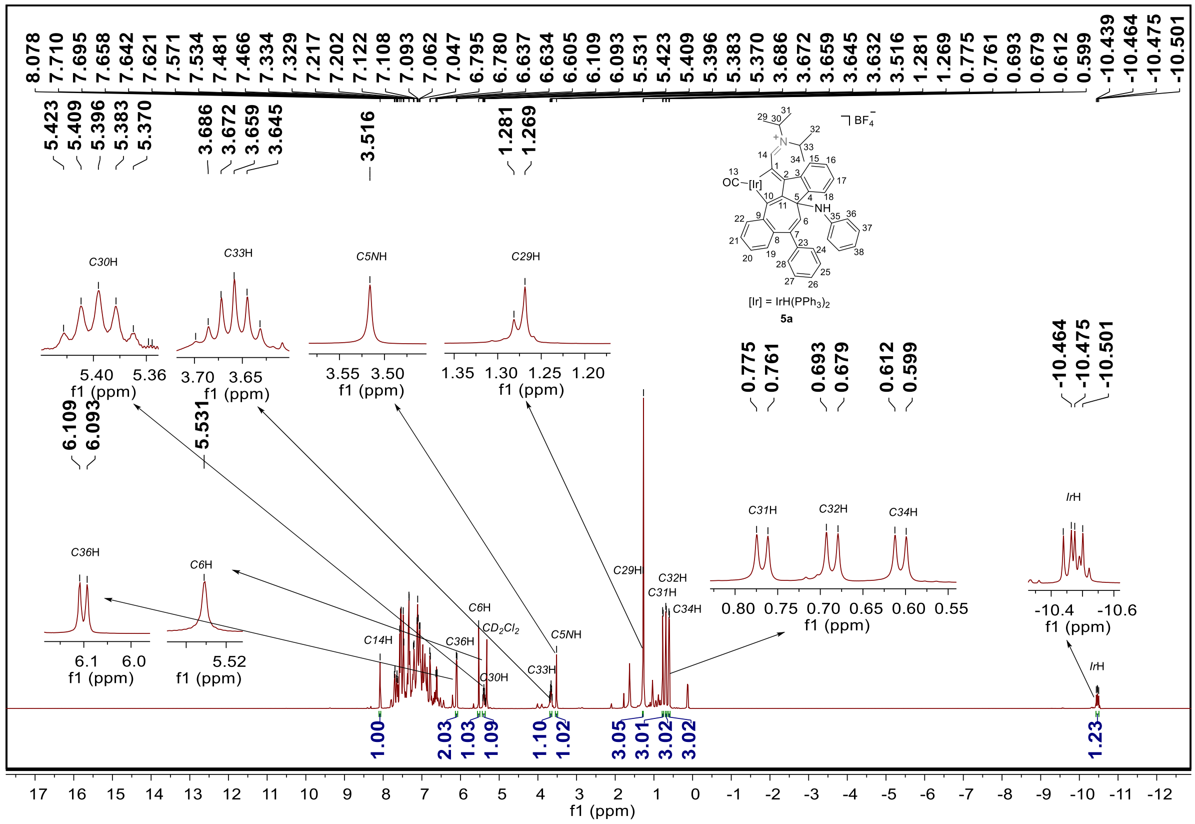

Supplementary Figure 120. The ^{1}H NMR (500.2 MHz, CD_2Cl_2) spectrum for complex **5a.**

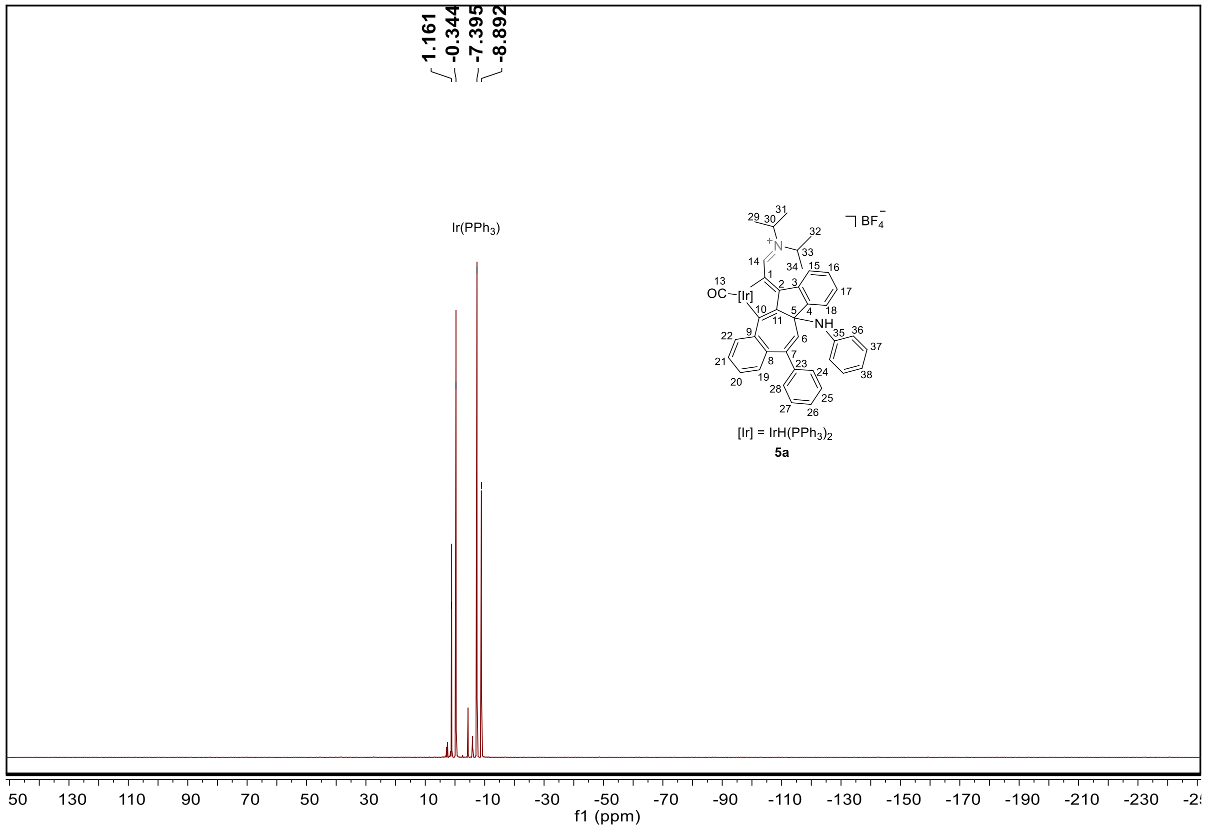

Supplementary Figure 121. The $^{31}P\{^{1}H\}$ NMR spectrum (202.5 MHz, CD_2Cl_2) for complex **5a.**

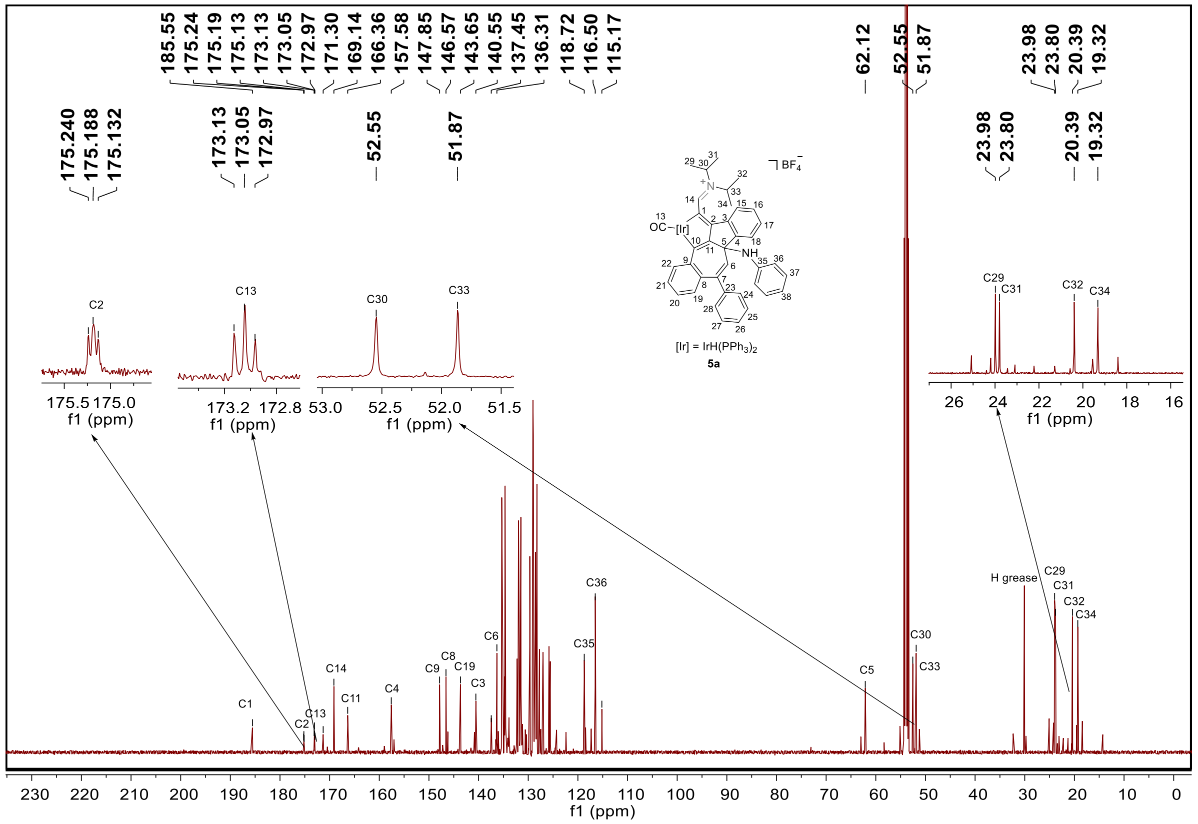

Supplementary Figure 122. The $^{13}C\{^{1}H\}$ NMR (125.8 MHz, CD_2Cl_2) spectrum for complex **5a.**

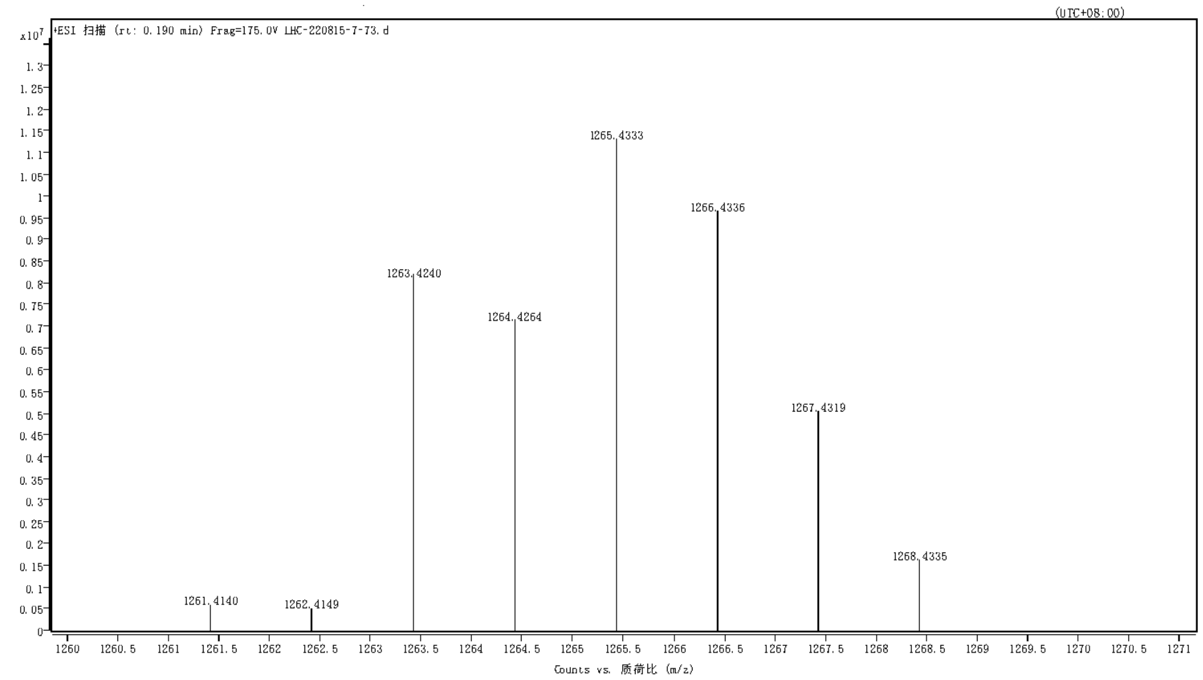

Supplementary Figure 123. Positive-ion ESI-MS spectrum for complex [**5a**]$^+$ measured in methanol

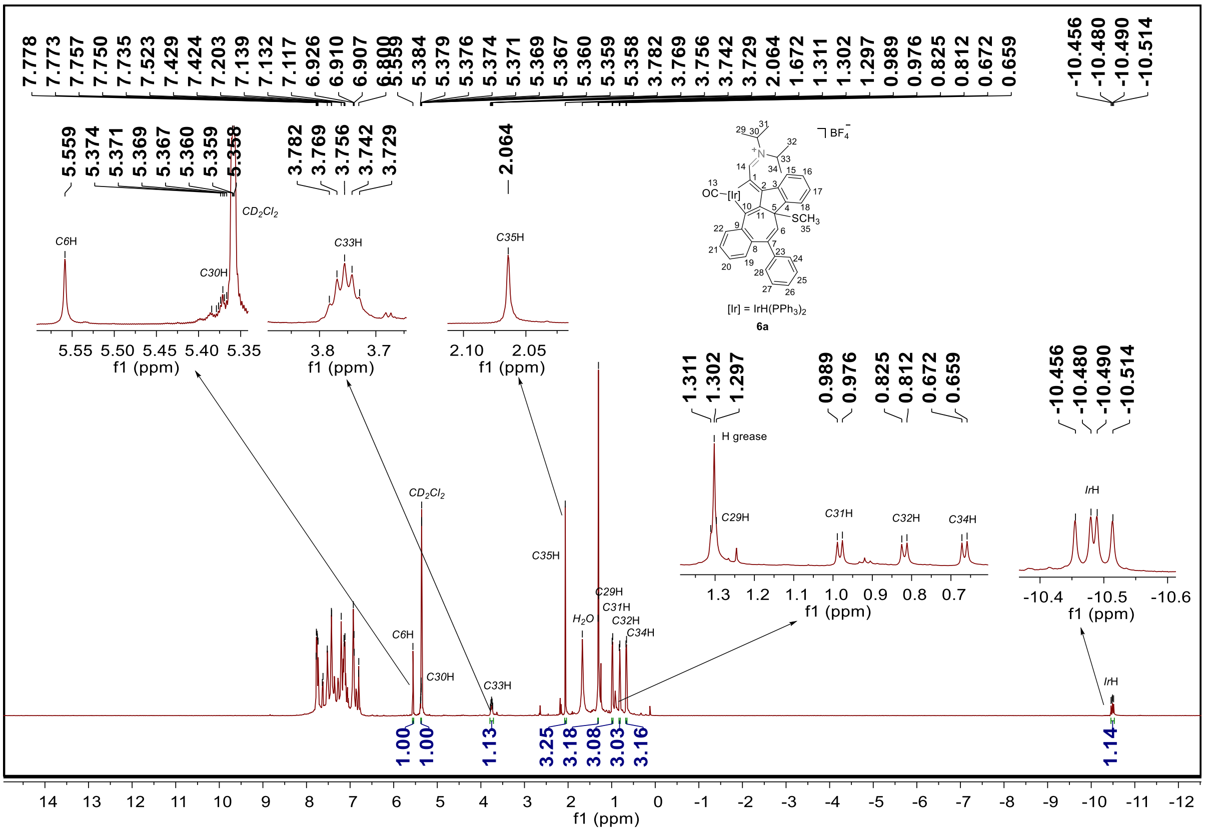

Supplementary Figure 124. The ^{1}H NMR (500.2 MHz, CD_2Cl_2) spectrum for complex **6a.**

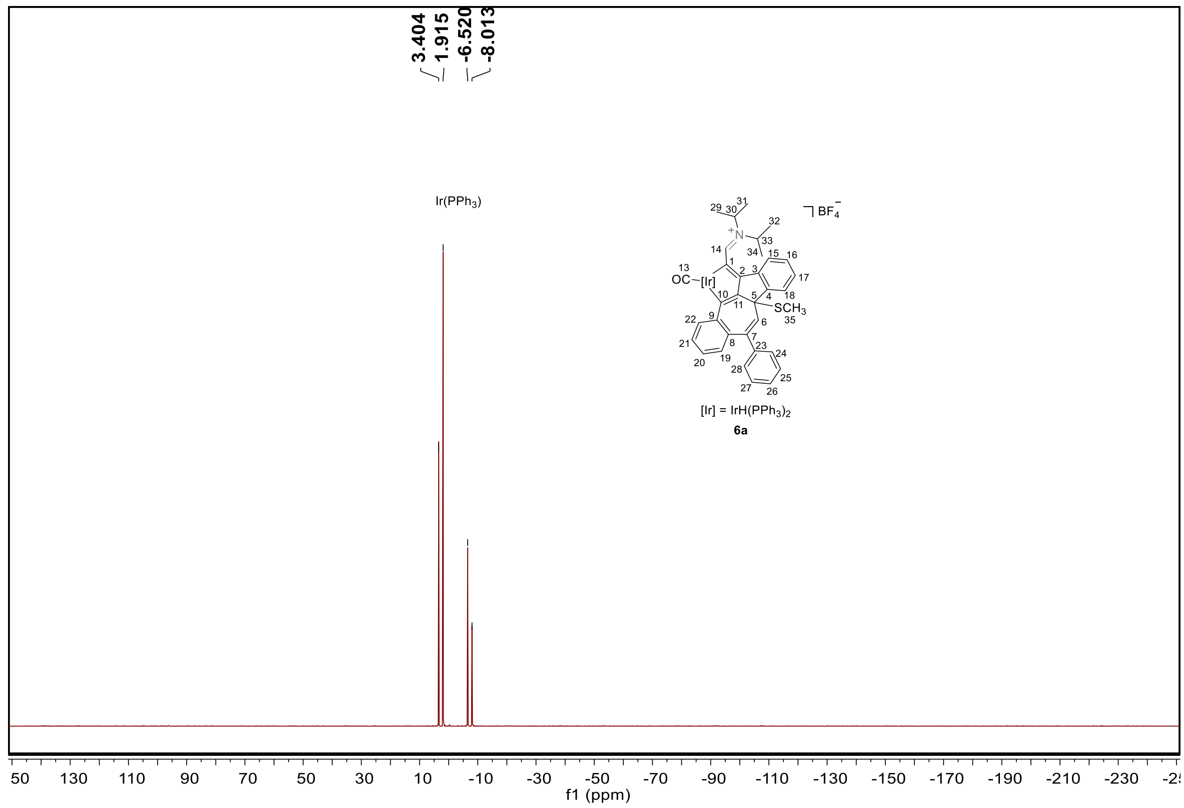

Supplementary Figure 125. The $^{31}P\{^1H\}$ NMR spectrum (202.5 MHz, CD_2Cl_2) for complex **6a.**

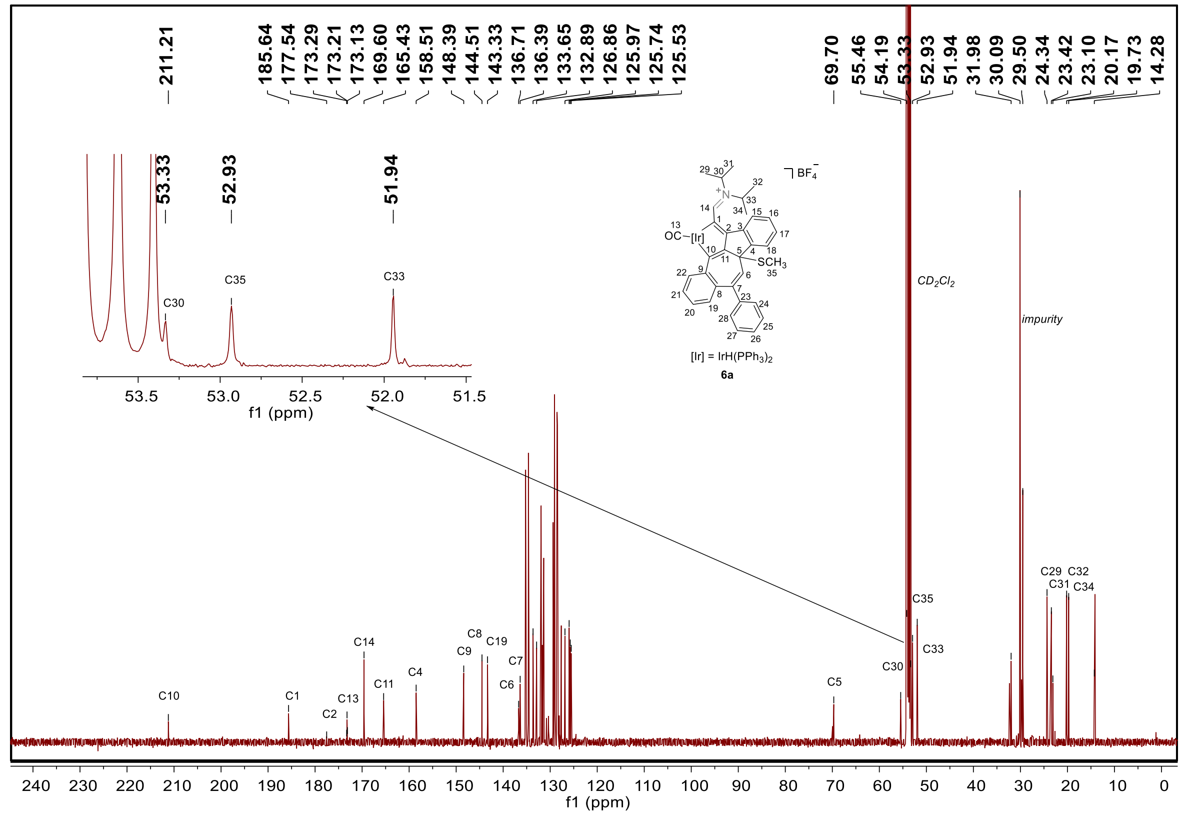

Supplementary Figure 126. The $^{13}C\{^1H\}$ NMR (125.8 MHz, CD_2Cl_2) spectrum for complex **6a.**

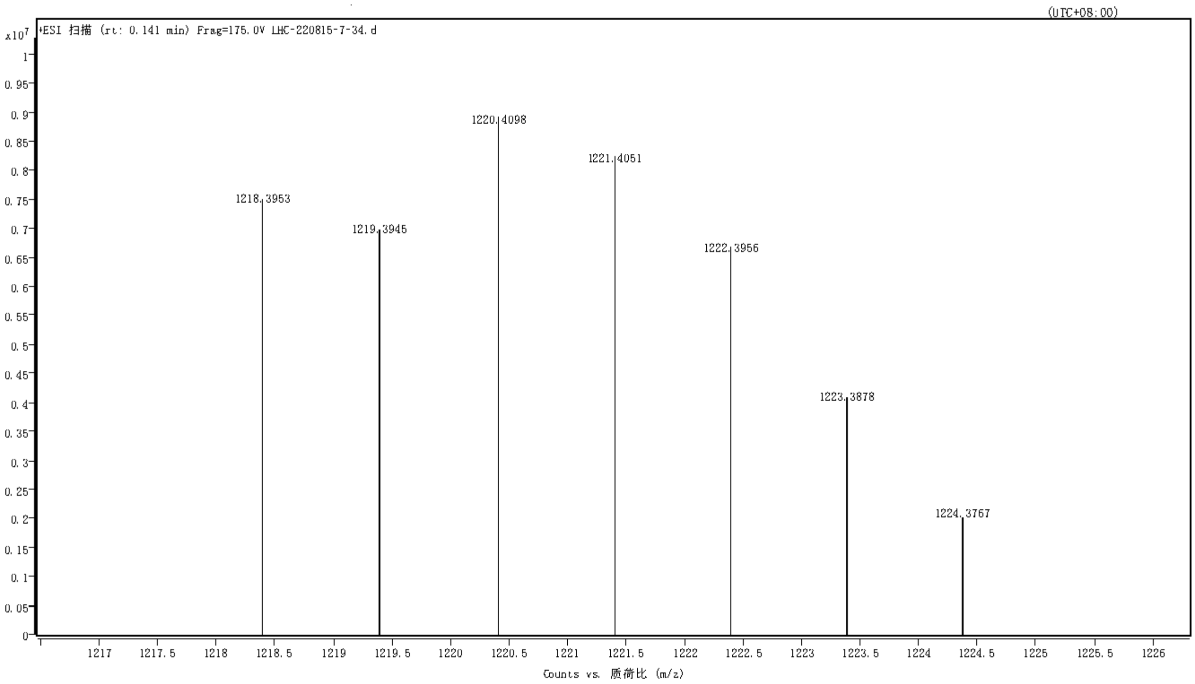

Supplementary Figure 127. Positive-ion ESI-MS spectrum for complex [**6a**]$^{+}$ measured in methanol.

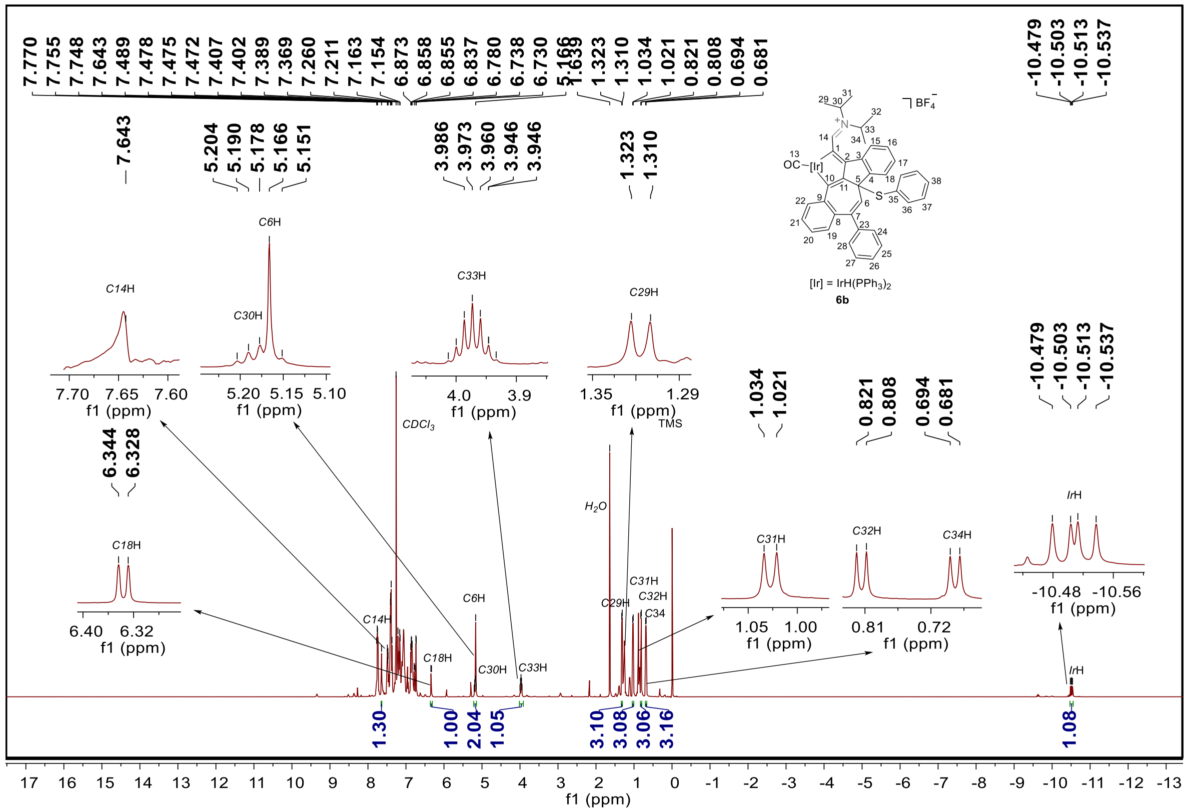

Supplementary Figure 128. The ^{1}H NMR (500.2 MHz, CD_2Cl_2) spectrum for complex **6b.**

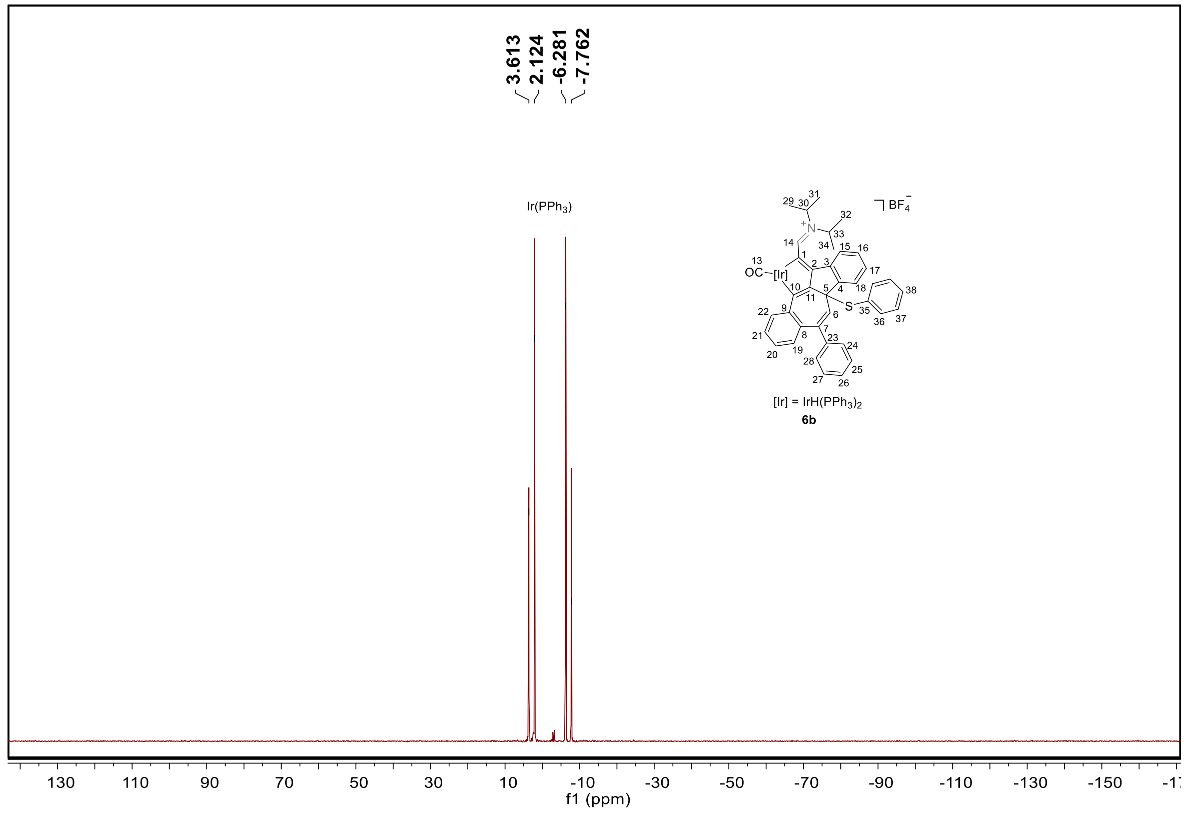

Supplementary Figure 129. The $^{31}P\{^{1}H\}$ NMR spectrum (202.5 MHz, CD_2Cl_2) for complex **6b.**

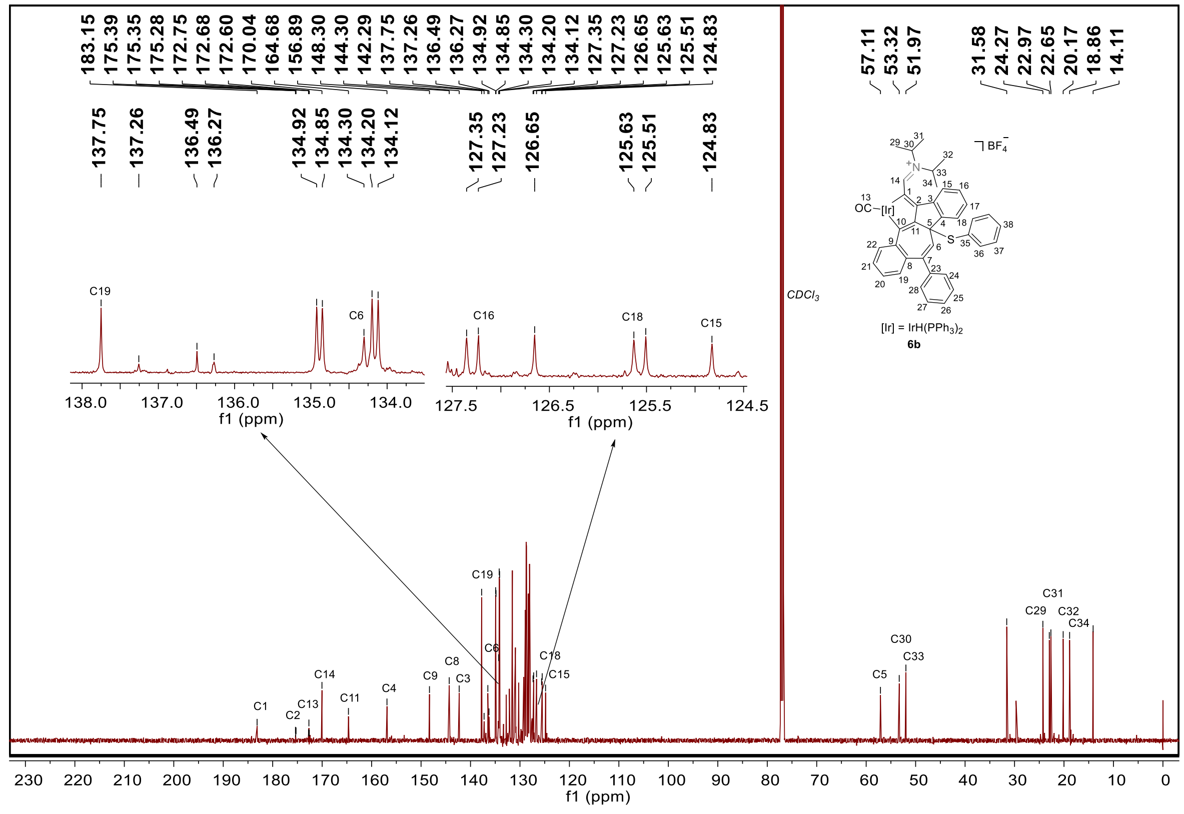

Supplementary Figure 130. The $^{13}C\{^{1}H\}$ NMR (125.8 MHz, CD_2Cl_2) spectrum for complex **6b.**

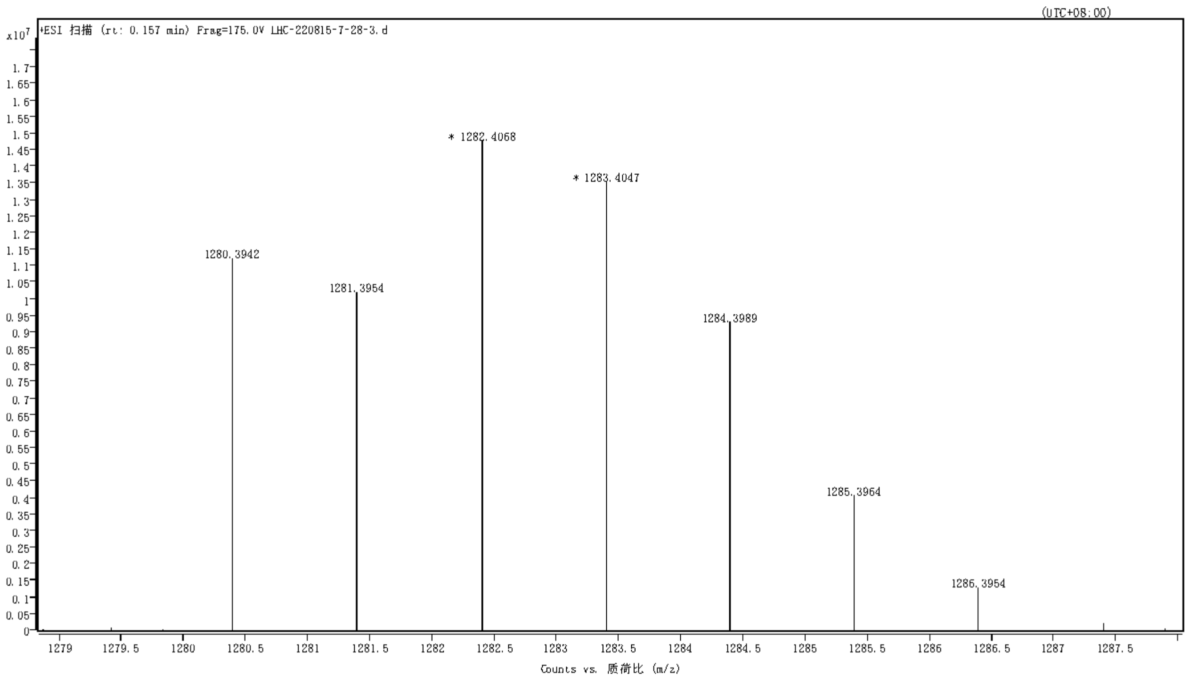

Supplementary Figure 131. Positive-ion ESI-MS spectrum for complex [**6b**]$^+$ measured in methanol.

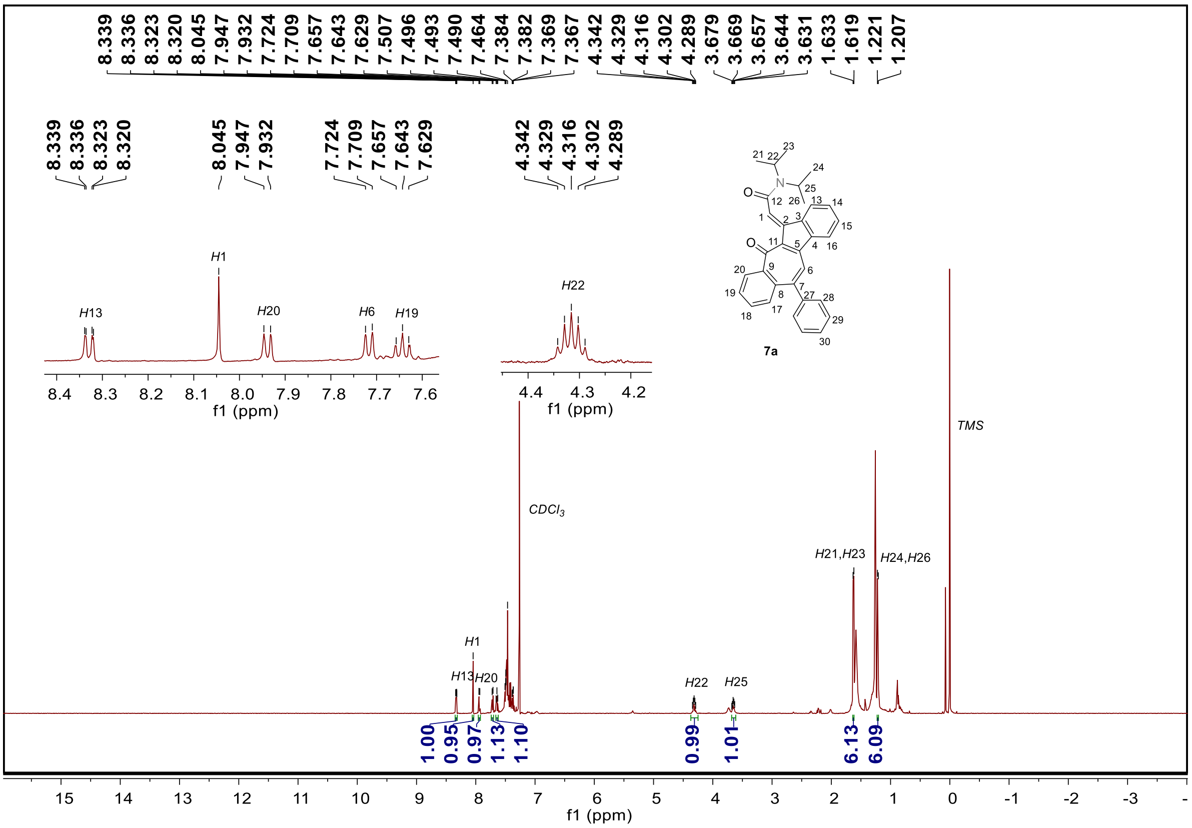

Supplementary Figure 132. The ^{1}H NMR (500.2 MHz, $CDCl_3$) spectrum for complex **7a.**

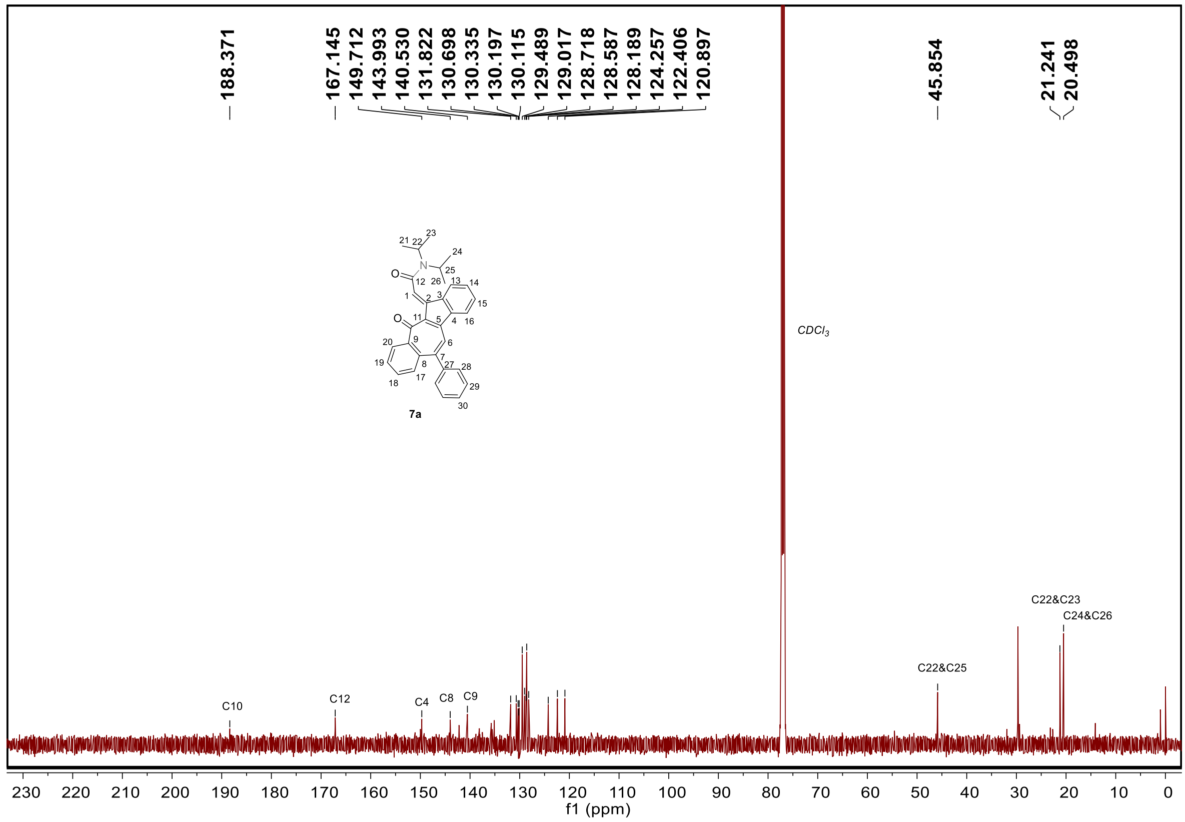

Supplementary Figure 133. The $^{13}C\{^{1}H\}$ NMR (125.8 MHz, $CDCl_3$) spectrum for complex **7a.**

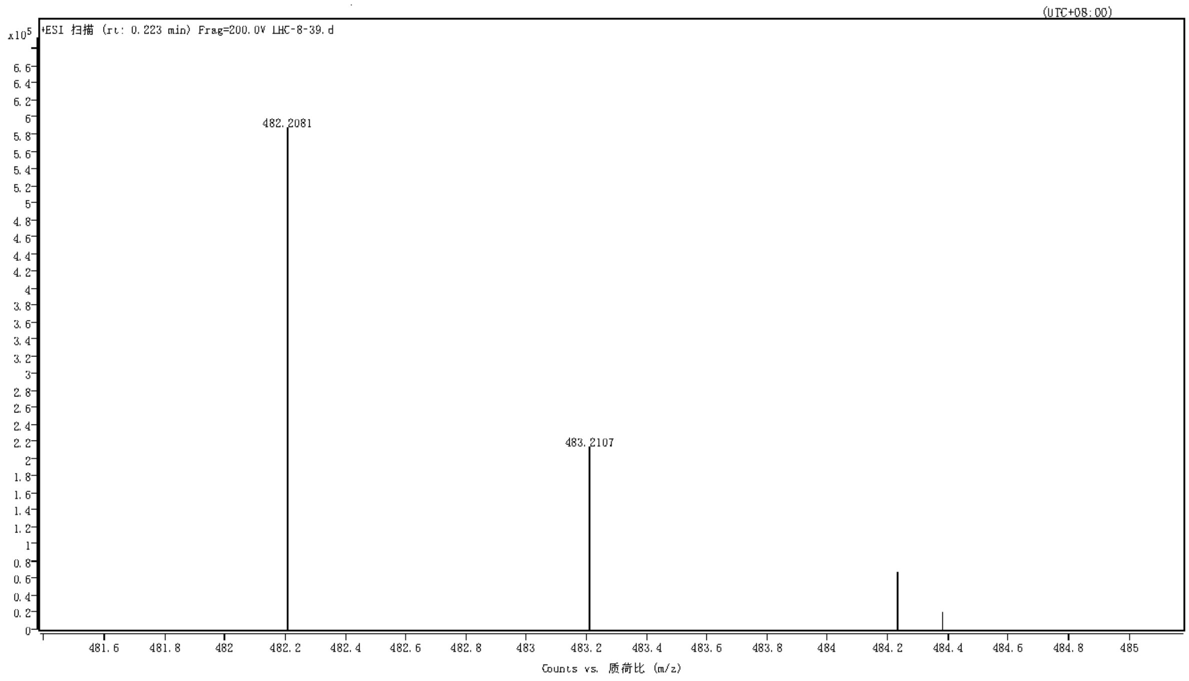

Supplementary Figure 134. Positive-ion ESI-MS spectrum for complex [**7a**+Na^{+}]$^{+}$ measured in methanol.

Supplementary references

1. Sheldrick, G. M. *SHELXTL*; Siemens Analytical X-ray Systems: Madison, Wisconsin, USA.

2. Schleyer, P. V. R., Maerker, C., Dransfeld, A., Jiao, H. & van Eikema Hommes, N. J. R. Nucleus-Independent Chemical Shifts: A Simple and Efficient Aromaticity Probe. *J. Am. Chem. Soc*. **118**, 6317-6318 (1996).

3. Chen, Z., Wannere, C. S., Corminboeuf, C., Puchta, R. & Schleyer, P. Nucleus-independent chemical shifts (NICS) as an aromaticity criterion. *Chem. Rev*. **105**, 3842-3888 (2005).

4. Fallah-Bagher-Shaidaei, H., Wannere, C. S., Corminboeuf, C., Puchta, R. & Schleyer, P. Which NICS aromaticity index for planar pi rings is best? *Org. Lett.* **8**, 863-866 (2006).

5. Geuenich, D., Hess, K., Kohler, F. & Herges, R. Anisotropy of the induced current density (ACID), a general method to quantify and visualize electronic delocalization. *Chem. Rev*. **105**, 3758-3772 (2005).

6. Herges, R. & Geuenich, D. Delocalization of Electrons in Molecules. *J. Phys. Chem. A* **105**, 3214-3220 (2001).

7. Morell, C.; Grand, A.; Toro-Labbé, A. *J. Phys. Chem. A* **109**, 205-212 (2005).

8. Hay, P. J. & Wadt, W. R. Ab initio effective core potentials for molecular calculations. Potentials for K to Au including the outermost core orbitals. *J. Chem. Phys*. **82**, 299-310 (1985).

9. Wadt, W. R. & Hay, P. J. Ab initio effective core potentials for molecular calculations. Potentials for main group elements Na to Bi. *J. Chem. Phys*. **82**, 284-298 (1985).

10. Ehlers, A. W. et al. A set of f-polarization functions for pseudo-potential basis sets of the transition metals Sc-Cu, Y-Ag and La-Au. *Chem. Phys. Lett.* **208**, 111-114 (1993).

11. Check, C. E. et al. Addition of Polarization and Diffuse Functions to the LANL2DZ Basis Set for P-Block

Elements. *J. Phys. Chem. A* **105**, 8111-8116 (2001).

12. Frisch, M. J. et al. Gaussian 16 Revision A.03 (Gaussian, Inc., Wallingford CT, 2016).

13. Mokar, B. D., Huple, D. B., Liu, R-S., *Angew. Chem. Int. Ed*., **55**, 11892-11896 (2016).

14 J. Wu, et al. Computational Study on the Relative Reactivities of Cobalt and Nickel Amidinates via β-H Migration. *Organometallics*, **26**, 2803-2805 (2007).

15 Chen, S., Yuan, F., Zhao, H. & Li, B. tert-BuOK-Catalyzed condensation of ethyl diazoacetate to aldehydes and palladium-catalyzed 1,2-hydrogen migration for the synthesis of β-ketoesters under solvent-free conditions. *RSC Adv*. **3**, 12616-12620 (2013).

16 Veltheer, J. E., Burger, P. & Bergman, R. G. Synthesis and Chemistry of the Aryliridium(III) Fluorides Cp'Ir(PMe_3)(Aryl)F: High Reactivity due to Surprisingly Easy Ir-F Ionization. *J. Am. Chem. Soc.* **117**, 12478-12488 (2002).

17 Fawcett, J., Harding, D. A., Hope, E. G., Singh, K. & Solan, G. A. Stabilisation of iridium (III) fluoride complexes with NHCs. *Dalton Trans* **39**, 10781-10789 (2010).

18 Choi, J. *et al.* Net oxidative addition of C(sp^3)-F bonds to iridium via initial C-H bond activation. *Science* **332**, 1545-1548 (2011).

19 Doherty, N. M. & Hoffmann, N. W. Transition-metal fluoro compounds containing carbonyl, phosphine, arsine, and stibine ligands. *Chem. Rev.* **91**, 553-573 (2002).

20 Analytical Methods Committee. Recommendations for the definition, estimation and use of the detection limit. *Analyst*, **112**, 199-204 (1987).

21. Glendening, E. D. et al. NBO 7.0, Theoretical Chemistry Institute, University of Wisconsin, Madison, WI (2018)

22. Becke, A. D. Density-functional thermochemistry. III. The role of exact exchange. *J. Chem. Phys*. **98**,

5648-5652 (1993).

23. Miehlich, B., Savin, A., Stoll, H. & Preuss, H. Results obtained with the correlation energy density functionals of Becke and Lee, Yang and Parr. *Chem. Phys. Lett*. **157**, 200-206 (1989).

24. Lee, C. T., Yang, W. T. & Parr, R. G. Development of the Colle-Salvetti Correlation-Energy Formula into a Functional of the Electron-Density. *Phys. Rev. B: Condens. Matter Mater. Phys*. **37**, 785-789 (1988).

25. Peng, C., Ayala, P. Y., Schlegel, H. B. & Frisch, M. J. Using redundant internal coordinates to optimize equilibrium geometries and transition states. *J. Comput. Chem*. **17**, 49-56 (1996).

26. Grimme, S., Antony, J., Ehrlich, S. & Krieg, H. A consistent and accurate ab initio parametrization of density functional dispersion correction (DFT-D) for the 94 elements H-Pu. *J. Chem. Phys.* **132**, 154104 (2010).

27. Grimme, S., Ehrlich, S. & Goerigk, L. Effect of the damping function in dispersion corrected density functional theory. *J. Comput. Chem*. **32**, 1456-1465 (2011).

28. Hehre, W. J., Ditchfield, R. & Pople, J. A. Self-Consistent Molecular Orbital Methods. XII. Further Extensions of Gaussian-Type Basis Sets for Use in Molecular Orbital Studies of Organic Molecules. *J. Chem. Phys*. **56**, 2257-2261 (1972).

29. Hariharan, P. C. & Pople, J. A. The influence of polarization functions on molecular orbital hydrogenation energies. *Theor. Chim. Acta* **28**, 213-222 (1973).

30. Weigend, F. & Ahlrichs, R. Balanced basis sets of split valence, triple zeta valence and quadruple zeta valence quality for H to Rn: Design and assessment of accuracy. *Phys. Chem. Chem. Phys.* **7**, 3297-3305 (2005).

31. Huzinaga, S. Basis-Sets for Molecular Calculations. *Comput. Phys. Rep*. **2**, 281-339 (1985).

32. Adamo, C. & Jacquemin, D. The calculations of excited-state properties with time-dependent density

functional theory. *Chem. Soc. Rev*. **42**, 845-856 (2013).
